# Experimental Quantification of Halogen⋅⋅⋅Arene van der Waals Contacts

**DOI:** 10.1002/anie.202309682

**Published:** 2023-08-09

**Authors:** Andrew M. L. West, Nicholas Dominelli‐Whiteley, Ivan V. Smolyar, Gary S. Nichol, Scott L. Cockroft

**Affiliations:** ^1^ EaStCHEM School of Chemistry University of Edinburgh Joseph Black Building David Brewster Road Edinburgh EH9 3FJ UK

**Keywords:** Halogens, Molecular Balances, Molecular Recognition, Noncovalent Interactions, π Interactions

## Abstract

Crystallographic and computational studies suggest the occurrence of favourable interactions between polarizable arenes and halogen atoms. However, the systematic experimental quantification of halogen⋅⋅⋅arene interactions in solution has been hindered by the large variance in the steric demands of the halogens. Here we have synthesized molecular balances to quantify halogen⋅⋅⋅arene contacts in 17 solvents and solvent mixtures using ^1^H NMR spectroscopy. Calculations indicate that favourable halogen⋅⋅⋅arene interactions arise from London dispersion in the gas phase. In contrast, comparison of our experimental measurements with partitioned SAPT0 energies indicate that dispersion is sufficiently attenuated by the solvent that the halogen⋅⋅⋅arene interaction trend was instead aligned with increasing exchange repulsion as the halogen increased in size (ΔG_X_⋅⋅⋅_Ph_=0 to +1.5 kJ mol^−1^). Halogen⋅⋅⋅arene contacts were slightly less disfavoured in solvents with higher solvophobicities and lower polarizabilities, but strikingly, were always less favoured than CH_3_⋅⋅⋅arene contacts (ΔG_Me_⋅⋅⋅_Ph_=0 to −1.4 kJ mol^−1^).

## Graphical abstract

Halogen⋅⋅⋅arene interactions are measured in 17 different solvents using molecular balances. The Me⋅⋅⋅arene interaction was always found to be more favorable than any halogen⋅⋅⋅arene interaction. London dispersion interactions involving the larger, more polarizable halogen atoms are unable to outcompete the electrostatic and steric terms that determine the interaction energy trend.

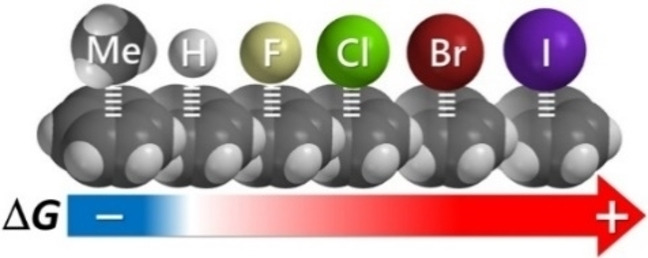

Halogen⋅⋅⋅arene interactions were first identified in the 1940 s^[1]^ and are receiving renewed attention through an emerging interest in secondary bonding interactions.^[2]^ Halogen⋅⋅⋅arene interactions have been observed in >20,000 crystal structures^[3]^ and have been implicated in supramolecular assembly^[4]^ and protein‐ligand interactions of importance to pharmaceutical design and biocatalysis.^[5]^ However, solvent effects^[6]^ and the multifaceted and directional nature of halogen interactions[^2a^, ^2b^, ^3b^, ^7^] complicates their understanding. The halogens also exhibit substantial variation in their covalent radii and atomic polarizabilities (0.60–1.36 Å and 0.56–5.35 10^−24^ cm^3^, respectively for fluorine to iodine).^[8]^ This variation modulates the balance of the attractive and repulsive components of van der Waals interactions: London dispersion vs. steric repulsion.^[9]^ Theoretical studies indicate that dispersion is the major stabilising component in halogen‐arene interactions in the gas phase, while dispersion‐correction is often required to predict interaction geometries observed in the solid state.^[10]^ Solvent effects on halogen bonds have been found to be relatively complex, and unlike H‐bonding, fail to conform to simple predictive solvation models such as the *α*/*β* H‐bond scale.[^6a^, ^11^] The energetics of interactions in solution are further complicated by the very substantial, but incomplete cancellation of dispersion due to competing interactions with the surrounding solvent.^[12]^

Here we present an experimental investigation of halogen⋅⋅⋅arene interactions. A series of Wilcox molecular torsion balances were synthesised (Figure 1),^[13]^ and thermodynamic double‐mutant cycles (Figure 2) were used to dissect the energetics of halogen‐arene interactions. The roles of solvophobic effects and competitive dispersion interactions were determined via experimental measurement in 17 solvent systems and by comparison with gas‐phase computational data (Figures 3 and 4).

**Figure 1:**
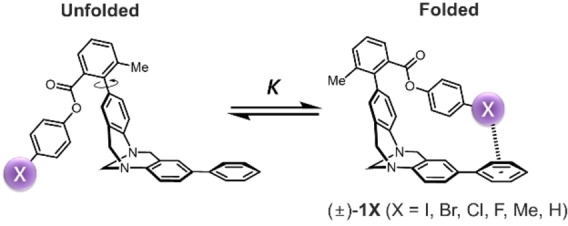
Wilcox molecular torsion balances synthesized for experimentally quantifying the energetics of “side‐on” halogen⋅⋅⋅arene van der Waals contacts. Conformational equilibrium constants, *K* were determined using ^1^H NMR spectroscopy.

**Figure 2:**
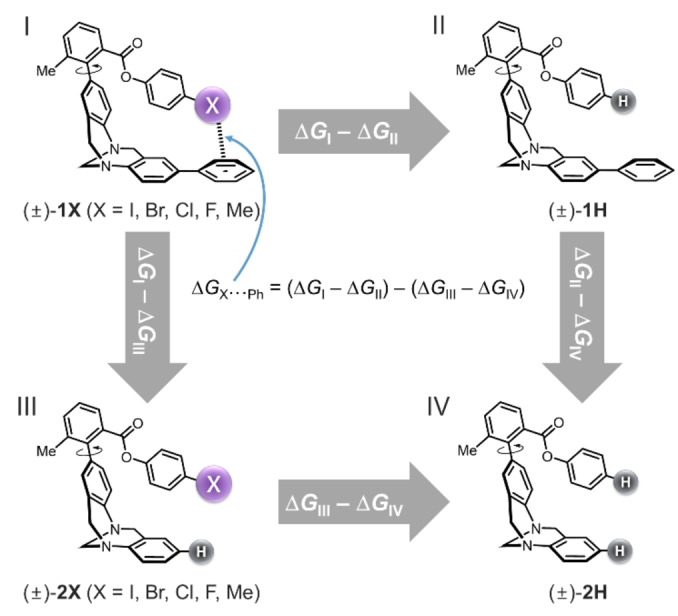
Thermodynamic double mutant cycle used to dissect the contribution of the X⋅⋅⋅arene interactions to the conformational preference for the folded conformation in balance series(±)‐**1X**. This thermodynamic dissection was applied to both the experimental and computational data depicted in Figure 3.

**Figure 3:**
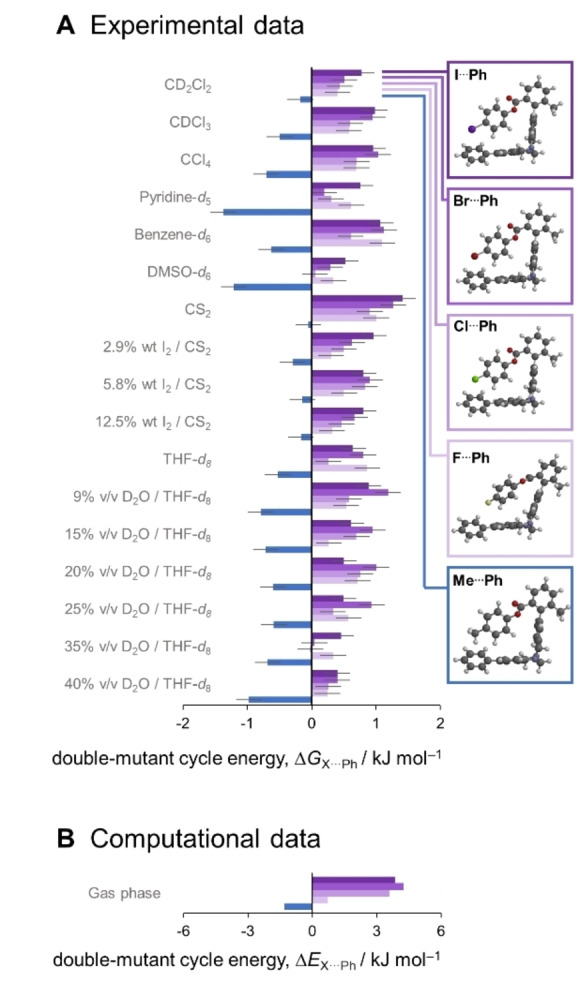
A) Experimental (300 K) and B) computed halogen⋅⋅⋅arene (purple) and CH_3_⋅⋅⋅arene (blue) interaction energies dissected using the thermodynamic double‐mutant cycle shown in Figure 2. Negative energies correspond to favourable X⋅⋅⋅arene interactions. The example inset structures were calculated using M06‐2X/def2‐TZVP. Calculated interaction energies and geometries determined using other theory/basis set combinations and X‐ray structures^[20]^ are provided in Tables S1–S6 and Figure S1–S3, Supporting Information).

**Figure 4:**
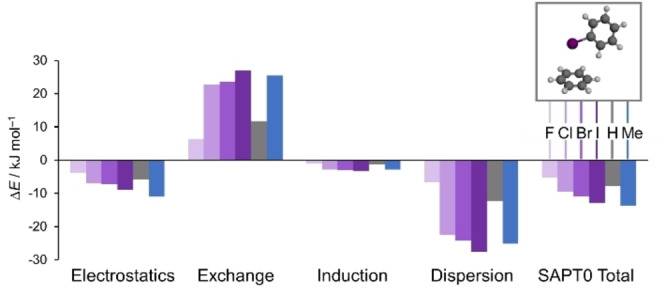
SAPT0/Jun‐cc‐pVTZ energy analysis of halogen⋅⋅⋅Ph (purple) and Me⋅⋅⋅Ph (blue) interactions based on the geometries of these interacting moieties in M06‐2X/def2‐TZVP minimized structures of the (±)‐**1X** series. SAPT0 and SAPT2 energies for geometries minimized with other theoryl/basis set combinations are provided in Section S2.2, Supporting Information.

Previous experimental studies of halogen⋅⋅⋅arene interactions have employed supramolecular complexes^[14]^ and molecular balances.^[15]^ Shimizu discovered stabilizing interactions between orthogonally orientated C−F bonds and aromatic rings,^[15c]^ which mirrored Diederich's earlier unveiling of orthogonal fluorine⋅⋅⋅amide interactions using Wilcox torsion balances.[^13^, ^16^] Both studies determined orthogonal fluorine interactions to be primarily electrostatic and dipolar in origin. However, the large variation in the radii of halogens often means that sterics dominate the energetic trends or preclude the study of the larger halogens.[^14^, ^15b^, ^15d^, ^15f^] Contrasting with prior studies in which halogen σ‐holes are pointed towards aromatic π‐clouds,[^14^, ^15c^, ^15d^] we reasoned that Wilcox molecular torsion balances[^12a^, ^12c^, ^13^, ^16^, ^17a^, ^17b^] could be adapted to instead examine near‐parallel halogen⋅⋅⋅arene contacts ((±)‐**1X** and (±)‐**2X** series in Figure 1). We anticipated this side‐on geometry would be useful for investigating dispersion in halogen⋅⋅⋅arene interactions, since the polarizable electron clouds are brought into contact, but perpendicular halogen bonds cannot form. Indeed, crystallographic and gas‐phase theoretical investigations indicate that dispersion energies are independent of the angle of contact between the halogen and the arene.^[3a][10]^ Even when a perpendicular halogen‐bonding contact is made between a halogen σ‐hole and an aromatic π cloud, the energetic contributions from electrostatics and electron delocalisation (*aka* polarisation, induction, donor‐acceptor, orbital interactions) are smaller than the dispersion component.^[10]^

Minimized structures of the (±)‐**1X** series of balances (calculated both with and without dispersion correction) consistently positioned the iodo, bromo, chloro, and methyl substituents in contact with the face of the terminal phenyl ring in the folded conformation (Figures 3 right, and Figure S3, Supporting Information). Having confirmed that the Wilcox balance framework was sufficiently flexible to accommodate halogen⋅⋅⋅arene contacts and the varied steric demands of the halogens, the (±)‐**1X** and (±)‐**2X** series of balances shown in Figure 1 were synthesized (Section S3, Supporting Information).

X‐ray crystal structures were determined for all six balances in the (±)‐**1X** series. All compound crystallized in the folded conformation (Figure S3, Supporting Information, CCDC deposition numbers: 2244208–2244213). Although the X⋅⋅⋅Ph interaction geometries in the crystal structures are influenced by crystal packing, they most closely resembled the DFT B3LYP minimizations. The crystal structures and B3LYP calculations featured longer halogen‐arene distances than those determined using computational methods that include long‐range correlation and dispersion corrections (M06‐2X, ωB97X‐D and ωB97X, Figure S3, Supporting Information).

The equilibrium folded/unfolded conformational ratio, *K* was determined for all 12 molecular balances in the (±)‐**1X** and (±)‐**2X** series (X=H, F, Cl, Br, I, Me) in 17 solvents and solvent mixtures using ^1^H NMR spectroscopy at 300 K (Section S1, Supporting Information). The conformational free energy differences between the folded and unfolded conformers were determined from ▵*G*=−*RT* ln*K*. All balances preferred the folded conformer in all solvents examined (Figure S1 and Tables S1–S2, Supporting Information). However, this conformational preference is not solely governed by the halogen⋅⋅⋅arene interactions. Hence, the thermodynamic double‐mutant cycle and equation shown in Figure 2 was used to dissect the contribution of the halogen⋅⋅⋅arene interactions (and associated solvent effects) to the position of the conformational equilibrium.[^12a^, ^16^, ^18^]

The dissected experimental Δ*G*_X⋅⋅⋅Ph_ values shown in Figure 3A reveal small interaction energies and weak solvent effects. All halogen substituents formed weaker interactions with the terminal phenyl ring (Δ*G*_X⋅⋅⋅Ph_=0 to +1 kJ mol^−1^) relative to the case where X=H. The I⋅⋅⋅Ph interaction was generally less favored than the F⋅⋅⋅Ph interaction. However, the F⋅⋅⋅Ph interaction was preferentially weakened by H‐bond acceptors such as pyridine‐*d*_5_, DMSO‐*d*_6_, and THF‐*d*_8_/D_2_O, presumably due to the edges of the fluorophenyl rings being better H‐bond donors than those bearing less electronegative halogens. In stark contrast to the disfavoured halogen⋅⋅⋅arene interactions, the Me⋅⋅⋅Ph interactions were universally favoured (Δ*G*_Me⋅⋅⋅Ph_=0 to −1.4 kJ mol^−1^). This measurement is similar to the −1.8 kJ mol^−1^ determined for the Me⋅⋅⋅Ph interaction by Wilcox and co‐workers in their seminal molecular torsion balance, albeit in a different geometry.^[13b]^ Although it is possible that solvent effects might be cancelled out by the double‐mutant cycle analysis, the Δ*G* values of the (±)‐**1X** series taken in isolation reveal the same major trends: there is little variation as the halogen is changed, while the balance hosting the Me⋅⋅⋅Ph interaction consistently presents the strongest preference for the folded conformation (Figure S1, Supporting Information).

The experimental interaction trends were reproduced via double‐mutant cycle analysis of the calculated energies of the minimized structures at several levels of theory, even when lower levels of theory and small basis sets were used (Figure 3B and Figure S2, Supporting Information). SAPT0 energy decomposition calculations provided further insight into the origins of the energetic trends (Figure 4 and Section S2.2, Supporting Information).^[17]^ The decreasing stability of the halogen⋅⋅⋅arene interactions as the halogens increase in size is mirrored in only the steric (exchange) component. Interestingly, the Me⋅⋅⋅Ph interaction has similar van der Waals components (dispersion and exchange) as the I⋅⋅⋅Ph interaction, but a more favourable electrostatic contribution. These dominant electrostatic and steric effects are also consistent with the limited solvent effects seen in Figure 3A and reproduced in the gas‐phase computational results in Figures 3B and S2, Supporting Information.

Gas‐phase computed energies may be useful for understanding energetic trends across a series of closely related compounds, but the accurate calculation of solvent effects remains an unsolved challenge. In contrast, even subtle solvent effects can be measured experimentally using molecular balances. Initially, we sought to examine solvophobic influences by the addition of D_2_O to deuterated tetrahydrofuran (THF‐*d*_8_, Figure 3A, bottom). Upon the addition of D_2_O, the Me⋅⋅⋅Ph and I⋅⋅⋅Ph interactions were more stabilized than those involving smaller halogens. Similarly, the most disfavoured halogen⋅⋅⋅Ph interactions were found in less solvophobic solvents such benzene, CS_2_, and CCl_4_. However, the solvophobic effect was insufficient to make any of the halogen⋅⋅⋅Ph interactions favourable (up to 40 % v/v D_2_O), and there was only limited evidence of the larger (more solvophobic) I⋅⋅⋅Ph contact being slightly less disfavoured than those involving smaller halogens. Moreover, these energetic trends cannot be wholly attributed solvophobic effects since they could also arise from changes in competitive dispersion interactions with the solvent; increasing water content decreases bulk polarizability, while less solvophobic apolar solvents tend to have higher bulk polarizabilities (e.g. CS_2_ and benzene).[^12c^, ^19^] Hence, we next examined whether halogen⋅⋅⋅Ph interactions might be weakened by increasing solvent competition for dispersion interactions by increasing the bulk polarizability of the solvent.

Counter to our expectations, adding the very polarizable molecule iodine to the most polarizable solvent, CS_2_ tended to push the folding free energies of all balances closer to zero rather than making the halogen⋅⋅⋅Ph interactions even less disfavoured (Figure 3A and Figure S1 in the Supporting Information). It is likely that adding iodine not only increased the bulk polarizability, but also increased solvent cohesion and hence the solvophobic effect; after all, iodine is a solid with a melting point of 114 °C. Nonetheless, it could still be seen that changes in the interaction energies upon varying the halogen substituent were largest in the most polarizable CS_2_/I_2_ mixtures, and smallest in the least polarizable solvent examined (THF/40 % D_2_O).

In summary, we have synthesized molecular torsion balances to measure halogen⋅⋅⋅arene interactions in a wide range of solvents. The adoption of the Wilcox balance framework and its inherent flexibility enabled the variable steric demands of the halogens to be accommodated. This enabled us to sidestep steric issues that have been widely encountered in previous experimental attempts to investigate interactions involving halogens. Side‐on halogen⋅⋅⋅arene contacts were found to be weakly disfavoured in all 17 solvents examined (0 to 1.5 kJ mol^−1^). In stark contrast, identically positioned methyl⋅⋅⋅arene interactions were weakly favourable in all solvents examined (0 to −1.4 kJ mol^−1^). SAPT energy decomposition calculations support the hypothesis that the CH_3_⋅⋅⋅arene interactions were favoured over the halogen⋅⋅⋅arene interactions due to electrostatic attraction, which is greatly diminished in halogen⋅⋅⋅arene interactions. The halogen⋅⋅⋅arene interactions, particularly those involving the larger halogens, were more sensitive than CH_3_‐arene interactions to increasing the solvophobic effect via the addition of water. However, the solvophobic effect could not be increased to sufficient extent by the addition of up to 40 % v/v D_2_O in THF‐*d*_8_ to make any halogen⋅⋅⋅arene interaction favourable. The main finding is that dispersion interactions alone are insufficient to drive the association of halogen⋅⋅⋅arene contacts in solution, even between functional groups considered to be highly polarizable. This investigation studied side‐on halogen⋅⋅⋅arene contacts; it is likely that the energetics of orthogonal halogen⋅⋅⋅arene contacts capable of forming halogen‐bonds via σ‐hole interactions will differ from those of the present study.

## Conflict of interest

The authors declare no conflict of interest.

## Supporting information

As a service to our authors and readers, this journal provides supporting information supplied by the authors. Such materials are peer reviewed and may be re‐organized for online delivery, but are not copy‐edited or typeset. Technical support issues arising from supporting information (other than missing files) should be addressed to the authors.

Supporting Information

## Data Availability

The data that support the findings of this study are available in the supplementary material of this article.
