# Supplementary material for: Experimental Quantification of Halogen⋅⋅⋅Arene van der Waals Contacts
Source: Angew Chem Int Ed Engl. 2023 Aug 9;62(38):e202309682. doi: 10.1002/anie.202309682 (PMC10953438; doi:10.1002/anie.202309682)
Supplement: Supplementary file 1 — Supporting Information [file ANIE-62-0-s001.pdf]

Angewandte Chemie
Eine Zeitschrift der Gesellschaft Deutscher Chemiker

Supporting Information

Experimental Quantification of Halogen···Arene van der Waals Contacts

*A. M. L. West, N. Dominelli-Whiteley, I. V. Smolyar, G. S. Nichol, S. L. Cockroft**

Supporting information

S1. Experimental Data and Methods 2
S2. Computational Data and Methods 6
S3. Synthetic Procedures and Compound Characterisation 24
S4. Crystallographic Data 71
S5. Supporting References 134

S1. Experimental Data and Methods

Determination of conformational folding free energies from ^{1}H NMR spectra

Molecular torsion balance series (±)-**1X** and (±)-**2X** were prepared as 10 mM solutions. Spectra were obtained at 300 K using ^{1}H NMR spectroscopy using a Bruker Ultrashield 600 MHz equipped with a TCI cryoprobe. Measurements in CS_2 and CCl_4 were locked on and calibrated using a sealed capillary tube containing a C_6D_6 internal standard. The conformer peaks were assigned based on the methyl peak being most shielded in the unfolded conformation. Equilibrium constants were determined from the ratio of the integrals of the methyl conformer peaks (in phase- and baseline-corrected) ^{1}H NMR spectra, K = [folded]/[unfolded]. Integration was performed using the line fitting tool in the MestReNova software package. The example below shows fitting for balance (±)-**2Cl** in DCM-d_2.

The conformational free energy difference between the folded and unfolded conformer was then determined using $\Delta G = -RT \ln K$.

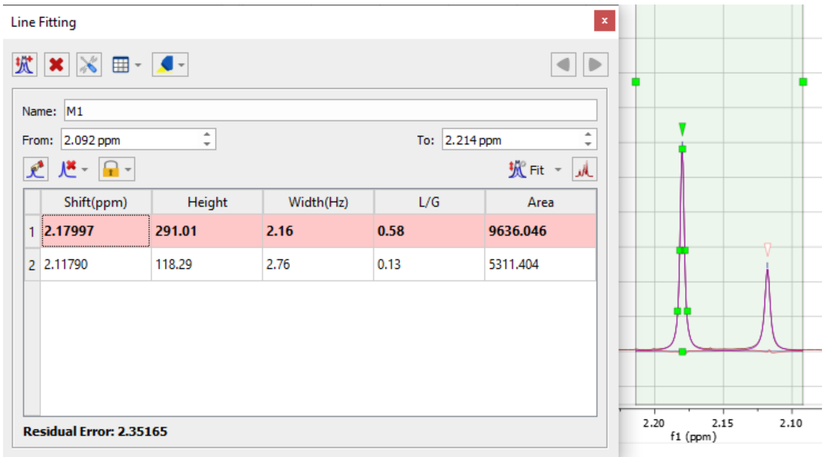

S1.1 Estimation of errors in Δ*G*

Errors in ΔG were estimated at a conservative ±0.2 kJ mol^{-1} for all compounds, in accordance with literature precedence for NMR-based studies of molecular balances.[1] The most extreme conformational free energy difference ΔG reported here is –3.4 kJ mol^{-1}, which corresponds to an approximate conformer ratio ≤ 4: 1. Integration errors associated with modern NMR spectrometers generally do not exceed 1–2%.[2] Hence, even based on a 3% error in integrals, the largest expected errors would be +0.14 kJ mol^{-1} and –0.027 kJ mol^{-1}, which is well within the ±0.2 kJ mol^{-1} estimate.

Errors in $\Delta G_{X\cdots Ph}$ double-mutant cycle values are estimated at ±0.4 kJ mol^{-1} according to the propagation of the conservative ±0.2 kJ mol^{-1} error used for the individual ΔG values (Equation S1).

$$Q_{\Delta\Delta G} = \sqrt{(Q_{\Delta G_{1X}})^2 + (Q_{\Delta G_{1H}})^2 + (Q_{\Delta G_{2X}})^2 + (Q_{\Delta G_{2H}})^2} \quad \text{(Equation S1)}$$

$$Q_{\Delta\Delta G} = \sqrt{(0.2)^2 + (0.2)^2 + (0.2)^2 + (0.2)^2}$$

$$Q_{\Delta\Delta G} = 0.4\ kJ\ mol^{-1}$$

Table S1. Experimental conformational free energy differences for the (±)-**1X** series in kJ mol^{-1} at 300 K.

**Solvent**	$\Delta \boldsymbol{G}_{1I}$	$\Delta \boldsymbol{G}_{1Br}$	$\Delta \boldsymbol{G}_{1Cl}$	$\Delta \boldsymbol{G}_{1}$	$\Delta \boldsymbol{G}_{1H}$	$\Delta \boldsymbol{G}_{1Me}$
CCl_4	-1.8	-1.8	-2.0	-1.9	-1.8	-2.4
C_6D_6	-1.9	-2.0	-2.3	-2.2	-1.9	-2.3
$CDCl_3$	-1.6	-1.6	-1.7	-1.6	-1.4	-1.9
CD_2Cl_2	-1.3	-1.4	-1.5	-1.5	-1.2	-1.6
C_5D_5N	-1.5	-1.6	-1.7	-1.7	-1.3	-2.1
DMSO-d_6	-1.5	-1.8	-2.0	-1.8	-1.6	-2.9
CS_2	-0.6	-0.8	-1.0	-1.0	-0.8	-1.2
2.9% wt I_2 / CS_2	-0.3	-0.5	-0.6	-0.6	-0.3	-0.6
5.8% wt I_2 / CS_2	-0.2	-0.4	-0.5	-0.5	-0.3	-0.6
12.5% wt I_2 / CS_2	-0.4	-0.5	-0.6	-0.5	-0.3	-0.4
THF-d_8	-1.4	-1.3	-1.7	-1.3	-1.7	-2.2
9% v/v D_2O / THF-d_8	-1.5	-1.5	-1.9	-1.8	-1.9	-2.7
15% v/v D_2O / THF-d_8	-1.8	-1.8	-2.0	-2.1	-2.1	-2.6
20% v/v D_2O / THF-d_8	-2.0	-2.1	-2.2	-2.3	-2.3	-3.0
25% v/v D_2O / THF-d_8	-2.2	-2.3	-2.6	-2.3	-2.3	-3.0
35% v/v D_2O / THF-d_8	-2.5	-2.9	-3.1	-2.6	-2.3	-3.2
40% v/v D_2O / THF-d_8	-2.9	-2.8	-2.8	-2.5	-2.3	-3.4
9% v/v CD_3OD / THF-d_8	-1.4	-1.7	-1.9	-1.7	-1.7	-2.4
15% v/v CD_3OD / THF-d_8	-1.4	-1.6	-1.7	-1.5	-1.8	-2.5
20% v/v CD_3OD / THF-d_8	-1.5	-1.7	-1.7	-1.8	-1.8	-2.8
25% v/v CD_3OD / THF-d_8	-1.6	-1.6	-1.8	-1.7	-1.8	-2.7
35% v/v CD_3OD / THF-d_8	-1.6	-1.7	-1.8	-1.4	-1.6	-2.7
40% v/v CD_3OD / THF-d_8	-1.6	-1.8	-1.8	-1.6	-1.7	-2.6

Table S2. Experimental conformational free energy difference for the (±)-**2X** series in kJ mol^{-1} at 300 K.

**Solvent**	ΔG_{2I}	ΔG_{2Br}	ΔG_{2Cl}	ΔG_{2F}	ΔG_{2H}	ΔG_{2Me}
CCl_4	-2.2	-2.3	-2.1	-1.9	-1.2	-1.1
C_6D_6	-2.6	-2.7	-2.5	-2.6	-1.5	-1.3
$CDCl_3$	-2.0	-2.0	-1.8	-1.6	-1.1	-0.9
CD_2Cl_2	-1.6	-1.5	-1.5	-1.5	-0.8	-1.0
C_5D_5N	-2.0	-1.6	-1.8	-1.7	-1.1	-0.5
DMSO-d_6	-1.4	-1.5	-1.4	-1.3	-1.0	-1.1
CS_2	-1.3	-1.3	-1.2	-1.3	-0.1	-0.4
2.9% wt I_2 / CS_2	-1.1	-0.9	-0.9	-0.7	-0.1	-0.1
5.8% wt I_2 / CS_2	-0.7	-1.0	-1.0	-0.7	0.0	-0.2
12.5% wt I_2 / CS_2	-1.0	-1.0	-0.9	-0.7	-0.1	-0.1
THF-d_8	-1.8	-1.8	-1.7	-1.9	-1.4	-1.4
9% v/v D_2O / THF-d_8	-1.7	-2.0	-1.8	-1.6	-1.2	-1.2
15% v/v D_2O / THF-d_8	-1.7	-2.1	-2.0	-1.7	-1.4	-1.2
20% v/v D_2O / THF-d_8	-1.5	-2.1	-2.0	-2.0	-1.3	-1.4
25% v/v D_2O / THF-d_8	-1.8	-2.3	-2.0	-1.9	-1.4	-1.5
35% v/v D_2O / THF-d_8	-2.1	-2.1	-2.3	-2.1	-1.5	-1.7
40% v/v D_2O / THF-d_8	-2.5	-2.4	-2.2	-1.9	-1.5	-1.6

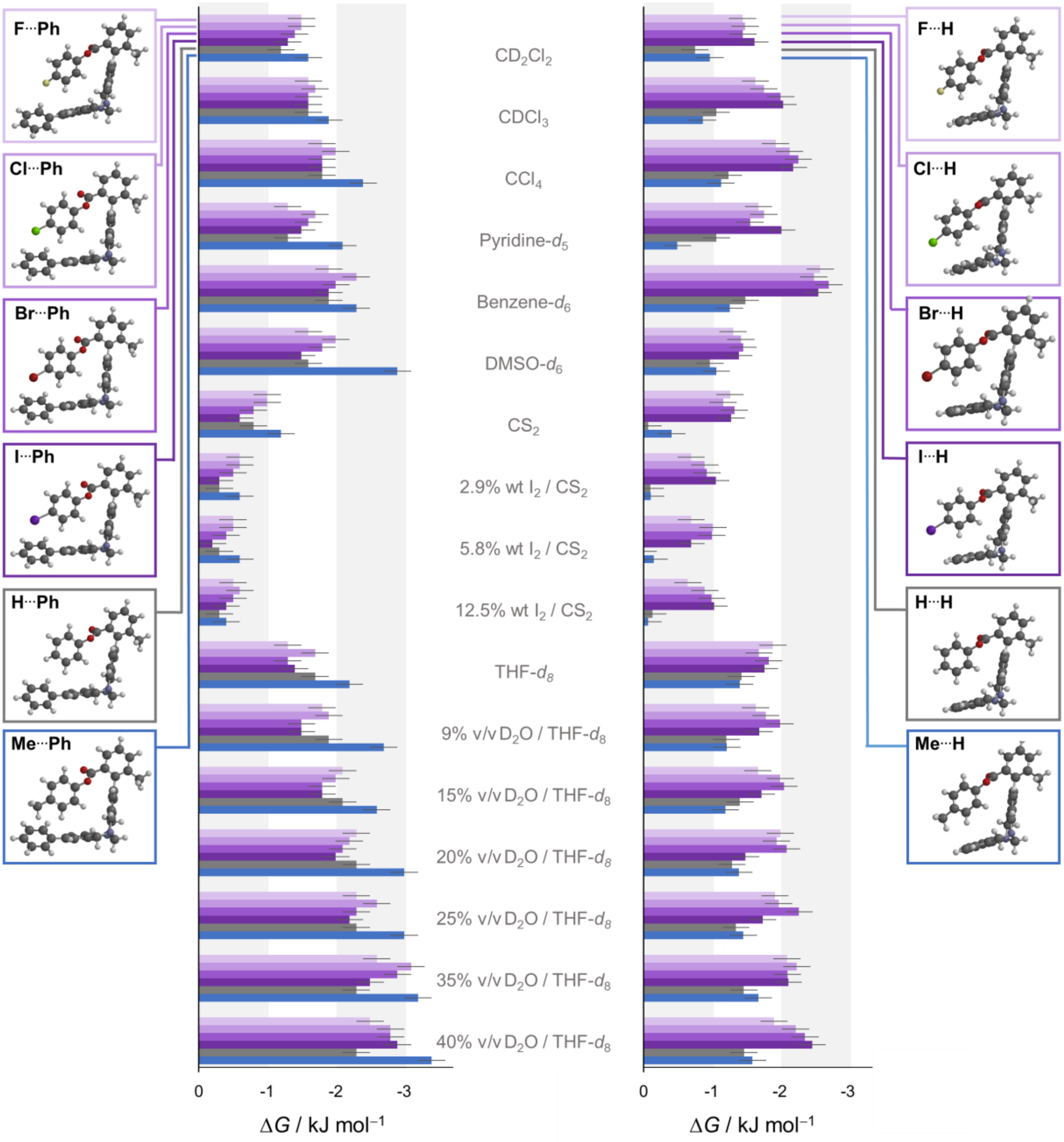

Figure S1. ΔG values obtained for the (±)-**1X** (left) and (±)-**2X** balance series (right) at 300 K. Inset structures were calculated using ωB97X-D/6-311++G**.

S2. Computational Data and Methods

The structures of balances in series (±)-**1X** and (±)-**2X** were initially minimized using Spartan ’14 at B3LYP (dispersion not included) or ωB97X-D (dispersion included) with the 6-311G* basis set. The geometries of the unfolded and folded conformers of each balance were then further geometry minimized using *Gaussian ‘09*,[3] *Spartan ‘14*, or *Spartan ‘20* using the theory/basis set combinations indicated. Conformer distributions and cross-minimisations (with different starting geometries from different calculations/x-ray structures) were also performed on select examples to check that the geometries represented true local minima. Conventional frequency calculations could not be performed at M06-2X/def2-TZVP as the calculations were requesting multiple terabytes of RAM. Hence, vibrational frequencies and thermodynamics were instead calculated using biased Hessian calculations[4] in the *xtb*[7] program for the geometries output by all theory/basis set combinations examined. These calculations further confirmed that local minima had been identified and no negative vibrational modes were observed. The biased Hessian calculated energies are reported in Table S3 for completeness, but we caution that these energies fail to reproduce the experimental energetic trends. In contrast, the total SCF energy differences correctly reproduced the experimental energetic trends, which are reported in main text Figure 3B, Figure S2, and Tables S3 to S6.

Table S3. Gas-phase calculated energies of balance series (±)-**1X** and (±)-**2X** (in kJ mol^{-1}). Energies marked with asterisks are biased Hessian calculations determined as described in supporting reference 4. Only the total SCF energies were able to reproduce the experimentally observed trends (Figure S2).

					*Biased Hessian calculations		
**Method**	**X**	**Ph (series 1X) / H (series 2X)**	**folded / unfolded**	**Total SCF energy**	***Total Energy**	***Total Enthalpy**	***Total Free Energy**
B3LYP/6-311G*	Br	H	folded	-10379476.6	-241434.7	-240208.8	-240434.0
B3LYP/6-311G*	Br	H	unfolded	-10379474.9	-241408.7	-240182.4	-240408.6
B3LYP/6-311G*	Br	Ph	folded	-10986235.9	-280558.5	-279114.8	-279366.0
B3LYP/6-311G*	Br	Ph	unfolded	-10986235.8	-280487.1	-279042.3	-279295.3
B3LYP/6-311G*	Cl	H	folded	-4829378.9	-242661.7	-241434.9	-241657.7
B3LYP/6-311G*	Cl	H	unfolded	-4829377.0	-242644.2	-241417.1	-241640.6
B3LYP/6-311G*	Cl	Ph	folded	-5436139.2	-281774.5	-280329.9	-280579.0
B3LYP/6-311G*	Cl	Ph	unfolded	-5436137.6	-281722.6	-280277.0	-280527.4
B3LYP/6-311G*	F	H	folded	-3883258.9	-243128.3	-241898.9	-242119.1
B3LYP/6-311G*	F	H	unfolded	-3883257.1	-243103.9	-241874.1	-242094.8
B3LYP/6-311G*	F	Ph	folded	-4490019.5	-282226.1	-280778.5	-281025.0
B3LYP/6-311G*****	F	Ph	unfolded	-4490017.7	-282202.8	-280755.0	-281002.3
B3LYP/6-311G*	H	H	folded	-3622636.4	-232025.0	-230776.6	-230992.2
B3LYP/6-311G*	H	H	unfolded	-3622635.4	-232007.0	-230758.4	-230974.4
B3LYP/6-311G*	H	Ph	folded	-4229398.1	-271123.5	-269656.9	-269899.0
B3LYP/6-311G*	H	Ph	unfolded	-4229396.5	-271099.7	-269632.7	-269875.5
B3LYP/6-311G*	I	H	folded	-21788263.7	-240723.2	-239497.5	-239724.5
B3LYP/6-311G*	I	H	unfolded	-21788262.4	-240679.7	-239453.2	-239681.2
B3LYP/6-311G*	I	Ph	folded	-22395022.5	-279839.7	-278396.2	-278649.2
B3LYP/6-311G*	I	Ph	unfolded	-22395023.2	-279770.2	-278325.5	-278580.2
B3LYP/6-311G*	Me	H	folded	-3725884.4	-240353.0	-239028.3	-239254.4
B3LYP/6-311G*	Me	H	unfolded	-3725883.8	-240333.6	-239008.6	-239235.4

B3LYP/6-311G*	Me	Ph	folded	-4332646.6	-279468.3	-277925.6	-278177.8
B3LYP/6-311G*	Me	Ph	unfolded	-4332644.5	-271099.7	-269632.7	-269875.5
M06-2X/6-311++G**	Br	H	folded	-10378282.2	-241409.1	-240182.3	-240405.8
M06-2X/6-311++G**	Br	H	unfolded	-10378267.5	-241384.4	-240157.4	-240384.0
M06-2X/6-311++G**	Br	Ph	folded	-10984818.3	-280486.2	-279040.7	-279292.4
M06-2X/6-311++G**	Br	Ph	unfolded	-10984804.1	-280472.7	-279027.4	-279281.0
M06-2X/6-311++G**	Cl	H	folded	-4828048.9	-242635.8	-241408.1	-241629.2
M06-2X/6-311++G**	Cl	H	unfolded	-4828035.0	-242619.5	-241391.7	-241615.7
M06-2X/6-311++G**	Cl	Ph	folded	-5434585.4	-281721.0	-280274.7	-280523.9
M06-2X/6-311++G**	Cl	Ph	unfolded	-5434570.9	-281708.3	-280262.1	-280512.9
M06-2X/6-311++G**	F	H	folded	-3881922.4	-243106.4	-241876.2	-242094.3
M06-2X/6-311++G**	F	H	unfolded	-3881910.3	-243085.6	-241855.4	-242076.7
M06-2X/6-311++G**	F	Ph	folded	-4488462.0	-282185.7	-280736.6	-280981.5
M06-2X/6-311++G**	F	Ph	unfolded	-4488446.6	-282167.1	-280718.4	-280968.7
M06-2X/6-311++G**	H	H	folded	-3621361.8	-232002.2	-230752.8	-230966.3
M06-2X/6-311++G**	H	H	unfolded	-3621352.3	-231976.7	-230727.1	-230944.9
M06-2X/6-311++G**	H	Ph	folded	-4227900.4	-271082.8	-269614.8	-269857.0
M06-2X/6-311++G**	H	Ph	unfolded	-4227888.2	-271071.6	-269603.8	-269847.3
M06-2X/6-311++G**	I	H	folded	-21788030.6	-240680.9	-239453.9	-239679.3
M06-2X/6-311++G**	I	H	unfolded	-21788015.8	-240667.7	-239440.9	-239669.4
M06-2X/6-311++G**	I	Ph	folded	-22394567.2	-279771.7	-278326.4	-278579.6
M06-2X/6-311++G**	I	Ph	unfolded	-22394552.5	-279749.3	-278304.0	-278562.1
M06-2X/6-311++G**	Me	H	folded	-3724563.8	-240323.5	-238997.8	-239221.6
M06-2X/6-311++G**	Me	H	unfolded	-3724551.3	-240302.0	-238976.1	-239207.1
M06-2X/6-311++G**	Me	Ph	folded	-4331104.9	-279409.0	-277864.6	-278116.8
M06-2X/6-311++G**	Me	Ph	unfolded	-4331087.7	-279389.9	-277845.6	-278103.3
M06-2X/def2-TZVP	Br	H	folded	-10378743.8	-241409.2	-240182.3	-240405.5
M06-2X/def2-TZVP	Br	H	unfolded	-10378731.2	-241384.4	-240157.4	-240383.9
M06-2X/def2-TZVP	Br	Ph	folded	-10985346.9	-280486.2	-279040.7	-279292.4
M06-2X/def2-TZVP	Br	Ph	unfolded	-10985336.5	-280472.5	-279027.1	-279280.9
M06-2X/def2-TZVP	Cl	H	folded	-4828450.5	-242635.8	-241408.0	-241629.2
M06-2X/def2-TZVP	Cl	H	unfolded	-4828439.0	-242619.6	-241391.8	-241615.5
M06-2X/def2-TZVP	Cl	Ph	folded	-5435054.2	-281721.0	-280274.7	-280524.0
M06-2X/def2-TZVP	Cl	Ph	unfolded	-5435044.2	-281708.3	-280262.1	-280512.8
M06-2X/def2-TZVP	F	H	folded	-3882348.0	-243093.7	-241863.3	-242082.0
M06-2X/def2-TZVP	F	H	unfolded	-3882337.1	-243085.7	-241855.4	-242076.7
M06-2X/def2-TZVP	F	Ph	folded	-4488955.2	-282185.7	-280736.7	-280981.4
M06-2X/def2-TZVP	F	Ph	unfolded	-4488943.0	-282174.6	-280725.8	-280973.8
M06-2X/def2-TZVP	H	H	folded	-3621756.2	-232002.1	-230752.8	-230966.3
M06-2X/def2-TZVP	H	H	unfolded	-3621748.6	-231976.7	-230727.1	-230944.9
M06-2X/def2-TZVP	H	Ph	folded	-4228362.9	-271082.9	-269614.9	-269856.9
M06-2X/def2-TZVP	H	Ph	unfolded	-4228353.1	-271071.7	-269603.8	-269847.4
M06-2X/def2-TZVP	I	H	folded	-4401617.7	-240680.8	-239453.9	-239679.2
M06-2X/def2-TZVP	I	H	unfolded	-4401605.4	-240667.8	-239440.9	-239669.1
M06-2X/def2-TZVP	I	Ph	folded	-5008221.3	-279771.6	-278326.4	-278579.6
M06-2X/def2-TZVP	I	Ph	unfolded	-5008210.8	-279749.3	-278304.0	-278561.6
M06-2X/def2-TZVP	Me	H	folded	-3724967.1	-240316.8	-238990.9	-239215.2
M06-2X/def2-TZVP	Me	H	unfolded	-3724957.3	-240301.9	-238975.9	-239205.3
M06-2X/def2-TZVP	Me	Ph	folded	-4331575.7	-279409.0	-277864.6	-278116.9
M06-2X/def2-TZVP	Me	Ph	unfolded	-4331562.5	-279389.9	-277845.6	-278105.0
ωB97X/6-311G*	Br	H	folded	-10378559.8	-241407.6	-240180.8	-240405.3
ωB97X/6-311G*	Br	H	unfolded	-10378549.9	-241384.2	-240157.4	-240384.2

ωB97X/6-311G*	Br	Ph	folded	-10985155.7	-280486.4	-279041.0	-279292.9
ωB97X/6-311G*	Br	Ph	unfolded	-10985144.2	-280473.1	-279027.9	-279281.8
ωB97X/6-311G*	Cl	H	folded	-4828422.9	-242641.2	-241413.8	-241635.3
ωB97X/6-311G*	Cl	H	unfolded	-4828413.4	-242619.6	-241391.9	-241615.9
ωB97X/6-311G*	Cl	Ph	folded	-5435019.2	-281721.7	-280275.8	-280525.4
ωB97X/6-311G*	Cl	Ph	unfolded	-5435007.6	-281708.5	-280262.5	-280513.6
ωB97X/6-311G*	F	H	folded	-3882287.5	-243085.3	-241855.1	-242076.8
ωB97X/6-311G*	F	H	unfolded	-3882279.7	-243085.6	-241855.4	-242076.6
ωB97X/6-311G*	F	Ph	folded	-4488885.7	-282185.3	-280736.4	-280982.8
ωB97X/6-311G*	F	Ph	unfolded	-4488873.5	-282174.7	-280726.1	-280974.4
ωB97X/6-311G*	H	H	folded	-3621714.4	-231982.0	-230732.7	-230950.1
ωB97X/6-311G*	H	H	unfolded	-3621708.1	-231983.0	-230733.6	-230950.1
ωB97X/6-311G*	H	Ph	folded	-4228312.4	-271074.7	-269606.7	-269852.0
ωB97X/6-311G*	H	Ph	unfolded	-4228301.8	-271072.0	-269604.3	-269848.0
ωB97X/6-311G*	I	H	folded	-3650011.4	-38472.7	-38387.6	-38479.4
ωB97X/6-311G*	I	H	unfolded	-3650000.9	-240667.8	-239441.1	-239669.7
ωB97X/6-311G*	I	Ph	folded	-4256607.2	-279771.9	-278326.6	-278579.9
ωB97X/6-311G*	I	Ph	unfolded	-4256594.7	-279756.8	-278311.7	-278567.4
ωB97X/6-311G*	Me	H	folded	-3724936.4	-240315.9	-238990.1	-239216.1
ωB97X/6-311G*	Me	H	unfolded	-3724929.5	-240308.6	-238983.0	-239210.2
ωB97X/6-311G*	Me	Ph	folded	-4331537.2	-279409.4	-277865.1	-278117.7
ωB97X/6-311G*	Me	Ph	unfolded	-4331523.3	-279397.6	-277853.6	-278108.0
ωB97X-D/6-311++G**	Br	H	folded	-10378513.7	-241408.2	-240181.5	-240405.3
ωB97X-D/6-311++G**	Br	H	unfolded	-10378498.9	-241397.1	-240170.4	-240396.7
ωB97X-D/6-311++G**	Br	Ph	folded	-10985087.7	-280530.3	-279085.8	-279337.0
ωB97X-D/6-311++G**	Br	Ph	unfolded	-10985070.2	-280487.9	-279042.9	-279296.0
ωB97X-D/6-311++G**	Cl	H	folded	-4828296.8	-242641.5	-241414.0	-241635.3
ωB97X-D/6-311++G**	Cl	H	unfolded	-4828282.8	-242644.8	-241417.6	-241641.2
ωB97X-D/6-311++G**	Cl	Ph	folded	-5434870.1	-281735.5	-280289.6	-280538.6
ωB97X-D/6-311++G**	Cl	Ph	unfolded	-5434853.3	-281745.0	-280299.7	-280550.1
ωB97X-D/6-311++G**	F	H	folded	-3882153.3	-243099.5	-241869.3	-242088.1
ωB97X-D/6-311++G**	F	H	unfolded	-3882141.3	-243098.4	-241868.4	-242089.2
ωB97X-D/6-311++G**	F	Ph	folded	-4488727.9	-282185.6	-280736.7	-280982.4
ωB97X-D/6-311++G**	F	Ph	unfolded	-4488711.7	-282218.7	-280771.0	-281018.9
ωB97X-D/6-311++G**	H	H	folded	-3621592.8	-231989.0	-230739.6	-230955.0
ωB97X-D/6-311++G**	H	H	unfolded	-3621583.1	-231995.8	-230746.6	-230962.9
ωB97X-D/6-311++G**	H	Ph	folded	-4228169.5	-271082.5	-269614.7	-269857.0
ωB97X-D/6-311++G**	H	Ph	unfolded	-4228153.9	-271086.4	-269618.9	-269862.0
ωB97X-D/6-311++G**	I	H	folded	-21787638.1	-240692.6	-239465.9	-239691.6
ωB97X-D/6-311++G**	I	H	unfolded	-21787621.9	-240692.9	-239466.6	-239694.5
ωB97X-D/6-311++G**	I	Ph	folded	-22394212.1	-279808.3	-278363.9	-278616.7
ωB97X-D/6-311++G**	I	Ph	unfolded	-22394196.1	-279756.8	-278311.8	-278568.2
ωB97X-D/6-311++G**	Me	H	folded	-3724818.8	-240330.2	-239004.6	-239229.4
ωB97X-D/6-311++G**	Me	H	unfolded	-3724806.6	-240348.0	-239025.5	-239247.4
ωB97X-D/6-311++G**	Me	Ph	folded	-4331397.9	-279416.9	-277872.7	-278124.8
ωB97X-D/6-311++G**	Me	Ph	unfolded	-4331376.6	-279412.5	-277868.7	-278122.4
ωB97X-D/6-311G*	Br	H	folded	-10378387.4	-241407.7	-240181.0	-240405.2
ωB97X-D/6-311G*	Br	H	unfolded	-10378371.9	-241384.4	-240157.5	-240384.4
ωB97X-D/6-311G*	Br	Ph	folded	-10984938.8	-280486.1	-279040.7	-279292.4
ωB97X-D/6-311G*	Br	Ph	unfolded	-10984921.5	-280473.1	-279027.9	-279281.6
ωB97X-D/6-311G*	Cl	H	folded	-4828166.9	-242641.5	-241414.0	-241635.4
ωB97X-D/6-311G*	Cl	H	unfolded	-4828152.1	-242619.7	-241392.0	-241616.0

ωB97X-D/6-311G*	Cl	Ph	folded	-5434718.4	-281721.1	-280274.9	-280524.4
ωB97X-D/6-311G*	Cl	Ph	unfolded	-5434701.4	-281708.6	-280262.5	-280513.5
ωB97X-D/6-311G*	F	H	folded	-3882018.2	-243106.3	-241876.1	-242094.4
ωB97X-D/6-311G*	F	H	unfolded	-3882004.8	-243085.6	-241855.4	-242076.6
ωB97X-D/6-311G*	F	Ph	folded	-4488572.3	-282185.0	-280736.3	-280982.9
ωB97X-D/6-311G*	F	Ph	unfolded	-4488553.8	-282167.1	-280718.4	-280968.3
ωB97X-D/6-311G*	H	H	folded	-3621461.7	-231988.8	-230739.5	-230955.0
ωB97X-D/6-311G*	H	H	unfolded	-3621451.5	-231976.7	-230727.2	-230944.8
ωB97X-D/6-311G*	H	Ph	folded	-4228017.1	-271081.9	-269614.0	-269856.6
ωB97X-D/6-311G*	H	Ph	unfolded	-4228000.6	-271071.7	-269604.0	-269847.7
ωB97X-D/6-311G*	I	H	folded	-21787513.2	-240692.5	-239465.9	-239691.7
ωB97X-D/6-311G*	I	H	unfolded	-21787496.2	-240667.9	-239441.2	-239669.7
ωB97X-D/6-311G*	I	Ph	folded	-22394065.1	-279771.9	-278326.7	-278580.0
ωB97X-D/6-311G*	I	Ph	unfolded	-22394045.8	-279749.3	-278304.0	-278561.7
ωB97X-D/6-311G*	Me	H	folded	-3724682.2	-240323.3	-238997.6	-239222.4
ωB97X-D/6-311G*	Me	H	unfolded	-3724669.0	-240308.6	-238983.0	-239210.1
ωB97X-D/6-311G*	Me	Ph	folded	-4331239.8	-279409.4	-277865.1	-278117.5
ωB97X-D/6-311G*	Me	Ph	unfolded	-4331217.9	-279389.8	-277848.1	-278099.6

Table S4. Gas-phase computational energy differences for the (±)-**1X** series (in kJ mol^{-1} using Total SCF energy differences).

**Method**	ΔE_{1I}	ΔE_{1Br}	ΔE_{1Cl}	ΔE_{1F}	ΔE_{1H}	ΔE_{1Me}
no dispersion correction						
B3LYP/6-311G*	+0.7	–0.1	–1.6	–1.8	–1.6	–2.1
ωB97X/6-311G*	–12.9	–11.5	–11.6	–12.2	–10.6	–13.9
dispersion corrected						
M06-2X/6-311++G**	–14.7	–14.2	–14.5	–15.4	–12.1	–17.1
M06-2X/def2-TZVP	–10.5	–10.4	–10.0	–12.2	–9.7	–13.2
ωB97X-D/6-311G*	–19.3	–17.3	–16.9	–18.5	–16.5	–21.9
ωB97X-D/6-311++G**	–16.0	–17.5	–16.8	–16.2	–15.6	–21.4

Table S5. Gas-phase computational energy differences for the (±)-**2X** series (in kJ mol^{-1} using Total SCF energy differences).

**Method**	ΔE_{2I}	ΔE_{2Br}	ΔE_{2Cl}	ΔE_{2F}	ΔE_{2H}	ΔE_{2Me}
no dispersion correction						
B3LYP/6-311G*	–1.3	–1.7	–1.9	–1.8	–0.9	–0.6
ωB97X/6-311G*	–10.6	–9.9	–9.4	–7.9	–6.3	–6.9
dispersion corrected						
M06-2X/6-311++G**	–14.8	–14.7	–13.8	–12.1	–9.5	–12.4
M06-2X/def2-TZVP	–12.3	–12.6	–11.5	–10.9	–7.7	–9.8
ωB97X-D/6-311G*	–17.0	–15.5	–14.8	–13.4	–10.2	–13.1
ωB97X-D/6-311++G**	–16.2	–14.8	–14.0	–12.0	–9.7	–12.2

Table S6. Computational double-mutant cycle energies for dissected halogen-arene interactions determined using the values in Tables S4 and S5 above (in kJ mol^{-1} using Total SCF energy differences).

Method	$\Delta E_{I\cdots Ph}$	$\Delta E_{Br\cdots Ph}$	$\Delta E_{Cl\cdots Ph}$	$\Delta E_{F\cdots Ph}$	$\Delta E_{Me\cdots Ph}$
no dispersion correction					
B3LYP/6-311G*	+2.7	+2.3	+1.0	+0.7	–0.8
ωB97X/6-311G*	+2.0	+2.6	+2.1	–0.1	–2.8
dispersion corrected					
M06-2X/6-311++G**	+2.7	+3.1	+2.0	–0.7	–2.1
M06-2X/def2-TZVP	+3.9	+4.3	+3.6	+0.7	–1.3
ωB97X-D/6-311G*	+4.0	+4.5	+4.2	+1.2	–2.5
ωB97X-D/6-311++G**	+6.0	+3.1	+3.1	+1.7	–3.3

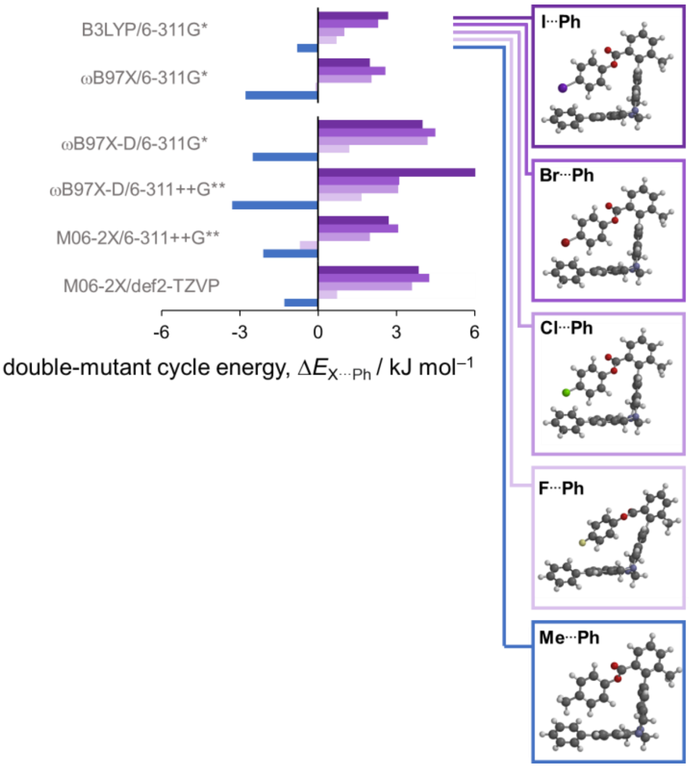

Figure S2. Computed halogen···arene (purple) and CH_3···arene (blue) interaction energies dissected using the thermodynamic double-mutant cycle shown in Figure 2 in the main text and using the Total SCF energy differences reported in Tables S3-S6. Negative energies correspond to favorable X···arene interactions. The example inset structures were calculated using M06-2X/def2-TZVP.

S2.1 Comparison of X-ray Crystal Structures and DFT-minimized geometries

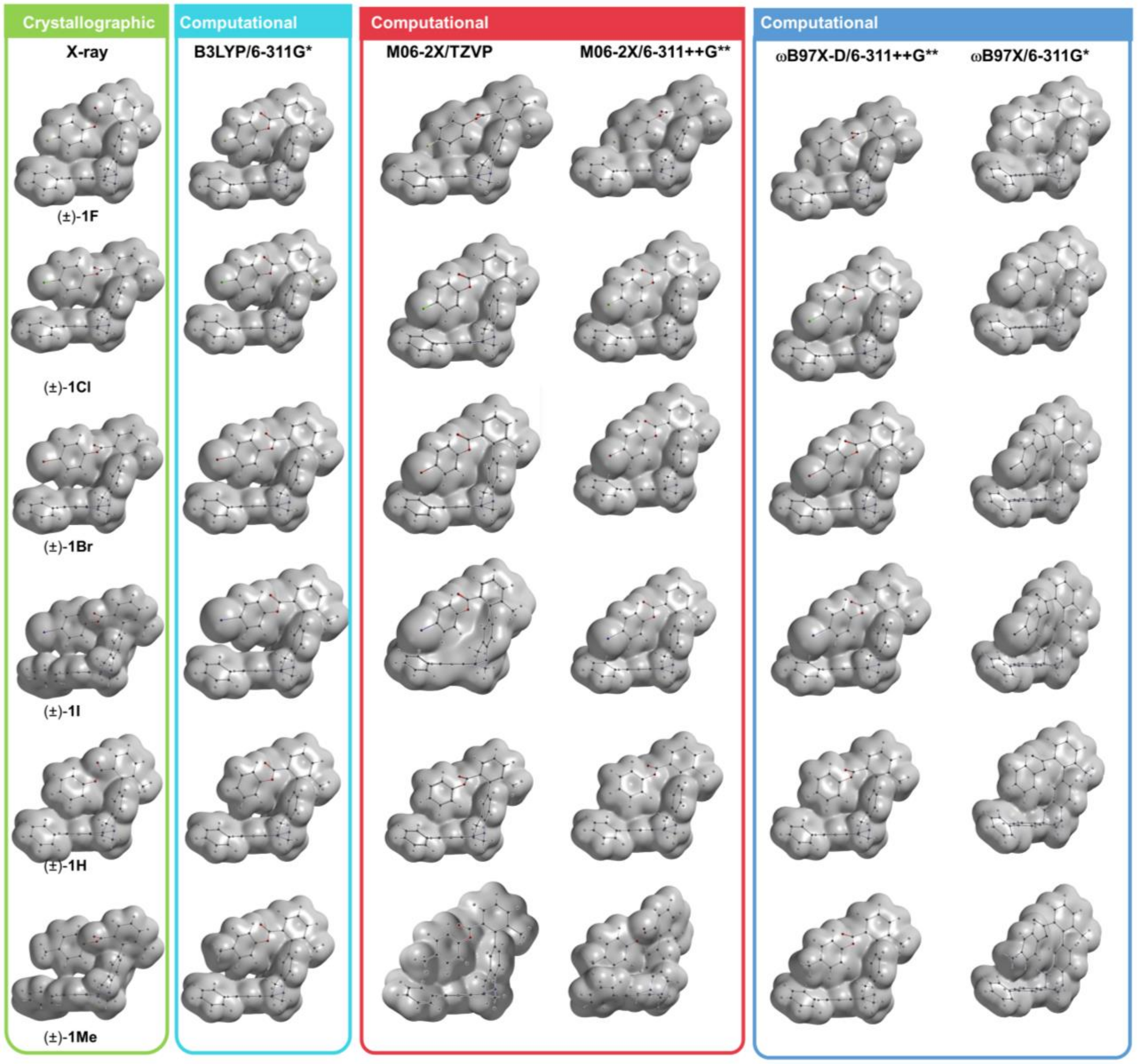

Figure S3. Geometries obtained from x-ray crystallography of the (±)-**1X** series and the corresponding DFT-minimized geometries. Electron surfaces at the 0.002 e⁻ / au^3 isosurface. Surfaces for the X-ray crystal structures were calculated without minimisation using B3LYP/6-311G*. CCDC 2244208-2244213 contain the supplementary crystallographic data for this paper. These data are provided free of charge by The Cambridge Crystallographic Data Centre.

S2.2 SAPT calculations

The SAPT calculations reported Table S7 and plotted in Figure 4 in the main text were performed on the halogenophenyl···phenyl interactions using the Psi4[5] software package with the jun-cc-pVTZ basis set using both SAPT0 and SAPT2. Although SAPT2 is the more recent method, benchmarking indicated that SAPT0/jun-cc-pVTZ was the better performing combination (data presented in Table S7 and main text Figure 4).[6]

The SAPT calculations used the minimized structures of the (±)-**1X** series balances determined using all theory/basis set combinations shown in the previous section (Figure S3 and uploaded .xyz files). Truncated versions of these minimized geometries were used in which the Ph-X group and the Ph group contacting X were retained, while the rest of the molecular balance was deleted and replaced with a proton with a default C-H bond distance of 1.096 Å. The resulting decomposed energies for these geometries determined with the jun-cc-pVTZ basis set using both SAPT0 and SAPT2 and the corresponding bar graphs are given below.

The calculations show that the small changes in interaction geometries between the different methods and use of SAPT0 vs. SAPT2 both play a minimal role in determining the energy compositions. The only major difference is that the energies for the B3LYP/6-311G* geometries are all much smaller in magnitude than for the geometries minimized using methods that better account for dispersion. This makes sense because the B3LYP geometry minimizations do not account for dispersion and accordingly, do not form close X···Ph contacts, unlike the other methods used. In contrast with both the experimental and computational double-mutant cycle calculations presented in the main text, the SAPT calculations predict the halogen···phenyl interactions to become less unfavorable as the size of the halogen increases.

Table S7. SAPT0/jun-cc-pVTZ energy decomposition (in kJ mol^{-1}) for the isolated Ph-X···Ph interaction in the (±)-**1X** series of balances geometry-minimized using M06-2X/def2-TZVP. These interaction energies are plotted in Figure 4 of the main text.

X	Electrostatic	Exchange	Induction	Dispersion	SAPT0 Total
H	-5.8	11.6	-1.4	-12.2	-7.8
F	-3.9	6.4	-1.1	-6.7	-5.2
Cl	-7.0	22.9	-2.9	-22.5	-9.5
Br	-7.3	23.6	-3.0	-24.2	-10.9
I	-8.9	27.0	-3.2	-27.7	-12.8
Me	-11.1	25.4	-2.9	-25.2	-13.7

Table S8. SAPT0/jun-cc-pVTZ energy decomposition (in kJ mol^{-1}) for the isolated Ph-X···Ph interaction in the (±)-**1X** series of balances geometry-minimized using B3LYP/6-311G*.

X	Electrostatic	Exchange	Induction	Dispersion	SAPT0 Total
H	-1.9	2.3	-0.4	-5.1	-5.2
F	-0.8	2.6	-0.6	-5.6	-4.4
Cl	-0.3	1.4	-0.4	-5.3	-4.6
Br	-0.7	3.7	-0.6	-8.4	-6.1
I	-0.3	1.9	-0.4	-7.0	-5.8
Me	-2.4	2.5	-0.5	-6.7	-7.0

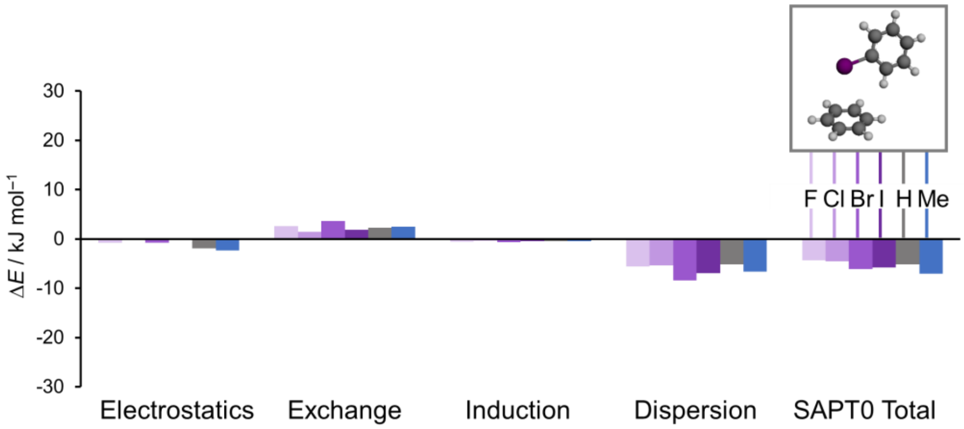

Table S9. SAPT0/jun-cc-pVTZ energy decomposition (in kJ mol^{-1}) for the isolated Ph-X···Ph interaction in the (±)-**1X** series of balances geometry-minimized using M06-2X/6-311++G**.

X	Electrostatic	Exchange	Induction	Dispersion	SAPT0 Total
H	-6.1	12.3	-1.5	-12.7	-7.9
F	-4.6	7.8	-1.3	-7.1	-5.3
Cl	-7.1	23.5	-2.9	-22.9	-9.4
Br	-7.4	24.1	-3.0	-24.5	-10.9
I	-8.7	26.6	-3.2	-27.5	-12.8
Me	-11.2	25.7	-2.9	-25.3	-13.7

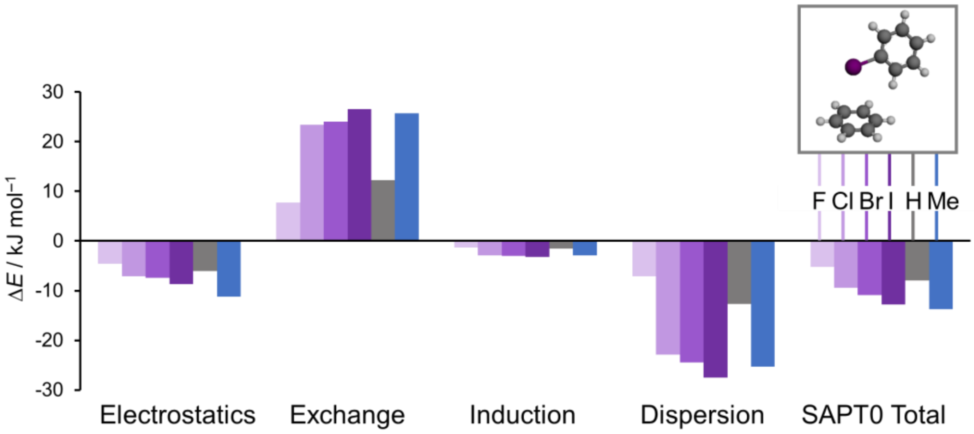

Table S10. SAPT0/jun-cc-pVTZ energy decomposition (in kJ mol^{-1}) for the isolated Ph-X···Ph interaction in the (±)-**1X** series of balances geometry-minimized using ωB97X/6-311G*.

X	Electrostatic	Exchange	Induction	Dispersion	SAPT0 Total
H	-4.8	8.9	-1.1	-10.8	-7.8
F	-3.9	8.5	-1.2	-8.3	-5.0
Cl	-3.4	12.4	-1.4	-13.8	-6.1
Br	-4.4	15.6	-2.2	-19.0	-10.0
I	-4.8	16.3	-2.2	-21.1	-11.8
Me	-7.4	14.9	-2.0	-17.8	-12.3

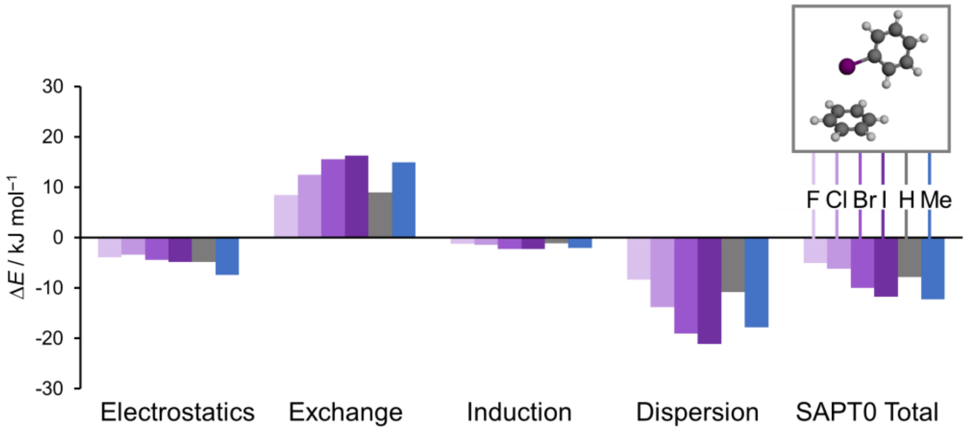

Table S11. SAPT0/jun-cc-pVTZ energy decomposition (in kJ mol^{-1}) for the isolated Ph-X···Ph interaction in the (±)-**1X** series of balances geometry-minimized using ωB97X-D/6-311++G**.

X	Electrostatic	Exchange	Induction	Dispersion	SAPT0 Total
H	-4.3	8.8	-1.1	-10.7	-7.2
F	-3.6	7.0	-1.1	-7.1	-4.9
Cl	-3.8	15.2	-2.2	-17.9	-8.6
Br	-4.4	17.2	-2.3	-20.8	-10.3
I	-4.8	17.4	-2.2	-22.2	-11.8
Me	-9.2	20.5	-2.4	-22.2	-13.3

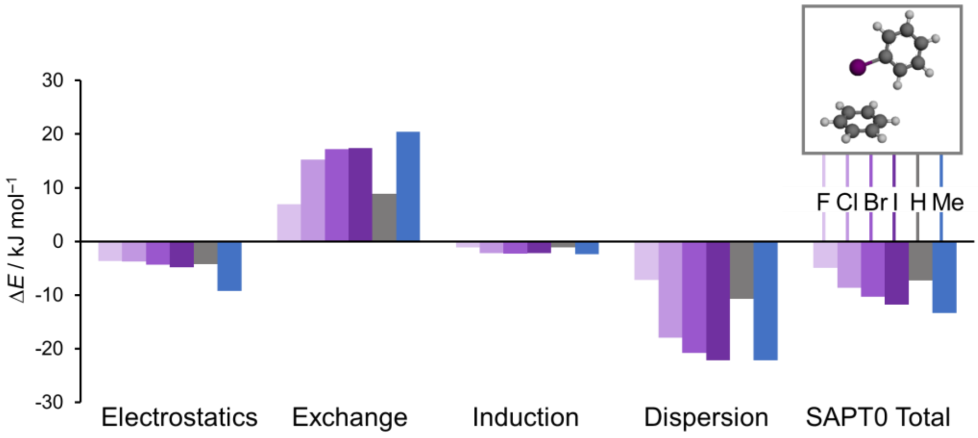

Table S12. SAPT0/jun-cc-pVTZ energy decomposition (in kJ mol^{-1}) for the isolated Ph-X···Ph interaction in the (±)-**1X** series of balances geometry-minimized using ωB97X-D/6-311G*.

X	Electrostatic	Exchange	Induction	Dispersion	SAPT0 Total
H	-6.4	12.5	-1.5	-13.0	-8.4
F	-2.9	8.3	-1.2	-8.5	-4.3
Cl	-4.1	16.0	-2.2	-18.2	-8.5
Br	-4.7	17.5	-2.4	-20.8	-10.4
I	-5.1	17.8	-2.3	-22.4	-12.0
Me	-9.4	20.9	-2.4	-22.1	-13.0

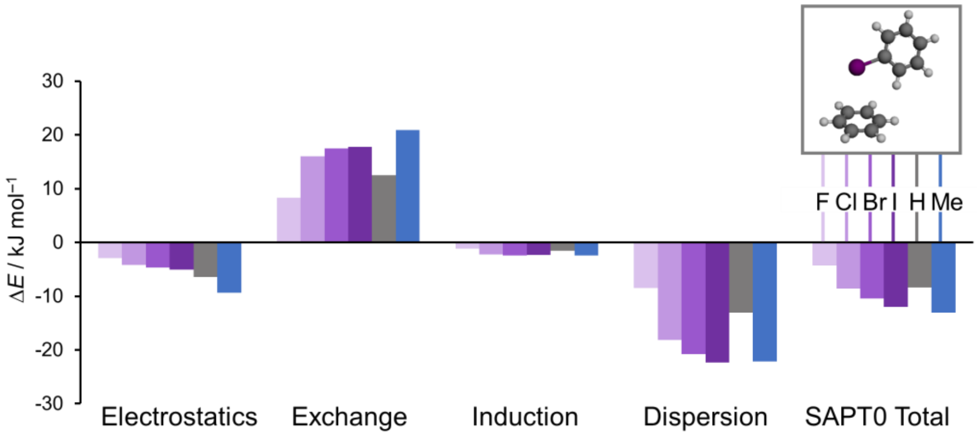

Table S15. SAPT2/jun-cc-pVTZ energy decomposition (in kJ mol^{-1}) for the isolated Ph-X···Ph interaction in the (±)-**1X** series of balances geometry-minimized using M06-2X/def2-TZVP.

X	Electrostatic	Exchange	Induction	Dispersion	SAPT2 Total
H	-5.7	11.9	-1.4	-12.2	-7.4
F	-3.9	7.4	-1.1	-6.7	-4.4
Cl	-7.5	23.3	-2.9	-22.5	-9.7
Br	-7.9	24.1	-2.9	-24.2	-11.0
I	-9.4	27.4	-3.3	-27.7	-13.0
Me	-13.7	26.2	-2.9	-25.2	-13.0

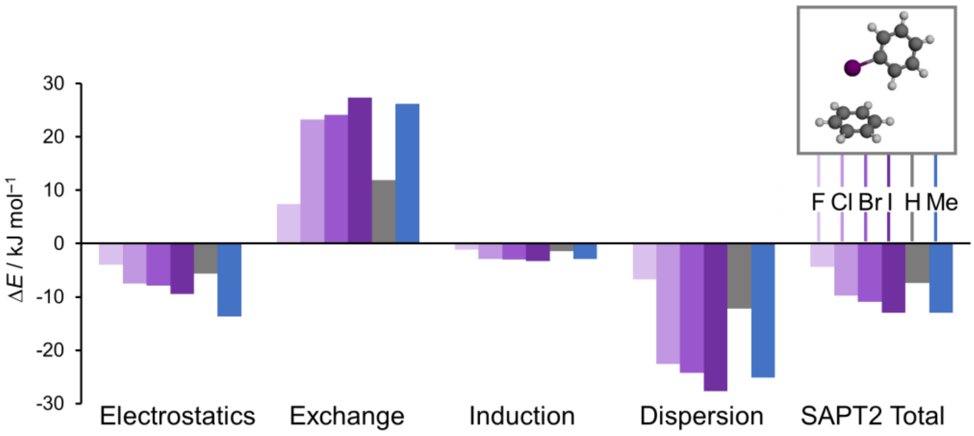

Table S13. SAPT2/jun-cc-pVTZ energy decomposition (in kJ mol^{-1}) for the isolated Ph-X···Ph interaction in the (±)-**1X** series of balances geometry-minimized using B3LYP/6-311G*.

X	Electrostatic	Exchange	Induction	Dispersion	SAPT2 Total
H	-1.8	2.3	-0.4	-5.1	-5.0
F	-1.0	2.6	-0.6	-5.6	-4.5
Cl	-0.4	1.5	-0.4	-5.3	-4.6
Br	-1.0	3.8	-0.6	-8.4	-6.2
I	-0.4	1.9	-0.4	-7.0	-5.9
Me	-2.2	2.6	-0.5	-6.7	-6.8

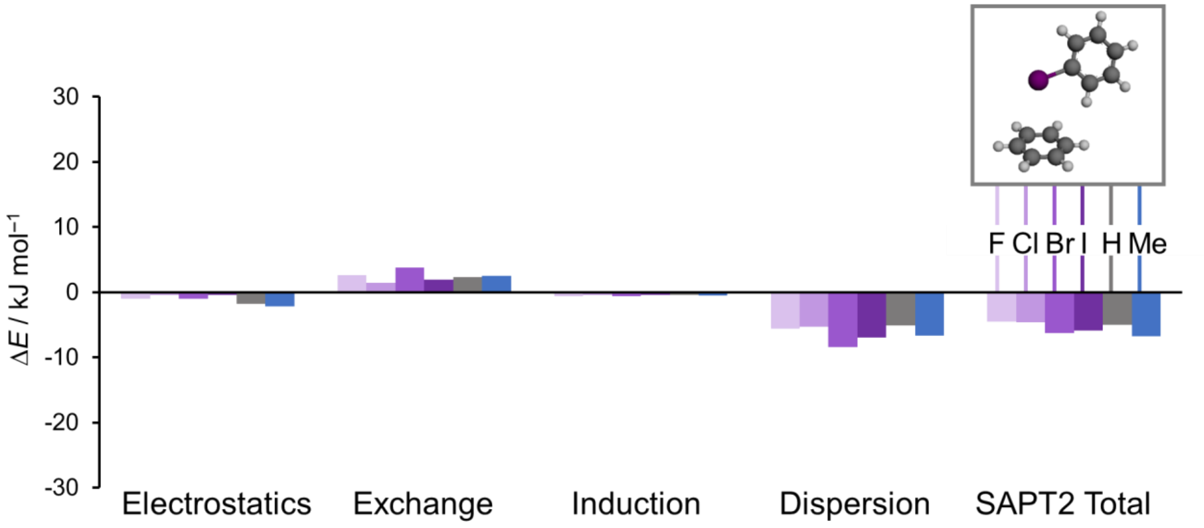

Table S14. SAPT2/jun-cc-pVTZ energy decomposition (in kJ mol^{-1}) for the isolated Ph-X···Ph interaction in the (±)-**1X** series of balances geometry-minimized using M06-2X/6-311++G**.

X	Electrostatic	Exchange	Induction	Dispersion	SAPT2 Total
H	-5.9	12.6	-1.5	-12.7	-7.5
F	-4.7	8.9	-1.4	-7.1	-4.3
Cl	-7.7	23.9	-3.0	-22.9	-9.7
Br	-8.1	24.6	-3.0	-24.5	-10.9
I	-9.2	26.9	-3.3	-27.5	-13.0
Me	-11.2	26.5	-3.0	-25.3	-13.0

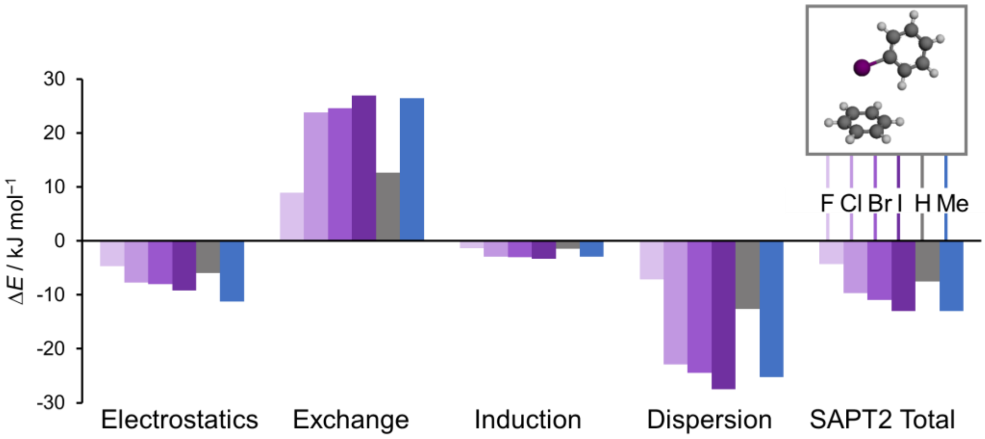

Table S16. SAPT2/jun-cc-pVTZ energy decomposition (in kJ mol^{-1}) for the isolated Ph-X···Ph interaction in the (±)-**1X** series of balances geometry-minimized using ωB97X/6-311G*.

X	Electrostatic	Exchange	Induction	Dispersion	SAPT2 Total
H	-4.6	9.2	-1.1	-10.8	-7.4
F	-4.4	9.7	-1.3	-8.3	-4.3
Cl	-3.9	12.8	-1.4	-13.8	-6.3
Br	-5.0	16.0	-2.1	-19.0	-10.1
I	-5.2	16.6	-2.2	-21.1	-12.0
Me	-7.3	15.5	-2.0	-17.8	-11.7

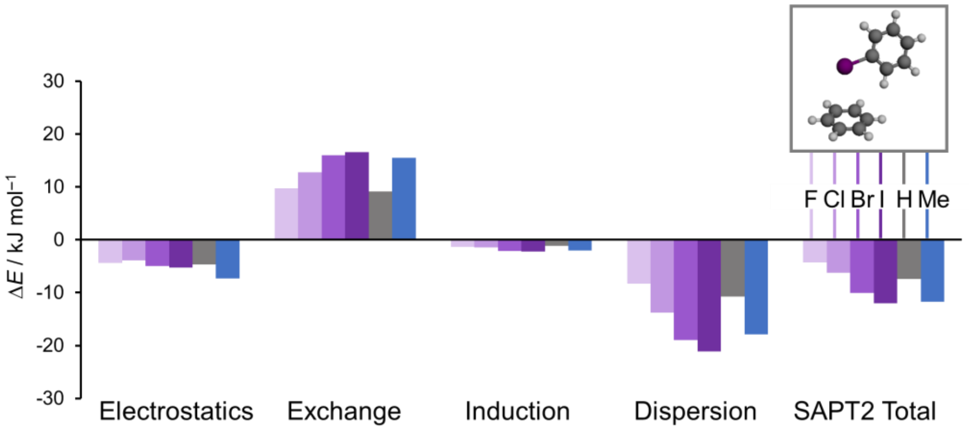

Table S17. SAPT2/jun-cc-pVTZ energy decomposition (in kJ mol^{-1}) for the isolated Ph-X···Ph interaction in the (±)-**1X** series of balances geometry-minimized using ωB97X-D/6-311++G**.

X	Electrostatic	Exchange	Induction	Dispersion	SAPT2 Total
H	-4.3	9.2	-1.1	-10.7	-6.9
F	-3.8	8.0	-1.2	-7.1	-4.2
Cl	-4.4	15.7	-2.2	-17.9	-8.9
Br	-5.2	17.8	-2.2	-20.8	-10.5
I	-5.5	17.8	-2.2	-22.2	-12.1
Me	-9.3	21.2	-2.4	-22.2	-12.7

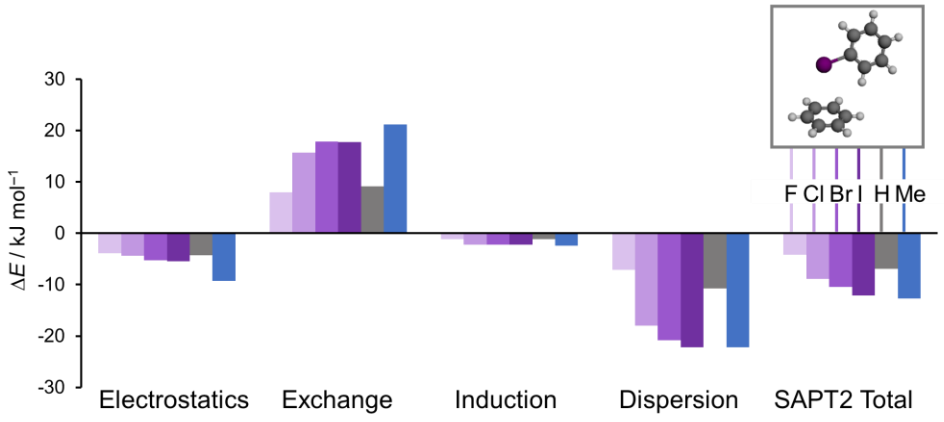

Table S18. SAPT2/jun-cc-pVTZ energy decomposition (in kJ mol^{-1}) for the isolated Ph-X···Ph interaction in the (±)-**1X** series of balances geometry-minimized using ωB97X-D/6-311G*.

X	Electrostatic	Exchange	Induction	Dispersion	SAPT2 Total
H	-6.1	12.7	-1.5	-13.0	-8.0
F	-3.5	9.4	-1.2	-8.5	-3.8
Cl	-4.8	16.4	-2.2	-18.2	-8.8
Br	-5.4	18.0	-2.4	-20.8	-10.5
I	-5.7	18.1	-2.3	-22.4	-12.3
Me	-9.4	21.6	-2.4	-22.1	-12.4

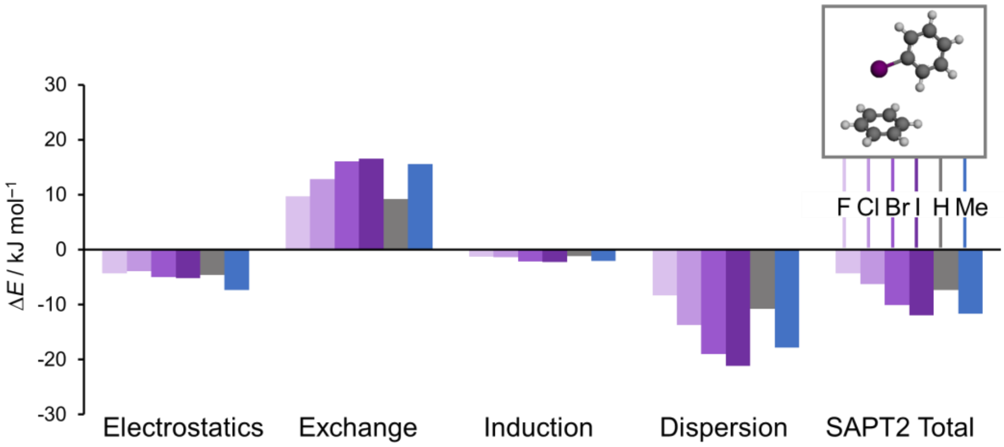

S3. Synthetic Procedures and Compound Characterisation

Unless stated otherwise all synthetic procedures reported were conducted used reagents purchased from commercial sources and used without additional purification. Solvents listed as dry were either purchased as anhydrous or were purified using a Glass Contour brand solvent purification system. In addition, reactions requiring dry conditions used oven dried glassware (130-140°C overnight). Nitrogen gas for inert atmospheres was provided via in house supply. Analytical TLC was conducted on aluminium backed silica gel 60 F254 plates and visualized under UV (254 nm) light. Flash chromatography was carried out using Geduran 60 (40 – 63 µm) silica gel eluting with the specified solvents/solvent mixtures. In all cases "petroleum either" refers to 40-60°C boiling point range grade.

NMR spectra were recorded using either a 500 MHz Bruker Ultrashield equipped with a prodigy cryoprobe, or a 600 MHz Bruker Ultrashield spectrometer equipped with a TCI cryoprobe at 300 K. All NMR analysis was conducted using standard Pyrex tubes, ambered tubes were used for analysis of photoreactive iodine species. Chemical shifts are reported in parts per million (ppm) coupling constants are reported in Hertz. High resolution mass spectroscopy was conducted using a Bruker micrOTOF II. Dr Logan Mackay of the University of Edinburgh is thanked for this service.

S3.1 Synthesis and characterisation of balance series (±)-1X

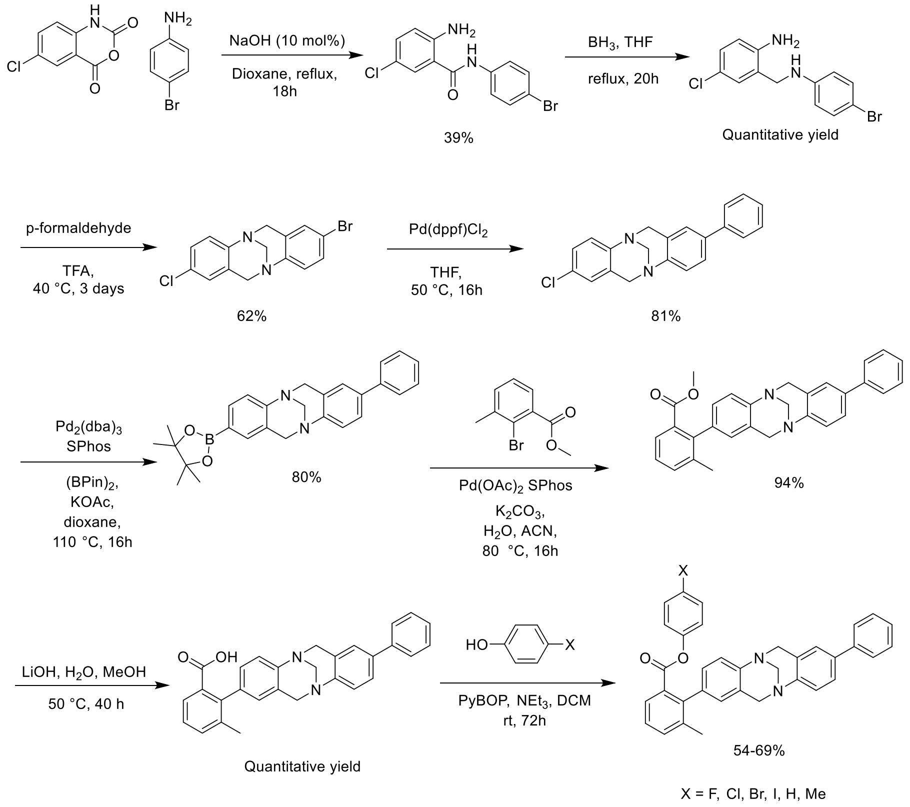

Step I. 2-Amino-*N*-(4-bromophenyl)-5-chloro-benzamide

4-Bromoaniline (1 eq, 45.2 mmol, 7.77 g), 5-chloroisatoic anhydride (1.1 eq, 49.7 mmol, 9.82 g) and sodium hydroxide (0.1 eq, 4.51 mmol, 0.18 g) were combined under an inert nitrogen atmosphere. Dioxane (anhydrous grade, 94 mL) was added and the mixture heated to reflux for 18 h. The reaction mixture was allowed to cool to room temperature, filtered and concentrated under reduced pressure. The residue was triturated from ethanol and dried under vacuum to yield 2-amino-*N*-(4-bromophenyl)-5-chloro-benzamide (5.74 g, 39%).

^{1}H NMR (500 MHz, DMSO-d_6) δ 10.19 (s, 1H), 7.71 – 7.66 (m, 3H), 7.56 – 7.49 (m, 2H), 7.24 (dd, J = 8.8, 2.5 Hz, 1H), 6.79 (d, J = 8.8 Hz, 1H), 6.47 (s, 2H).

^{13}C NMR (126 MHz, DMSO-d_6) δ 166.6, 148.7, 138.4, 132.0, 131.3, 127.8, 122.5, 118.1, 117.8, 115.6, 115.3.

HRMS (ESI) obtained m/z 346.9538, 348.9531 (M+Na$^+$). Expected 346.9557, 348.9535.

^{1}H NMR

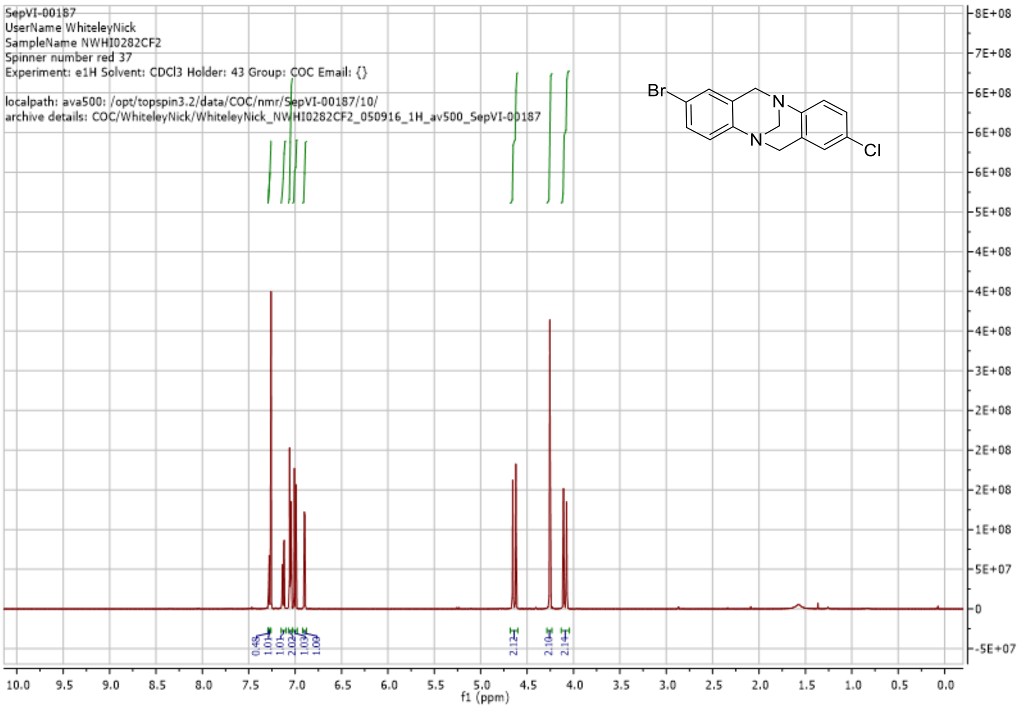

^{13}C NMR

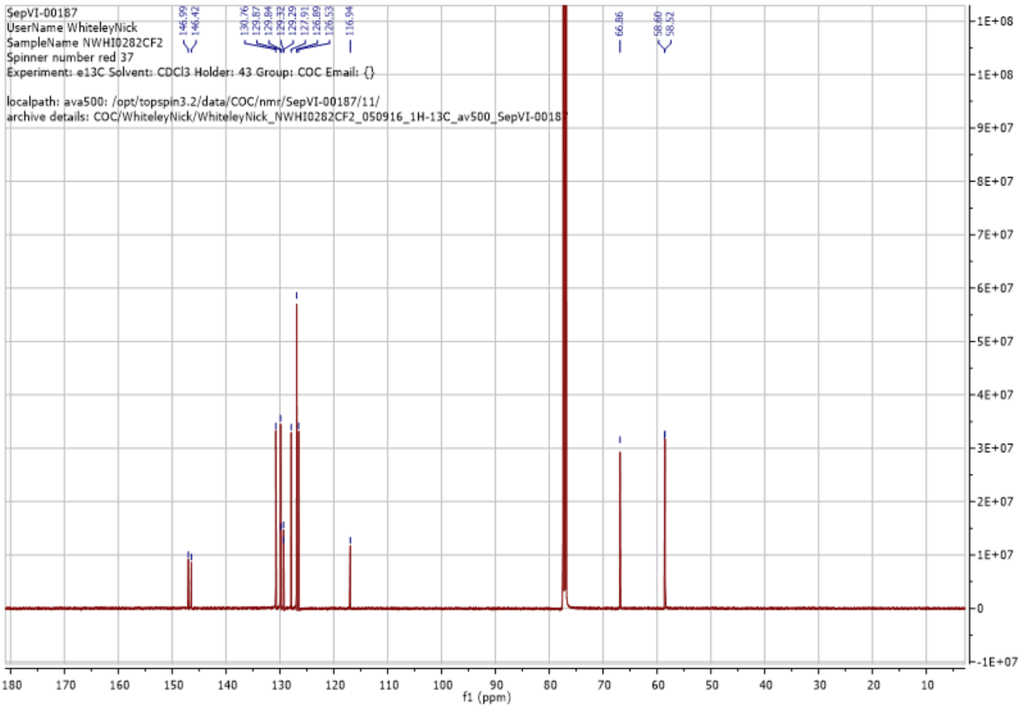

Steps II-III. 2-Bromo-8-chloro-6*H*,12*H*-5,11-methanodibenzo[*b,f*][1,5]diazocine

2-amino-4-bromo-*N*-(4-chlorophenyl)benzamide (1 eq, 14.3 mmol, 4.65 g) was dissolved in THF (24 mL) and cooled to 0 °C. BH_3.THF (5.8 eq, 1.0 M in THF, 82.9 mmol, 83 mL) was added slowly and the mixture stirred at 0 °C for 30 minutes. The reaction was then slowly heated to reflux and refluxed for 20 h. The solution was then added slowly to 1.0 M aqueous sodium hydroxide (200 mL) at 0°C. The resulting mixture was extracted with ethyl acetate (3 x 150 mL). Combined organics were washed with brine (150 mL), dried over magnesium sulfate and solvents removed under reduced pressure to yield crude (~90% pure) 2-[(4-bromoanilino)methyl]-4-chloro-aniline in quantitative yield (4.17 g).

2-[(4-bromoanilino)methyl]-4-chloro-aniline (1 eq, 21.1 mmol, 6.59 g) and paraformaldehyde (6 eq, 126.9 mmol, 3.81 g) were combined in TFA (350 mL) under an inert nitrogen atmosphere. The reaction was stirred at room temperature for 30 minutes and then heated to 40 °C for 24 h. The reaction mixture was added dropwise to 35% aqueous ammonia (500 mL) at 0 °C and extracted with DCM (3 x 300 mL). Combined organics dried over magnesium sulfate and solvents removed under reduced pressure. The residue was purified by flash chromatography (10-40% ethyl acetate in petroleum ether gradient) to yield 2-bromo-8-chloro-6*H*,12*H*-5,11-methanodibenzo[*b,f*][1,5]diazocine (4.40 g, 62%).

^{1}H NMR (500 MHz, CDCl3) δ 7.27 (dd, J = 8.6, 2.4 Hz, 1H), 7.13 (dd, J = 8.6, 2.4 Hz, 1H), 7.06 – 7.04 (m, 2H), 7.00 (d, J = 8.6 Hz, 1H), 6.90 (d, J = 2.4 Hz, 1H), 4.64 (app dd, J = 16.8, 0.8 Hz, 2H), 4.25 (app t, J = 1.2 Hz, 2H), 4.09 (app d, J = 16.8 Hz, 2H).

^{13}C NMR (126 MHz, CDCl3) δ 147.0, 146.4, 130.8, 129.9, 129.8, 129.3, 129.3, 127.9, 126.9, 126.5, 116.9, 66.7, 58.6, 58.5. Two peaks appear to coalesce at 129.9 ppm.

HRMS (ESI) obtained m/z 334.9948, 336.9931 ($M+H^+$). Expected 334.9945, 336.9923.

^{1}H NMR

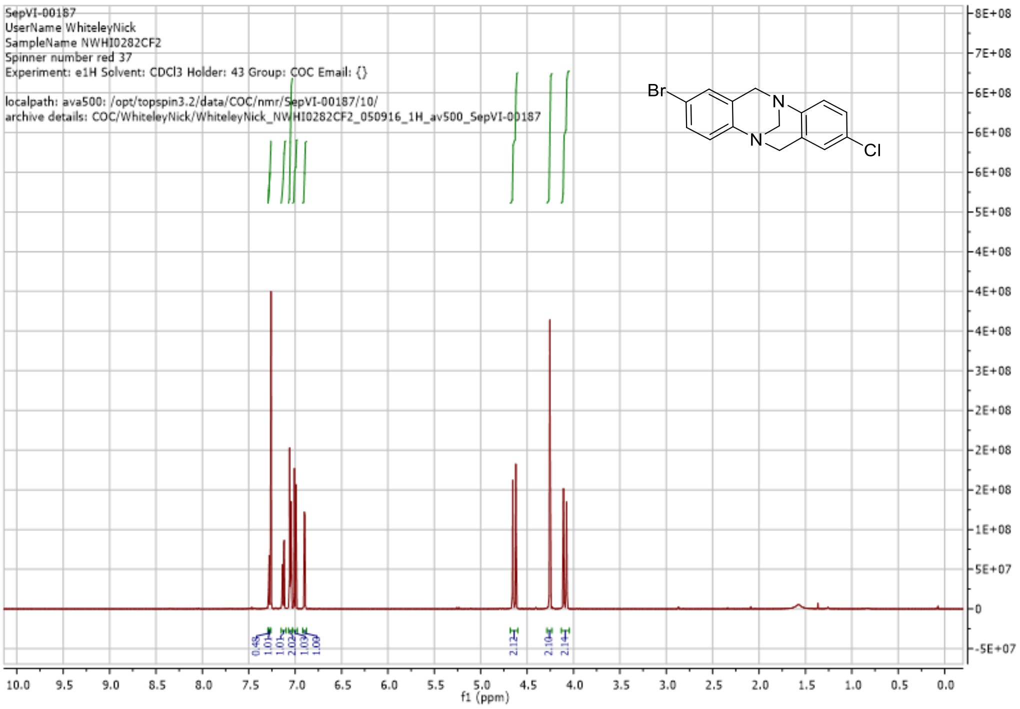

^{13}C NMR

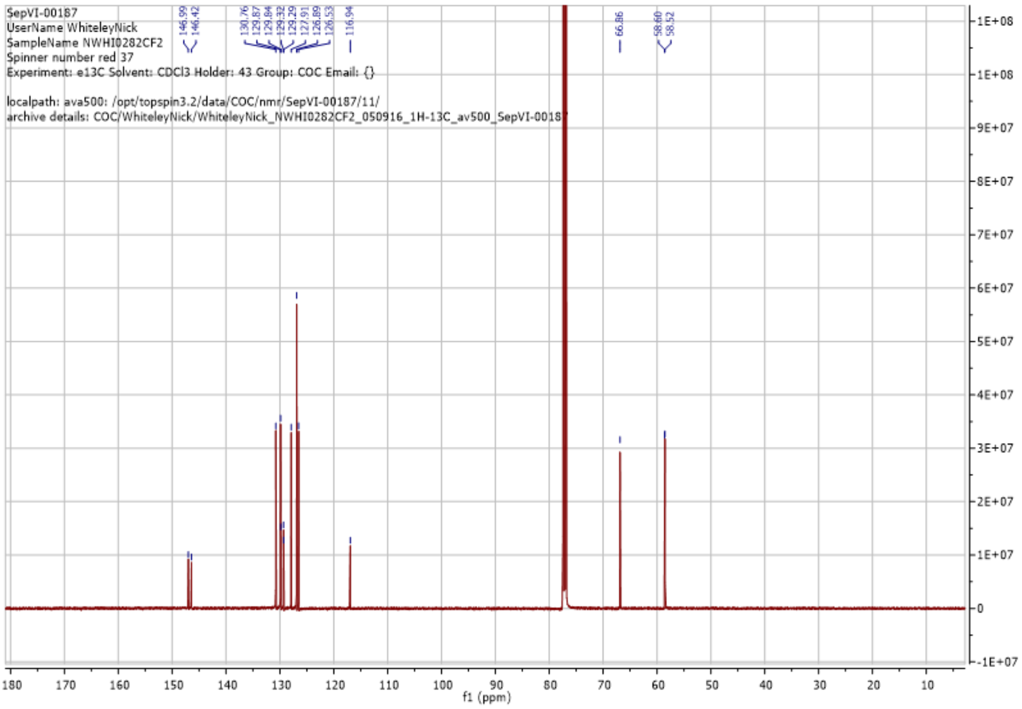

Step IV: 2-Chloro-8-phenyl-6*H*,12*H*-5,11-methanodibenzo[*b,f*][1,5]diazocine

2-Bromo-8-chloro-6*H*,12*H*-5,11-methanodibenzo[*b,f*][1,5]diazocine (1 eq, 13.1 mmol, 4.40 g), phenylboronic acid (1.5 eq, 20.0 mmol, 2.40 g), Pd(dppf)Cl_2.DCM (0.04 eq, 0.524 mmol, 0.43 g), K_3PO_4 (2 eq, 26.2 mmol, 5.56 g) and water (10 eq, 131 mmol, 2.10 g, 2.1 mL) were combined in THF (65 mL) under an inert nitrogen atmosphere. The mixture was heated to 50 °C for 24 h. The reaction mixture was filtered through kieselguhr eluting with ethyl acetate (250 mL). Organics were washed with sodium bicarbonate (100 mL), brine (100 mL), dried over magnesium sulfate and solvents removed under reduced pressure. The residue was purified by flash chromatography (10-20%) ethyl acetate in petroleum ether gradient to yield 2-chloro-8-phenyl-6*H*,12*H*-5,11-methanodibenzo[*b,f*][1,5]diazocine (3.53 g, 81%) and further crude product (0.80 g).

^{1}H NMR (500 MHz, $CDCl_3$) δ 7.50 – 7.47 (m, 2H), 7.42 – 7.36 (m, 3H), 7.32 – 7.27 (m, 1H), 7.19 (app d, J = 8.3 Hz, 1H), 7.15 – 7.11 (m, 2H), 7.09 (app d, J = 8.6 Hz, 1H), 6.93 (d, J = 1.9 Hz, 1H), 4.75 (d, J = 16.7 Hz, 1H), 4.69 (d, J = 16.7 Hz, 1H), 4.33 (app q, J = 12.9 Hz, 2H), 4.19 (app d, J = 16.7 Hz, 2H).

^{13}C NMR (126 MHz, $CDCl_3$) δ 147.3, 146.8, 140.8, 137.4, 129.7, 129.1, 128.9, 127.9, 127.8, 127.2, 127.0, 126.9, 126.6, 126.5, 125.7, 125.5, 67.1, 59.0, 58.6.

HRMS (ESI) obtained m/z 333.1145 ($M+H^+$). Expected 333.1153.

^{1}H NMR

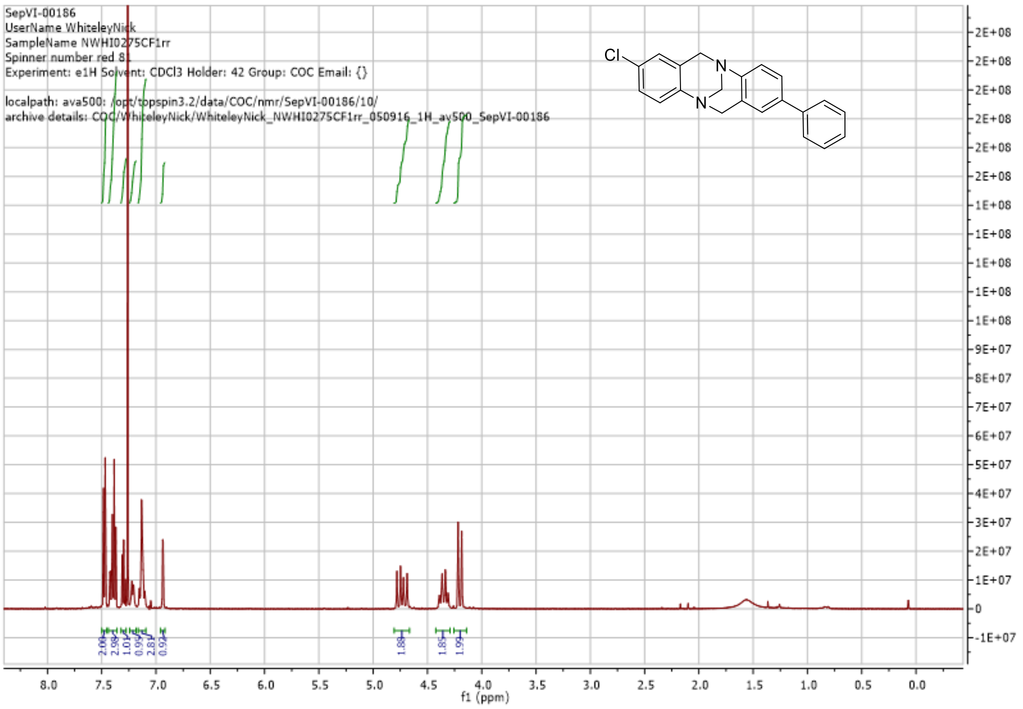

^{13}C NMR

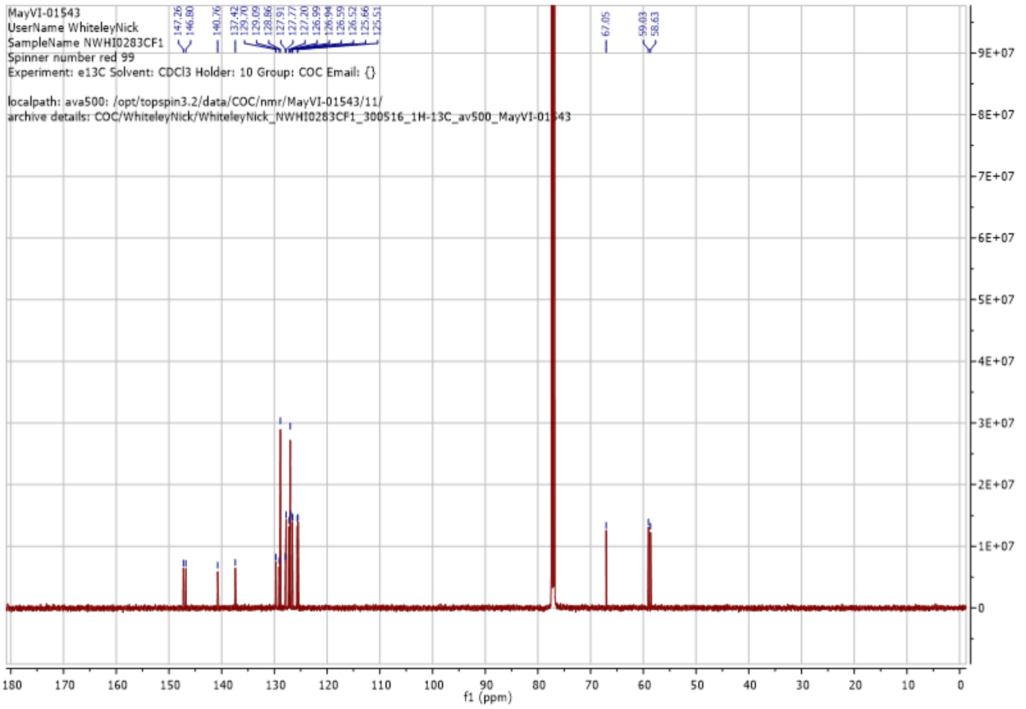

Step V. 2-(Bispinacolatodiboron)-8-phenyl-6*H*,12*H*-5,11-methanodibenzo[*b,f*][1,5]diazocine

2-Chloro-8-phenyl-6*H*,12*H*-5,11-methanodibenzo[*b,f*][1,5]diazocine (1 eq, 10.4 mmol, 3.50 g), $(BPin)_2$ (3 eq, 31.1 mmol, 7.90 g), $Pd_2(dba)_3$ (0.02 eq, 0.21 mmol, 0.19 g), SPhos (0.04 eq, 0.41 mmol, 0.17 g) and potassium acetate (3 eq, 31.1 mmol, 3.05 g) were combined in degassed dioxane (7.3 mL) under an inert nitrogen atmosphere. The reaction was heated to reflux for 16 h. The reaction mixture was cooled and filtered through kieselguhr eluting with ethyl acetate (200 mL). Organics were washed with saturated sodium bicarbonate (150 mL), brine (50 mL) and then dried over magnesium sulfate. Solvents were removed under reduced pressure and the residue purified by flash chromatography (10-25% ethyl acetate in petroleum ether) to yield 2-(bispinacolatodiboron)-8-phenyl-6*H*,12*H*-5,11-methanodibenzo[*b,f*][1,5]diazocine (3.50 g, 80%).

^{1}H NMR (500 MHz, $CDCl_3$) δ 7.62 (dd, J = 8.0, 1.1 Hz, 1H), 7.48 – 7.44 (m, 2H), 7.43 (s, 1H), 7.39 – 7.35 (m, 3H), 7.30 – 7.26 (m, 1H), 7.17 (app dd, J = 12.9, 8.2 Hz, 2H), 7.10 (d, J = 2.0 Hz, 1H), 4.80 – 4.69 (m, 2H), 4.36 (s, 2H), 4.26 (app t, J = 16.9 Hz, 2H), 1.29 (s, 12H).

^{13}C NMR (126 MHz, $CDCl_3$) δ 151.3, 147.4, 140.9, 137.2, 134.1, 133.9, 128.8, 128.1, 127.3, 127.1, 127.0, 126.4, 125.7, 125.5, 124.5, 83.8, 67.1, 59.0, 58.8, 25.0. No peak observed for boron bound carbon.

HRMS (ESI) obtained m/z 447.2197 ($M+Na^+$). Expected 447.2219.

^{1}H NMR

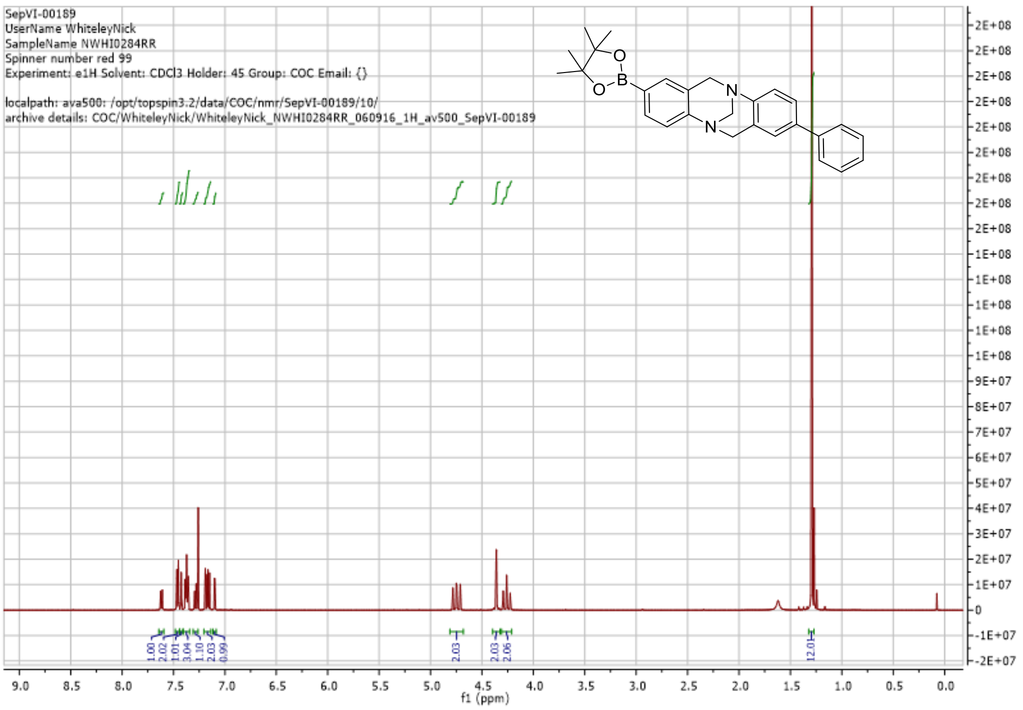

^{13}C NMR

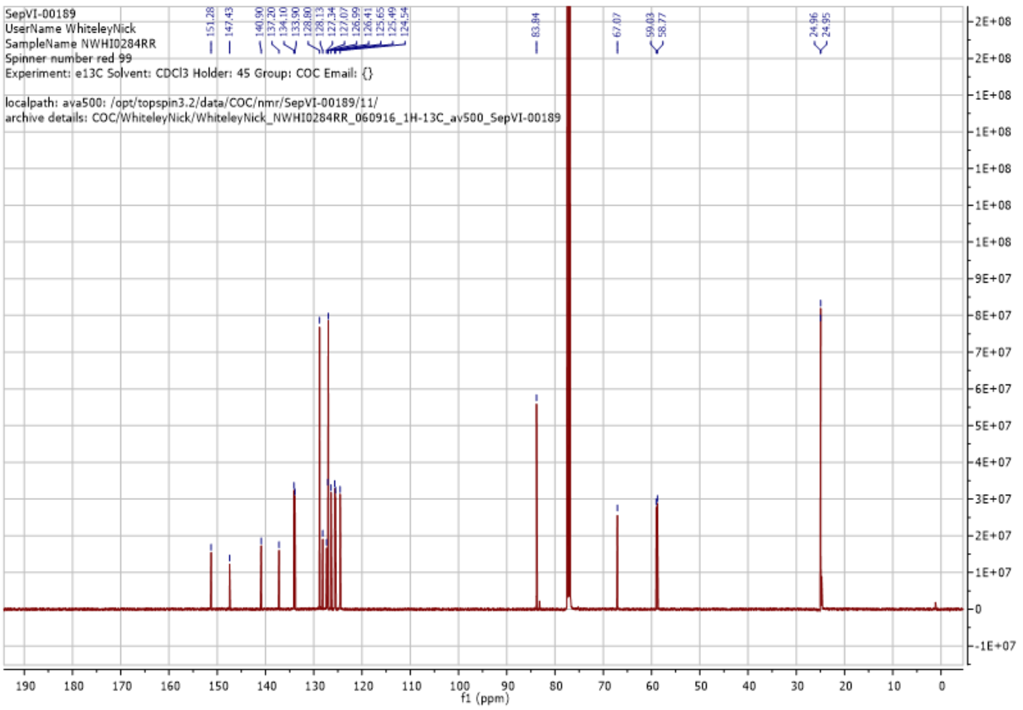

Step VI- VII. 3-methyl-2-[8-(phenyl)-6*H*,12*H*-5,11-methano dibenzo[*b,f*][1,5]diazocin-2-yl]benzoic acid

2-(Bispinacolatodiboron)-8-phenyl-6*H*,12*H*-5,11-methanodibenzo[*b,f*][1,5]diazocine (1 eq, 8.09 mmol, 3.43 g), methyl 2-bromo-3-methyl-benzoate (1.5 eq, 12.1 mmol, 2.78 g), $Pd(OAc)_2$ (0.02 Eq, 0.162 mmol, 0.036 g) and SPhos (0.04 eq, 0.323 mmol, 0.13 g) were combined in acetonitrile (27 mL). Aqueous potassium carbonate solution (2 M, 2.5 eq, 20.2 mmol, 10 mL) was added and the reaction heated to reflux under an inert nitrogen atmosphere for 16 h. The reaction mixture was diluted with ethyl acetate (250 mL), washed with saturated aqueous sodium bicarbonate (2 x 150 mL) and then brine (2 x 100 mL). Organics were dried over magnesium sulfate and solvents removed under reduced pressure. The residue was purified by flash chromatography (10-40% ethyl acetate in petroleum ether) to yield product which was further purified by trituration from methanol to yield methyl 3-methyl-2-[8-(phenyl)-6*H*,12*H*-5,11-methano dibenzo[*b,f*][1,5]diazocin-2-yl]benzoate (3.40 g, 94%).

Methyl 3-methyl-2-[8-(trifluoromethyl)-6*H*,12*H*-5,11-methano dibenzo[*b,f*][1,5]diazocin-2-yl]benzoate (1 eq, 0.208 mmol, 1.31 g) and lithium hydroxide (17 eq, 49.8 mmol, 2.10 g) were combined in methanol (44 mL), THF (44 mL) and water (0.4 mL) and heated to 50 °C for 40 h. Solvents were removed under reduced pressure and the residue taken up in saturated aqueous ammonium chloride (100 mL). 1 M Hydrochloric acid (17 eq, 49.8 mmol, 48 mL) was added to neutralize and the mixture was extracted with ethyl acetate (3 x 100 mL). Organics were washed with brine (50 mL), dried over magnesium sulfate and solvents were removed under reduced pressure to give 3 methyl-2-[8-(phenyl)-6*H*,12*H*-5,11-methano dibenzo[*b,f*][1,5]diazocin-2-yl]benzoic acid in quantitative yield (1.30 g).

^{1}H NMR (500 MHz, $CDCl^3$) δ 7.64 (d, J = 7.1 Hz, 1H), 7.50 (app d, J = 7.5 Hz, 2H), 7.46 (app d, J = 6.9 Hz, 1H), 7.42 – 7.37 (m, 3H), 7.35 – 7.27 (m, 3H), 7.24 – 7.12 (m, 3H), 6.85 (s, 1H), 4.80 (app d, J = 16.8 Hz, 1H), 4.52 (app d, J = 17.0 Hz, 1H), 4.32 – 4.14 (m, 3H), 3.86 (app d, J = 12.8 Hz, 1H), 2.07 (s, 3H).

^{13}C NMR (126 MHz, $CDCl_3$) δ 172.2, 145.6, 145.1, 140.4, 139.9, 138.2, 137.8, 136.9, 134.1, 132.6, 129.1, 129.0, 127.7, 127.4, 127.4, 127.3, 127.0, 126.7, 126.6, 126.5, 125.7, 125.5, 124.4, 65.8, 58.1, 57.8, 20.9.

HRMS (ESI) obtained m/z 455.1740 ($M+Na^+$). Expected 455.1730.

1H NMR

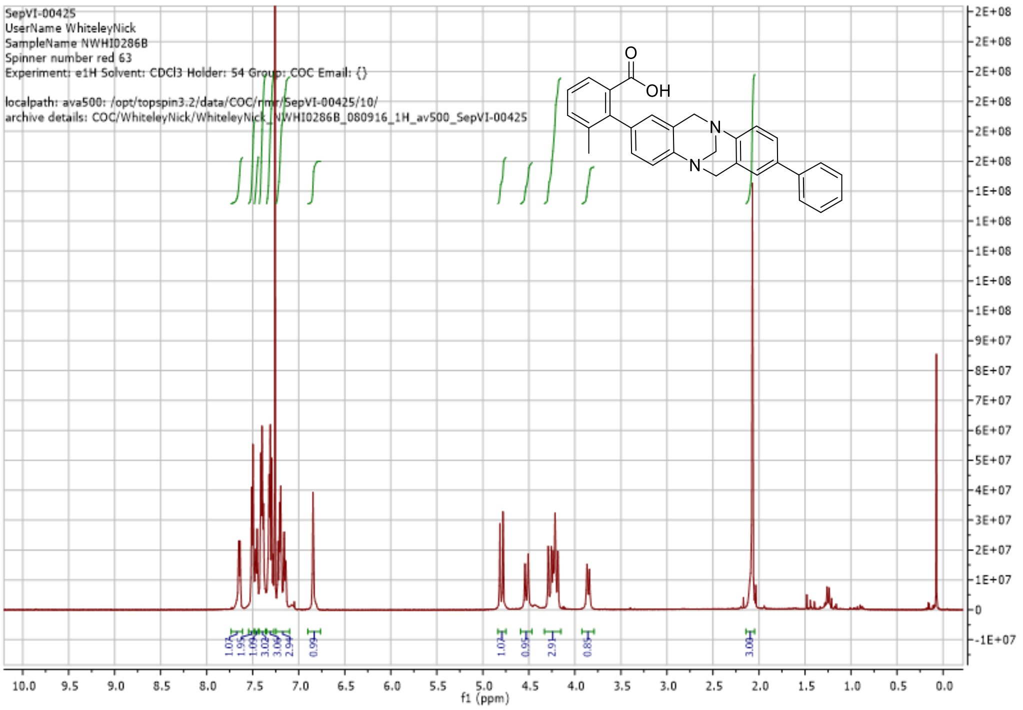

13C NMR

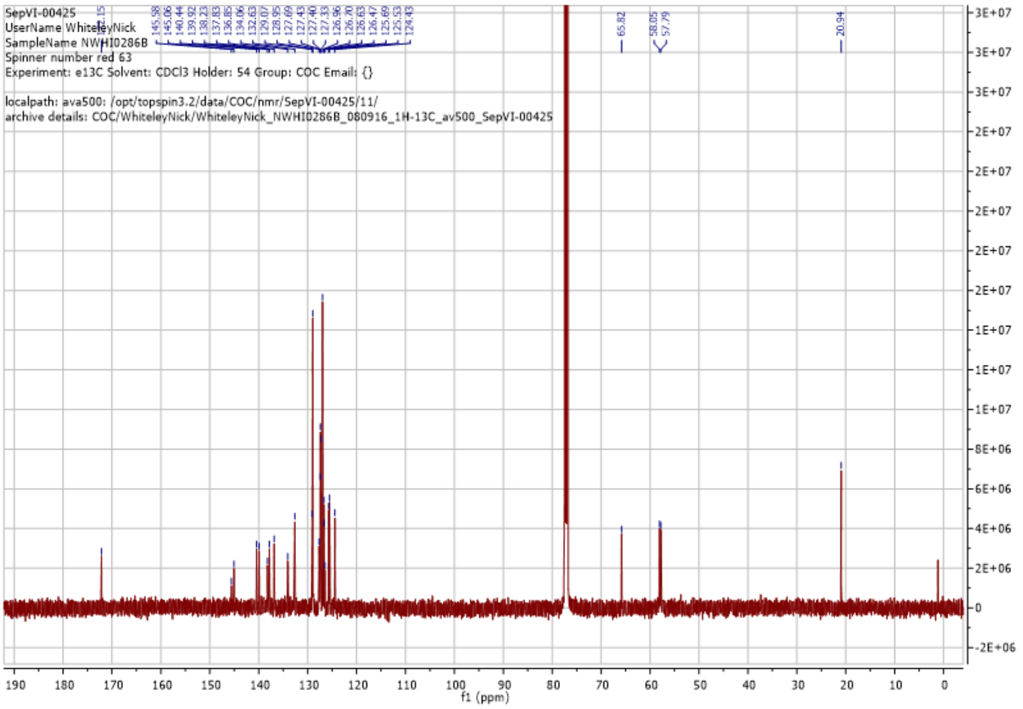

Step VIII (1F). 4-Fluorophenyl 3-methyl-2-[8-(phenyl)-6*H*,12*H*-5,11-methano dibenzo[*b,f*][1,5]diazocin-2-yl]benzoate

3 Methyl-2-[8-(phenyl)-6*H*,12*H*-5,11-methano dibenzo[*b,f*][1,5]diazocin-2-yl]benzoic acid (1 eq, 0.462 mmol, 0.20 g), 4-fluorophenol (3 eq, 1.39 mmol, 0.16 g) and PyBOP (2 eq, 0.924 mmol, 0.48 g) were combined in DCM (11 mL). Triethylamine (8 eq, 3.70 mmol, 0.37 g, 0.515 mL) was added and the stirred at room temperature for 48 h. Further DCM was added (50 mL) and the solution washed with saturated aqueous sodium bicarbonate (40 mL). Organics were dried over magnesium sulfate and solvents removed under reduced pressure. The residue was purified by flash chromatography (20-40% ether in hexane) to yield 4-fluorophenyl 3-methyl-2-[8-(phenyl)-6*H*,12*H*-5,11-methano dibenzo[*b,f*][1,5]diazocin-2-yl]benzoate (0.14 mg, 55%).

^{1}H NMR (600 MHz, $CDCl_3$) δ 7.74 (d, J = 7.5 Hz, 0.3H, minor conformer), 7.65 (d, J = 7.6 Hz, 0.6H, major conformer), 7.57 – 7.51 (m, 2H), 7.49 – 7.38 (m, 4H), 7.34 (app. t, J = 7.5 Hz, 1.6H), 7.30 (app. t, J = 7.4 Hz, 0.4H), 7.24 – 7.19 (m, 3H), 7.12 (app. dd, J = 8.2, 1.5 Hz, 0.6H, major conformer), 7.09 (app. d, J = 8.2 Hz, 0.4H, minor conformer), 6.99 (app. t, J = 8.5 Hz, 0.7H, minor conformer), 6.84 (app. d, J = 0.8 Hz, 0.7H, major conformer), 6.80 (app. s, 0.3H, minor conformer), 6.74 – 6.71 (m, 0.6H, minor conformer), 6.41 (app. t, J = 8.6 Hz, 1.3H, major conformer), 6.08 – 6.04 (m, 1.2H, major conformer), 4.86 – 4.76 (m, 1.7H), 4.69 (d, J = 16.8 Hz, 0.4H, minor conformer), 4.42 – 4.18 (m, 4H), 2.16 (s, 2H, major conformer), 2.09 (s, 1H, minor conformer).

^{13}C NMR (126 MHz, $CDCl_3$) δ 168.2, 167.6, 160.3 (d, J = 244.4 Hz), 159.9 (d, J = 244.4 Hz), 147.7, 147.6, 147.4, 146.6 (d, J = 2.5 Hz), 146.0 (d, J = 2.6 Hz), 141.2, 140.7, 140.5, 137.7, 137.5 , 137.1, 136.2, 135.9, 133.7, 133.5, 131.9, 131.8, 129.0, 128.9, 128.6, 128.3, 128.3, 128.1, 127.7, 127.5, 127.3, 127.3, 127.2, 127.1, 127.1, 126.9, 126.5, 126.3, 125.9, 125.7, 125.6, 125.5, 125.0, 122.8 (d, J = 8.4 Hz), 122.2 (d, J = 8.5 Hz), 116.0 (d, J = 23.8 Hz), 115.8 (d, J = 23.7 Hz), 67.1, 67.0, 58.8, 58.7, 58.6, 58.5, 21.0, 20.9. Two conformers. Some aryl conformer peaks occluded.

^{19}F NMR (376 MHz, 1H decoupled, $CDCl_3$) δ -116.80 (s, 0.7F), -117.08 (s, 0.3F).

HRMS (ESI) obtained m/z 549.1935 ($M+Na^+$). Expected 549.1949.

^{1}H NMR

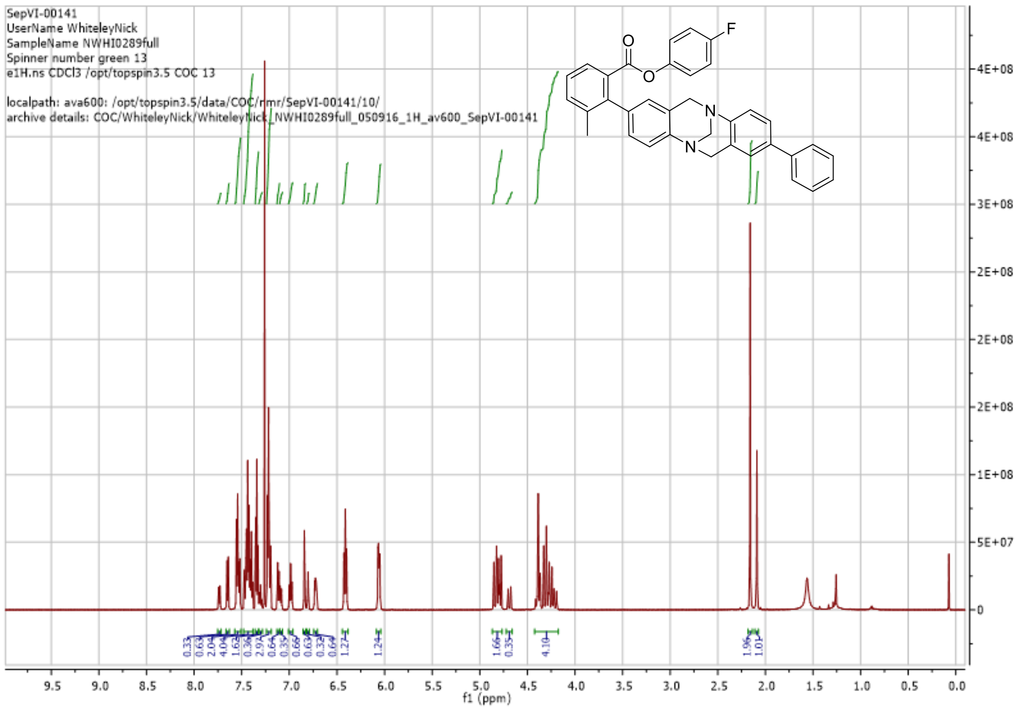

^{13}C NMR

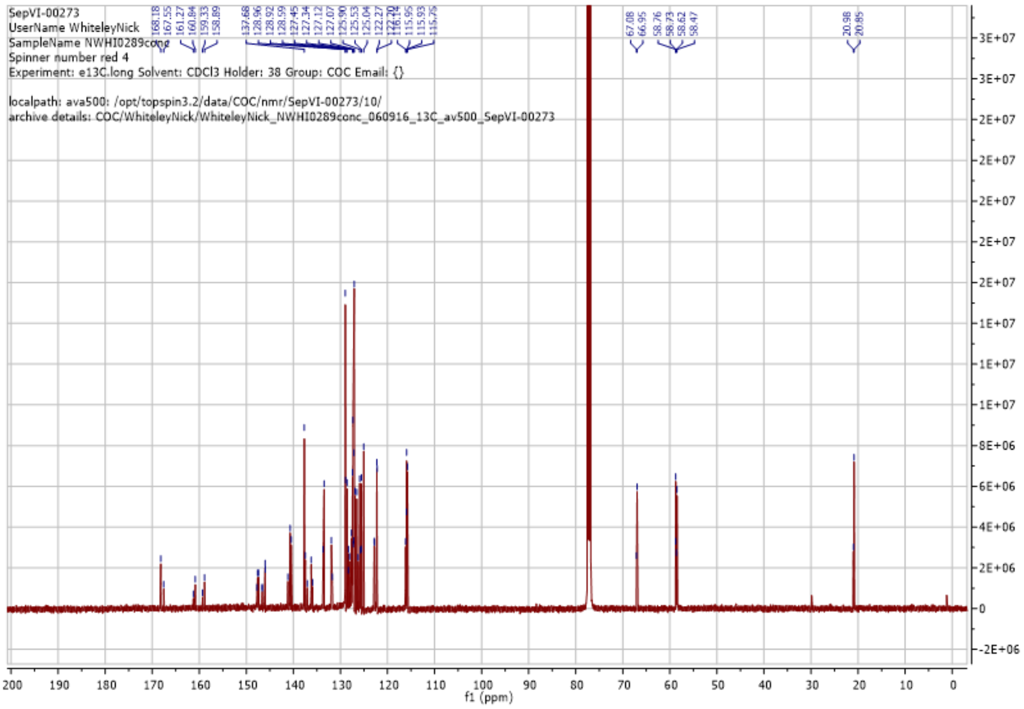

Step VIII (1Cl). 4-Chlorophenyl 3-methyl-2-[8-(phenyl)-6*H*,12*H*-5,11-methano dibenzo[*b,f*][1,5]diazocin-2-yl]benzoate

3 Methyl-2-[8-(phenyl)-6*H*,12*H*-5,11-methano dibenzo[*b,f*][1,5]diazocin-2-yl]benzoic acid (1 eq, 0.391 mmol, 0.17 g), 4-chlorophenol (3 eq, 1.17 mmol, 0.15 g) and PyBOP (2 eq, 0.781 mmol, 0.41 g) were combined in DCM (11 mL). Triethylamine (8 eq, 3.70 mmol, 0.37 mg, 0.515 mL) was added and the reaction stirred at room temperature for 48 h. Further DCM was added (50 mL) and the solution washed with saturated aqueous sodium bicarbonate 40 mL). Organics were dried over magnesium sulfate and solvents removed under reduced pressure. The residue was purified by flash chromatography (20-50% ether in hexane) to yield 4-chlorophenyl 3-methyl-2-[8-(phenyl)-6*H*,12*H*-5,11-methano dibenzo[*b,f*][1,5]diazocin-2-yl]benzoate (0.13 mg, 59%).

^{1}H NMR (500 MHz, $CDCl_3$) δ 7.76 (d, J = 7.5 Hz, 0.3H, minor conformer), 7.68 (d, J = 7.6 Hz, 0.7H, major conformer), 7.61 (app d, J = 7.6 Hz, 1.4H, major confomer), 7.55 (app d, J = 7.6 Hz, 1.4H, major conformer), 7.52 (dd, J = 8.3, 1.7 Hz, 0.7H), 7.48 – 7.40 (m, 3.5H), 7.39 – 7.30 (m, 2.5H), 7.27 – 7.20 (m, 2.4H), 7.15 – 7.09 (m, 1H), 6.84 (app d, J = 21.3 Hz, 1H), 6.73 (d, J = 8.7 Hz, 2H), 6.07 (app d, J = 8.7 Hz, 1.3H), 4.89 – 4.77 (m, 1.7H), 4.70 (app d, J = 16.8 Hz, 0.3H), 4.45 – 4.20 (m, 4H), 2.19 (s, 2H), 2.12 (s, 1H).

^{13}C NMR (126 MHz, $CDCl_3$) δ 168.2, 167.5, 149.5, 148.9, 147.9, 147.7, 141.5, 141.0, 140.5, 138.0, 137.6, 137.3, 136.4, 136.1, 134.0, 133.8, 132.0, 131.9, 131.5, 131.3, 129.7, 129.5, 129.3, 129.2, 128.8, 128.6, 128.5, 128.3, 128.0, 127.9, 127.7, 127.6, 127.5, 127.3, 127.1, 126.7, 126.5, 126.1, 126.0, 125.9, 125.6, 125.3, 123.0, 122.5, 67.3, 67.2, 59.0, 58.9, 58.8, 21.2, 21.1. Two conformers, some aromatic and one Tröger's base bridgehead conformer peaks occluded.

HRMS (ESI) obtained m/z 543.1819 ($M+H^+$). Expected 543.1834.

^{1}H NMR

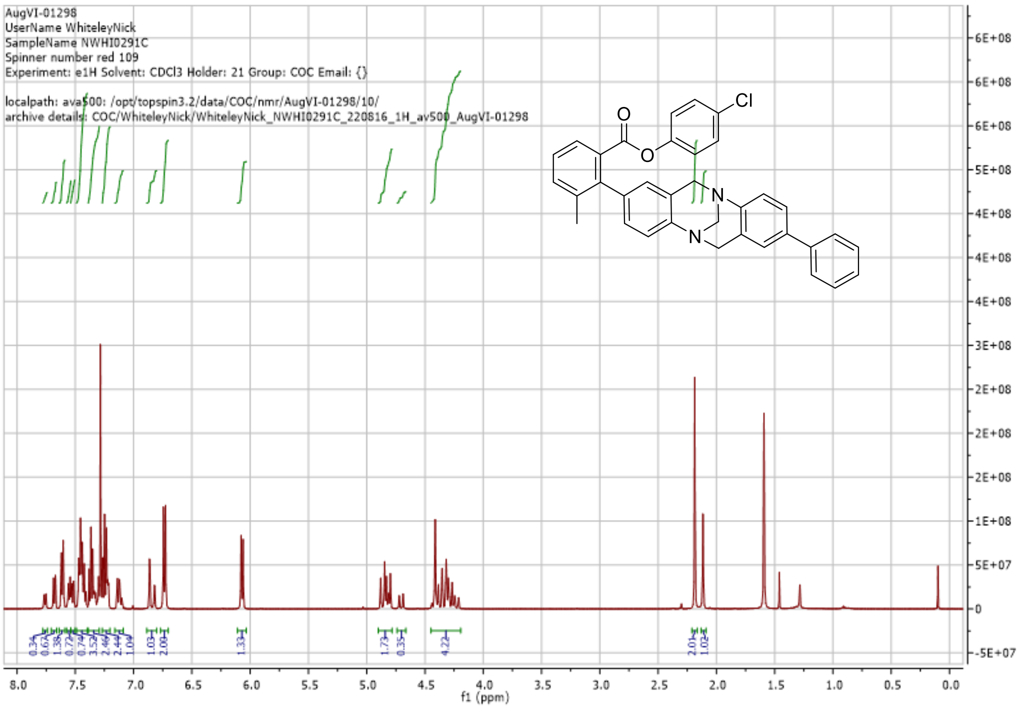

^{13}C NMR

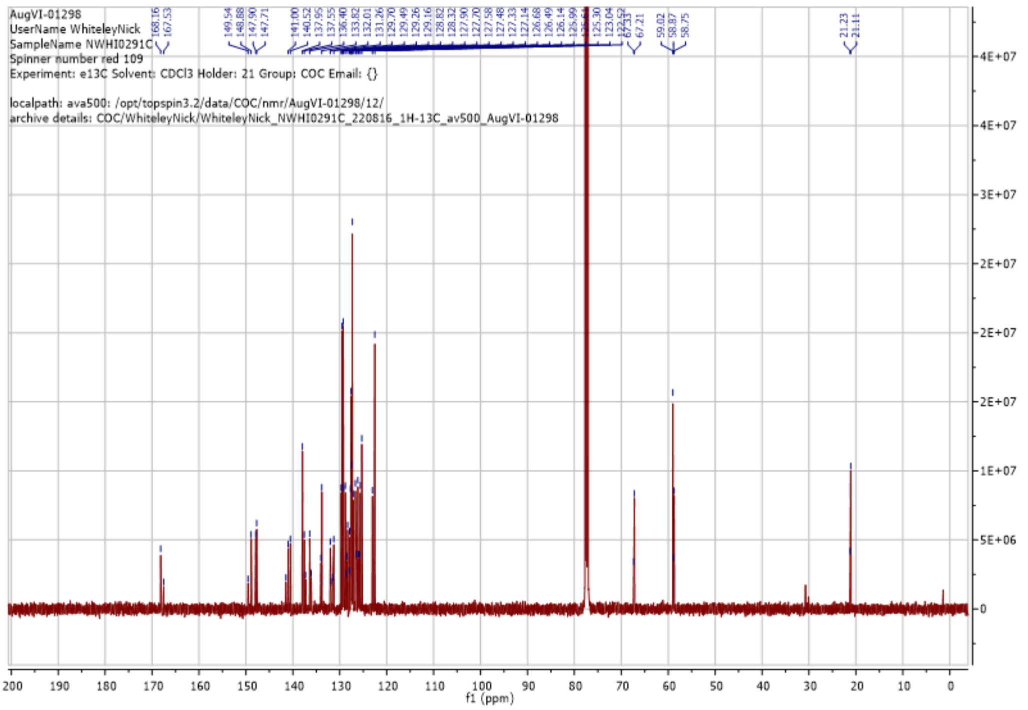

Step VIII (1Br). 4-Bromophenyl 3-methyl-2-[8-(phenyl)-6*H*,12*H*-5,11-methano dibenzo[*b,f*][1,5]diazocin-2-yl]benzoate

3 Methyl-2-[8-(phenyl)-6*H*,12*H*-5,11-methano dibenzo[*b,f*][1,5]diazocin-2-yl]benzoic acid (1 eq, 0.462 mmol, 0.20 g), 4-bromophenol (3 eq, 0.924 mmol, 0.48 g) and PyBOP (2 eq, 0.924 mmol, 0.48 g) were combined in DCM (11 mL). Triethylamine (8 eq, 3.70 mmol, 0.37 g, 0.515 mL) was added and the stirred at room temperature for 48 h. Further DCM was added (50 mL) and the solution washed with saturated aqueous sodium bicarbonate 40 mL). Organics were dried over magnesium sulfate and solvents removed under reduced pressure. The residue was purified by flash chromatography (20-40% ether in hexane) to yield 4-bromophenyl 3-methyl-2-[8-(phenyl)-6*H*,12*H*-5,11-methanodibenzo[*b,f*][1,5]diazocin-2-yl]benzoate (0.16 g, 59%).

^{1}H NMR (600 MHz, $CDCl_3$) δ 7.73 (d, J = 7.6 Hz, 0.3H, minor conformer), 7.65 (d, J = 7.5 Hz, 0.6H, major conformer), 7.60 (d, J = 7.4 Hz, 1.3H), 7.54 – 7.49 (m, 1.3H), 7.46 – 7.38 (m, 4H), 7.36 – 7.29 (m, 2H), 7.28 – 7.26 (m, 0.7H), 7.25 – 7.18 (m, 2.3H), 7.13 – 7.06 (m, 1H), 6.86 (d, J = 8.7 Hz, 1.3H, major conformer), 6.84 (d, J = 1.1 Hz, 0.6H, major conformer), 6.79 (app. s, 0.3H, minor conformer) ,6.65 (d, J = 8.7 Hz, 0.7H, minor conformer), 5.99 (d, J = 8.7 Hz, 1.2H, major conformer), 4.87 – 4.76 (m, 1.7H), 4.68 (app. d, J = 16.8 Hz, 0.3H, minor conformer), 4.43 – 4.17 (m, 4H), 2.16 (s, 2H, major conformer), 2.09 (s, 1H, minor conformer).

^{13}C NMR (126 MHz, $CDCl_3$) δ 167.8, 167.2, 149.9, 149.2, 147.8, 147.8, 147.6, 147.4, 141.2, 140.8, 140.7, 140.7, 140.2, 137.7, 137.3, 137.0, 136.1, 135.9, 133.8, 133.6, 132.4, 132.2, 131.7, 131.6, 129.1, 128.9, 128.6, 128.4, 128.3, 128.1, 127.7, 127.6, 127.5, 127.3, 127.3, 127.1, 126.9, 126.4, 126.2, 125.9, 125.7, 125.6, 125.3, 125.1, 123.2, 122.7, 119.0, 118.8, 67.1, 67.0, 58.8, 58.6, 58.5, 21.0, 20.9. Two conformers, some aromatic and one Tröger's base bridgehead conformer peaks occluded.

HRMS (ESI) obtained m/z 609.1101, 611.1086 (M+Na$^+$). Expected 609.1148, 611.1134.

^{1}H NMR

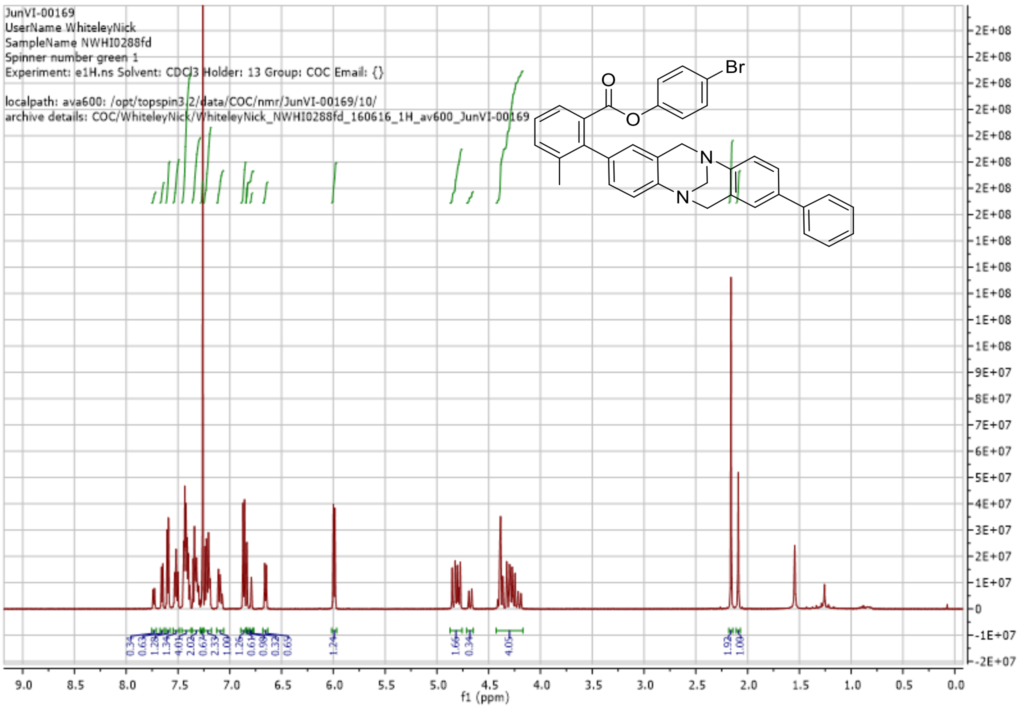

^{13}C NMR

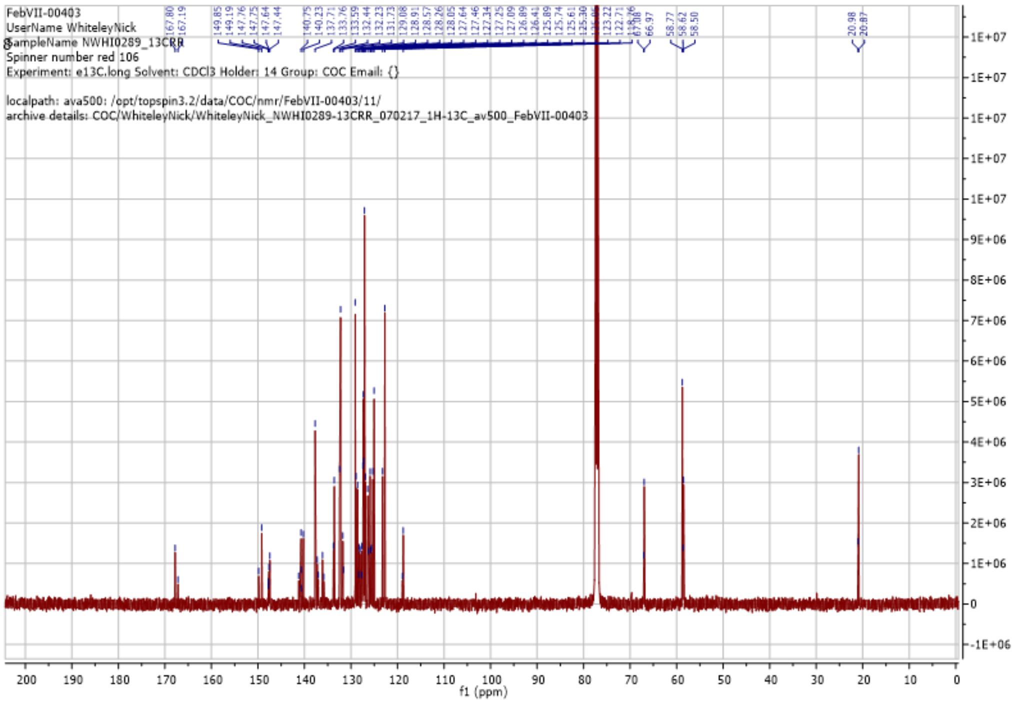

Step VIII (1l). 4-Iodophenyl 3-methyl-2-[8-(phenyl)-6*H*,12*H*-5,11-methano dibenzo[*b,f*][1,5]diazocin-2-yl]benzoate

3 Methyl-2-[8-(phenyl)-6*H*,12*H*-5,11-methano dibenzo[*b,f*][1,5]diazocin-2-yl]benzoic acid (1 eq, 0.201 mmol, 0.087 g), 4-iodophenol (3 eq, 0.603 mmol, 0.13 g) and PyBOP (2 eq, 0.402 mmol, 0.21 g) were combined in DCM (5 mL). Triethylamine (8 Eq, 1.61 mmol, 0.16 g, 0.225 mL) was added and the reaction stirred at room temperature for 72 h. Further DCM was added (50 mL) and the solution washed with saturated aqueous sodium bicarbonate (30 mL) and brine (2 x 30 mL). Organics were dried over magnesium sulfate and solvents removed under reduced pressure. The residue was purified by flash chromatography (20-40% ethyl acetate in petroleum ether) to yield 4-iodophenyl 3-methyl-2-[8-(phenyl)-6*H*,12*H*-5,11-methano dibenzo[*b,f*][1,5]diazocin-2-yl]benzoate (0.067 g, 54%).

^{1}H NMR (601 MHz, $CDCl_3$) δ 7.75 (d, J = 7.6 Hz, 0.3H, minor conformer), 7.68 (d, J = 7.5 Hz, 0.7H, major conformer), 7.65 – 7.61 (m, 2H), 7.56 – 7.53 (m, 1.3H), 7.49 – 7.40 (m, 3.5H), 7.38 – 7.29 (m, 2.7H), 7.27 – 7.20 (m, 2.4H), 7.14 – 7.08 (m, 2.3H), 6.86 (d, J = 1.3 Hz, 0.6H, major conformer), 6.81 (app. s, 0.3H, minor conformer), 6.56 (app d, J = 8.7 Hz, 0.7H, minor conformer), 5.90 (app. d, J = 8.7 Hz, 1.3H, major conformer), 4.88 – 4.78 (m, 1.7H), 4.70 (d, J = 16.8 Hz, 0.3H, minor conformer), 4.44 – 4.19 (m, 4H), 2.18 (s, 2H, major conformer), 2.11 (s, 1H, minor conformer).

^{13}C NMR (126 MHz, $CDCl_3$) δ 167.6, 167.0, 150.5, 149.9, 147.6, 147.5, 147.3, 141.1, 140.6, 140.6, 140.1, 138.3, 138.1, 137.6, 137.0, 136.9, 136.0, 135.7, 133.6, 133.5, 131.6, 131.5, 129.1, 128.8, 128.4, 128.2, 128.1, 127.9, 127.6, 127.5, 127.3, 127.2, 127.2, 127.1, 127.1, 127.0, 126.8, 126.2, 126.1, 125.7, 125.6, 125.5, 125.1, 124.9, 123.5, 123.0, 89.7, 89.5, 66.9, 66.8, 58.6, 58.5, 58.4, 20.8, 20.7. Two conformers, some aromatic and one Tröger's base bridgehead conformer peaks occluded.

HRMS (ESI) obtained m/z 635.1179 ($M+H^{+}$). Expected 635.1190.

^{1}H NMR

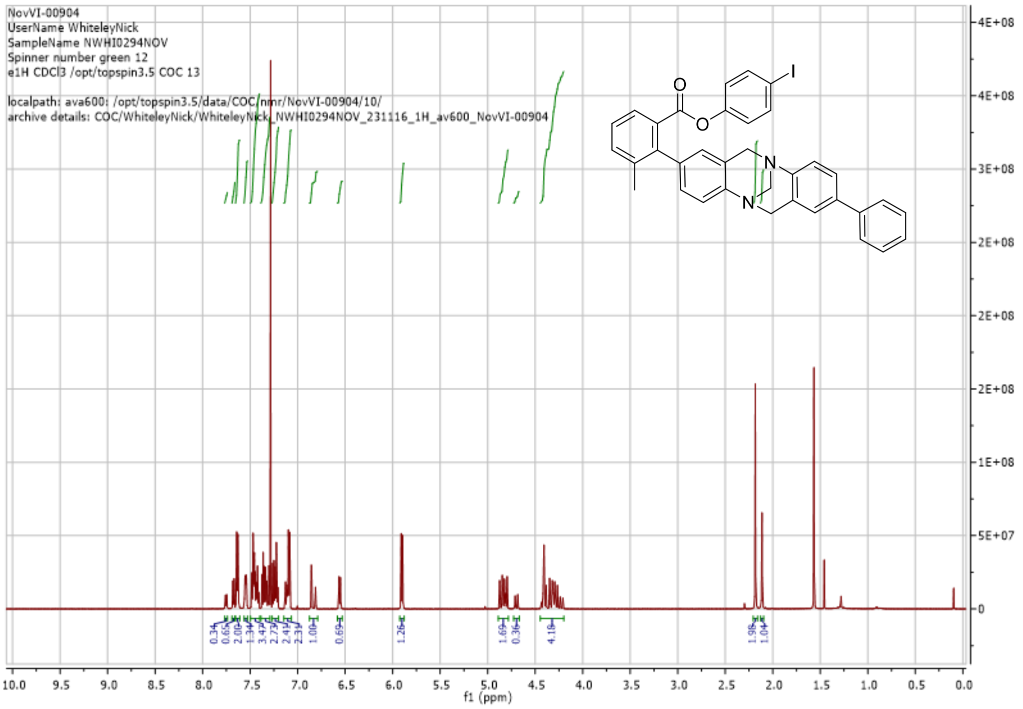

^{13}C NMR

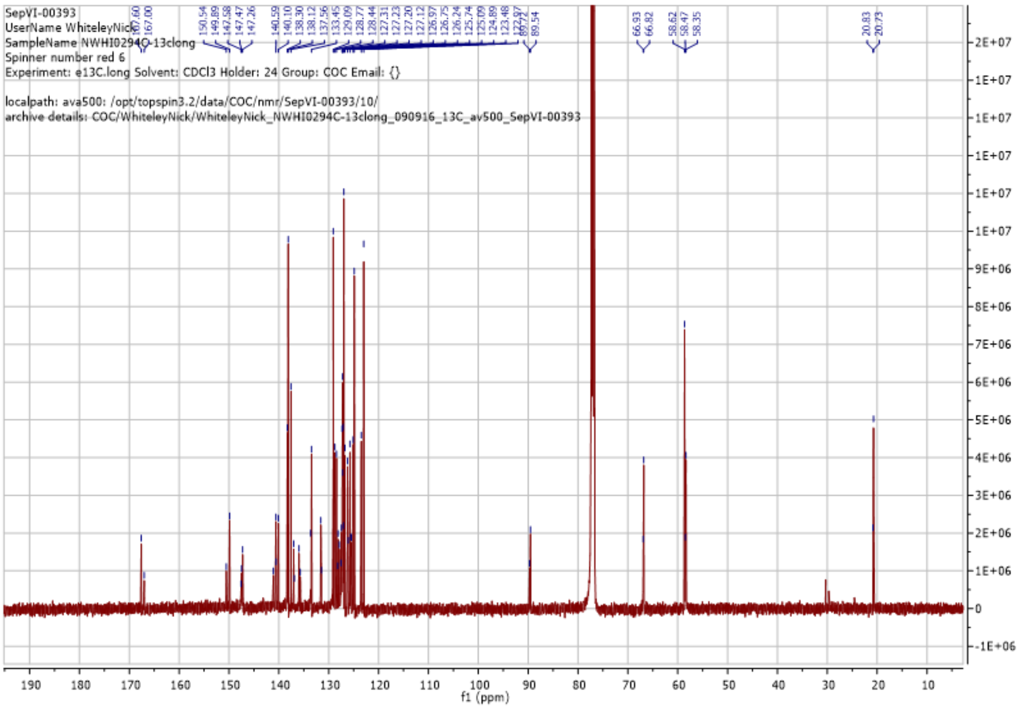

Step VIII (1H). Phenyl 3-methyl-2-[8-(phenyl)-6*H*,12*H*-5,11-methano dibenzo[*b,f*][1,5]diazocin-2-yl]benzoate

3 Methyl-2-[8-(phenyl)-6*H*,12*H*-5,11-methano dibenzo[*b,f*][1,5]diazocin-2-yl]benzoic acid (1 eq, 0.430 mmol, 0.19 g), phenol (3 eq, 1.29 mmol, 0.12 g) and PyBOP (2 eq, 1.29 mmol, 0.12 g) were combined in DCM (10 mL). Triethylamine (8 eq, 3.44 mmol, 0.35 g, 0.480 mL) was added and the stirred at room temperature for 72 h. Further DCM was added (50 mL) and the solution washed with saturated aqueous sodium bicarbonate 40 mL). Organics were dried over magnesium sulfate and solvents removed under reduced pressure. The residue was purified by flash chromatography (20-40% ether in hexane) to yield phenyl 3-methyl-2-[8-(phenyl)-6*H*,12*H*-5,11-methano dibenzo[*b,f*][1,5]diazocin-2-yl]benzoate (0.12 g, 55%).

^{1}H NMR (500 MHz, $CDCl_3$) δ 7.75 (d, J = 7.6 Hz, 0.4H, minor conformer), 7.66 (d, J = 7.6 Hz, 0.7H, major conformer), 7.56 – 7.50 (m, 2H), 7.48 – 7.37 (m, 4H), 7.37 – 7.28 (m, 2.6H), 7.24 – 7.15 (m, 3.3H), 7.15 – 7.08 (m, 1H), 6.85 (s, 0.6H, major conformer), 6.82 (s, 0.4H, minor conformer), 6.77 (app d, J = 7.9 Hz, 0.7H, minor conformer), 6.75 – 6.70 (m, 1.3H, major conformer), 6.69 – 6.65 (m, 0.6H, minor conformer), 6.11 (d, J = 7.9 Hz, 1.2H, major conformer), 4.86 – 4.75 (m, 1.6H), 4.69 (app d, J = 16.8 Hz, 0.4H, minor conformer), 4.43 – 4.17 (m, 4H), 2.16 (s, 1.9H, major conformer), 2.10 (s, 1.1H, minor conformer).

^{13}C NMR (126 MHz, $CDCl_3$) δ 168.2, 167.6, 150.9, 150.2, 147.8, 147.7, 147.4, 147.4, 141.1, 140.8, 140.7, 137.6, 137.3, 137.0, 136.2, 135.9, 133.5, 133.3, 132.2, 132.1, 129.4, 129.2, 128.9, 128.6, 128.4, 128.3, 128.1, 127.7, 127.4, 127.3, 127.2, 127.2, 127.1, 127.0, 126.9, 126.5, 126.2, 125.9, 125.8, 125.6, 125.5, 125.0, 121.4, 120.8, 67.0, 67.0, 58.8, 58.7, 58.7, 58.5, 21.0, 20.8. Two conformers, some peaks occluded.

HRMS (ESI) obtained m/z 509.2185 ($M+H^+$). Expected 509.2224. Significant dimer signal also observed in mass spec m/z 1017.4278.

^{1}H NMR

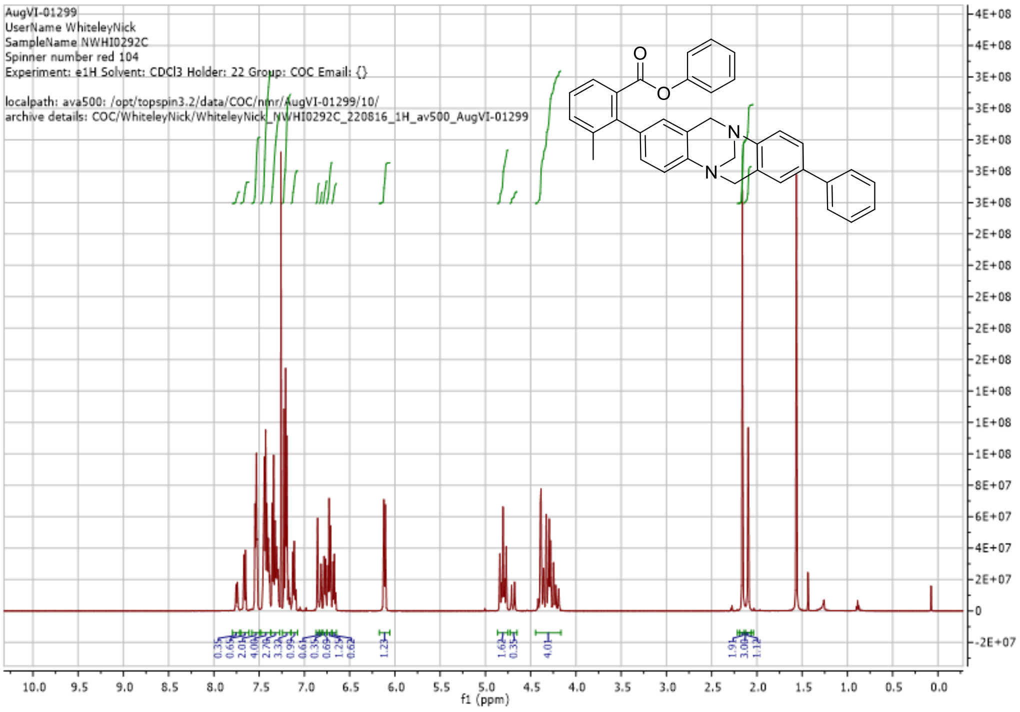

^{13}C NMR

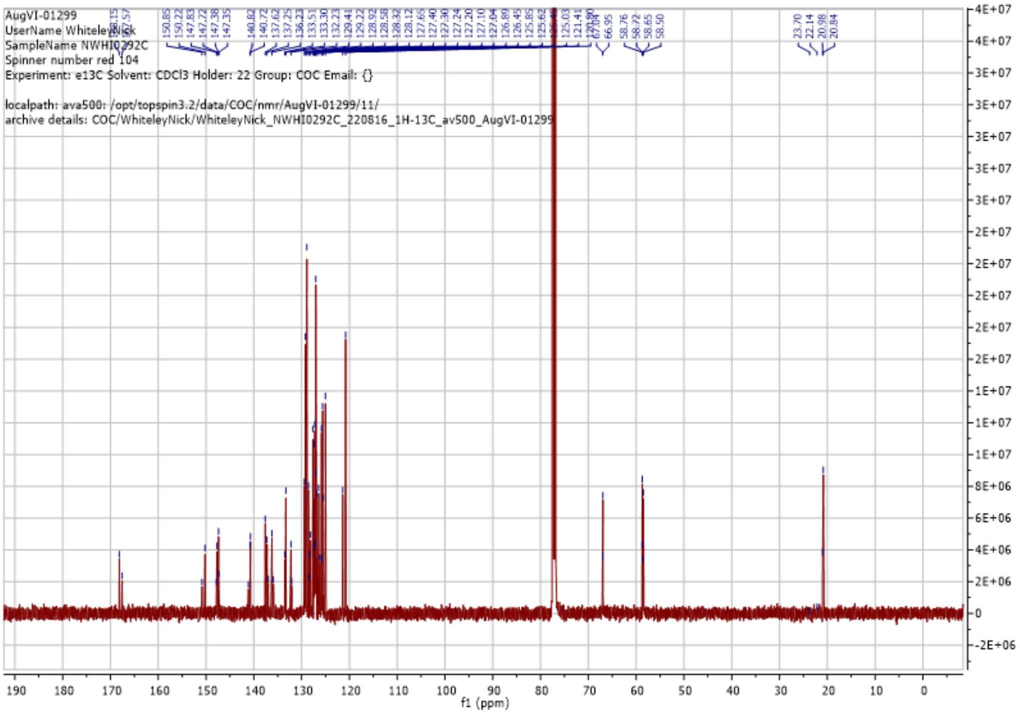

Step VIII (1Me) 4-methylphenyl 3-methyl-2-[8-(phenyl)-6*H*,12*H*-5,11-methano dibenzo[*b,f*][1,5]diazocin-2-yl]benzoate

3 methyl-2-[8-(phenyl)-6*H*,12*H*-5,11-methano dibenzo[*b,f*][1,5]diazocin-2-yl]benzoic acid (1 Eq, 0.417 mmol, 0.18 g), 4-methylphenol (3 eq, 1.24 mmol, 0.14 g) and PyBOP (2 eq, 0.832 mmol, 0.43 g) were combined in DCM (10 mL). Triethylamine (8 eq, 3.32 mmol, 0.34 g, 0.465 mL) was added and the reaction stirred at room temperature for 96 h. Further DCM was added (50 mL) and the solution washed with saturated aqueous sodium carbonate (30 mL), bicarbonate (30 mL) and brine (30 mL). Organics were dried over magnesium sulfate and solvents removed under reduced pressure. The residue was purified by flash chromatography (20-40% ether in hexane) to yield 4-methylphenyl 3-methyl-2-[8-(phenyl)-6*H*,12*H*-5,11-methano dibenzo[*b,f*][1,5]diazocin-2-yl]benzoate (0.14 g, 62%).

^{1}H NMR (601 MHz, $CDCl_3$) δ 7.73 (d, J = 7.5 Hz, 0.3H, minor conformer), 7.64 (d, J = 7.5 Hz, 0.7H, major conformer), 7.58 (app. d, J = 7.3 Hz, 1.4H), 7.53 (app. d, J = 7.5 Hz, 0.6H), 7.49 (dd, J = 8.3, 1.9 Hz, 0.7H), 7.45 – 7.38 (m, 3.3H), 7.35 – 7.28 (m, 2H), 7.25 – 7.18 (m, 2.3H), 7.27 (app. s, obscured by solvent peak, 0.7H) 7.13 – 7.08 (m, 1.6H), 6.84 (d, J = 1.1 Hz, 0.7H, major conformer), 6.81 (app. s, 0.3H, minor conformer), 6.64 (d, J = 8.3 Hz, 0.6H, minor conformer), 6.52 (d, J = 8.2 Hz, 1.3H, major conformer), 5.95 (d, J = 8.4 Hz, 1.3H, major conformer), 4.86 – 4.76 (m, 1.7H), 4.69 (d, J = 16.7 Hz, 0.3H, minor conformer), 4.43 – 4.18 (m, 4H), 2.31 (s, 1H, minor conformer), 2.16 (s, 2H, major conformer), 2.09 (s, 1H, minor conformer), 1.72 (s, 2H, major conformer).

^{13}C NMR (126 MHz, $CDCl_3$) δ 168.4, 167.8, 148.6, 147.9, 147.8, 147.8, 147.3, 141.1, 140.7, 140.6, 140.5, 137.5, 137.0, 136.9, 136.3, 135.9, 135.9, 135.5, 135.2, 133.4, 133.2, 132.4, 132.3, 129.9, 129.7, 129.0, 128.9, 128.7, 128.4, 128.4, 128.3, 128.2, 127.6, 127.4, 127.4, 127.4, 127.3, 127.2, 127.2, 127.1, 127.0, 126.9, 126.3, 126.2, 125.9, 125.8, 125.6, 125.4, 125.0, 121.1, 120.5, 67.1, 67.0, 58.8, 58.7, 58.6, 30.5, 21.0, 20.8, 20.3. Two conformers, one aromatic and one Tröger's base bridgehead conformer peaks occluded.

HRMS (ESI) obtained m/z 523.2356 ($M+H^+$). Expected 523.2380.

^{1}H NMR

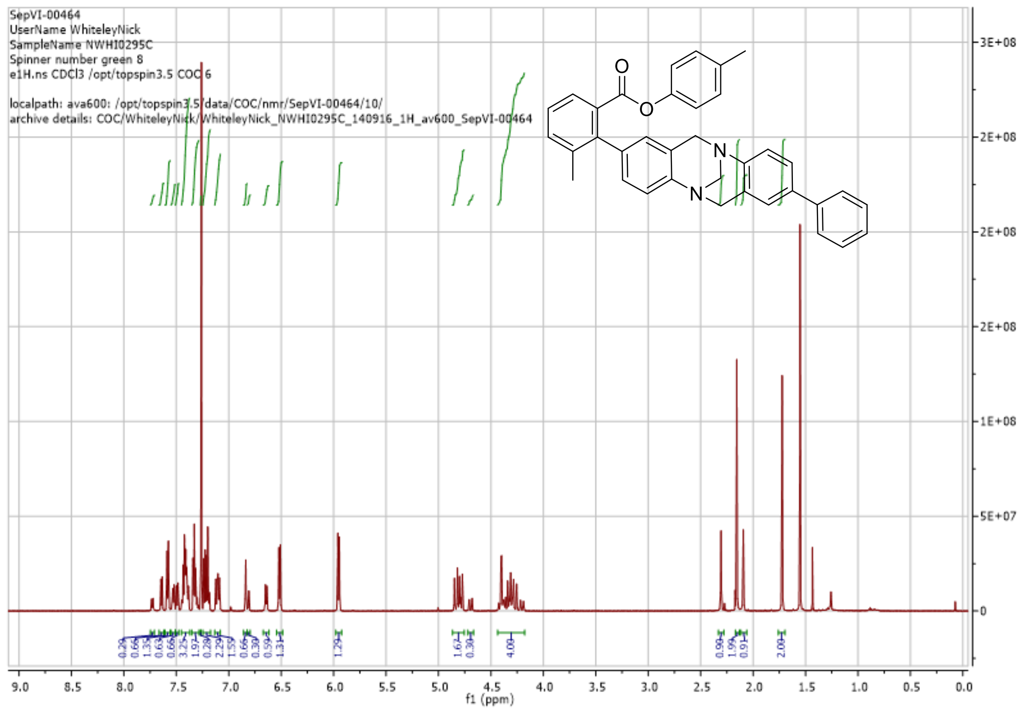

^{13}C NMR

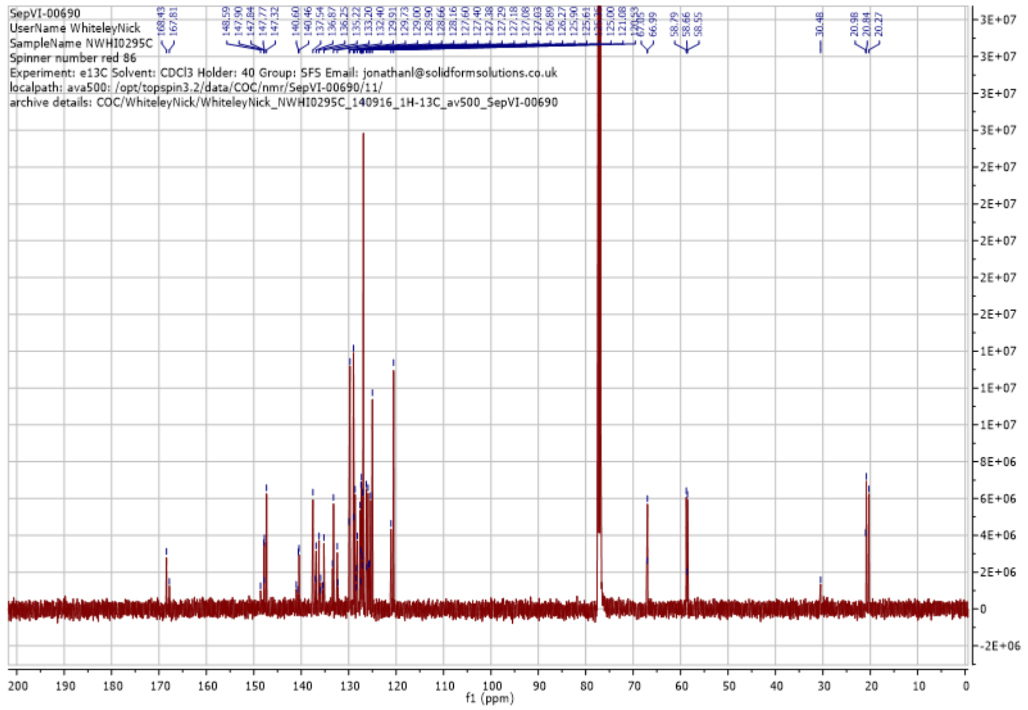

S3.2 Synthesis and characterisation of balance series (±)-2X

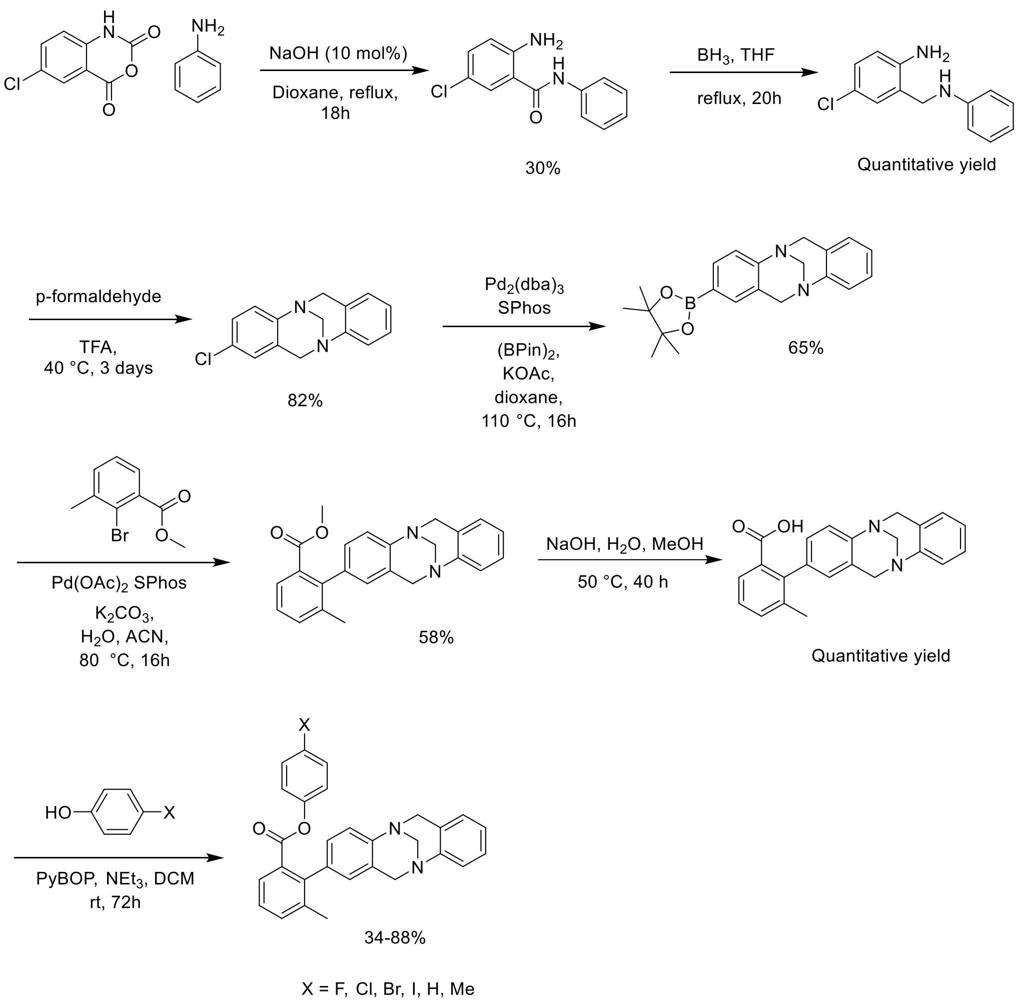

Step I. 2-Amino-5-chlorobenzanilide

5-Chloroisatoic anhydride (1.1 eq, 15.00 g), aniline (1 eq, 6.23 mL) and sodium hydroxide (0.1 eq, 0.30 g) were dissolved in dioxane (anhydrous grade, 150 mL) under an inert nitrogen atmosphere. The mixture was then heated to reflux and refluxed for 48 hours. Reaction mixture was cooled to room temperature, filtered and concentrated under reduced pressure before being diluted with ethyl acetate (50 mL) and washed with water (2 x 50 mL). Organics were dried with magnesium sulfate before solvent was removed under reduced pressure. The residue was then trituration from ethanol and dried under vacuum to yield 2-amino-5-chlorobenzanilide (6.33 g, 30%).

^{1}H NMR (500 MHz, $CDCl_3$) δ 7.71 (s, 1H), δ 7.59 - 7.57 (dd, 2H), δ 7.47 - 7.46 (d, J = 2.4 Hz, 1H), δ 7.42 – 7.38 (m, 2H), δ 7.24 – 7.22 (dd, J = 8.8, 2.4 Hz, 1H), δ 7.21 – 7.17 (m, 1H), δ 6.70 – 6.68 (d, J = 8.8 Hz, 1H), δ 5.50 (s, 2H).

^{13}C NMR (126 MHz, DMSO-d_6) δ 167.0, 149.1, 139.5, 132.3, 129.0, 128.4, 124.1, 121.1, 118.5, 118.3, 116.6.

HRMS (ESI) obtained m/z 269.0452, 271.0426 (M+Na+). Expected 269.0452, 271.0423.

^{1}H NMR

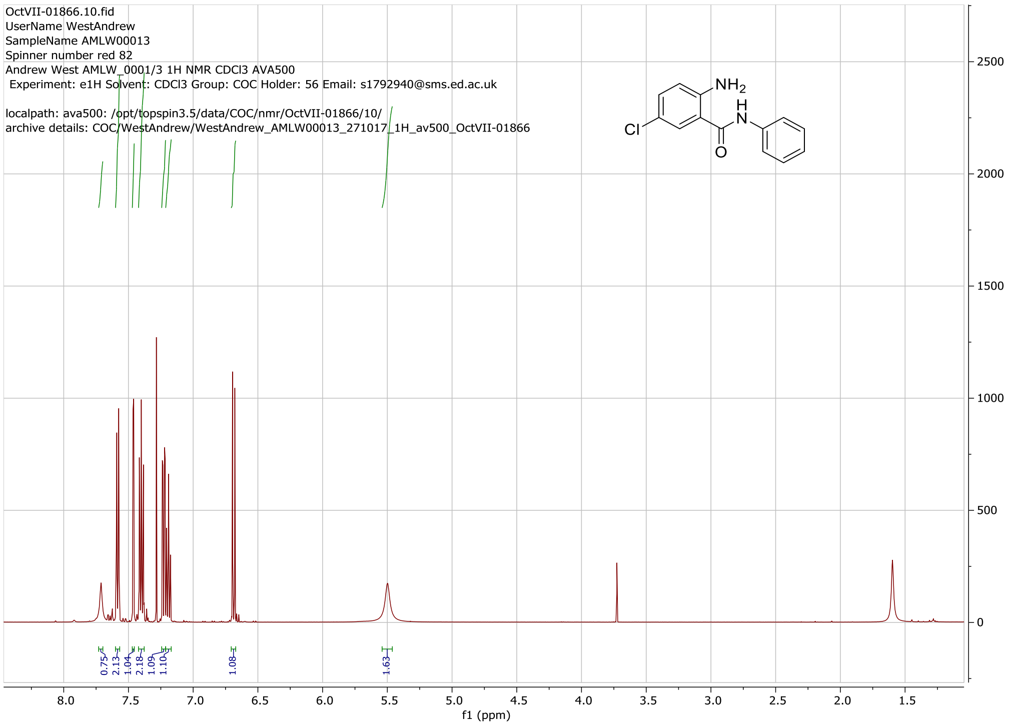

^{13}C NMR

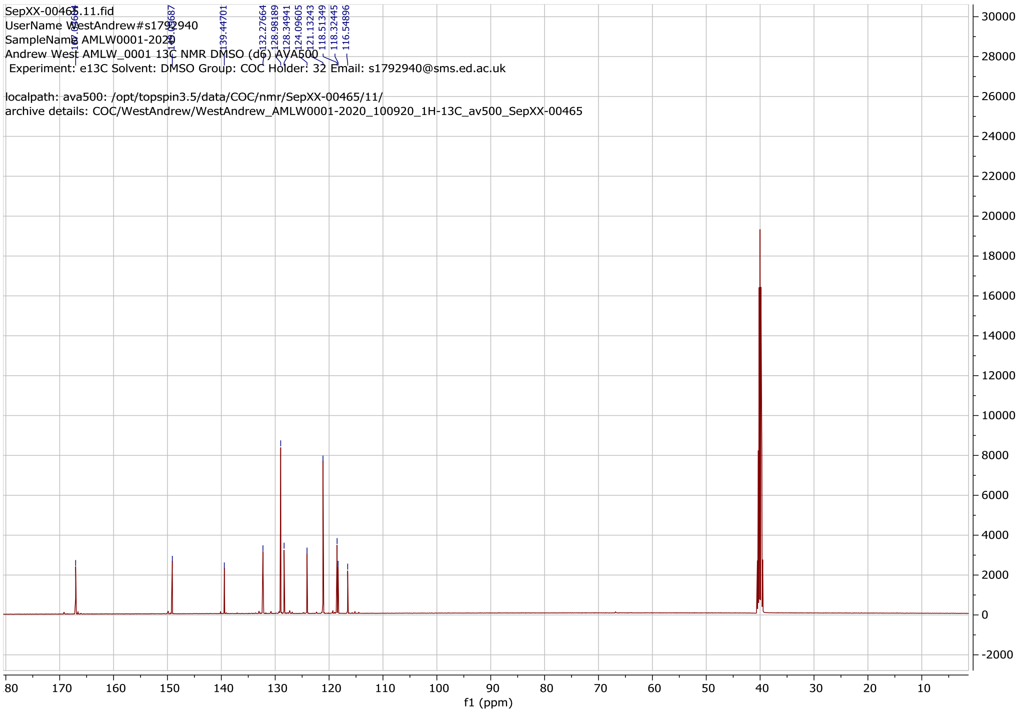

Step II-III. 2-chloro-6*H*,12*H*-5,11-Methanodibenzo[*b*,*f*][1,5]diazocine

2-Amino-5-chlorobenzanilide (1 eq, 6.00 g) was dissolved in THF (38 mL) at 0 °C under an inert nitrogen atmosphere. BH_3.THF (5.8 eq, 1.0 M in THF, 142 mL), was added slowly and stirred at 0 °C for 30 minutes. The reaction mixture was then slowly heated to reflux and refluxed for 18 hours. The reaction mixture was then slowly added to a 1.0 M aqueous sodium hydroxide (200 mL) at 0 °C. The reaction mixture was then extracted with ethyl acetate (3 x 150 mL) before organics were washed with brine (150 mL) and dried over magnesium sulfate. Solvent was then removed under reduced pressure to yield crude (~95% pure) 2-Amino-5-chloro-*N*-phenyl-benzenemethanamine in quantitative yields (5.25 g,).

2-Amino-5-chloro-*N*-phenyl-benzenemethanamine (1 eq, 5.00 g) and p-formaldehyde (6 eq, 3.91 g) were dissolved in TFA (500 mL) under an inert nitrogen atmosphere. The resultant mixture was then stirred at room temperature for 30 minutes before being heated to 40 °C and stirred for 72 hours. The reaction mixture was then added dropwise to a 0 °C 35% aqueous ammonia solution (700 mL) before extraction with DCM (3 x 300 mL). Combined organics dried over magnesium sulfate and solvents removed under reduced pressure. Resultant residue was purified by flash chromatography (10-40% ethyl acetate in petroleum ether) to yield 2-chloro-6*H*,12*H*-5,11-methanodibenzo[*b,f*][1,5]diazocine (4.59 g, 81.8%).

^{1}H NMR (500 MHz, $CDCl_3$) δ 7.20 – 7.15 (m, 1H), δ 7.13 -7.10 (m, 2H), δ 7.08 – 7.05 (d, J = 8.6 Hz, 1H), δ 7.02 – 6.97 (td, J = 7.4, 1.4 Hz, 1H), δ 6.92 – 6.89 (m, 2H), δ 4.73 – 4.62 (app dd, J = 18.0, 2.7 Hz, 2H), δ 4.36 – 4.24 (app m, 2H), δ 4.19 – 4.08 (app t J = 15.5 Hz, 2H).

^{13}C NMR (126 MHz, $CDCl_3$) δ 147.8, 146.7, 129.6, 128.9, 127.6, 127.5, 127.0, 126.8, 126.4, 125.1, 124.2, 66.8, 58.8, 58.5.

HRMS (ESI) obtained m/z 257.0840, 259.0814 ($M+H^+$). Expected 257.0840, 259.0811.

^{1}H NMR

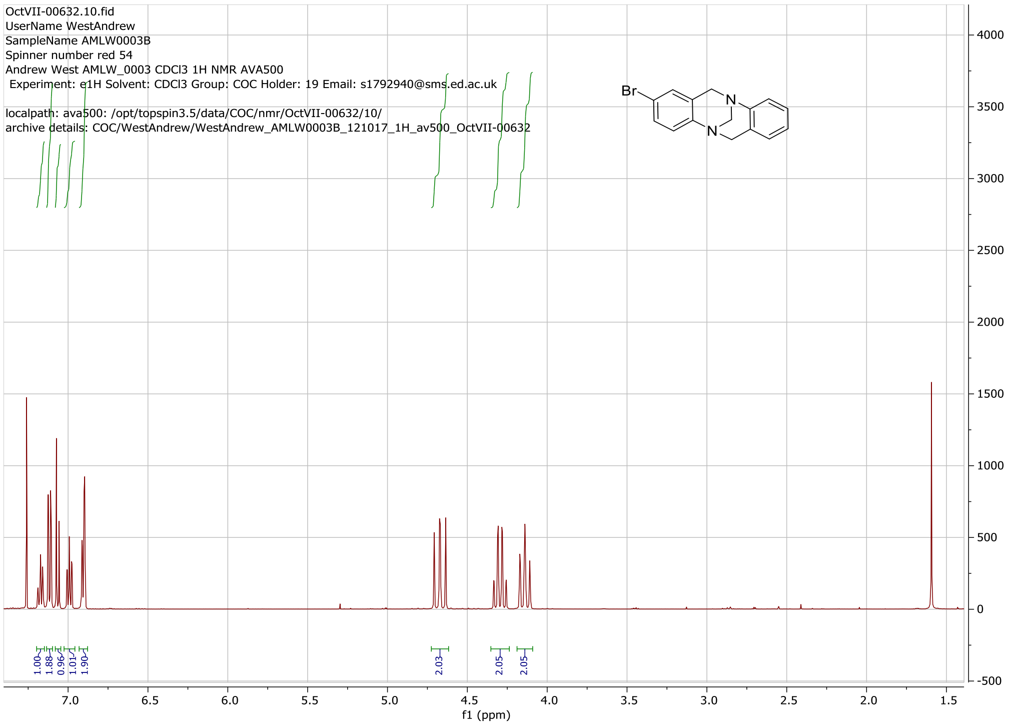

^{13}C NMR

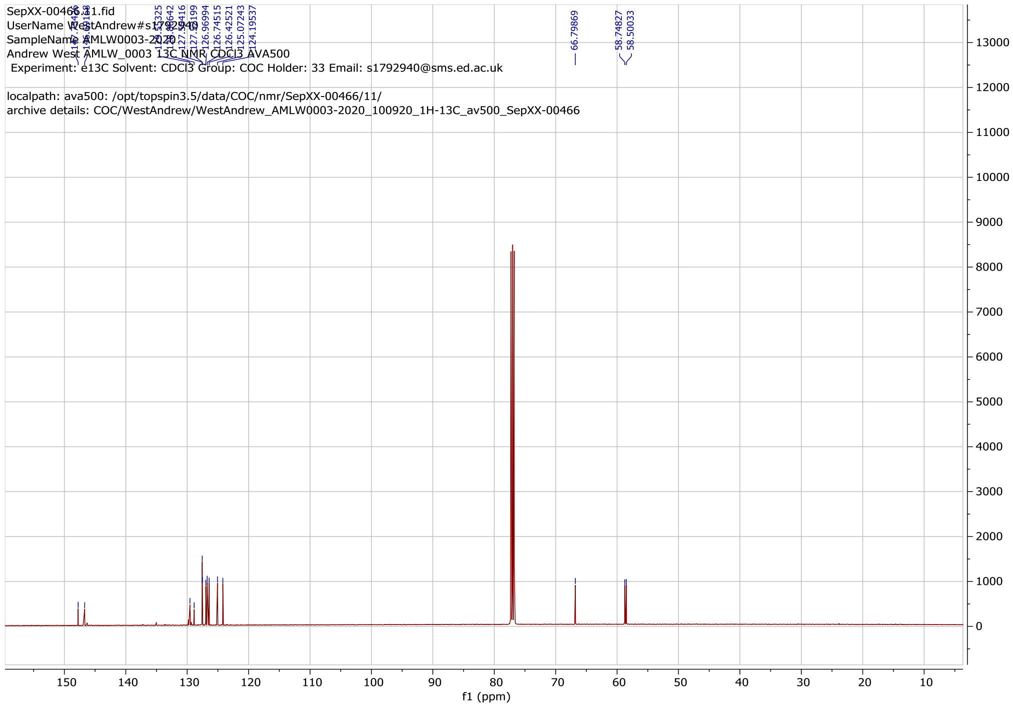

Step IV. 2-(4,4,5,5-Tetramethyl-1,3,2-dioxaborolan-2-yl)-6*H*,12*H*-5,11-methanodibenzo[*b,f*][1,5]diazocine

2-chloro-6*H*,12*H*-5,11-methanodibenzo[*b,f*][1,5]diazocine (1 eq, 4.50 g), bis(pinacolato)diboron (3 eq, 13.35 g), tris(dibenzylideneacetone)dipalladium (0.02 eq, 0.32 g), SPhos (0.04 eq, 0.29 g) and $Pd(OAc)_2$ (3 eq, 5.16 g) were dissolved in degassed dioxane (anhydrous grade, 14 mL). The reaction mixture was then heated to reflux and refluxed for 16 hours. The reaction mixture was then cooled and filtered through kieselguhr with ethyl acetate. Organics were washed with saturated sodium bicarbonate solution (150 mL), brine (50 mL) and dried over magnesium sulfate before the solvent was removed under vacuum and purified via flash chromatography (40% ethyl acetate in petroleum ether) to yield 2-(4,4,5,5-tetramethyl-1,3,2-dioxaborolan-2-yl)-6*H*,12*H*-5,11-methanodibenzo[*b,f*][1,5]diazocine (3.94 g, 65%).

^{1}H NMR (500 MHz, $CDCl_3$) δ 7.62 – 7.57 (d, J = 8.0 Hz, 1H), δ 7.39 (s, 1H), δ 7.17 – 7.08 (m, 3H), δ 6.98 – 6.91 (td, J = 1.6, 7.3, 6.9 Hz, 1H), δ 6.90 – 6.85 (d, J = 7.3 Hz, 1H), δ 4.74 – 4.66 (app dd, J = 6.6, 16.6 Hz, 2H), δ 4.35 – 4.31 (app s, 2H), δ 4.24 – 4.17 (app d, J = 16.6, 2H), δ 1.29 (s, 12H).

^{13}C NMR (126 MHz, $CDCl_3$) δ 151.2, 148.0, 133.9, 133.7, 127.8, 127.4, 127.2, 126.9, 83.7, 66.8, 58.8, 58.6, 24.8, 24.8.

HRMS (ESI) obtained m/z 349.2064 (M+H^{+}). Expected 349.2082.

^{1}H NMR

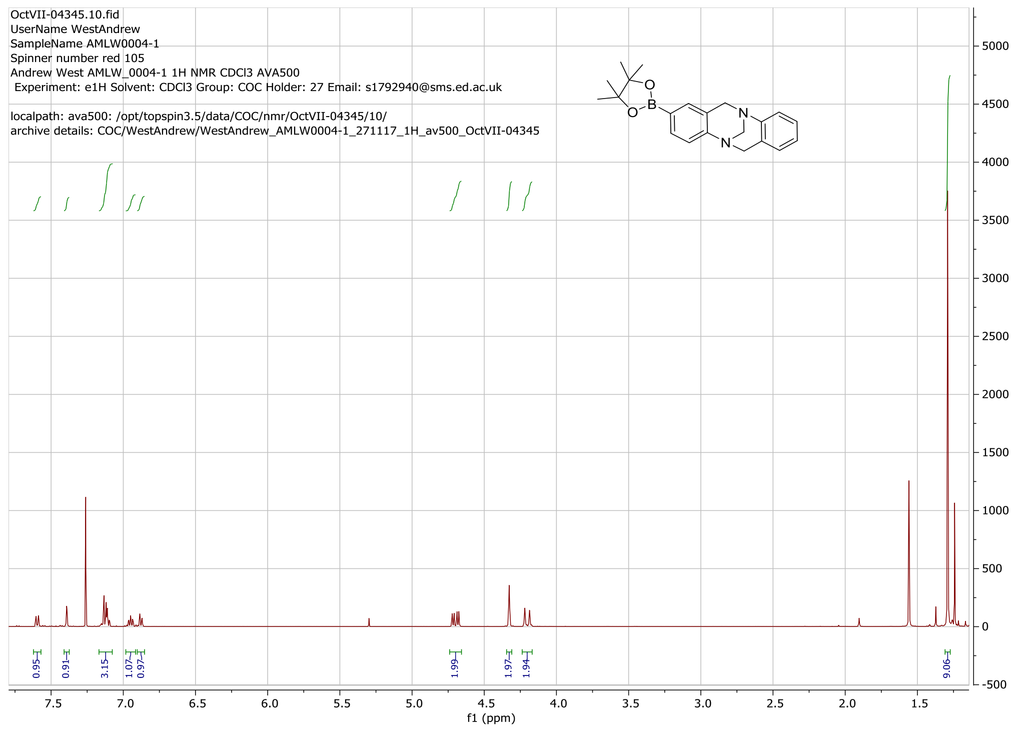

^{13}C NMR

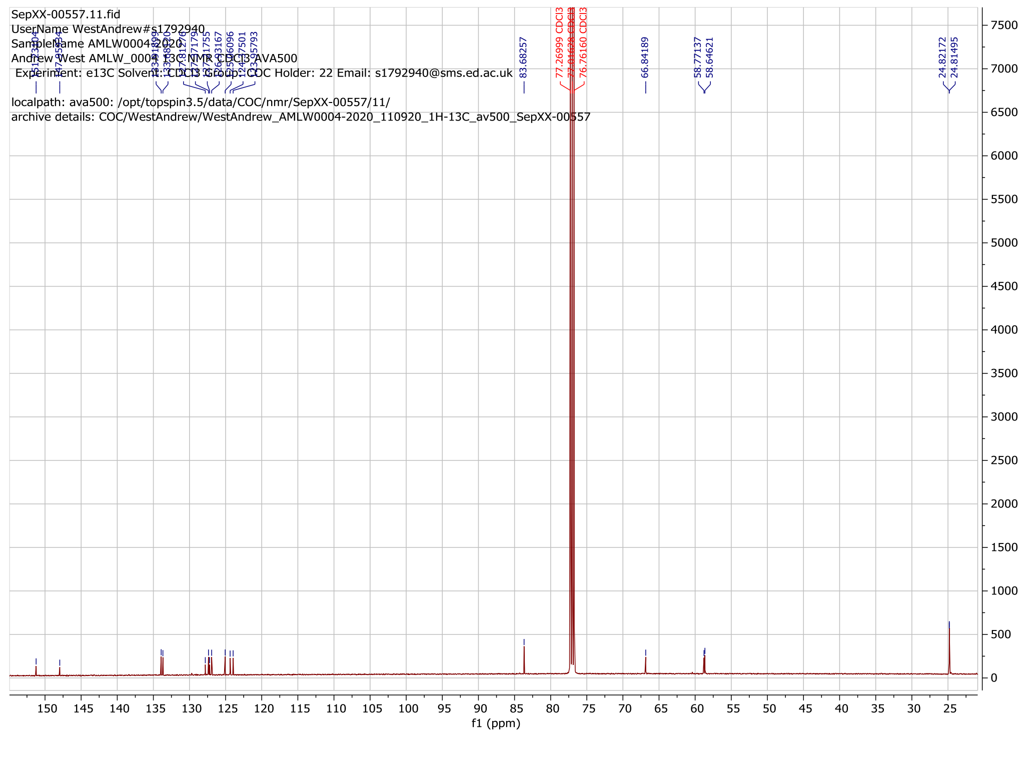

Step V. Benzoic acid, 2-(6*H*,12*H*-5,11-methanodibenzo[*b,f*][1,5]diazocin-2-yl)-3-methyl-, methyl ester

2-(4,4,5,5-tetramethyl-1,3,2-dioxaborolan-2-yl)-6*H*,12*H*-5,11-methanodibenzo[*b,f*][1,5]diazocine (1 eq, 2.00 g) palladium(II)acetate (0.02 eq, 0.026 g), SPhos (0.04 eq, 0.094 g) and methyl 2-bromo-3methyl-benzoate (1.5 eq, 1.97 g) were dissolved in acetonitrile. To the resultant mixture, 2 M potassium carbonate solution (2.5 eq, 7.2 mL) was added before the reaction mixture was heated to reflux under a nitrogen atmosphere and allowed to react for 16 hours. The reaction mixture was then diluted with ethyl acetate (250 mL) and washed with sodium bicarbonate solution (2 x 150 mL) and brine (100 mL) before the organics were dried with magnesium sulfate before solvent was removed under reduced pressure. The crude reaction mixture was then purified by flash chromatography (10-14% ethyl acetate in petroleum ether) to yield benzoic acid, 2-(6H,12H-5,11-methanodibenzo[*b,f*][1,5]diazocin-2-yl)-3-methyl-,methyl ester (1.25 g, 58%).

^{1}H NMR (500 MHz, $CDCl_3$) δ 7.62 – 7.49 (dd, J = 7.7, 33.8 Hz, 1H), δ 7.39 – 7.30 (dd, J = 7.8, 13.97 Hz, 1H) δ 7.29 – 7.23 (m, 1H), δ 7.22 – 7.10 (m, 3H), δ 7.05 – 6.92 (m, 2H), 6.92 – 9.87 (d, J = 7.6 Hz, 1H), 6.73 – 6.63 (m, 1H), δ 4.79 – 4.68 (app m, 2H), δ 4.50 – 4.30 (app m, 2H), δ 4.21 – 4.08 (app m, 2H), δ 3.58 (s, 1.2H, minor conformer), δ 2.78 (s, 1.8H, major conformer), δ 2.18 (s, 1.8H, major conformer), δ 2.02 (s, 1.2H, minor conformer).

13C NMR (126 MHz, $CDCl_3$) δ 169.7, 169.1, 143.3, 147.9, 146.9, 146.7, 136.8, 135.9, 132.9, 132.8, 128.1, 127.9, 127.4, 127.3, 127.0, 126.9, 126.8, 126.7, 125.3, 124.9, 124.6, 124.2, 123.8, 67.4, 67.0, 59.3, 59.2, 58.6, 51.9, 50.9, 20.8, 20.7.

HRMS (ESI) obtained m/z 393.1572 (M+Na^{+}). Expected 393.1573.

^{1}H NMR

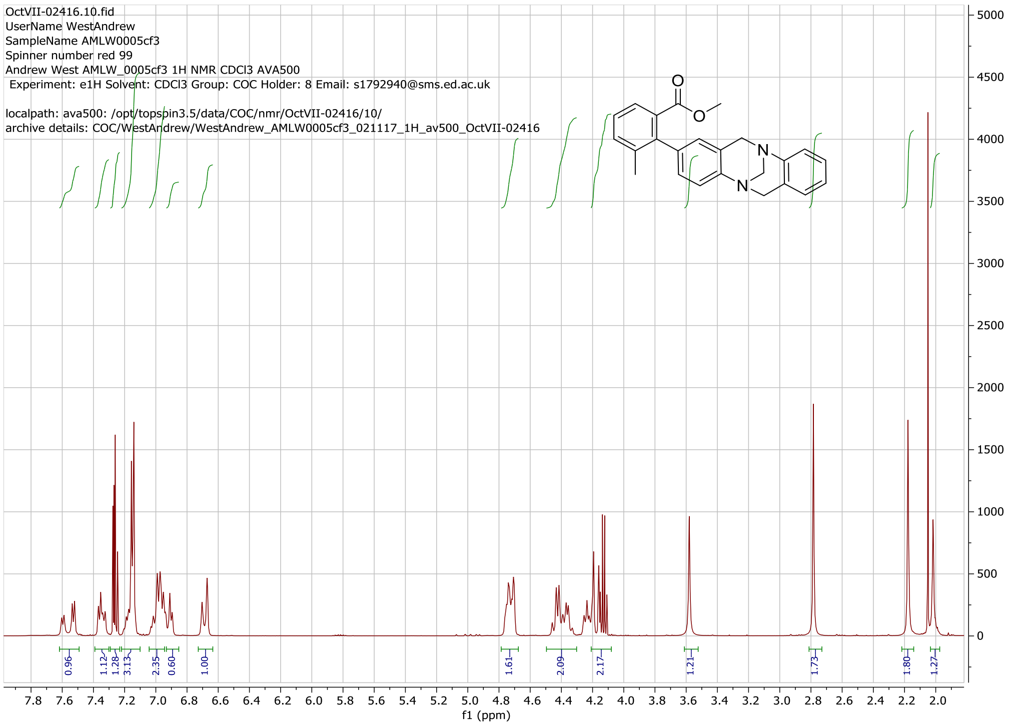

^{13}C NMR

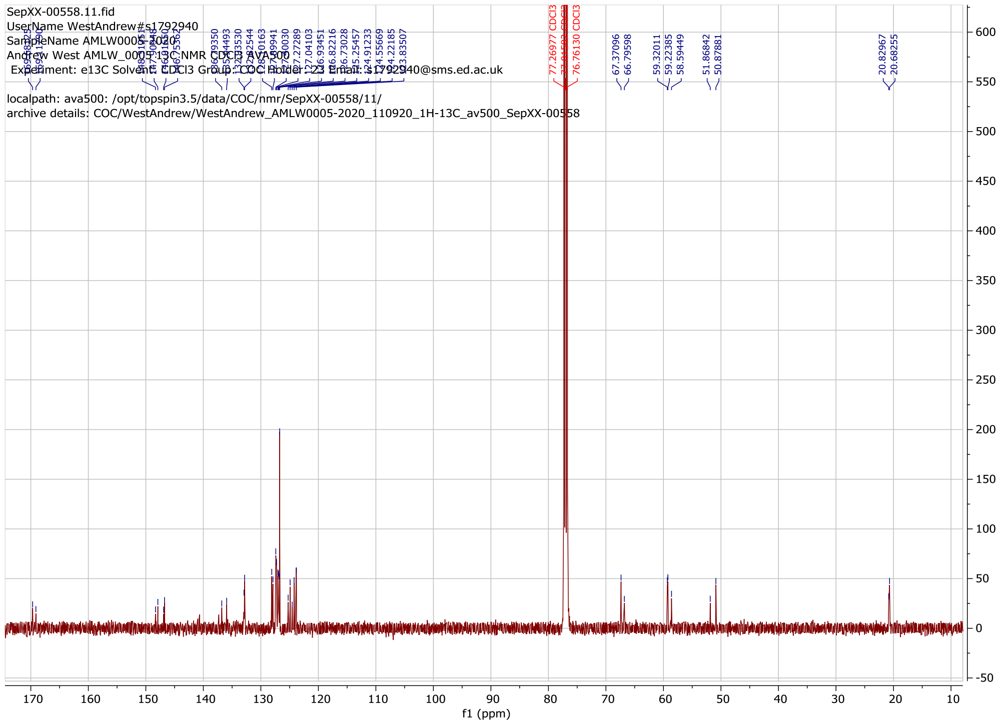

Step VI. 2-(6*H*,12*H*-5,11-methanodibenzo[*b,f*][1,5]diazocin-2-yl)-3-methyl-benzoic acid.

Benzoic acid, 2-(6*H*,12*H*-5,11-methanodibenzo[*b,f*][1,5]diazocin-2-yl)-3-methyl-, methyl ester (1 eq, 0.57 g) and sodium hydroxide (17 eq, 1.1 g) were dissolved in a solution of methanol (44 mL), tetrahydrofuran (44 mL) and water (0.4 mL) before being heated to 55 °C for 40 hours. The reaction mixture was cooled to room temperature before 1 M hydrochloride acid (1.67 mL) was added dropwise. Phases were separated and the solvent removed under reduced pressure to yield 2-(6*H*,12*H*-5,11-methanodibenzo[*b,f*][1,5]diazocin-2-yl)-3-methyl-benzoic acid (0.55 g) in quantitative yield.

^{1}H NMR (500 MHz, DMSO-d_6) δ 12.41 (s, 1H), δ 7.48 – 7.44 (bs, 1H) δ 7.40 – 7.36 (bs, 1H), δ 7.34 – 7.27 (t, J = 7.6 Hz, 1H), δ 7.20 – 7.16 (m, 2H), δ 7.14 – 7.10 (d, J = 5.7 Hz, 1H), δ 7.04 – 6.93 (m, 3H), δ 6.79 – 6.75 (d, J = 2.02, 1H), δ 4.71 – 4.64 (app d, J = 16.8 Hz, 1H), δ 4.33 – 4.05 (app m, 4H), δ 3.66 – 3.59 (app m, 1H), δ 2.10 – 1.90 (d, J = 28.8 Hz, 3H).

^{13}C NMR (126 MHz, DMSO-d_6) δ 170.1, 148.8, 147.3, 140.0, 135.1, 132.4, 128.6, 128.2, 127.6, 127.5, 127.4, 127.3, 125.5, 124.7, 123.9, 66.5, 58.4, 20.9.

HRMS (ESI) obtained m/z 379.4139. ($M+Na^+$). Expected 379.4142.

^{1}H NMR

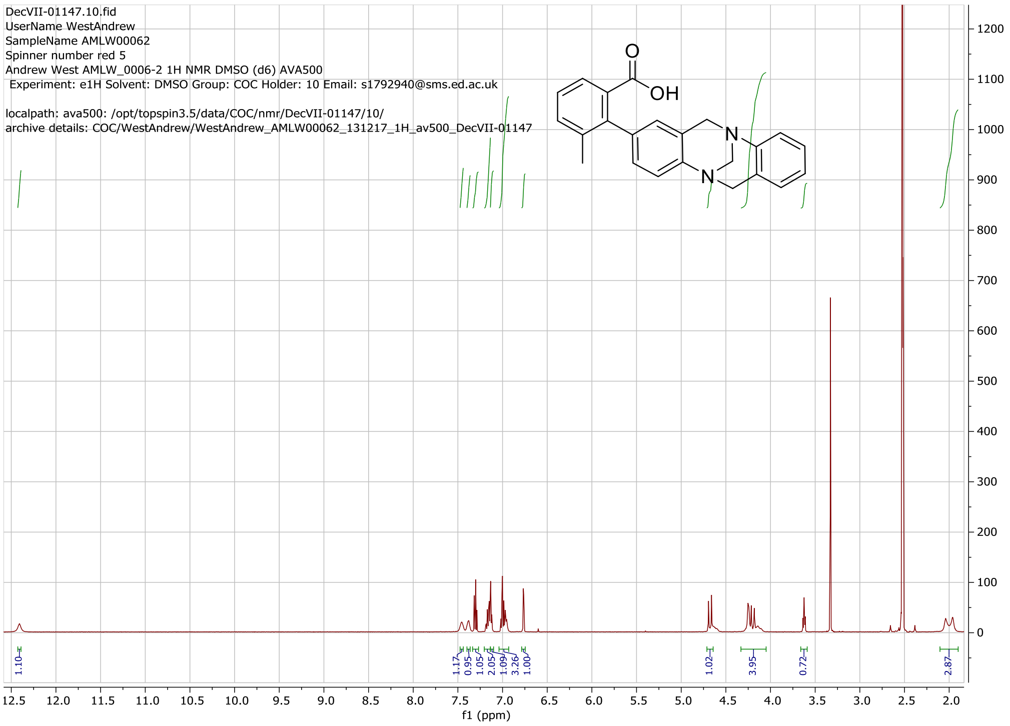

^{13}C NMR

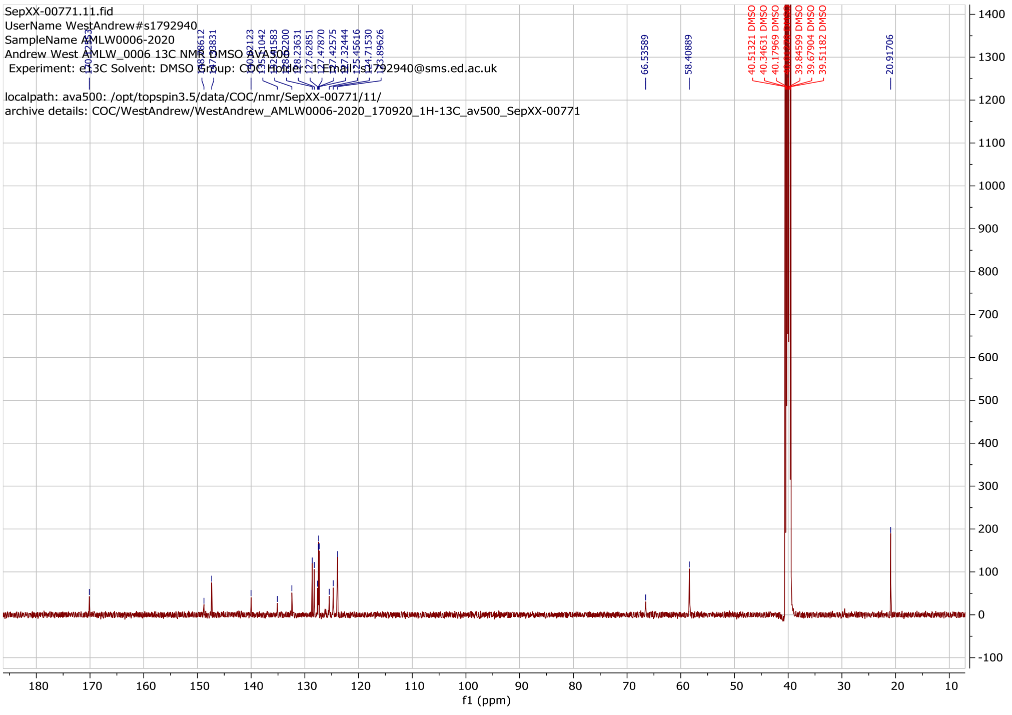

Step VII (±)-(2F)

4-Fluorophenyl 3-methyl-2-[6*H*,12*H*-5,11-methano dibenzo[*b,f*][1,5]diazocin-2-yl]benzoate

To a solution of 2-(4,4,5,5-tetramethyl-1,3,2-dioxaborolan-2-yl)-6*H*,12*H*-5,11-methanodibenzo[*b,f*][1,5]diazocine (1 eq, 0.50 g) dissolved in acetonitrile (10 mL) was added $Pd(OAc)_2$ (0.02 eq, 0.0065 g), SPhos (0.04 eq, 0.024 g) and methyl 2-bromo-3-(4-fluorophenol)-benzoate (1.5 eq, 0.66 g). 2 M potassium carbonate solution (2.5 eq, 1.44 mL) was added to the resultant solution, before being heated to reflux under an inert nitrogen atmosphere for 16 hours. The reaction mixture was then diluted with ethyl acetate (250 mL) and washed with saturated sodium bicarbonate solution (2 x 150 mL) and brine (100 mL) before organics were dried over magnesium sulfate and the solvent removed under reduced pressure. The crude reaction mixture was purified by flash chromatography (30% ethyl acetate in petroleum ether) to yield 4-fluorophenyl 3-methyl-2-[6*H*,12*H*-5,11-methano dibenzo[*b,f*][1,5]diazocin-2-yl]benzoate (0.38 g, 58%).

^{1}H NMR (500 MHz, $CDCl_3$) δ 7.75 (d, J = 7.6 Hz, 0.3H, minor conformer), 7.67 (d, J = 7.7 Hz, 0.6H major conformer), 7.44 (t, 1H), 7.36 (t, 1H), 7.27-7.07 (m, 5H), 7.07-6.96 (m, 2H), 6.84 (d, J = 1.9 Hz, 0.7H major conformer), 6.80 (d, J = 1.9 Hz, 0.3H, minor conformer), 6.77-6.70 (m, 0.6H, minor conformer), 6.56 (m, 1H), 6.11-6.05 (m, 1.3H, major conformer), 4.78 (m, J = 16.8 Hz, 1.7H, major conformer), 4.68 (m, J = 16.8 Hz, 0.3H, minor conformer), 4.43-4.33 (m, 2H), 4.31-4.15 (m, 2H), 2.18 (s, 2H, major conformer), 2.11 (s, 1H minor conformer).

^{13}C NMR (126 MHz, $CDCl_3$) δ 168.0, 167.4, 160.7, 158.8, 148.2, 147.3, 145.9, 141.0, 140.6, 137.5, 136.0, 135.7, 133.5, 133.3, 131.8, 131.6, 129.7, 128.3, 128.1, 127.7, 127.6, 127.3, 127.2, 127.0, 127.0, 125.4, 125.3, 124.9, 124.3, 123.9, 122.7, 122.6, 122.1, 122.0, 116.0, 115.9, 115.8, 115.7, 66.8, 66.7, 58.5, 58.4, 20.8, 20.7. Two conformers. Some peaks occluded.

HRMS (ESI) obtained m/z 473.1637. ($M+Na^{+}$). Expected 473.1641.

^{1}H NMR

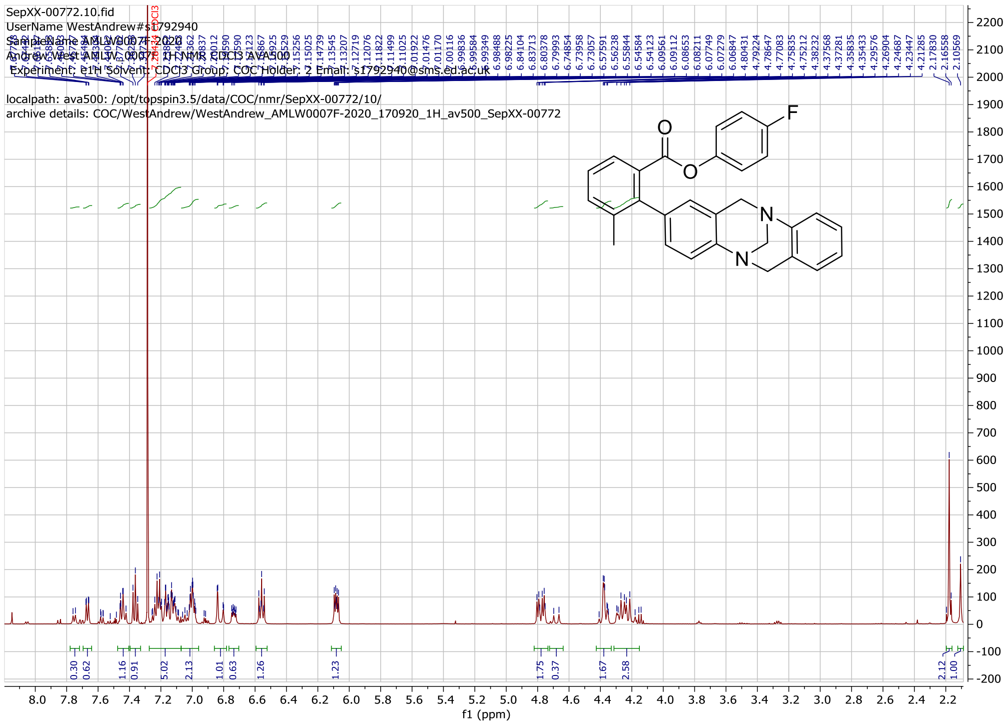

^{13}C NMR

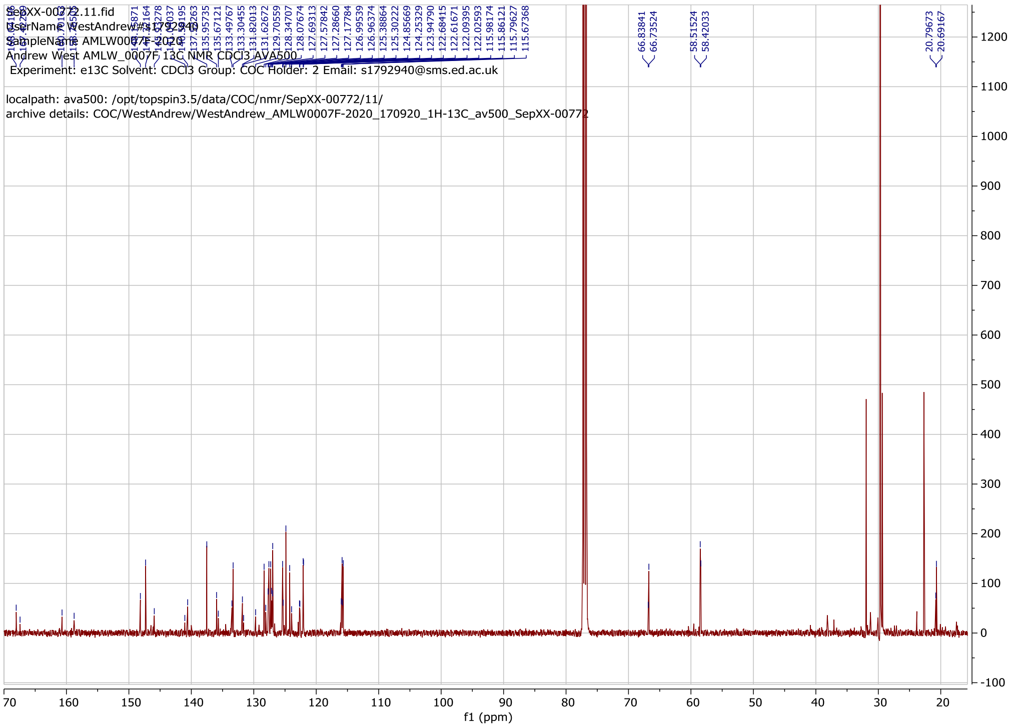

Step VII (±)-2Cl

4-Chlorophenyl 3-methyl-2-[6*H*,12*H*-5,11-methano dibenzo[*b,f*][1,5]diazocin-2-yl]benzoate

2-(6H,12H-5,11-methanodibenzo[b,f][1,5]diazocin-2-yl)-3-methyl-benzoic acid (1 Eq, 0.099 g), 4-chlorophenol (3 Eq, 0.11 g), and PyBOP (2 Eq, 0.29 g) were dissolved in DCM (5 mL). To the resultant mixture, triethylamine (8 Eq, 0.726 mL) was added before the solution was stirred at room temperature for 72 hours. Additional DCM (50 mL) was added to the reaction mixture before the mixture was washed with saturated aqueous sodium bicarbonate solution (30 mL) and brine (2 x 30 mL). Organics were then dried on magnesium sulfate before the solvent was removed under reduced pressure and the resultant residue was purified by flash chromatography (20% ethyl acetate in petroleum ether). 4-Chlorophenyl 3-methyl-2-[6H,12H-5,11-methano dibenzo[b,f][1,5]diazocin-2-yl]benzoate was isolated as a while solid (0.090 g, 67%).

^{1}H NMR (500 MHz, $CDCl_3$) δ 7.73 (d, J = 7.6 Hz, 0.3H, minor conformer), 7.64 (d, J = 7.5 Hz, 0.7H, major conformer), 7.42 (t, 1H), 7.34 (t, 1H), 7.28-7.15 (m, 4H), 7.13 (m, 2H), 7.09 (dd, 1H), 6.98 (d, 1H), 6.81-676 (m, 2H), 6.70 (d, J = 8.8 Hz, 0.5H, minor conformer) 6.05 (d, J = 8.8 Hz, 0.7H), 4.75 (dd, J = 16.8 Hz, 1.7H, major conformer), 4.65 (d, J = 16.7 Hz, 0.3H, minor conformer), 4.38-4.33 (m, 2H), 4.26-4.15 (m, 2H), 2.15 (s, 2H, major conformer), 2.08 (2, 1H, minor conformer).

^{13}C NMR (126 MHz, $CDCl_3$) δ 168.06, 167.76, 148.60, 148.11, 147.36, 136.07, 133.38, 129.42, 129.42, 129.18, 128.31, 127.60, 127.30, 127.00, 126.92, 125.36, 124.86, 124.32, 122.63, 122.00, 66.83, 66.73, 58.52, 58.41, 37.11, 36.66, Two conformers. Some peaks occluded.

HRMS (ESI) obtained m/z 489.1301, 489.1429. (M+Na+). Expected 489.1346, 489.1316.

^{1}H NMR

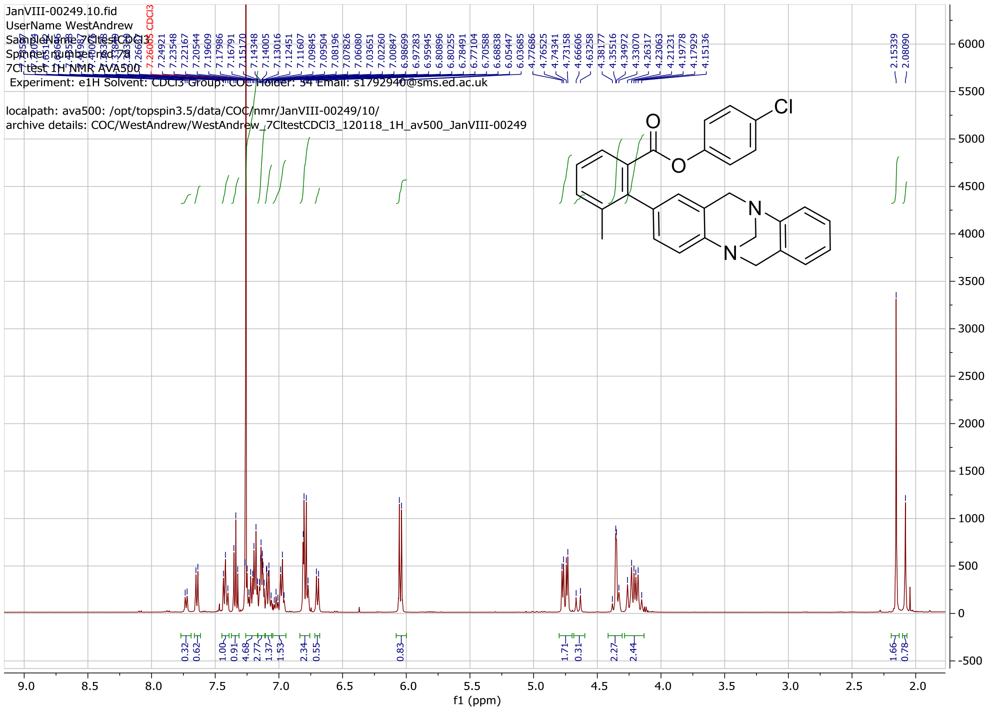

^{13}C NMR

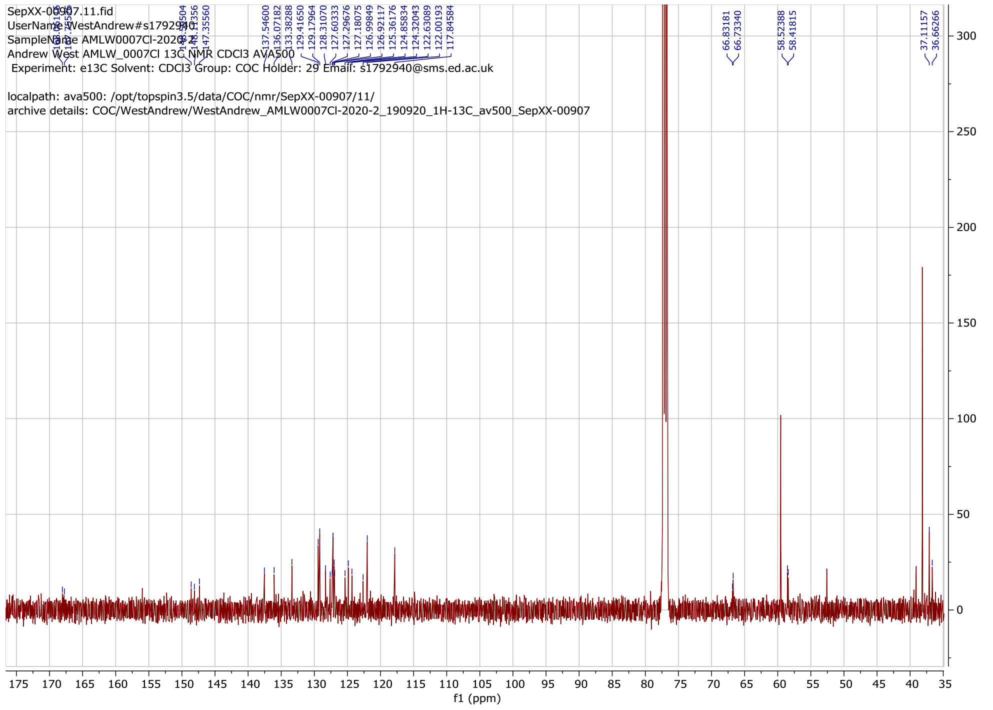

Step VII (±)-2Br

4-Brorophenyl 3-methyl-2-[6*H*,12*H*-5,11-methano dibenzo[*b*,*f*][1,5]diazocin-2-yl]benzoate

2-(6*H*,12*H*-5,11-methanodibenzo[*b,f*][1,5]diazocin-2-yl)-3-methyl-benzoic acid (1 aq, 0.087 g), 4-bromophenol (3 Eq, 0.13 g), and PyBOP (2 Eq, 0.26 g) were dissolved in DCM (5 mL). To the resultant mixture, triethylamine (8 eq, 0.73 mL) was added before the solution was stirred at room temperature for 72 hours. Additional DCM (50 mL) was added to the reaction mixture before the mixture was washed with saturated aqueous sodium bicarbonate solution (30 mL) and brine (2 x 30 mL). Organics were then dried on magnesium sulfate before the solvent was removed under reduced pressure and the resultant residue was purified by flash chromatography (20% ethyl acetate in petroleum ether). 4-Brorophenyl 3-methyl-2-[6*H*,12*H*-5,11-methano dibenzo[*b,f*][1,5]diazocin-2-yl]benzoate was isolated as a while solid (0.11 g, 86%).

^{1}H NMR (500 MHz, $CDCl_3$) δ 7.75 (d, J = 7.7 Hz, 0.3 H, minor conformer), 7.67 (d, J = 7.7 Hz, 0.7H, major conformer), 7.44 (t, 1H), 7.36 (t, 1H), 7.27-7.14 (m, 4H), 7.13-6.98 (m, 3H), 6.95 (m, 1H), 6.83 (d, J = 2.0 Hz, 0.7H, major conformer), 6.79 (d, J = 1.9 Hz, 0.3H, minor conformer), 6.67 (m, 0.6H, minor conformer), 6.01 (m, 1.3H, major conformer), 4.77 (dt, J = 16.8 Hz, 1.7H, major conformer), 4.67 (d, J = 16.8 Hz, 0.3H, minor conformer), 4.42-4.33 (m, 2H), 4.30-4.16 (m, 2H), 2.18 (s, 2H, major conformer), 2.11 (s, 1H, minor conformer).

^{13}C NMR (126 MHz, $CDCl_3$) δ 167.7, 167.1, 149.7, 149.1, 148.2, 148.1, 147.4, 141.1, 140.6, 137.5, 135.9, 135.6, 133.6, 133.4, 132.3, 132.2, 131.7, 131.5, 128.3, 128.1, 128.0, 127.7, 127.6, 127.6, 127.3, 127.2, 127.2, 127.0, 126.9, 125.4, 125.3, 124.9, 124.9, 124.4, 123.9, 123.1, 122.4, 118.8, 118.4, 66.8, 66.7, 58.5, 58.4, 20.8, 20.7. Two conformers. Some peaks occluded.

HRMS (ESI) obtained m/z 533.7021, 535.0831 ($M+Na^{+}$). Expected 533.0835, 535.0815.

^{1}H NMR

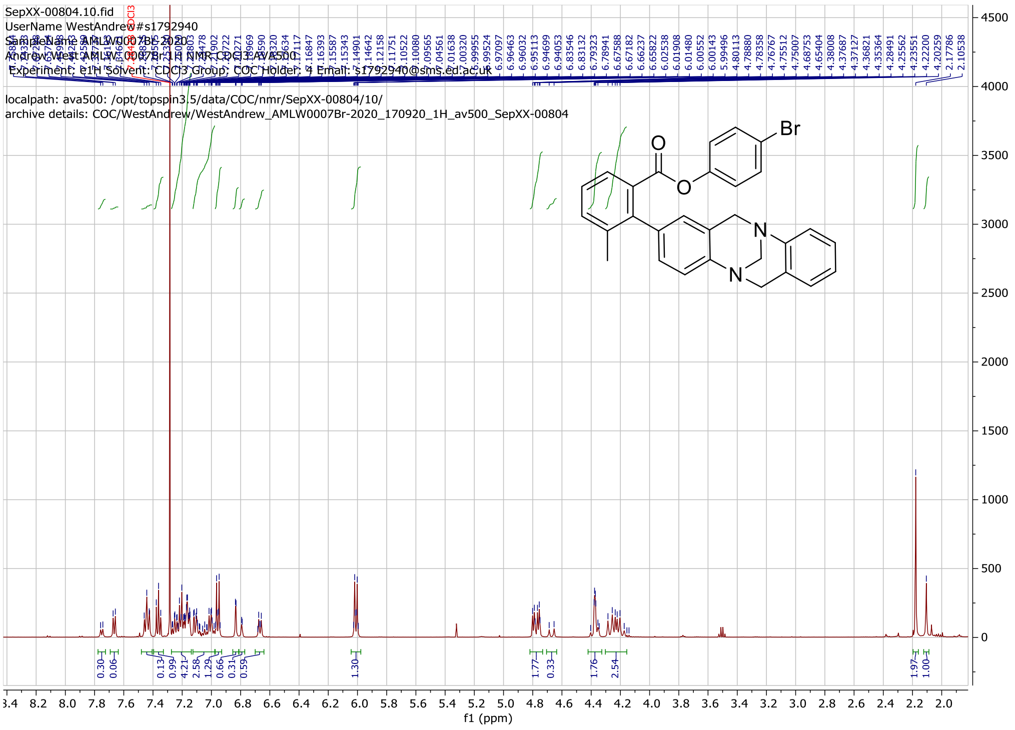

^{13}C NMR

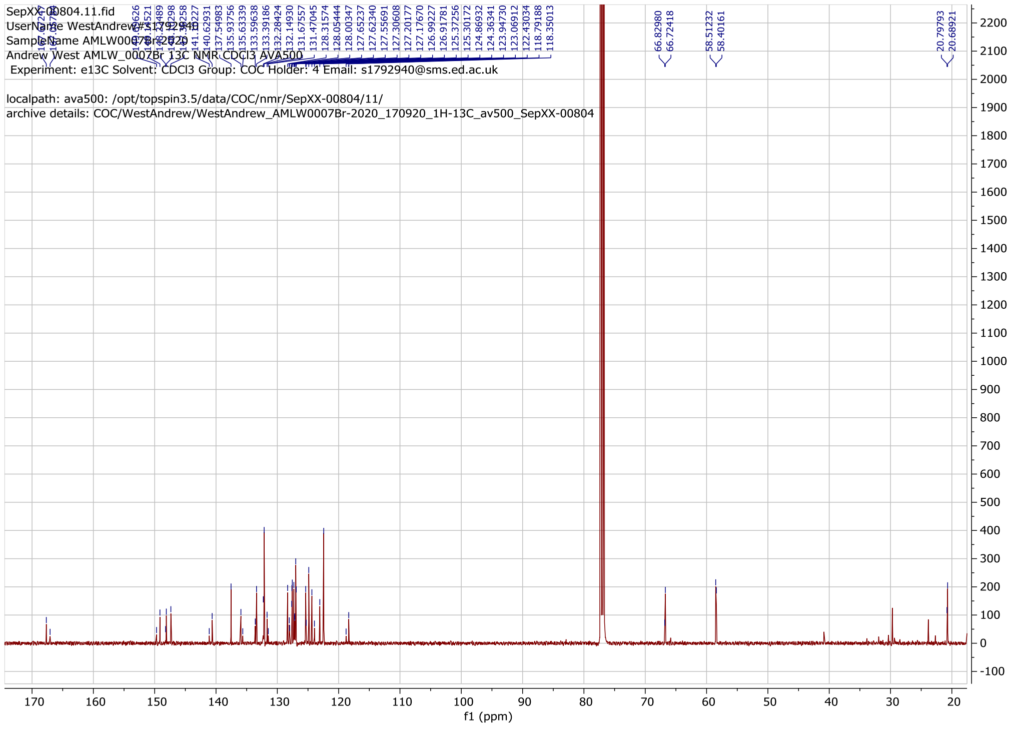

Step VII (±)-2l

4-Iodophenyl 3-methyl-2-[6*H*,12*H*-5,11-methano dibenzo[*b,f*][1,5]diazocin-2-yl]benzoate

2-(6*H*,12*H*-5,11-methanodibenzo[*b,f*][1,5]diazocin-2-yl)-3-methyl-benzoic acid (1 eq, 0.070 g), and 4-iododphenol (3 eq, 0.13 g), and PyBOP (2 eq, 0.21 g) were dissolved in DCM (5 mL). Triethylamine (8 eq, 0.73 mL) was added to the resultant mixture, before the solution was stirred at room temperature for 72 hours. Additional DCM (50 mL) was added to the reaction mixture before the mixture was washed with saturated aqueous sodium bicarbonate solution (30 mL) and brine (2 x 30 mL). Organics were then dried on magnesium sulfate before the solvent was removed under reduced pressure and the resultant residue was purified by flash chromatography (20% ethyl acetate in petroleum ether). 4-Iodophenyl 3-methyl-2-[6*H*,12*H*-5,11-methano dibenzo[*b,f*][1,5]diazocin-2-yl]benzoate was isolated as a while solid (0.13 g, 88%).

^{1}H NMR (500 MHz, $CDCl_3$) δ 7.75 (d, J = 7.2 Hz, 0.5H, minor conformer), 7.66 (d, J = 7.6, 0.6H, major conformer), 7.44 (t, 1H), 7.36 (t, 1H), 7.27-7.23 (m, 1H), 7.23-7.06 (m, 5H), 7.06-6.96 (m, 2H), 6.84-6.81 (m, 0.7H major conformer), 6.80-6.78 (m, 0.4H minor conformer), 6.55 (d, J = 8.7 Hz, 0.6H minor conformer), 5.88 (d, J = 8.8 Hz, 1.3H, major conformer), 4.43-4.32 (m, 1.5H, major conformer), 4.70-4.60 (m, 0.5H, minor conformer), 4.43-4.32 (m, 2H), 4.31-4.15 (m, 2H), 2.17 (s, 2H, major conformer), 2.10 (s, 1H, minor conformer).

^{13}C NMR (126 MHz, $CDCl_3$) δ 167.65, 167.29, 149.98, 148.11, 147.38, 145.97, 144.54, 143.4, 138.29, 138.11, 137.54, 135.95, 135.79, 133.38, 132.16, 131.69, 128.34, 128.32, 127.65, 127.63, 127.56, 127.31, 127.13, 126.99, 126.96, 126.92, 126.90, 126.22, 125.53, 125.39, 125.31, 124.87, 124.39, 67.99, 66.71, 58.49, 58.37, 22.70, 20.68. Two conformers. Some peaks occluded.

HRMS (ESI) obtained m/z 581.3996. ($M+Na^+$). Expected 581.4087.

^{1}H NMR

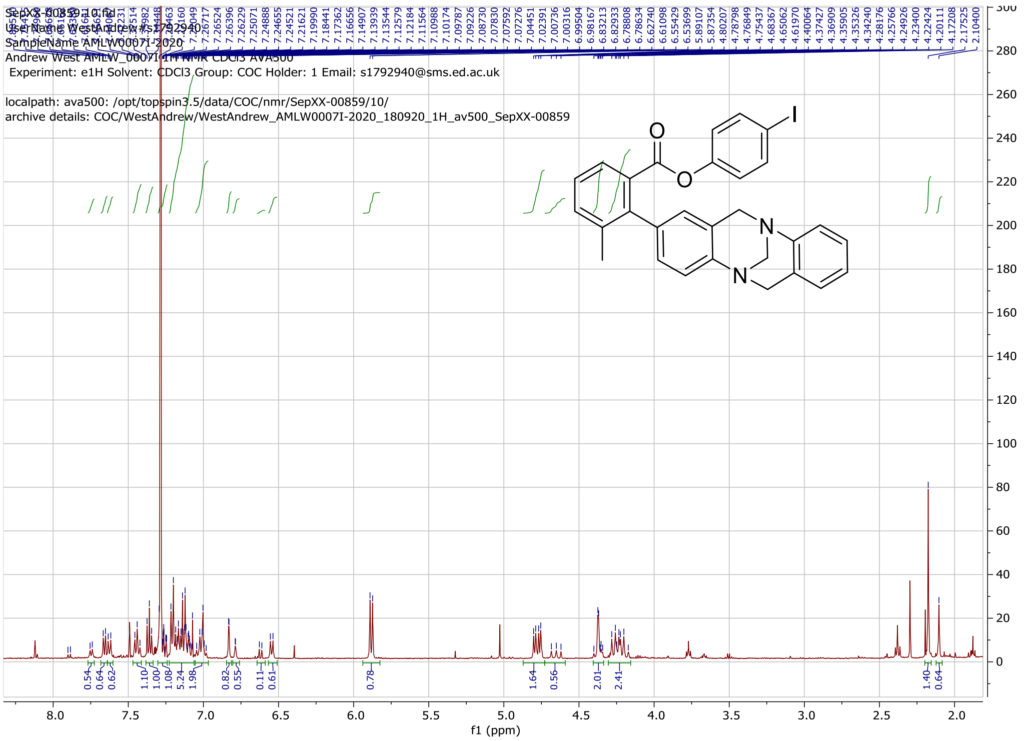

^{13}H NMR

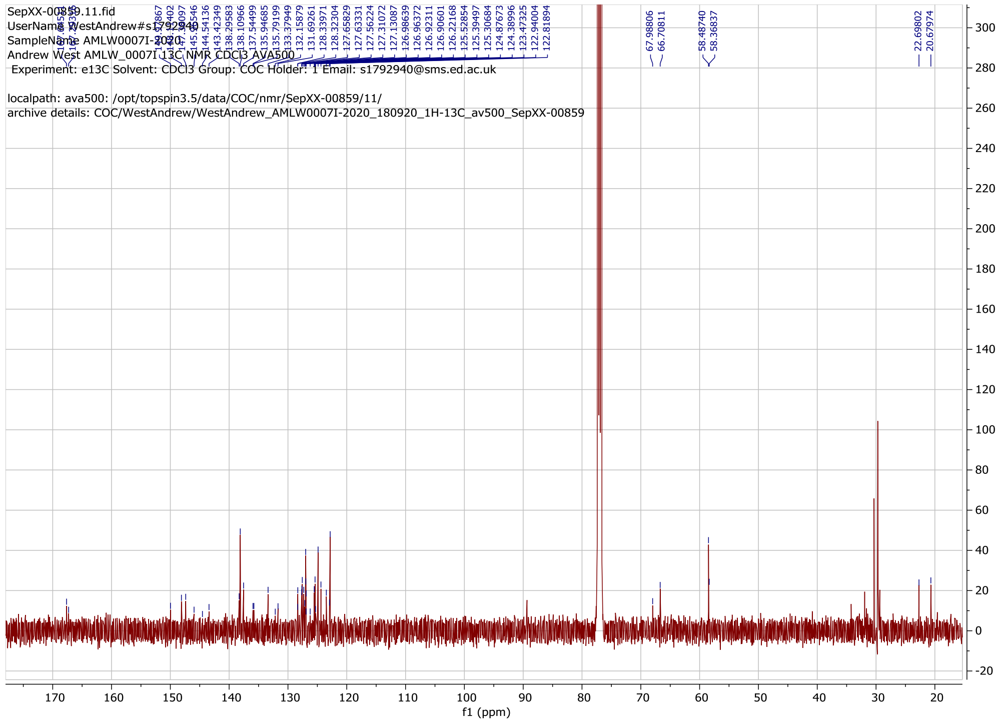

Step VII (±)-2H

Phenyl 3-methyl-2-[6*H*,12*H*-5,11-methano dibenzo[*b,f*][1,5]diazocin-2-yl]benzoate

To a solution of 2-(4,4,5,5-tetramethyl-1,3,2-dioxaborolan-2-yl)-6*H*,12*H*-5,11-methanodibenzo[*b,f*][1,5]diazocine (1 eq, 0.50 g) dissolved in acetonitrile (10 mL) was added $Pd(OAc)_2$ (0.02 eq, 0.0065 g), SPhos (0.04 eq, 0.024 g) and methyl 2-bromo-3-phenol benzoate (1.5 Eq, 0.69 g). 2 M potassium carbonate solution (2.5 Eq, 1.44 mL) was added to the resultant solution before being heated to reflux under an inert nitrogen atmosphere and refluxed for 16 hours. The reaction mixture was diluted with ethyl acetate (250 mL) and washed with saturated sodium bicarbonate solution (2 x 150 mL) and brine (100 mL) before organics were dried over magnesium sulfate. The solvent was removed under reduced pressure and the crude reaction mixture was purified by flash chromatography (30% ethyl acetate in petroleum ether) to yield phenyl 3-methyl-2-[6*H*,12*H*-5,11-methano dibenzo[*b,f*][1,5]diazocin-2-yl]benzoate (0.34 g, 54%).

^{1}H NMR (500 MHz, $CDCl_3$) δ 7.77 (d, J = 7.6 Hz, 0.4H, minor conformer), 7.69 (d, J = 7.6 Hz, 0.6H, major conformer), 7.44 (m, 1H), 7.34 (m, 1H), 7.26-7.09 (m, 5H), 7.07-6.97 (m, 2H), 6.92 (t, 1H), 6.86 (s, 0.6H, major conformer), 6.82 (s, 0.4H, minor conformer), 6.79 (d, J = 7.9 Hz, 0.8H, minor conformer), 6.17 (d, J = 7.8 Hz, 1.1 H, major conformer), 4.78 (dd, J = 16.8 Hz, 1.6H, major conformer), 4.69 (d, J = 16.7 Hz, 0.4H minor conformer), 4.44-4.34 (m, 2H), 4.33-4.19 (m, 2H), 2.18 (s, 1.9H, major conformer), 2.11 (s, 1.1H minor conformer).

^{13}C NMR (126 MHz, $CDCl_3$) δ 168.0, 167.4, 150.7, 150.2, 148.3, 148.1, 147.2, 141.0, 140.6, 137.5, 135.9, 135.7, 133.4, 133.2, 132.1, 132.0, 129.7, 129.3, 129.2, 128.4, 128.1, 127.9, 127.7, 127.5, 127.2, 127.1, 127.0, 125.7, 125.3, 125.3, 124.9, 124.2, 123.9, 121.5, 121.3, 120.8, 66.8, 66.7, 58.5, 58.4, 29.7, 29.7. Two conformers. Some peaks occluded.

HRMS (ESI) obtained m/z 433.1671. ($M+H^+$). Expected 433.1911.

^{1}H NMR

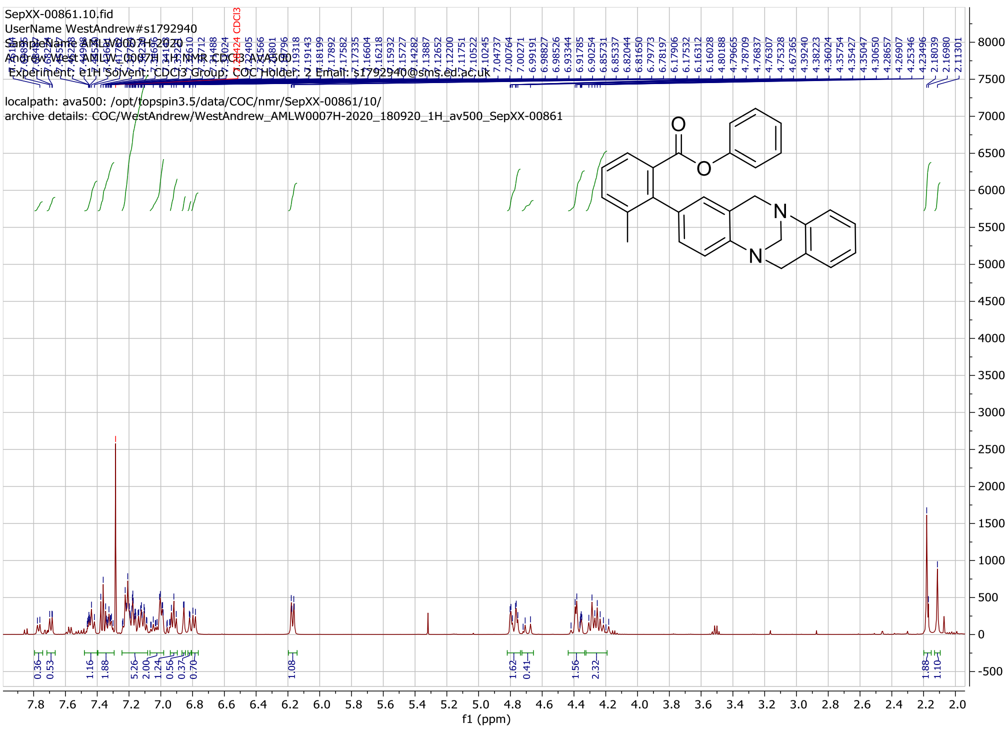

^{13}C NMR

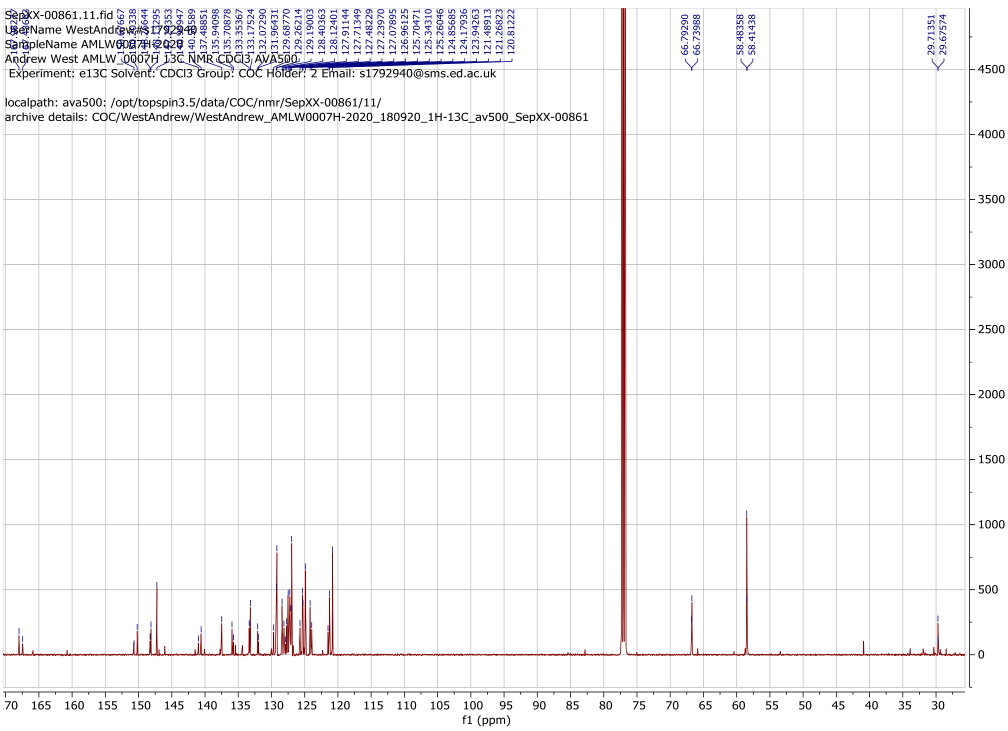

Step VII (±)-2Me

4-Methylphenyl 3-methyl-2-[6*H*,12*H*-5,11-methano dibenzo[*b,f*][1,5]diazocin-2-yl]benzoate

To a solution of 2-(4,4,5,5-tetramethyl-1,3,2-dioxaborolan-2-yl)-6*H*,12*H*-5,11-methanodibenzo[*b,f*][1,5]diazocine (1 eq, 0.50 g) dissolved in acetonitrile (10 mL), palladium(II)acetate (0.02 eq, 0.0065 g), SPhos (0.04 eq, 0.024 g) and methyl 2-bromo-3-(4-methyl phenol)-benzoate (1.5 eq, 0.72 g) were added. 2 M potassium carbonate solution (2.5 eq, 1.44 mL) was to the resultant solution before being heated to reflux under an inert nitrogen atmosphere for 16 hours. The reaction mixture was diluted with ethyl acetate (250 mL) and washed with saturated sodium bicarbonate solution (2 x 150 mL) and brine (100 mL) before organics were dried over magnesium sulfate. The solvent was removed under reduced pressure and the crude reaction mixture was purified by flash chromatography (30% ethyl acetate in petroleum ether) to yield 4-methylphenyl 3-methyl-2-[6*H*,12*H*-5,11-methano dibenzo[*b,f*][1,5]diazocin-2-yl]benzoate (0.22 g, 34%).

^{1}H NMR (500 MHz, $CDCl_3$) δ 7.75 (d, J = 7.6 Hz, 0.4H, minor conformer), d, J = 7.6 Hz, 0.6H, major conformer), 7.44 (t, 1H), 7.35 (t, 1H), 7.26-7.09 (m, 6H), 7.07-6.98 (m, 1H), 6.85 (s, 0.6H, major conformer), 6.81 (s, 0.4H, minor conformer), 6.67 (dd, 2H), 6.04 (d, 1H), 4.78 (dt, J = 16.7 Hz, 1.6H, major conformer), 4.69 (d, J = 16.8 Hz, 0.6H, minor conformer), 4.42-4.14 (m, 4H), 2.32 (s, 1.2H, minor conformer), 2.23 (s, 1.8H, major conformer), 2.17 (s, 1.8H, major conformer), 2.11 (s, 1.2H, minor conformer).

^{13}C NMR (126 MHz, $CDCl_3$) δ 168.2, 167.7, 148.4, 148.3, 148.2, 148.0, 147.3, 140.9, 140.5, 137.4, 137.4, 136.0, 135.7, 135.3, 134.8, 133.2, 133.1, 132.3, 132.1, 129.8, 129.7, 128.4, 128.1, 128.1, 127.8, 127.5, 127.5, 127.2, 127.0, 126.8, 125.4, 125.3, 124.8, 124.1, 123.9, 121.1, 120.9, 120.4, 66.8, 66.7, 58.5, 58.5, 58.4, 58.3, 20.8, 20.8, 20.7, 20.7. Two conformers. Some peaks occluded.

HRMS (ESI) obtained m/z 447.1886. (M+H+). Expected 447.2017.

^{1}H NMR

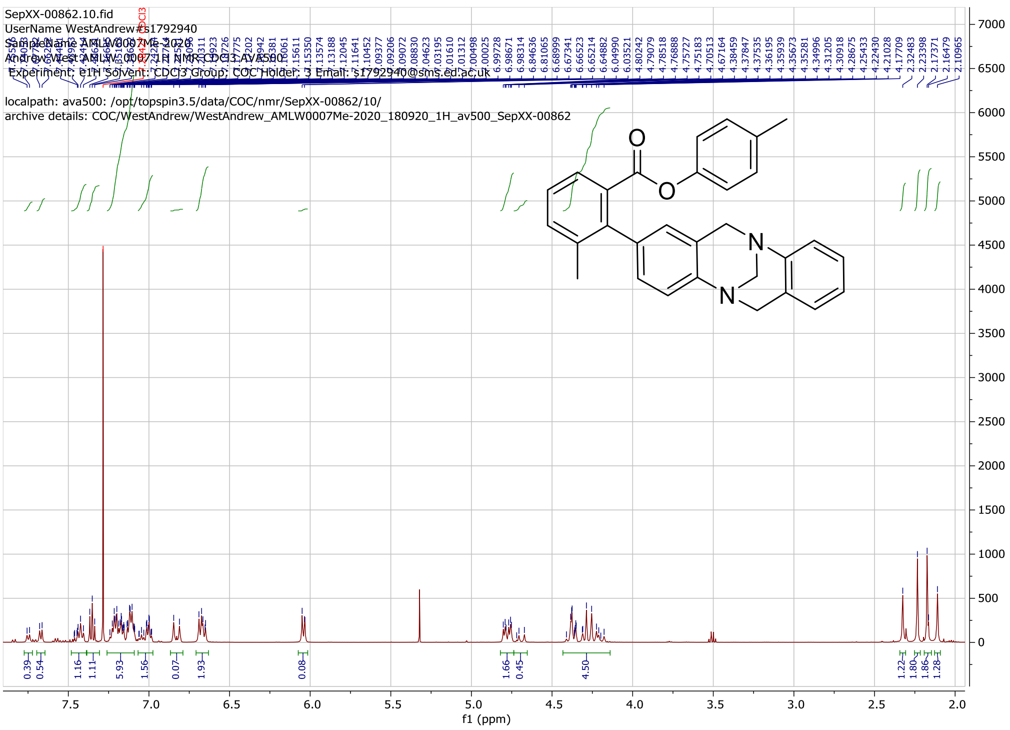

^{13}C NMR

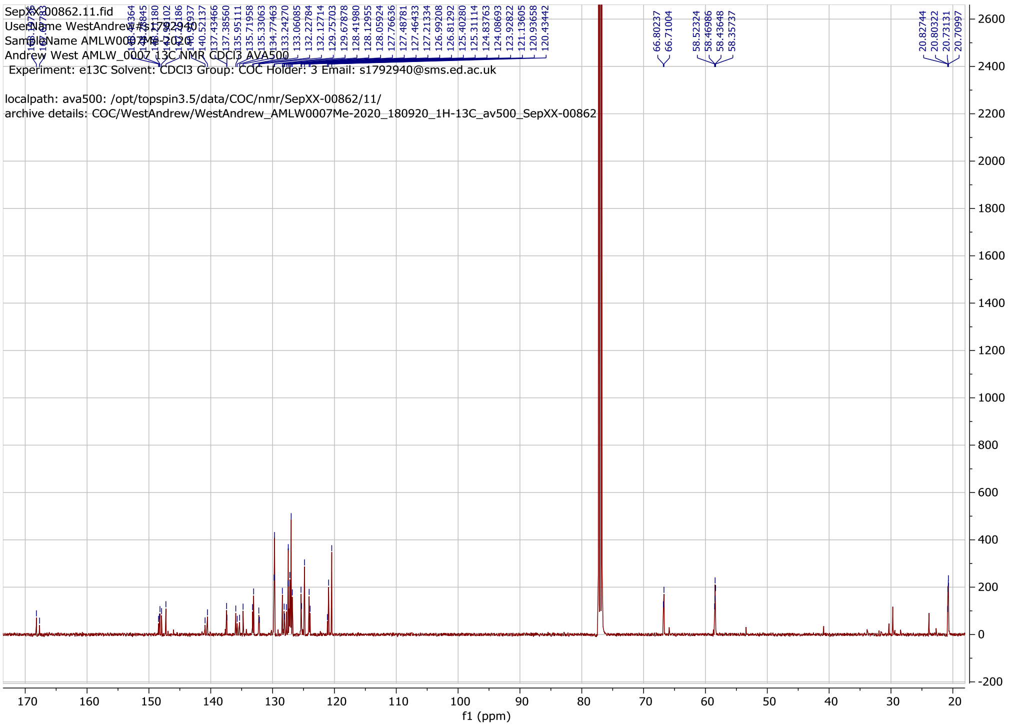

S3. Crystallographic data

Compound (±)-**1H** CCDC Deposition Number 2244209

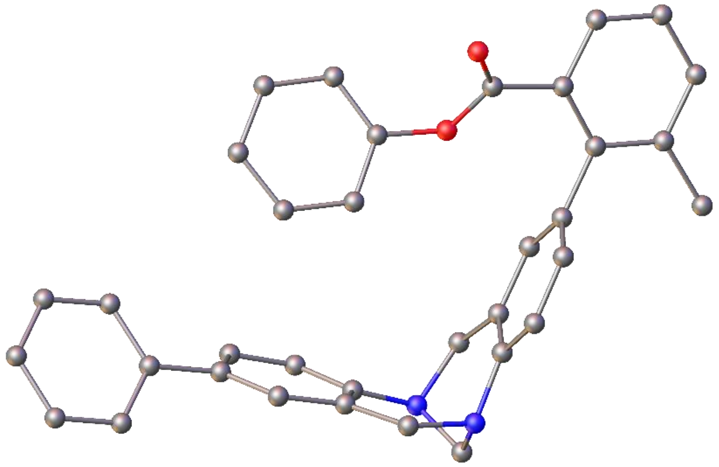

**Compound**	**SC16017**
Formula	$C_{35}H_{28}N_2O_2$
$D_{calc.}$/ g cm^{-3}	1.281
μ/mm^{-1}	0.625
Formula Weight	508.59
Colour	colourless
Shape	plate
Size/mm^3	0.49×0.06×0.03
T/K	120.0
Crystal System	monoclinic
Space Group	I2/a
a/Å	35.4860(15)
b/Å	6.2732(2)
c/Å	48.865(2)
α/°	90
β/°	104.050(4)
γ/°	90
V/Å^3	10552.4(8)
Z	16
Z'	2
Wavelength/Å	1.54184
Radiation type	Cu K$_\alpha$
Θ_{min}/°	3.521
Θ_{max}/°	68.246
Measured Refl.	54755
Independent Refl.	9692
Reflections Used	7229
R_{int}	0.1017
Parameters	705
Restraints	0
Largest Peak	0.476
Deepest Hole	-0.377
GooF	1.037
wR_2 (all data)	0.2251
wR_2	0.2040
R_1 (all data)	0.0993
R_1	0.0774

Experimental. Single colourless plate-shaped crystals of (**SC16017**) were recrystallised from a mixture of ethanol and DCM by slow evaporation. A suitable crystal (0.49×0.06×0.03) mm^3 was selected and mounted on a MITIGEN holder in Paratone oil on a Rigaku Oxford Diffraction SuperNova diffractometer. The crystal was kept at T = 120.0 K during data collection. Using **Olex2** (Dolomanov et al., 2009), the structure was solved with the **ShelXS** (Sheldrick, 2008) structure solution program, using the Direct Methods solution method. The model was refined with version 2016/6 of **ShelXL** (Sheldrick, 2015) using Least Squares minimisation.

Crystal Data. $C_{35}H_{28}N_2O_2$, M_r = 508.59, monoclinic, I2/a (No. 15), a = 35.4860(15) Å, b = 6.2732(2) Å, c = 48.865(2) Å, β = 104.050(4)°, α = γ = 90°, V = 10552.4(8) Å^3, T = 120.0 K, Z = 16, Z' = 2, μ(Cu K$_\alpha$) = 0.625, 54755 reflections measured, 9692 unique (R_{int} = 0.1017) which were used in all calculations. The final wR_2 was 0.2251 (all data) and R_1 was 0.0774 (I > 2(I)).

Structure Quality Indicators

**Reflections:**	d min (Cu) 0.83	I/σ 8.0	Rint 10.17%	complete at 2θ=136° 100%
**Refinement:**	Shift 0.000	Max Peak 0.5	Min Peak -0.4	GooF 1.037

A colourless plate-shaped crystal with dimensions 0.49×0.06×0.03 mm^3 was mounted on a MITIGEN holder in Paratone oil.X-ray diffraction data were collected using a Rigaku Oxford Diffraction SuperNova diffractometer equipped with a Oxford Cryosystems Cryostream 700+ low-temperature device, operating at T = 120.0 K.

Data were measured using ω scans scans of 1.0 ° per frame for 5.0 s using Cu K_{α} radiation (micro-focus sealed X-ray tube, 50 kV, 0.8 mA). The total number of runs and images was based on the strategy calculation from the program CrysAlisPro (Agilent).The maximum resolution achieved was Θ = 68.246. °

Cell parameters were retrieved using the CrysAlisPro (Agilent) software and refined using CrysAlisPro (Agilent) on 8510 reflections, 16 % of the observed reflections. Data reduction was performed using the CrysAlisPro (Agilent) software which corrects for Lorentz polarisation. The final completeness is 99.94 out to 68.246 in Θ. The absorption coefficient μ of this material is 0.625 at this wavelength (λ = 1.54184) and the minimum and maximum transmissions are 0.750 and 1.000.

The structure was solved in the space group I2/a (# 15) by Direct Methods using the **ShelXS** (Sheldrick, 2008) structure solution program and refined by Least Squares using version 2016/6 of **ShelXL** (Sheldrick, 2015). All non-hydrogen atoms were refined anisotropically. Hydrogen atom positions were calculated geometrically and refined using the riding model.

The value of Z' is 2. This means that there are two independent molecules in the asymmetric unit.

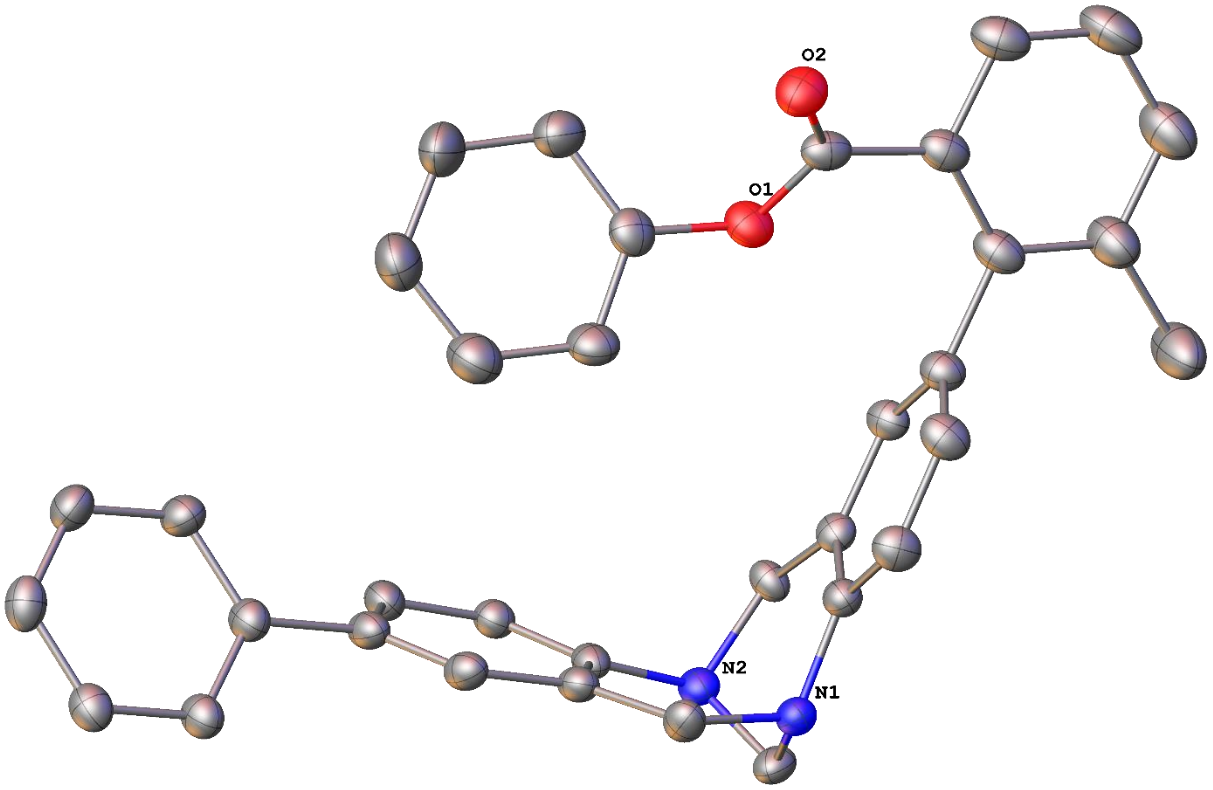

Figure S4: One of the two unique molecules in the asymmetric unit of structure SC16017. Displacement ellipsoids are at the 50% probability level and H atoms are not shown.

Data Plots: Diffraction Data

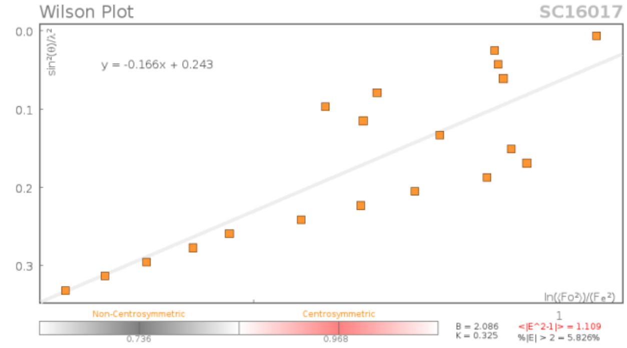

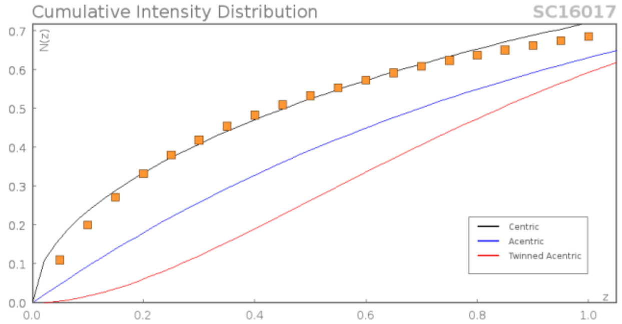

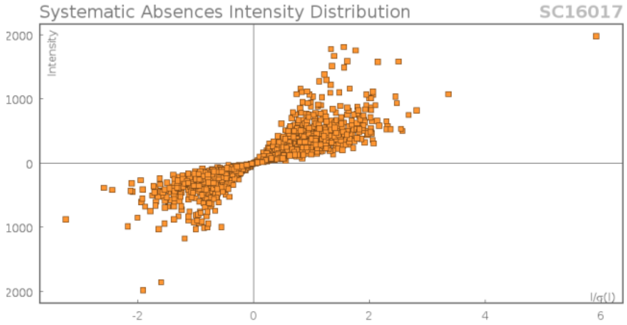

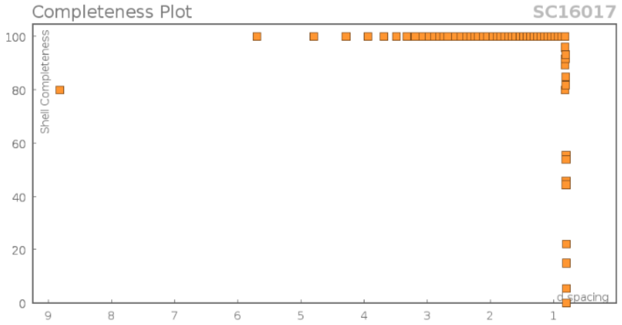

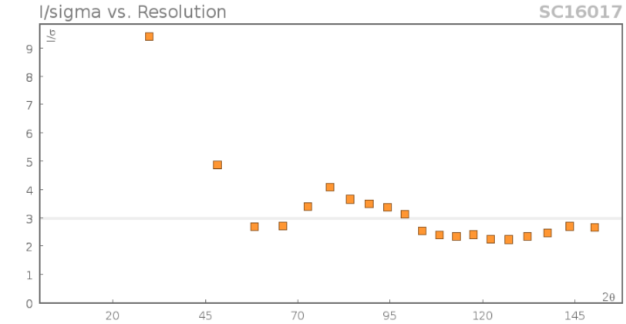

Data Plots: Refinement and Data

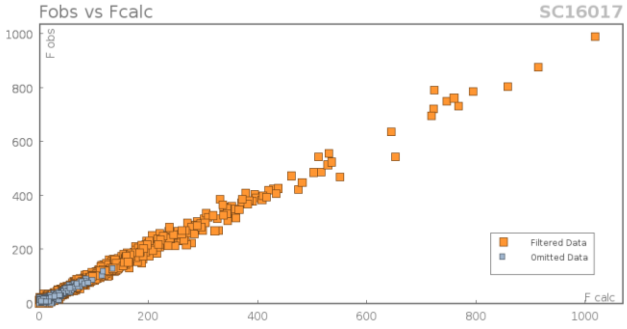

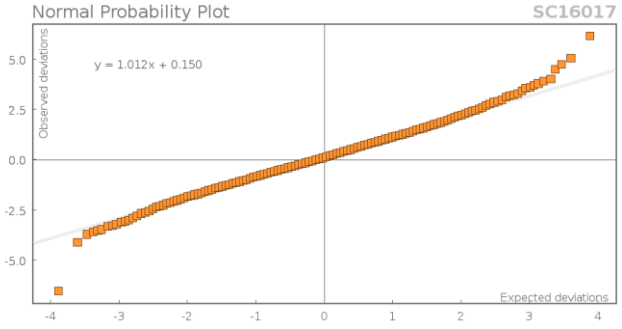

Reflection Statistics

Total reflections (after filtering)	57456	Unique reflections	9692
Completeness	1.0	Mean I/σ	7.98
hkl_{max} collected	(43, 6, 56)	hkl_{min} collected	(-44, -7, -60)
hkl_{max} used	(41, 7, 58)	hkl_{min} used	(-42, 0, 0)
Lim d_{max} collected	999.0	Lim d_{min} collected	0.83

d_{max} used	13.2	d_{min} used	0.83
Friedel pairs	9984	Friedel pairs merged	1
Inconsistent equivalents	0	R_{int}	0.1017
R_{sigma}	0.0611	Intensity transformed	0
Omitted reflections	0	Omitted by user (OMIT hkl)	0
Multiplicity	(14633, 9481, 4282, 1874, 762, 371, 87, 45, 13)	Maximum multiplicity	18
Removed systematic absences	2701	Filtered off (Shel/OMIT)	3603

Table S19: Fractional Atomic Coordinates (×10[4]) and Equivalent Isotropic Displacement Parameters (Å^2×10^3) for **SC16017**. U_{eq} is defined as 1/3 of the trace of the orthogonalised U_{ij}.

Atom	x	y	z	U_{eq}
O51	3311.1(6)	4649(3)	1326.9(4)	28.8(4)
O52	2658.4(6)	4947(4)	1238.9(5)	34.5(5)
N51	4378.0(7)	11296(3)	1379.9(5)	21.9(5)
C66	4208.0(7)	7671(4)	1186.0(5)	20.2(5)
N52	4833.4(6)	8393(3)	1534.3(5)	22.0(5)
C67	4100.0(8)	9766(4)	1227.9(6)	21.3(5)
C53	4394.5(8)	9864(4)	2131.5(6)	23.6(5)
C52	4484.4(7)	9937(4)	1869.3(6)	21.3(5)
C57	4715.8(7)	8335(4)	1794.9(6)	20.4(5)
C71	3933.9(8)	6304(4)	1023.3(6)	22.5(5)
C51	4323.7(8)	11727(4)	1665.2(6)	23.8(5)
C54	4522.5(8)	8210(4)	2323.2(6)	22.5(5)
C56	4845.8(8)	6683(4)	1984.5(6)	22.9(5)
C65	4617.0(7)	6892(4)	1320.7(6)	21.6(5)
C58	4400.8(8)	8069(4)	2594.2(6)	24.7(6)
C68	3719.3(8)	10417(4)	1120.9(6)	27.0(6)
C76	2677.4(9)	3237(5)	688.2(7)	32.3(6)
C72	3256.7(8)	5450(4)	742.6(6)	26.2(6)
C61	4156.0(9)	7730(5)	3100.4(6)	34.1(7)
C79	2947.4(8)	4777(4)	1154.5(6)	25.1(6)
C80	3359.0(8)	4669(5)	1619.4(6)	26.1(6)
C70	3550.8(8)	6939(4)	912.7(6)	24.4(6)
C69	3445.4(8)	9004(5)	966.6(6)	27.5(6)
C81	3539.7(8)	6418(5)	1765.3(7)	31.4(6)
C64	4775.5(8)	10530(4)	1411.7(6)	25.3(6)
C55	4752.1(8)	6611(4)	2241.2(6)	24.7(5)
C77	2963.9(8)	4547(4)	853.6(6)	27.0(6)
C60	4101.5(9)	9600(5)	2946.8(7)	35.3(7)
C63	4452.7(9)	6185(5)	2753.1(6)	30.1(6)
C82	3616.1(9)	6401(6)	2057.9(7)	39.1(7)
C85	3255.1(10)	2909(5)	1752.6(7)	36.4(7)
C59	4220.6(9)	9776(5)	2697.1(7)	31.5(6)
C75	2689.2(10)	2792(5)	413.2(7)	38.5(7)
C83	3520.7(10)	4646(7)	2198.3(7)	43.4(8)
C62	4336.9(10)	6023(5)	3001.7(7)	36.7(7)
C73	3260.5(9)	5019(5)	460.4(7)	32.6(6)
C84	3340.4(11)	2906(6)	2045.3(8)	43.6(8)
C74	2975.5(10)	3682(6)	301.1(7)	39.5(7)
C78	3558.9(10)	5996(6)	326.8(7)	43.2(8)
O1	1959.4(6)	9849(3)	1170.3(5)	30.2(4)
N2	415.4(6)	13335(4)	1039.7(5)	22.9(5)
O2	2612.9(6)	9914(4)	1243.3(5)	34.3(5)
N1	864.1(7)	16312(4)	1129.1(5)	23.7(5)
C16	1071.7(8)	12825(4)	1353.5(6)	21.7(5)
C7	483.6(7)	13127(4)	765.1(6)	22.2(5)
C21	1371.8(8)	11561(4)	1513.1(6)	22.8(5)
C2	678.7(8)	14701(4)	651.1(6)	23.7(5)
C30	1898.4(8)	9815(5)	875.3(7)	29.3(6)
C13	679.4(9)	10826(5)	-210.0(6)	29.5(6)

Atom	x	y	z	U_{eq}
C6	330.1(8)	11353(4)	601.3(6)	24.3(5)
C29	2327.6(8)	9880(4)	1335.2(7)	26.1(6)
C9	411.0(8)	14364(5)	-278.9(6)	27.9(6)
C1	873.1(8)	16550(4)	831.1(6)	26.0(6)
C3	691.8(8)	14529(4)	368.8(6)	25.5(6)
C12	686.6(9)	10736(5)	-492.6(7)	31.2(6)
C15	663.2(8)	11960(4)	1255.8(6)	22.3(5)
C10	420.0(8)	14262(5)	-560.6(6)	28.4(6)
C4	537.2(8)	12774(4)	203.2(6)	27.0(6)
C27	2331.1(8)	9808(5)	1642.1(7)	28.1(6)
C26	2622.7(9)	8533(5)	1810.3(8)	35.9(7)
C14	479.1(8)	15538(4)	1139.5(6)	26.6(6)
C31	1671.7(9)	11429(5)	731.3(7)	33.7(7)
C18	1544.2(8)	15614(4)	1355.3(6)	28.6(6)
C5	365.4(8)	11153(5)	326.9(6)	26.3(6)
C17	1161.1(8)	14887(4)	1279.4(6)	22.4(5)
C11	558.9(9)	12457(5)	-667.7(6)	30.4(6)
C8	544.2(8)	12647(5)	-98.9(6)	26.5(6)
C22	2070.7(8)	10925(5)	1760.3(7)	29.8(6)
C19	1837.5(8)	14335(5)	1504.1(6)	29.6(6)
C20	1753.2(8)	12290(5)	1589.2(6)	26.3(6)
C23	2113.3(10)	10853(6)	2054.1(7)	39.1(7)
C32	1582.4(10)	11389(6)	437.3(8)	42.0(8)
C35	2035.3(10)	8154(5)	742.0(8)	39.4(7)
C25	2655.3(10)	8409(6)	2095.3(8)	43.3(8)
C34	1946.9(11)	8144(6)	450.4(8)	46.2(8)
C33	1719.7(11)	9766(6)	296.2(8)	44.8(8)
C24	2407.1(10)	9578(6)	2217.1(8)	44.9(8)
C28	1853.0(12)	12124(8)	2191.9(8)	53.3(10)

Table S20: Anisotropic Displacement Parameters (×10[4]) **SC16017.** The anisotropic displacement factor exponent takes the form: $-2\pi^2[h^2a^{*2} \times U_{11} + ... + 2hka^* \times b^* \times U_{12}]$

Atom	U_{11}	U_{22}	U_{33}	U_{23}	U_{13}	U_{12}
O51	19.8(9)	41.4(11)	25.1(10)	0.0(8)	5.5(8)	-0.1(8)
O52	21.8(10)	44.2(12)	38.3(12)	3.5(10)	8.8(9)	2.9(9)
N51	23.5(11)	21.9(10)	20.7(11)	0.0(8)	5.9(9)	-4.3(9)
C66	18.1(12)	25.1(12)	18.8(12)	2.4(10)	7.3(10)	-2.3(10)
N52	20.1(11)	25.1(10)	21.6(11)	2.4(9)	6.6(9)	-3.4(9)
C67	21.4(12)	22.8(12)	20.0(12)	3(1)	5.9(10)	-2.2(10)
C53	23.0(13)	24.6(12)	23.3(13)	-3(1)	5.8(10)	-5(1)
C52	19.4(12)	23.0(12)	20.6(12)	-0.1(10)	3.2(10)	-5.7(10)
C57	15.3(11)	25.3(12)	20.5(13)	0.4(10)	4.1(10)	-4.3(10)
C71	22.6(13)	23.3(12)	23.3(13)	0.4(10)	8.7(11)	-0.8(10)
C51	29.7(14)	19.6(11)	22.7(13)	-0.4(10)	7.3(11)	-2(1)
C54	21.3(12)	24.1(12)	21.8(13)	-0.9(10)	4.9(10)	-5.6(10)
C56	20.4(12)	25.6(12)	23.1(13)	0.3(10)	5.9(10)	-1.6(10)
C65	17.5(12)	26.4(12)	22.0(13)	-1.4(10)	6.8(10)	-0.5(10)
C58	21.1(12)	29.5(13)	23.1(13)	-0.3(11)	4.8(10)	-4(1)
C68	26.2(14)	24.9(12)	29.5(14)	4.2(11)	6.2(11)	1.4(11)
C76	22.9(14)	33.4(14)	37.1(17)	2.5(13)	0.4(12)	-4.6(12)
C72	21.2(13)	27.6(13)	26.5(14)	2.2(11)	-0.4(11)	1.6(11)
C61	35.2(16)	46.5(17)	22.2(14)	-2.0(12)	10.5(12)	-9.9(13)
C79	18.6(12)	23.5(12)	32.0(15)	0.1(11)	4.1(11)	1.6(10)
C80	19.7(12)	33.1(14)	25.6(14)	1.5(11)	5.9(10)	3.8(11)
C70	20.4(13)	28.0(13)	23.9(13)	2.1(11)	3.8(10)	-4.3(10)
C69	20.2(13)	32.5(14)	28.4(15)	2.5(11)	3.1(11)	2.6(11)
C81	21.5(13)	39.3(15)	33.9(16)	-2.0(13)	7.5(12)	-4.7(12)
C64	22.3(13)	27.6(13)	27.0(14)	3.1(11)	7.9(11)	-5.4(10)
C55	22.2(13)	27.6(13)	22.7(13)	4.6(10)	2.2(10)	-1(1)
C77	22.6(13)	26.1(13)	31.0(15)	4.2(11)	3.8(11)	2.4(11)

Atom	U_{11}	U_{22}	U_{33}	U_{23}	U_{13}	U_{12}
C60	34.4(16)	41.4(16)	33.5(16)	-3.3(13)	14.8(13)	1.1(13)
C63	39.0(16)	30.8(14)	23.0(14)	3.2(11)	12.5(12)	4.6(12)
C82	24.4(15)	58(2)	34.5(17)	-11.8(15)	5.5(13)	-10.2(14)
C85	34.3(16)	37.3(16)	39.0(18)	0.8(14)	11.3(13)	-6.1(13)
C59	32.9(15)	33.6(14)	30.9(15)	5.4(12)	13.4(12)	6.7(12)
C75	33.9(16)	42.0(17)	33.0(17)	-3.8(13)	-4.8(13)	-9.0(13)
C83	31.1(16)	71(2)	28.5(16)	0.9(16)	7.1(13)	-5.6(16)
C62	48.3(19)	37.2(15)	26.6(16)	5.3(12)	13.2(14)	-2.6(14)
C73	28.5(15)	39.3(15)	26.7(15)	0.5(12)	0.3(12)	-0.4(12)
C84	43.4(19)	51.6(19)	37.4(18)	12.3(15)	13.2(15)	0.1(16)
C74	36.3(17)	50.4(19)	27.7(16)	-1.1(14)	-0.3(13)	-3.4(14)
C78	38.2(18)	64(2)	25.9(16)	4.8(15)	4.8(13)	-5.1(16)
O1	20.9(10)	37.7(10)	31.9(11)	2.5(9)	6.3(8)	-0.6(8)
N2	18.7(10)	28.1(11)	23.2(12)	0.4(9)	7.6(9)	3.8(9)
O2	25.2(11)	40.5(11)	38.6(12)	-3.6(9)	10.8(9)	-2.6(9)
N1	23.5(11)	23.9(10)	24.5(12)	-0.5(9)	7.4(9)	3.2(9)
C16	20.4(13)	26.2(12)	20.7(12)	-0.7(10)	9.3(10)	1(1)
C7	18.5(12)	27.9(12)	20.4(13)	2.1(10)	5.1(10)	6.3(10)
C21	22.7(13)	25.6(12)	22.3(13)	5.1(10)	9.7(11)	2.8(10)
C2	23.9(13)	23.6(12)	23.7(13)	2.7(10)	6.1(10)	6.8(10)
C30	20.0(13)	34.1(14)	32.6(16)	-1.0(12)	4.0(11)	-4.4(11)
C13	30.7(15)	29.9(13)	27.9(15)	1.4(11)	7.0(12)	7.1(11)
C6	22.4(12)	25.4(12)	24.4(14)	3.2(11)	4.1(11)	2.9(10)
C29	19.9(13)	22.4(12)	36.8(16)	-0.1(11)	8.3(11)	-1.5(10)
C9	27.6(14)	32.6(14)	25.1(14)	1.4(11)	9.3(11)	4.1(11)
C1	30.9(14)	22.6(12)	25.0(14)	3.2(11)	7.8(11)	3.0(11)
C3	27.6(13)	25.5(12)	24.7(14)	4.6(11)	9.1(11)	5.2(11)
C12	34.0(15)	30.7(14)	30.8(16)	-6.3(12)	11.6(12)	2.0(12)
C15	19.7(12)	28.4(13)	19.4(12)	4(1)	6.1(10)	0.7(10)
C10	24.9(13)	36.5(14)	24.5(14)	3.4(12)	7.7(11)	-1.3(11)
C4	28.3(14)	28.1(13)	25.5(14)	3.0(11)	8.3(11)	7.6(11)
C27	21.4(13)	28.3(13)	34.0(16)	4.6(11)	5.5(11)	-2.2(11)
C26	25.5(14)	37.1(15)	46.2(19)	8.6(14)	11.0(13)	4.3(12)
C14	23.5(13)	30.1(13)	26.7(14)	-3.8(11)	7.3(11)	6.9(11)
C31	25.7(14)	38.5(15)	38.7(17)	-0.4(13)	11.1(13)	6.9(12)
C18	26.5(14)	26.7(13)	33.5(16)	2.9(11)	9.1(12)	-2.3(11)
C5	24.3(13)	31.3(13)	22.0(14)	0.1(11)	3.4(11)	5.5(11)
C17	22.7(13)	25.2(12)	20.6(12)	0.6(10)	7.4(10)	2.9(10)
C11	30.6(15)	38.6(15)	24.2(14)	-3.7(12)	10.9(12)	-9.2(12)
C8	24.3(13)	33.6(14)	22.5(14)	2.2(11)	7.4(11)	2.4(11)
C22	20.9(13)	35.3(14)	33.0(16)	8.9(12)	6.4(12)	1.9(11)
C19	21.6(13)	34.6(14)	32.7(15)	4.4(12)	6.9(11)	-4.0(11)
C20	22.9(13)	30.6(13)	25.7(14)	2.9(11)	6.6(11)	3.4(11)
C23	30.9(16)	53.0(19)	33.8(17)	9.3(15)	8.5(13)	5.9(14)
C32	34.4(17)	48.3(19)	41.2(19)	5.9(15)	5.4(14)	8.1(14)
C35	35.3(17)	36.8(16)	43.5(19)	-4.2(14)	4.3(14)	5.7(13)
C25	29.5(16)	53.9(19)	44(2)	20.6(16)	5.0(14)	9.1(14)
C34	46(2)	47.8(19)	40.1(19)	-12.5(16)	1.0(16)	5.6(16)
C33	42.3(19)	56(2)	31.2(17)	-6.3(15)	-0.2(14)	1.4(16)
C24	37.7(18)	62(2)	34.4(18)	16.1(16)	7.4(14)	3.4(16)
C28	46(2)	82(3)	33.7(18)	8.3(19)	12.5(16)	19(2)

Table S21: Bond Lengths in Å for **SC16017**.

Atom	Atom	Length/Å
O51	C79	1.361(3)
O51	C80	1.398(3)
O52	C79	1.199(3)
N51	C67	1.446(3)
N51	C51	1.478(3)
N51	C64	1.462(3)
C66	C67	1.398(4)
C66	C71	1.392(4)
C66	C65	1.522(4)
N52	C57	1.434(3)
N52	C65	1.475(3)
N52	C64	1.462(3)

Atom	Atom	Length/Å
C67	C68	1.386(4)
C53	C52	1.394(4)
C53	C54	1.398(4)
C52	C57	1.400(4)
C52	C51	1.519(4)
C57	C56	1.392(4)
C71	C70	1.393(4)
C54	C58	1.492(4)
C54	C55	1.410(4)
C56	C55	1.374(4)
C58	C63	1.402(4)
C58	C59	1.402(4)
C68	C69	1.394(4)
C76	C77	1.401(4)
C76	C75	1.383(5)
C72	C70	1.494(4)
C72	C77	1.403(4)
C72	C73	1.409(4)
C61	C60	1.381(5)
C61	C62	1.393(5)
C79	C77	1.492(4)
C80	C81	1.379(4)
C80	C85	1.377(4)
C70	C69	1.391(4)
C81	C82	1.389(5)
C60	C59	1.389(4)
C63	C62	1.378(4)
C82	C83	1.382(5)
C85	C84	1.388(5)
C75	C74	1.383(5)
C83	C84	1.388(6)
C73	C74	1.396(5)
C73	C78	1.503(5)
O1	C30	1.405(4)
O1	C29	1.359(3)
N2	C7	1.426(3)
N2	C15	1.477(3)
N2	C14	1.465(4)
O2	C29	1.203(4)

Atom	Atom	Length/Å
N1	C1	1.472(3)
N1	C14	1.462(4)
N1	C17	1.440(4)
C16	C21	1.403(4)
C16	C15	1.512(4)
C16	C17	1.400(4)
C7	C2	1.398(4)
C7	C6	1.401(4)
C21	C20	1.391(4)
C2	C1	1.516(4)
C2	C3	1.396(4)
C30	C31	1.376(4)
C30	C35	1.378(5)
C13	C12	1.388(4)
C13	C8	1.398(4)
C6	C5	1.382(4)
C29	C27	1.497(4)
C9	C10	1.386(4)
C9	C8	1.399(4)
C3	C4	1.397(4)
C12	C11	1.384(5)
C10	C11	1.387(4)
C4	C5	1.396(4)
C4	C8	1.484(4)
C27	C26	1.405(4)
C27	C22	1.392(4)
C26	C25	1.371(5)
C31	C32	1.394(5)
C18	C17	1.396(4)
C18	C19	1.374(4)
C22	C20	1.498(4)
C22	C23	1.408(5)
C19	C20	1.403(4)
C23	C24	1.399(5)
C23	C28	1.498(5)
C32	C33	1.383(5)
C35	C34	1.383(5)
C25	C24	1.387(5)
C34	C33	1.399(5)

Table S22: Bond Angles in ° for **SC16017**.

Atom	Atom	Atom	Angle/°
C79	O51	C80	119.6(2)
C67	N51	C51	112.1(2)
C67	N51	C64	110.9(2)
C64	N51	C51	107.6(2)
C67	C66	C65	120.3(2)
C71	C66	C67	119.0(2)
C71	C66	C65	120.7(2)
C57	N52	C65	113.5(2)
C57	N52	C64	110.2(2)
C64	N52	C65	107.3(2)
C66	C67	N51	121.5(2)
C68	C67	N51	118.6(2)
C68	C67	C66	119.9(2)
C52	C53	C54	122.0(3)
C53	C52	C57	119.6(2)
C53	C52	C51	119.7(2)

Atom	Atom	Atom	Angle/°
C57	C52	C51	120.7(2)
C52	C57	N52	121.9(2)
C56	C57	N52	119.3(2)
C56	C57	C52	118.8(2)
C66	C71	C70	121.7(2)
N51	C51	C52	111.7(2)
C53	C54	C58	121.5(2)
C53	C54	C55	117.0(2)
C55	C54	C58	121.4(2)
C55	C56	C57	121.2(2)
N52	C65	C66	112.3(2)
C63	C58	C54	121.0(3)
C59	C58	C54	121.9(3)
C59	C58	C63	117.1(3)
C67	C68	C69	120.3(3)
C75	C76	C77	119.4(3)

Atom	Atom	Atom	Angle/˚
C77	C72	C70	121.4(3)
C77	C72	C73	119.0(3)
C73	C72	C70	119.6(3)
C60	C61	C62	118.7(3)
O51	C79	C77	110.2(2)
O52	C79	O51	123.6(3)
O52	C79	C77	126.0(3)
C81	C80	O51	117.5(3)
C85	C80	O51	119.7(3)
C85	C80	C81	122.6(3)
C71	C70	C72	121.4(3)
C69	C70	C71	118.3(3)
C69	C70	C72	120.3(3)
C70	C69	C68	120.7(3)
C80	C81	C82	118.4(3)
N51	C64	N52	112.4(2)
C56	C55	C54	121.3(3)
C76	C77	C72	121.0(3)
C76	C77	C79	115.4(3)
C72	C77	C79	123.5(3)
C61	C60	C59	120.8(3)
C62	C63	C58	121.6(3)
C83	C82	C81	120.5(3)
C80	C85	C84	118.2(3)
C60	C59	C58	121.1(3)
C76	C75	C74	120.1(3)
C82	C83	C84	119.7(3)
C63	C62	C61	120.6(3)
C72	C73	C78	121.5(3)
C74	C73	C72	118.9(3)
C74	C73	C78	119.6(3)
C85	C84	C83	120.7(3)
C75	C74	C73	121.6(3)
C29	O1	C30	119.7(2)
C7	N2	C15	114.3(2)
C7	N2	C14	110.6(2)
C14	N2	C15	107.2(2)
C14	N1	C1	108.2(2)
C17	N1	C1	112.3(2)
C17	N1	C14	110.5(2)
C21	C16	C15	120.9(2)
C17	C16	C21	118.5(2)
C17	C16	C15	120.6(2)
C2	C7	N2	121.6(2)
C2	C7	C6	119.7(3)
C6	C7	N2	118.6(2)
C20	C21	C16	121.4(2)
C7	C2	C1	120.6(2)
C3	C2	C7	118.5(3)

Atom	Atom	Atom	Angle/˚
C3	C2	C1	120.9(2)
C31	C30	O1	116.0(3)
C31	C30	C35	123.0(3)
C35	C30	O1	120.8(3)
C12	C13	C8	120.7(3)
C5	C6	C7	120.8(3)
O1	C29	C27	111.5(2)
O2	C29	O1	123.7(3)
O2	C29	C27	124.8(3)
C10	C9	C8	120.2(3)
N1	C1	C2	112.3(2)
C2	C3	C4	122.1(3)
C11	C12	C13	120.0(3)
N2	C15	C16	112.0(2)
C9	C10	C11	120.5(3)
C3	C4	C8	121.3(3)
C5	C4	C3	118.3(3)
C5	C4	C8	120.4(3)
C26	C27	C29	115.4(3)
C22	C27	C29	123.7(3)
C22	C27	C26	120.9(3)
C25	C26	C27	119.7(3)
N1	C14	N2	111.8(2)
C30	C31	C32	118.2(3)
C19	C18	C17	120.9(3)
C6	C5	C4	120.4(3)
C16	C17	N1	121.5(2)
C18	C17	N1	118.5(2)
C18	C17	C16	120.0(3)
C12	C11	C10	119.8(3)
C13	C8	C9	118.7(3)
C13	C8	C4	121.3(3)
C9	C8	C4	120.0(3)
C27	C22	C20	122.8(3)
C27	C22	C23	119.3(3)
C23	C22	C20	117.9(3)
C18	C19	C20	120.1(3)
C21	C20	C22	120.8(3)
C21	C20	C19	119.0(3)
C19	C20	C22	120.1(3)
C22	C23	C28	121.1(3)
C24	C23	C22	118.7(3)
C24	C23	C28	120.2(3)
C33	C32	C31	120.5(3)
C30	C35	C34	118.2(3)
C26	C25	C24	120.0(3)
C35	C34	C33	120.6(3)
C32	C33	C34	119.6(3)
C25	C24	C23	121.4(3)

Table S23: Torsion Angles in ˚ for **SC16017**.

Atom	Atom	Atom	Atom	Angle/˚
O51	C79	C77	C76	137.0(3)
O51	C79	C77	C72	-39.1(4)
O51	C80	C81	C82	-175.3(3)
O51	C80	C85	C84	174.0(3)
O52	C79	C77	C76	-39.6(4)
O52	C79	C77	C72	144.3(3)

Atom	Atom	Atom	Atom	Angle/°
N51	C67	C68	C69	178.3(2)
C66	C67	C68	C69	-1.3(4)
C66	C71	C70	C72	-178.9(3)
C66	C71	C70	C69	0.5(4)
N52	C57	C56	C55	177.5(2)
C67	N51	C51	C52	77.1(3)
C67	N51	C64	N52	-52.7(3)
C67	C66	C71	C70	-3.1(4)
C67	C66	C65	N52	12.0(3)
C67	C68	C69	C70	-1.4(4)
C53	C52	C57	N52	-176.7(2)
C53	C52	C57	C56	1.0(4)
C53	C52	C51	N51	-167.8(2)
C53	C54	C58	C63	165.0(3)
C53	C54	C58	C59	-13.2(4)
C53	C54	C55	C56	0.1(4)
C52	C53	C54	C58	-175.5(2)
C52	C53	C54	C55	0.6(4)
C52	C57	C56	C55	-0.3(4)
C57	N52	C65	C66	75.6(3)
C57	N52	C64	N51	-54.0(3)
C57	C52	C51	N51	11.0(3)
C57	C56	C55	C54	-0.3(4)
C71	C66	C67	N51	-176.1(2)
C71	C66	C67	C68	3.5(4)
C71	C66	C65	N52	-166.9(2)
C71	C70	C69	C68	1.7(4)
C51	N51	C67	C66	-105.9(3)
C51	N51	C67	C68	74.5(3)
C51	N51	C64	N52	70.3(3)
C51	C52	C57	N52	4.5(4)
C51	C52	C57	C56	-177.8(2)
C54	C53	C52	C57	-1.2(4)
C54	C53	C52	C51	177.6(2)
C54	C58	C63	C62	-179.1(3)
C54	C58	C59	C60	178.6(3)
C65	C66	C67	N51	5.0(4)
C65	C66	C67	C68	-175.5(2)
C65	C66	C71	C70	175.8(2)
C65	N52	C57	C52	-104.4(3)
C65	N52	C57	C56	77.9(3)
C65	N52	C64	N51	70.0(3)
C58	C54	C55	C56	176.3(2)
C58	C63	C62	C61	1.3(5)
C76	C75	C74	C73	0.7(5)
C72	C70	C69	C68	-178.9(3)
C72	C73	C74	C75	0.6(5)
C61	C60	C59	C58	-0.2(5)
C79	O51	C80	C81	-112.7(3)
C79	O51	C80	C85	72.5(4)
C80	O51	C79	O52	1.4(4)
C80	O51	C79	C77	-175.3(2)
C80	C81	C82	C83	1.5(5)
C80	C85	C84	C83	0.9(5)
C70	C72	C77	C76	176.8(3)
C70	C72	C77	C79	-7.4(4)
C70	C72	C73	C74	-177.8(3)
C70	C72	C73	C78	1.1(4)
C81	C80	C85	C84	-0.6(5)
C81	C82	C83	C84	-1.2(5)
C64	N51	C67	C66	14.5(3)
C64	N51	C67	C68	-165.1(2)

Atom	Atom	Atom	Atom	Angle/°
C64	N51	C51	C52	-45.2(3)
C64	N52	C57	C52	16.0(3)
C64	N52	C57	C56	-161.7(2)
C64	N52	C65	C66	-46.4(3)
C55	C54	C58	C63	-11.1(4)
C55	C54	C58	C59	170.8(3)
C77	C76	C75	C74	-1.7(5)
C77	C72	C70	C71	106.6(3)
C77	C72	C70	C69	-72.8(4)
C77	C72	C73	C74	-0.9(4)
C77	C72	C73	C78	178.0(3)
C60	C61	C62	C63	-1.1(5)
C63	C58	C59	C60	0.3(5)
C82	C83	C84	C85	0.0(6)
C85	C80	C81	C82	-0.6(5)
C59	C58	C63	C62	-0.9(5)
C75	C76	C77	C72	1.4(4)
C75	C76	C77	C79	-174.8(3)
C62	C61	C60	C59	0.6(5)
C73	C72	C70	C71	-76.6(4)
C73	C72	C70	C69	104.1(3)
C73	C72	C77	C76	-0.1(4)
C73	C72	C77	C79	175.7(3)
C78	C73	C74	C75	-178.3(3)
O1	C30	C31	C32	-176.0(3)
O1	C30	C35	C34	176.3(3)
O1	C29	C27	C26	140.4(3)
O1	C29	C27	C22	-40.3(4)
N2	C7	C2	C1	8.4(4)
N2	C7	C2	C3	-171.9(2)
N2	C7	C6	C5	175.6(2)
O2	C29	C27	C26	-38.1(4)
O2	C29	C27	C22	141.2(3)
C16	C21	C20	C22	179.9(3)
C16	C21	C20	C19	0.0(4)
C7	N2	C15	C16	76.8(3)
C7	N2	C14	N1	-54.4(3)
C7	C2	C1	N1	6.2(4)
C7	C2	C3	C4	-4.0(4)
C7	C6	C5	C4	-3.4(4)
C21	C16	C15	N2	-166.9(2)
C21	C16	C17	N1	-176.6(2)
C21	C16	C17	C18	2.3(4)
C2	C7	C6	C5	-0.8(4)
C2	C3	C4	C5	0.0(4)
C2	C3	C4	C8	178.3(3)
C30	O1	C29	O2	1.2(4)
C30	O1	C29	C27	-177.3(2)
C30	C31	C32	C33	0.0(5)
C30	C35	C34	C33	-0.9(6)
C13	C12	C11	C10	-0.7(5)
C6	C7	C2	C1	-175.4(2)
C6	C7	C2	C3	4.3(4)
C29	O1	C30	C31	-123.8(3)
C29	O1	C30	C35	61.0(4)
C29	C27	C26	C25	178.4(3)
C29	C27	C22	C20	1.2(4)
C29	C27	C22	C23	-176.2(3)
C9	C10	C11	C12	0.5(4)
C1	N1	C14	N2	69.3(3)
C1	N1	C17	C16	-105.6(3)
C1	N1	C17	C18	75.4(3)

Atom	Atom	Atom	Atom	Angle/˚
C1	C2	C3	C4	175.8(3)
C3	C2	C1	N1	-173.5(2)
C3	C4	C5	C6	3.7(4)
C3	C4	C8	C13	130.6(3)
C3	C4	C8	C9	-49.9(4)
C12	C13	C8	C9	0.9(4)
C12	C13	C8	C4	-179.6(3)
C15	N2	C7	C2	-106.1(3)
C15	N2	C7	C6	77.6(3)
C15	N2	C14	N1	70.9(3)
C15	C16	C21	C20	176.0(2)
C15	C16	C17	N1	5.3(4)
C15	C16	C17	C18	-175.8(2)
C10	C9	C8	C13	-1.0(4)
C10	C9	C8	C4	179.4(3)
C27	C26	C25	C24	-1.5(5)
C27	C22	C20	C21	101.7(3)
C27	C22	C20	C19	-78.5(4)
C27	C22	C23	C24	-2.8(5)
C27	C22	C23	C28	176.7(3)
C26	C27	C22	C20	-179.5(3)
C26	C27	C22	C23	3.1(4)
C26	C25	C24	C23	1.8(6)
C14	N2	C7	C2	15.0(3)
C14	N2	C7	C6	-161.2(2)
C14	N2	C15	C16	-46.2(3)
C14	N1	C1	C2	-42.3(3)
C14	N1	C17	C16	15.3(3)
C14	N1	C17	C18	-163.7(2)
C31	C30	C35	C34	1.5(5)
C31	C32	C33	C34	0.6(6)
C18	C19	C20	C21	2.0(4)
C18	C19	C20	C22	-177.8(3)
C5	C4	C8	C13	-51.1(4)
C5	C4	C8	C9	128.4(3)
C17	N1	C1	C2	79.9(3)
C17	N1	C14	N2	-54.0(3)
C17	C16	C21	C20	-2.2(4)
C17	C16	C15	N2	11.2(3)
C17	C18	C19	C20	-1.8(5)
C8	C13	C12	C11	0.0(5)
C8	C9	C10	C11	0.3(4)
C8	C4	C5	C6	-174.6(2)
C22	C27	C26	C25	-1.0(5)
C22	C23	C24	C25	0.4(6)
C19	C18	C17	N1	178.6(3)
C19	C18	C17	C16	-0.4(4)
C20	C22	C23	C24	179.7(3)
C20	C22	C23	C28	-0.8(5)
C23	C22	C20	C21	-80.8(4)
C23	C22	C20	C19	99.0(4)
C35	C30	C31	C32	-1.0(5)
C35	C34	C33	C32	-0.1(6)
C28	C23	C24	C25	-179.1(4)

Table S24: Hydrogen Fractional Atomic Coordinates (×10^4) and Equivalent Isotropic Displacement Parameters (Å^2×10^3) for **SC16017**. U_{eq} is defined as 1/3 of the trace of the orthogonalised U_{ij}.

Atom	x	y	z	U_{eq}
H53	4241.55	10972.89	2181.37	28
H71	4009.97	4901.91	986.66	27
H51A	4043.28	11904.17	1654.41	29
H51B	4456.24	13076.56	1736.72	29
H56	5002.06	5586.53	1935.57	27
H65A	4601.79	5490.67	1410.48	26
H65B	4760.03	6694.05	1171.94	26
H68	3644.84	11832.06	1152.76	32
H76	2477.13	2659.46	764.16	39
H61	4071.92	7610.35	3270.09	41
H69	3183.97	9456.34	897.66	33
H81	3610.36	7607.02	1668.03	38
H64A	4957.68	11530	1533.81	30
H64B	4836.63	10512.23	1224.51	30
H55	4843.84	5460.39	2366.03	30
H60	3980.91	10781.27	3012.62	42
H63	4570.58	4990.72	2687.79	36
H82	3734.79	7603.67	2162.25	47
H85	3128.48	1729.13	1647.19	44
H59	4179.25	11075.4	2594.4	38
H75	2500.35	1873.56	301.07	46
H83	3578.15	4630.57	2398.53	52
H62	4380.71	4735.22	3106.95	44
H84	3274.73	1700.29	2141.98	52
H74	2978.24	3375.73	111.23	47
H78A	3554.01	7550.44	346.06	65
H78B	3500.05	5616.76	126.33	65
H78C	3817.18	5459.18	420.85	65
H21	1313.39	10177.5	1570.34	27
H13	767.28	9639.22	-90.84	35
H6	200.28	10275.57	679.79	29
H9	314.03	15604.99	-207.88	34
H1A	1146.54	16653.81	817.94	31
H1B	740.27	17891.6	756.48	31
H3	809.44	15639.35	286.62	31
H12	779.26	9491.49	-565.78	37
H15A	673.37	10512.97	1177.1	27
H15B	546.44	11841.39	1419.77	27
H10	330.3	15437.76	-681.4	34
H26	2796.4	7760.07	1726.97	43
H14A	278.52	16466.73	1021.2	32
H14B	451.31	15623.2	1335.95	32
H31	1578.47	12540.66	829.51	40
H18	1602.97	17011.33	1303.31	34
H5	272.23	9905.32	221.9	32
H11	566.32	12402.17	-860.56	36
H19	2097.88	14835.88	1549.45	36
H32	1426.18	12485.56	333.07	50
H35	2186.41	7046.07	847.62	47
H25	2848.02	7522.02	2209.26	52
H34	2041.15	7026.61	353.94	55
H33	1660.07	9752.43	95.9	54
H24	2437.09	9512.09	2415.49	54
H28A	1846.23	13607.98	2128.3	80
H28B	1952.35	12068.4	2397.22	80
H28C	1590.03	11526.37	2140.3	80

Citations

O.V. Dolomanov and L.J. Bourhis and R.J. Gildea and J.A.K. Howard and H. Puschmann, Olex2: A complete structure solution, refinement and analysis program, *J. Appl. Cryst.*, (2009), **42**, 339-341.

Sheldrick, G.M., A short history of ShelX, *Acta Cryst.*, (2008), **A64**, 339-341.

Sheldrick, G.M., Crystal structure refinement with ShelXL, *Acta Cryst.*, (2015), **C27**, 3-8.

```
#==============================================================================
# PLATON/CHECK-(150616) versus check.def version of 160610 for Entry: sc16017
# Data: SC16017.cif - Type: CIF                  Bond Precision   C-C = 0.0043 A
# Refl: SC16017.fcf - Type: LIST4                                   Temp = 120 K
# X-ray CuKa              R(int) = 0.102,  wR2/R(int) =  2.2,  Nref/Npar =  13.7
# Cell 35.4860(15)   6.2732(2)   48.865(2)           90   104.050(4)          90
# Wavelength 1.54184    Volume Reported    10552.4(8)   Calculated    10552.5(7)
# SpaceGroup from Symmetry I 2/a      Hall: -I 2ya                monoclinic
#                 Reported I 1 2/a 1        -I 2ya                monoclinic
# MoietyFormula C35 H28 N2 O2
#      Reported C35 H28 N2 O2
#    SumFormula C35 H28 N2 O2
#      Reported C35 H28 N2 O2
# Mr        =    508.59[Calc],    508.59[Rep]
# Dx,gcm-3  =     1.281[Calc],     1.281[Rep]
# Z         =        16[Calc],        16[Rep]
# Mu (mm-1) =     0.625[Calc],     0.625[Rep]
# F000      =    4288.0[Calc],    4288.0[Rep]  or F000' =   4300.05[Calc]
# Reported   T Limits: Tmin=0.750              Tmax=1.000  AbsCorr = GAUSSIAN
# Calculated T Limits: Tmin=0.957 Tmin'=0.738  Tmax=0.980
# Reported   Hmax= 42, Kmax=  7, Lmax= 58, Nref=   9692        , Th(max)= 68.246
# Obs in FCF Hmax= 42, Kmax=  7, Lmax= 58, Nref=   9692[  9692], Th(max)= 68.246
# Calculated Hmax= 42, Kmax=  7, Lmax= 58, Nref=   9695        , Ratio  =  1.000
# Reported   Rho(min) = -0.38, Rho(max) =  0.48 e/Ang**3 (From CIF)
# Calculated Rho(min) = -0.38, Rho(max) =  0.47 e/Ang**3 (From CIF+FCF data)
# w=1/[sigma**2(Fo**2)+(0.1366P)**2+  5.2404P], P=(Fo**2+2*Fc**2)/3
# R= 0.0774(  7229), wR2= 0.2251(  9692), S = 1.037      (From CIF+FCF data)
# R= 0.0774(  7229), wR2= 0.2251(  9692), S = 1.037      (From FCF data only)
# R= 0.0774(  7229), wR2= 0.2251(  9692), S = 1.037, Npar=  705
#==============================================================================
For Documentation: http://http://www.platonsoft.nl/CIF-VALIDATION.pdf
#==============================================================================

#==============================================================================
>>> The Following Improvement and Query ALERTS were generated - (Acta-Mode) <<<
#==============================================================================
Format: alert-number_ALERT_alert-type_alert-level text

340_ALERT_3_C Low Bond Precision on  C-C Bonds ..............    0.00435 Ang.
790_ALERT_4_C Centre of Gravity not Within Unit Cell: Resd.  #          1 Note
C35 H28 N2 O2
906_ALERT_3_C Large K value in the Analysis of Variance ......      6.901 Check
#==============================================================================
072_ALERT_2_G SHELXL First  Parameter in WGHT  Unusually Large       0.14 Report
083_ALERT_2_G SHELXL Second Parameter in WGHT  Unusually Large       5.24 Why ?
910_ALERT_3_G Missing # of FCF Reflection(s) Below Theta(Min)           3 Note
978_ALERT_2_G Number C-C Bonds with Positive Residual Density           1 Note
#==============================================================================

ALERT_Level and ALERT_Type Summary
==================================
3 ALERT_Level_C = Check. Ensure it is Not caused by an Omission or Oversight
4 ALERT_Level_G = General Info/Check that it is not Something Unexpected

3 ALERT_Type_2 Indicator that the Structure Model may be Wrong or Deficient.
3 ALERT_Type_3 Indicator that the Structure Quality may be Low.
1 ALERT_Type_4 Improvement, Methodology, Query or Suggestion.
#==============================================================================

0 Missing  Experimental  Info Issue(s) (Out of  62 Tests) -  100 % Satisfied
0 Experimental  Data  Related Issue(s) (Out of  28 Tests) -  100 % Satisfied
3 Structural  Model   Related Issue(s) (Out of 126 Tests) -   98 % Satisfied
4 Unresolved or to be Checked Issue(s) (Out of 247 Tests) -   98 % Satisfied

#==============================================================================
```

Compound (±)-**1F** CCDC Deposition Number 2244212

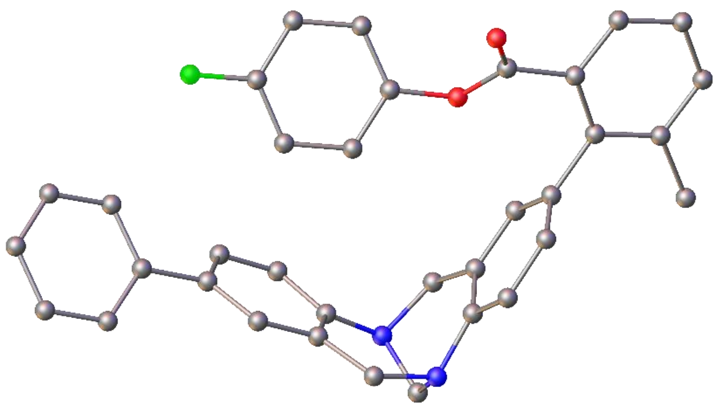

Experimental. Single colourless block-shaped crystals of (**SC16016**) were recrystallised from a mixture of methanol and chloroform by slow evaporation. A suitable crystal (0.57×0.35×0.17) mm^3 was selected and mounted on a MITIGEN holder in Paratone oil on a Rigaku Oxford Diffraction SuperNova diffractometer. The crystal was kept at T = 120.0 K during data collection. Using **Olex2** (Dolomanov et al., 2009), the structure was solved with the **ShelXS** (Sheldrick, 2008) structure solution program, using the Direct Methods solution method. The model was refined with version 2016/6 of **ShelXL** (Sheldrick, 2015) using Least Squares minimisation.

Crystal Data. $C_{35}H_{27}FN_2O_2$, M_r = 526.58, monoclinic, P2$_1$/n (No. 14), a = 17.4782(5) Å, b = 6.38739(19) Å, c = 24.9672(9) Å, β = 107.173(4)°, α = γ = 90°, V = 2663.07(16) Å^3, T = 120.0 K, Z = 4, Z' = 1, μ(Mo K$_\alpha$) = 0.086, 54509 reflections measured, 8067 unique (R_{int} = 0.0369) which were used in all calculations. The final wR_2 was 0.1294 (all data) and R_1 was 0.0533 (I > 2(I)).

**Compound**	**SC16016**
Formula	$C_{35}H_{27}FN_2O_2$
$D_{calc.}$/ g cm^{-3}	1.313
μ/mm^{-1}	0.086
Formula Weight	526.58
Colour	colourless
Shape	block
Size/mm^3	0.57×0.35×0.17
T/K	120.0
Crystal System	monoclinic
Space Group	P2$_1$/n
a/Å	17.4782(5)
b/Å	6.38739(19)
c/Å	24.9672(9)
α/°	90
β/°	107.173(4)
γ/°	90
V/Å^3	2663.07(16)
Z	4
Z'	1
Wavelength/Å	0.71073
Radiation type	Mo K$_\alpha$
Θ_{min}/°	3.146
Θ_{max}/°	31.200
Measured Refl.	54509
Independent Refl.	8067
Reflections Used	7020
R_{int}	0.0369
Parameters	469
Restraints	0
Largest Peak	0.343
Deepest Hole	-0.310
GooF	1.109
wR_2 (all data)	0.1294
wR_2	0.1245
R_1 (all data)	0.0626
R_1	0.0533

Structure Quality Indicators

**Reflections:**	d min (Mo) 0.69	I/σ 23.0	Rint 3.69%	complete at 2θ=56° 94%
**Refinement:**	Shift 0.000	Max Peak 0.3	Min Peak -0.3	GooF 1.109

A colourless block-shaped crystal with dimensions 0.57×0.35×0.17 mm^3 was mounted on a MITIGEN holder in Paratone oil.X-ray diffraction data were collected using a Rigaku Oxford Diffraction SuperNova diffractometer equipped with a Oxford Cryosystems Cryostream 700+ low-temperature device, operating at T = 120.0 K.

Data were measured using ω scans scans of 1.0 ° per frame for 15.0 s using Mo K_α radiation (micro-focus sealed X-ray tube, 50 kV, 0.8 mA). The total number of runs and images was based on the strategy calculation from the program CrysAlisPro (Agilent).The maximum resolution achieved was Θ = 31.200. °

Cell parameters were retrieved using the CrysAlisPro (Agilent) software and refined using CrysAlisPro (Agilent) on 16956 reflections, 31 % of the observed reflections. Data reduction was performed using the CrysAlisPro (Agilent) software which corrects for Lorentz polarisation. The final completeness is 99.79 out to 31.200 in Θ. The absorption coefficient μ of this material is 0.086 at this wavelength (λ = 0.71073) and the minimum and maximum transmissions are 0.503 and 1.000.

The structure was solved in the space group P2$_1$/n (# 14) by Direct Methods using the **ShelXS** (Sheldrick, 2008) structure solution program and refined by Least Squares using version 2016/6 of **ShelXL** (Sheldrick, 2015). All non-hydrogen atoms were refined anisotropically. Hydrogen atom positions were calculated geometrically and refined using the riding model.

_refine_special_details: All H atoms were identified from a difference Fourier map and freely refined.

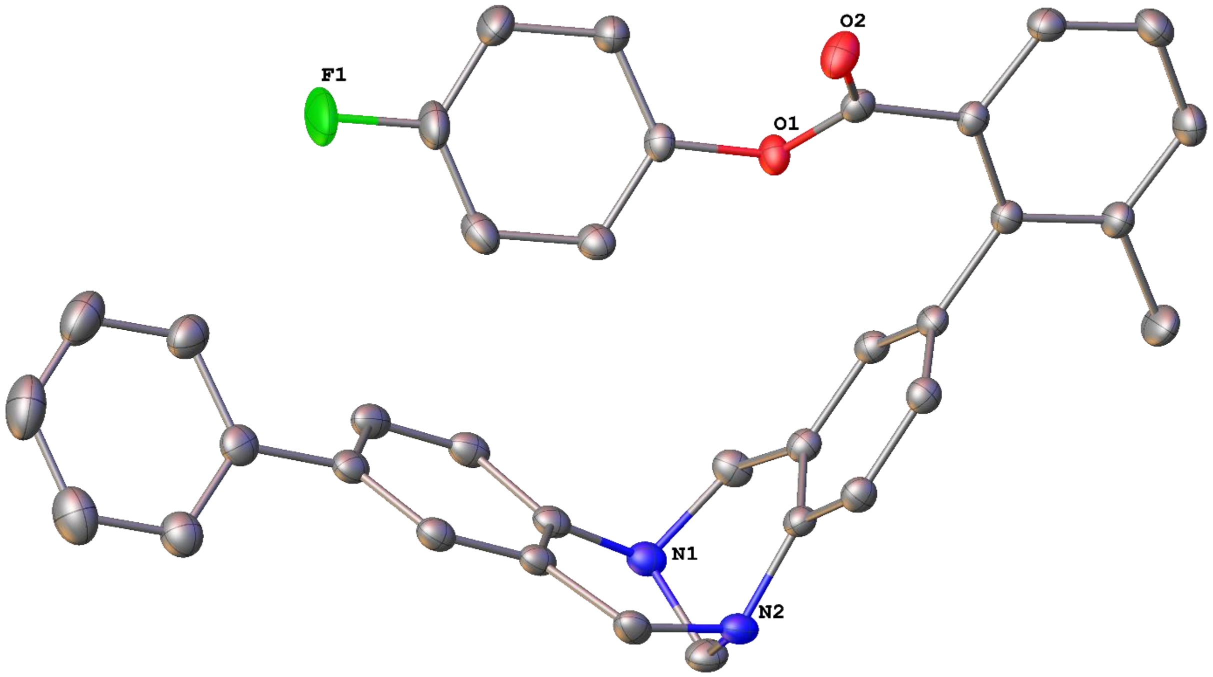

Figure S5: The molecular structure of SC16016. Displacement ellipsoids are at the 50% probability level and H atoms are not shown.

Reflection Statistics

Total reflections (after filtering)	57337	Unique reflections	8067
Completeness	0.935	Mean I/σ	23.0
hkl_{max} collected	(25, 8, 35)	hkl_{min} collected	(-25, -8, -36)
hkl_{max} used	(24, 8, 36)	hkl_{min} used	(-25, 0, 0)
Lim d_{max} collected	100.0	Lim d_{min} collected	0.36
d_{max} used	6.48	d_{min} used	0.69
Friedel pairs	12742	Friedel pairs merged	1
Inconsistent equivalents	1	R_{int}	0.0369
R_{sigma}	0.0272	Intensity transformed	0
Omitted reflections	0	Omitted by user (OMIT hkl)	5
Multiplicity	(11292, 9600, 4324, 1891, 765, 284, 69, 27, 9)	Maximum multiplicity	22
Removed systematic absences	2823	Filtered off (Shel/OMIT)	0

Table S25: Fractional Atomic Coordinates (×10⁴) and Equivalent Isotropic Displacement Parameters (Å²×10³) for **SC16016.** U_{eq} is defined as 1/3 of the trace of the orthogonalised U_{ij}.

Atom	x	y	z	U_{eq}
F1	778.7(7)	4504(2)	4236.4(4)	46.8(3)
O1	1144.1(5)	4301.4(15)	2137.6(4)	19.02(19)
O2	2495.6(6)	4425.0(17)	2434.9(4)	25.3(2)
N1	-1948.6(6)	360.4(18)	1416.1(5)	20.1(2)
N2	-988.9(6)	-2419.6(17)	1460.8(5)	19.3(2)
C1	-872.5(8)	-2863(2)	2059.7(6)	20.2(2)
C2	-1212.1(7)	-1134.0(19)	2339.8(5)	18.2(2)
C3	-1676.6(7)	464(2)	2016.8(5)	18.7(2)
C4	-1913.0(7)	2153(2)	2286.1(6)	20.9(3)
C5	-1730.6(7)	2202(2)	2862.9(6)	21.9(3)
C6	-1324.0(7)	533(2)	3190.8(6)	20.5(2)
C7	-1059.6(7)	-1103(2)	2919.8(6)	19.9(2)
C8	-1206.6(7)	505(2)	3804.6(6)	23.3(3)
C9	-1343.9(9)	-1320(3)	4070.2(6)	28.5(3)
C10	-1267.9(10)	-1328(3)	4638.7(7)	38.3(4)
C11	-1043.6(11)	480(3)	4953.2(7)	42.8(4)
C12	-903.2(10)	2296(3)	4696.6(7)	39.6(4)
C13	-987.0(9)	2320(3)	4126.5(7)	29.6(3)
C14	-1819.8(8)	-1734(2)	1220.0(6)	22.6(3)
C15	-1545.0(7)	1866(2)	1141.2(6)	20.0(2)
C16	-694.5(7)	1172(2)	1194.5(5)	16.8(2)
C17	-442.4(7)	-855.5(19)	1376.8(5)	16.2(2)
C18	355.4(7)	-1420(2)	1466.1(5)	18.2(2)
C19	896.9(7)	-8(2)	1362.5(5)	18.2(2)
C20	647.4(7)	1988.6(19)	1158.9(5)	16.0(2)
C21	-146.8(7)	2559(2)	1079.8(5)	16.7(2)
C22	1233.0(7)	3457.0(19)	1028.7(5)	16.1(2)
C23	1234.8(8)	3684(2)	468.4(5)	20.3(2)
C24	1802.4(9)	4978(2)	346.1(6)	25.2(3)
C25	2370.9(8)	6038(2)	764.4(6)	25.3(3)
C26	2384.5(8)	5792(2)	1317.3(6)	21.1(3)
C27	1818.9(7)	4513.5(19)	1449.7(5)	16.4(2)
C28	644.7(10)	2508(3)	3.1(6)	29.4(3)
C29	1880.4(7)	4384.1(19)	2059.2(5)	17.0(2)
C30	1087.6(7)	4354(2)	2684.8(5)	17.3(2)
C31	1388.1(8)	6036(2)	3031.7(6)	23.1(3)
C32	1282.1(9)	6091(3)	3561.9(6)	29.0(3)
C33	872.1(9)	4470(3)	3714.2(6)	28.3(3)
C34	557.1(8)	2811(2)	3369.5(6)	25.4(3)
C35	666.1(7)	2754(2)	2840.3(5)	20.6(2)

Table S26: Anisotropic Displacement Parameters (×10[4]) **SC16016.** The anisotropic displacement factor exponent takes the form: $-2\pi^2[h^2a^{*2} \times U_{11} + ... + 2hka^* \times b^* \times U_{12}]$

Atom	U_{11}	U_{22}	U_{33}	U_{23}	U_{13}	U_{12}
F1	52.9(6)	73.5(8)	20.6(4)	-9.0(5)	21.1(4)	-20.7(6)
O1	16.6(4)	27.2(5)	12.8(4)	0.5(3)	3.6(3)	-0.4(3)
O2	17.7(4)	36.9(6)	18.5(4)	-5.4(4)	1.1(3)	3.9(4)
N1	15.6(5)	19.2(5)	25.3(5)	-0.7(4)	5.5(4)	-1.1(4)
N2	17.1(5)	15.7(5)	25.6(5)	-2.5(4)	6.9(4)	-2.6(4)
C1	21.6(6)	14.0(6)	27.2(6)	1.0(5)	10.8(5)	0.3(5)
C2	14.7(5)	15.1(5)	26.3(6)	-1.0(5)	8.5(5)	-1.4(4)
C3	13.6(5)	18.0(6)	25.3(6)	-0.2(5)	6.8(4)	-2.5(4)
C4	15.3(5)	17.1(6)	31.2(7)	1.5(5)	8.4(5)	1.0(5)
C5	17.1(5)	18.3(6)	33.2(7)	-3.4(5)	11.9(5)	-0.1(5)
C6	15.6(5)	20.5(6)	27.1(6)	-2.8(5)	9.2(5)	-3.3(5)
C7	16.7(5)	17.0(6)	27.1(6)	1.1(5)	8.2(5)	-0.9(5)
C8	15.9(5)	27.1(7)	28.5(7)	-2.2(5)	8.8(5)	0.9(5)
C9	24.7(6)	32.3(8)	28.7(7)	0.9(6)	8.2(6)	-0.3(6)
C10	35.6(8)	48.8(10)	32.3(8)	7.9(7)	12.6(7)	2.8(8)
C11	39.1(9)	65.1(13)	26.5(8)	-3.7(8)	13.4(7)	5.3(9)
C12	35.4(8)	49.7(11)	35.6(9)	-15.8(8)	13.3(7)	1.2(8)
C13	25.3(7)	32.0(8)	33.5(8)	-8.5(6)	12.0(6)	-0.4(6)
C14	17.2(5)	21.5(6)	27.8(7)	-4.8(5)	4.8(5)	-4.2(5)
C15	15.8(5)	19.3(6)	24.4(6)	2.6(5)	4.9(5)	2.1(5)
C16	15.5(5)	17.6(6)	16.1(5)	-1.6(4)	2.9(4)	0.7(4)
C17	16.6(5)	15.8(6)	16.1(5)	-2.8(4)	4.6(4)	-1.4(4)
C18	18.9(5)	15.8(6)	19.9(6)	0.1(4)	5.8(5)	2.4(4)
C19	15.6(5)	19.4(6)	19.2(6)	-0.8(4)	4.8(4)	2.0(4)
C20	16.5(5)	17.7(6)	12.9(5)	-1.8(4)	3.2(4)	-1.9(4)
C21	17.8(5)	15.8(6)	15.2(5)	-0.3(4)	2.8(4)	0.1(4)
C22	16.3(5)	16.3(5)	15.8(5)	-0.3(4)	5.0(4)	0.7(4)
C23	23.4(6)	21.8(6)	15.7(6)	-1.6(5)	5.9(5)	-2.4(5)
C24	29.7(7)	29.1(7)	19.1(6)	0.7(5)	10.9(5)	-3.6(6)
C25	24.9(6)	26.8(7)	26.6(7)	1.0(5)	11.1(5)	-6.7(5)
C26	18.1(5)	21.7(6)	23.2(6)	-3.4(5)	5.7(5)	-3.9(5)
C27	15.9(5)	17.6(6)	15.8(5)	-0.8(4)	4.7(4)	1.8(4)
C28	36.8(8)	35.3(8)	15.6(6)	-6.0(6)	6.9(6)	-10.7(7)
C29	17.2(5)	15.6(5)	17.8(5)	-2.0(4)	4.4(4)	1.6(4)
C30	16.3(5)	22.3(6)	12.8(5)	1.2(4)	3.6(4)	1.3(4)
C31	25.4(6)	24.0(7)	21.3(6)	-2.6(5)	9.1(5)	-4.5(5)
C32	31.9(7)	34.1(8)	22.6(7)	-9.6(6)	10.8(6)	-8.9(6)
C33	26.3(6)	44.1(9)	17.0(6)	-2.3(6)	10.0(5)	-5.5(6)
C34	21.5(6)	33.0(8)	22.6(6)	3.9(5)	7.8(5)	-6.0(5)
C35	17.5(5)	23.1(6)	19.7(6)	-0.2(5)	3.3(5)	-2.8(5)

Table S27: Bond Lengths in Å for **SC16016**.

Atom	Atom	Length/Å
F1	C33	1.3609(15)
O1	C29	1.3586(14)
O1	C30	1.3991(14)
O2	C29	1.2004(15)
N1	C3	1.4348(17)
N1	C14	1.4649(18)
N1	C15	1.4762(17)
N2	C1	1.4757(17)
N2	C14	1.4651(17)
N2	C17	1.4398(16)
C1	C2	1.5184(17)
C2	C3	1.4017(18)
C2	C7	1.3931(18)
C3	C4	1.3966(18)

Atom	Atom	Length/Å
C4	C5	1.380(2)
C5	C6	1.4029(19)
C6	C7	1.3958(18)
C6	C8	1.4850(19)
C8	C9	1.397(2)
C8	C13	1.398(2)
C9	C10	1.386(2)
C10	C11	1.386(3)
C11	C12	1.382(3)
C12	C13	1.387(2)
C15	C16	1.5190(17)
C16	C17	1.4001(17)
C16	C21	1.3947(17)
C17	C18	1.3929(16)

Atom	Atom	Length/Å
C18	C19	1.3858(17)
C19	C20	1.3944(17)
C20	C21	1.3916(16)
C20	C22	1.4934(16)
C22	C23	1.4072(16)
C22	C27	1.4048(17)
C23	C24	1.3924(18)
C23	C28	1.5066(19)
C24	C25	1.387(2)

Atom	Atom	Length/Å
C25	C26	1.3826(19)
C26	C27	1.3950(17)
C27	C29	1.4959(16)
C30	C31	1.3828(18)
C30	C35	1.3810(18)
C31	C32	1.3902(19)
C32	C33	1.375(2)
C33	C34	1.374(2)
C34	C35	1.3900(18)

Table S28: Bond Angles in ° for **SC16016**.

Atom	Atom	Atom	Angle/°
C29	O1	C30	118.86(9)
C3	N1	C14	110.81(11)
C3	N1	C15	113.56(10)
C14	N1	C15	107.33(10)
C14	N2	C1	107.25(10)
C17	N2	C1	112.45(10)
C17	N2	C14	110.66(10)
N2	C1	C2	111.81(10)
C3	C2	C1	120.22(12)
C7	C2	C1	120.54(11)
C7	C2	C3	119.24(11)
C2	C3	N1	121.65(11)
C4	C3	N1	119.02(11)
C4	C3	C2	119.27(12)
C5	C4	C3	120.66(12)
C4	C5	C6	120.84(12)
C5	C6	C8	120.21(12)
C7	C6	C5	117.99(12)
C7	C6	C8	121.77(12)
C2	C7	C6	121.68(12)
C9	C8	C6	120.62(13)
C9	C8	C13	118.40(14)
C13	C8	C6	120.93(13)
C10	C9	C8	120.75(16)
C9	C10	C11	120.17(17)
C12	C11	C10	119.74(16)
C11	C12	C13	120.37(17)
C12	C13	C8	120.55(16)
N1	C14	N2	111.54(10)
N1	C15	C16	111.04(10)
C17	C16	C15	120.27(11)
C21	C16	C15	120.67(11)
C21	C16	C17	119.03(11)
C16	C17	N2	122.05(11)

Atom	Atom	Atom	Angle/°
C18	C17	N2	118.23(11)
C18	C17	C16	119.70(11)
C19	C18	C17	120.56(12)
C18	C19	C20	120.38(11)
C19	C20	C22	119.54(10)
C21	C20	C19	118.88(11)
C21	C20	C22	121.58(11)
C20	C21	C16	121.38(11)
C23	C22	C20	118.96(11)
C27	C22	C20	122.26(10)
C27	C22	C23	118.64(11)
C22	C23	C28	120.87(12)
C24	C23	C22	119.34(12)
C24	C23	C28	119.77(12)
C25	C24	C23	121.53(12)
C26	C25	C24	119.58(12)
C25	C26	C27	119.92(12)
C22	C27	C29	123.53(11)
C26	C27	C22	120.98(11)
C26	C27	C29	115.49(11)
O1	C29	C27	111.22(10)
O2	C29	O1	123.81(11)
O2	C29	C27	124.92(11)
C31	C30	O1	120.47(11)
C35	C30	O1	117.00(11)
C35	C30	C31	122.32(12)
C30	C31	C32	118.90(13)
C33	C32	C31	118.11(13)
F1	C33	C32	118.20(13)
F1	C33	C34	118.24(13)
C34	C33	C32	123.56(13)
C33	C34	C35	118.28(13)
C30	C35	C34	118.81(12)

Table S29: Torsion Angles in ° for **SC16016**.

Atom	Atom	Atom	Atom	Angle/°
F1	C33	C34	C35	-178.73(13)
O1	C30	C31	C32	176.47(12)
O1	C30	C35	C34	-176.41(11)
N1	C3	C4	C5	173.49(11)

Atom	Atom	Atom	Atom	Angle/°
N1	C15	C16	C17	12.49(16)
N1	C15	C16	C21	-165.52(11)
N2	C1	C2	C3	8.82(16)
N2	C1	C2	C7	-170.50(11)
N2	C17	C18	C19	176.55(11)
C1	N2	C14	N1	71.39(13)
C1	N2	C17	C16	-107.86(13)
C1	N2	C17	C18	74.01(14)
C1	C2	C3	N1	9.70(17)
C1	C2	C3	C4	-173.38(11)
C1	C2	C7	C6	176.11(11)
C2	C3	C4	C5	-3.52(18)
C3	N1	C14	N2	-52.60(14)
C3	N1	C15	C16	74.04(13)
C3	C2	C7	C6	-3.21(18)
C3	C4	C5	C6	-1.81(19)
C4	C5	C6	C7	4.53(18)
C4	C5	C6	C8	-173.62(11)
C5	C6	C7	C2	-2.00(18)
C5	C6	C8	C9	135.09(14)
C5	C6	C8	C13	-42.38(18)
C6	C8	C9	C10	-177.23(13)
C6	C8	C13	C12	177.96(13)
C7	C2	C3	N1	-170.97(11)
C7	C2	C3	C4	5.95(17)
C7	C6	C8	C9	-43.00(18)
C7	C6	C8	C13	139.54(13)
C8	C6	C7	C2	176.12(11)
C8	C9	C10	C11	-0.8(2)
C9	C8	C13	C12	0.4(2)
C9	C10	C11	C12	0.6(3)
C10	C11	C12	C13	0.1(3)
C11	C12	C13	C8	-0.7(2)
C13	C8	C9	C10	0.3(2)
C14	N1	C3	C2	11.65(15)
C14	N1	C3	C4	-165.28(11)
C14	N1	C15	C16	-48.79(13)
C14	N2	C1	C2	-46.24(13)
C14	N2	C17	C16	12.05(16)
C14	N2	C17	C18	-166.08(11)
C15	N1	C3	C2	-109.23(13)
C15	N1	C3	C4	73.84(14)
C15	N1	C14	N2	71.90(13)
C15	C16	C17	N2	6.92(18)
C15	C16	C17	C18	-174.97(11)
C15	C16	C21	C20	176.08(11)
C16	C17	C18	C19	-1.63(18)
C17	N2	C1	C2	75.63(13)
C17	N2	C14	N1	-51.60(14)
C17	C16	C21	C20	-1.95(18)
C17	C18	C19	C20	-1.00(19)

Atom	Atom	Atom	Atom	Angle/˚
C18	C19	C20	C21	2.11(18)
C18	C19	C20	C22	-177.59(11)
C19	C20	C21	C16	-0.63(18)
C19	C20	C22	C23	99.51(14)
C19	C20	C22	C27	-76.07(15)
C20	C22	C23	C24	-177.13(12)
C20	C22	C23	C28	1.38(19)
C20	C22	C27	C26	176.63(12)
C20	C22	C27	C29	-4.07(18)
C21	C16	C17	N2	-175.04(11)
C21	C16	C17	C18	3.07(17)
C21	C20	C22	C23	-80.19(15)
C21	C20	C22	C27	104.23(14)
C22	C20	C21	C16	179.07(11)
C22	C23	C24	C25	0.5(2)
C22	C27	C29	O1	-36.70(16)
C22	C27	C29	O2	145.88(14)
C23	C22	C27	C26	1.04(18)
C23	C22	C27	C29	-179.66(11)
C23	C24	C25	C26	0.8(2)
C24	C25	C26	C27	-1.2(2)
C25	C26	C27	C22	0.3(2)
C25	C26	C27	C29	-179.09(12)
C26	C27	C29	O1	142.63(11)
C26	C27	C29	O2	-34.79(18)
C27	C22	C23	C24	-1.39(19)
C27	C22	C23	C28	177.12(13)
C28	C23	C24	C25	-178.05(14)
C29	O1	C30	C31	59.28(16)
C29	O1	C30	C35	-125.82(12)
C30	O1	C29	O2	2.80(19)
C30	O1	C29	C27	-174.66(10)
C30	C31	C32	C33	-0.8(2)
C31	C30	C35	C34	-1.6(2)
C31	C32	C33	F1	178.98(14)
C31	C32	C33	C34	-0.5(2)
C32	C33	C34	C35	0.7(2)
C33	C34	C35	C30	0.3(2)
C35	C30	C31	C32	1.9(2)

Table S30: Hydrogen Fractional Atomic Coordinates (×10[4]) and Equivalent Isotropic Displacement Parameters (Å[2]×10[3]) for **SC16016.** U_{eq} is defined as 1/3 of the trace of the orthogonalised U_{ij}.

Atom	x	y	z	U_{eq}
H1A	-1139(10)	-4190(30)	2095(7)	21(4)
H1B	-282(10)	-3020(30)	2259(7)	22(4)
H4	-2228(10)	3270(30)	2060(7)	21(4)
H5	-1912(10)	3340(30)	3032(7)	29(4)
H7	-749(10)	-2240(30)	3141(7)	27(4)
H9	-1502(11)	-2600(30)	3856(8)	37(5)
H10	-1365(13)	-2580(40)	4817(9)	54(6)
H11	-990(14)	390(40)	5357(10)	59(7)
H12	-752(13)	3610(40)	4910(10)	57(6)
H13	-898(11)	3630(30)	3941(8)	32(5)
H14A	-2196(10)	-2780(30)	1323(7)	21(4)
H14B	-1963(9)	-1700(20)	807(7)	16(4)
H15A	-1547(9)	3270(30)	1305(7)	19(4)
H15B	-1863(10)	2010(30)	737(7)	24(4)
H18	530(10)	-2810(30)	1599(7)	22(4)
H19	1456(10)	-410(30)	1415(7)	25(4)
H21	-325(9)	3980(30)	954(7)	19(4)
H24	1786(10)	5110(30)	-55(8)	30(4)
H25	2762(10)	6950(30)	671(7)	28(4)
H26	2771(10)	6500(30)	1604(7)	26(4)
H28A	733(11)	2840(30)	-364(8)	37(5)
H28B	732(12)	1030(30)	58(8)	42(5)
H28C	84(13)	2880(30)	-27(9)	46(6)
H31	1660(11)	7120(30)	2899(8)	33(5)
H32	1501(11)	7210(30)	3834(8)	35(5)
H34	278(11)	1710(30)	3503(8)	38(5)
H35	445(10)	1640(30)	2579(7)	25(4)

Citations

O.V. Dolomanov and L.J. Bourhis and R.J. Gildea and J.A.K. Howard and H. Puschmann, Olex2: A complete structure solution, refinement and analysis program, *J. Appl. Cryst.*, (2009), **42**, 339-341.

Sheldrick, G.M., A short history of ShelX, *Acta Cryst.*, (2008), **A64**, 339-341.

Sheldrick, G.M., Crystal structure refinement with ShelXL, *Acta Cryst.*, (2015), **C27**, 3-8.

```
#==============================================================================
# PLATON/CHECK-(150616) versus check.def version of 160610 for Entry: sc16016
# Data: SC16016.cif - Type: CIF                   Bond Precision   C-C = 0.0020 A
# Refl: SC16016.fcf - Type: LIST4                                  Temp = 120 K
# X-ray MoKa              R(int) = 0.037,  wR2/R(int) =  3.5,  Nref/Npar =  17.2
# Cell  17.4782(5) 6.38739(19)  24.9672(9)          90   107.173(4)          90
# Wavelength 0.71073    Volume Reported   2663.07(16)   Calculated   2663.07(16)
# SpaceGroup from Symmetry P 21/n     Hall: -P 2yn                monoclinic
#                 Reported P 1 21/n 1       -P 2yn                monoclinic
# MoietyFormula C35 H27 F N2 O2
#      Reported C35 H27 F N2 O2
#    SumFormula C35 H27 F N2 O2
#      Reported C35 H27 F N2 O2
# Mr        =    526.59[Calc],    526.58[Rep]
# Dx,gcm-3  =     1.313[Calc],     1.313[Rep]
# Z         =         4[Calc],         4[Rep]
# Mu (mm-1) =     0.086[Calc],     0.086[Rep]
# F000      =    1104.0[Calc],    1104.0[Rep]  or F000' =   1104.50[Calc]
# Reported   T Limits: Tmin=0.503              Tmax=1.000  AbsCorr = GAUSSIAN
# Calculated T Limits: Tmin=0.965 Tmin'=0.952  Tmax=0.986
# Reported   Hmax= 25, Kmax=  8, Lmax= 36, Nref=   8067        , Th(max)= 31.200
# Obs in FCF Hmax= 25, Kmax=  8, Lmax= 36, Nref=   8067[  8067], Th(max)= 31.200
# Calculated Hmax= 25, Kmax=  9, Lmax= 36, Nref=   8633        , Ratio  =  0.934
# Reported   Rho(min) = -0.31, Rho(max) =  0.34 e/Ang**3 (From CIF)
# Calculated Rho(min) = -0.32, Rho(max) =  0.38 e/Ang**3 (From CIF+FCF data)
# w=1/[sigma**2(Fo**2)+(0.0478P)**2+  1.4149P], P=(Fo**2+2*Fc**2)/3
# R= 0.0533(  7018), wR2= 0.1294(  8067), S = 1.109      (From CIF+FCF data)
# R= 0.0533(  7018), wR2= 0.1294(  8067), S = 1.109      (From FCF data only)
# R= 0.0533(  7020), wR2= 0.1294(  8067), S = 1.109, Npar=  469
#==============================================================================
For Documentation: http://http://www.platonsoft.nl/CIF-VALIDATION.pdf
#==============================================================================

#==============================================================================
>>> The Following Improvement and Query ALERTS were generated - (Acta-Mode) <<<
#==============================================================================
Format: alert-number_ALERT_alert-type_alert-level text

906_ALERT_3_C Large K value in the Analysis of Variance ......      6.743 Check
910_ALERT_3_C Missing # of FCF Reflection(s) Below Theta(Min)           6 Note
911_ALERT_3_C Missing # FCF Refl Between THmin & STh/L=  0.600          2 Report
#==============================================================================
912_ALERT_4_G Missing # of FCF Reflections Above STh/L=  0.600        466 Note
913_ALERT_3_G Missing # of Very Strong Reflections in FCF ....          1 Note
978_ALERT_2_G Number C-C Bonds with Positive Residual Density          24 Note
#==============================================================================

ALERT_Level and ALERT_Type Summary
==================================
3 ALERT_Level_C = Check. Ensure it is Not caused by an Omission or Oversight
3 ALERT_Level_G = General Info/Check that it is not Something Unexpected

1 ALERT_Type_2 Indicator that the Structure Model may be Wrong or Deficient.
4 ALERT_Type_3 Indicator that the Structure Quality may be Low.
1 ALERT_Type_4 Improvement, Methodology, Query or Suggestion.
#==============================================================================

0 Missing  Experimental  Info Issue(s) (Out of  62 Tests) -  100 % Satisfied
0 Experimental  Data  Related Issue(s) (Out of  28 Tests) -  100 % Satisfied
2 Structural  Model   Related Issue(s) (Out of 126 Tests) -   98 % Satisfied
4 Unresolved or to be Checked Issue(s) (Out of 247 Tests) -   98 % Satisfied

#==============================================================================
```

Compound (±)-**1Cl** CCDC Deposition Number 2244213

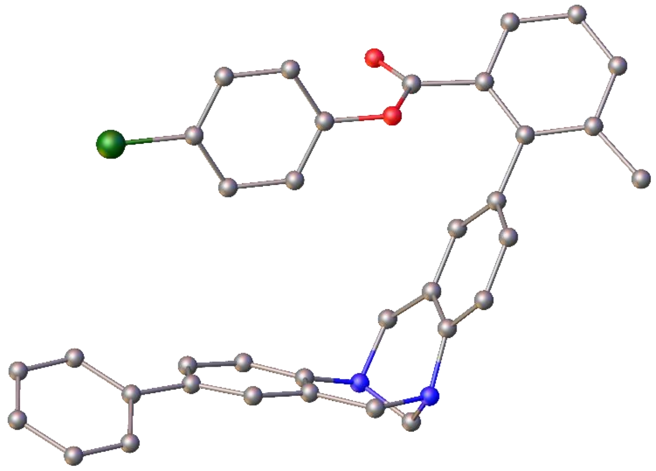

**Compound**	**SC17001**
Formula	$C_{35}H_{27}ClN_2O_2$
$D_{calc.}$/ g cm^{-3}	1.349
μ/mm^{-1}	0.180
Formula Weight	543.03
Colour	colourless
Shape	block
Size/mm^3	0.45×0.38×0.19
T/K	120.0
Crystal System	monoclinic
Space Group	$P2_1/n$
a/Å	9.85577(17)
b/Å	21.1751(3)
c/Å	12.8496(2)
α/°	90
β/°	94.5118(15)
γ/°	90
V/Å^3	2673.36(8)
Z	4
Z'	1
Wavelength/Å	0.71073
Radiation type	MoK_α
Θ_{min}/°	2.710
Θ_{max}/°	32.992
Measured Refl.	102973
Independent Refl.	9722
Reflections Used	8876
R_{int}	0.0374
Parameters	362
Restraints	0
Largest Peak	0.472
Deepest Hole	-0.490
GooF	1.122
wR_2 (all data)	0.1360
wR_2	0.1322
R_1 (all data)	0.0635
R_1	0.0564

Experimental. Single colourless block-shaped crystals of (**SC17001**) were recrystallised from a mixture of diethyl ether and hexane by slow evaporation. A suitable crystal (0.45×0.38×0.19) mm^3 was selected and mounted on a MITIGEN holder in Paratone oil on a Rigaku Oxford Diffraction SuperNova diffractometer. The crystal was kept at T = 120.0 K during data collection. Using **Olex2** (Dolomanov et al., 2009), the structure was solved with the **ShelXS** (Sheldrick, 2008) structure solution program, using the Direct Methods solution method. The model was refined with version 2016/6 of **ShelXL** (Sheldrick, 2015) using Least Squares minimisation.

Crystal Data. $C_{35}H_{27}ClN_2O_2$, M_r = 543.03, monoclinic, $P2_1/n$ (No. 14), a = 9.85577(17) Å, b = 21.1751(3) Å, c = 12.8496(2) Å, β = 94.5118(15)°, α = γ = 90°, V = 2673.36(8) Å^3, T = 120.0 K, Z = 4, Z' = 1, $\mu(MoK_\alpha)$ = 0.180, 102973 reflections measured, 9722 unique (R_{int} = 0.0374) which were used in all calculations. The final wR_2 was 0.1360 (all data) and R_1 was 0.0564 (I > 2(I)).

Structure Quality Indicators

**Reflections:**	d min (Mo) 0.65	I/σ 29.9	Rint 3.74%	complete at 2θ=61° 97%
**Refinement:**	Shift 0.001	Max Peak 0.5	Min Peak -0.5	GooF 1.122

A colourless block-shaped crystal with dimensions 0.45×0.38×0.19 mm^3 was mounted on a MITIGEN holder in Paratone oil. X-ray diffraction data were collected using a Rigaku Oxford Diffraction SuperNova diffractometer equipped with a Oxford Cryosystems Cryostream 700+ low-temperature device, operating at T = 120.0 K.

Data were measured using ω scans scans of 0.5 ° per frame for 10.0 s using MoK_α radiation (micro-focus sealed X-ray tube, 50 kV, 0.8 mA). The total number of runs and images was based on the strategy calculation from the program **CrysAlisPro** (Rigaku, V1.171.38.43h, 2015).The maximum resolution achieved was Θ = 32.992°.

Cell parameters were retrieved using the **CrysAlisPro** (Rigaku, V1.171.38.43h, 2015) software and refined using **CrysAlisPro** (Rigaku, V1.171.38.43h, 2015) on 34155 reflections, 33 % of the observed reflections. Data reduction was performed using the **CrysAlisPro** (Rigaku, V1.171.38.43h, 2015) software which corrects for Lorentz polarisation. The final completeness is 99.80 out to 32.992 in Θ. The absorption coefficient μ of this material is 0.180 at this wavelength (λ = 0.71073) and the minimum and maximum transmissions are 0.520 and 1.000.

The structure was solved in the space group $P2_1/n$ (# 14) by Direct Methods using the **ShelXS** (Sheldrick, 2008) structure solution program and refined by Least Squares using version 2016/6 of **ShelXL** (Sheldrick, 2015). All non-hydrogen atoms were refined anisotropically. Hydrogen atom positions were calculated geometrically and refined using the riding model.

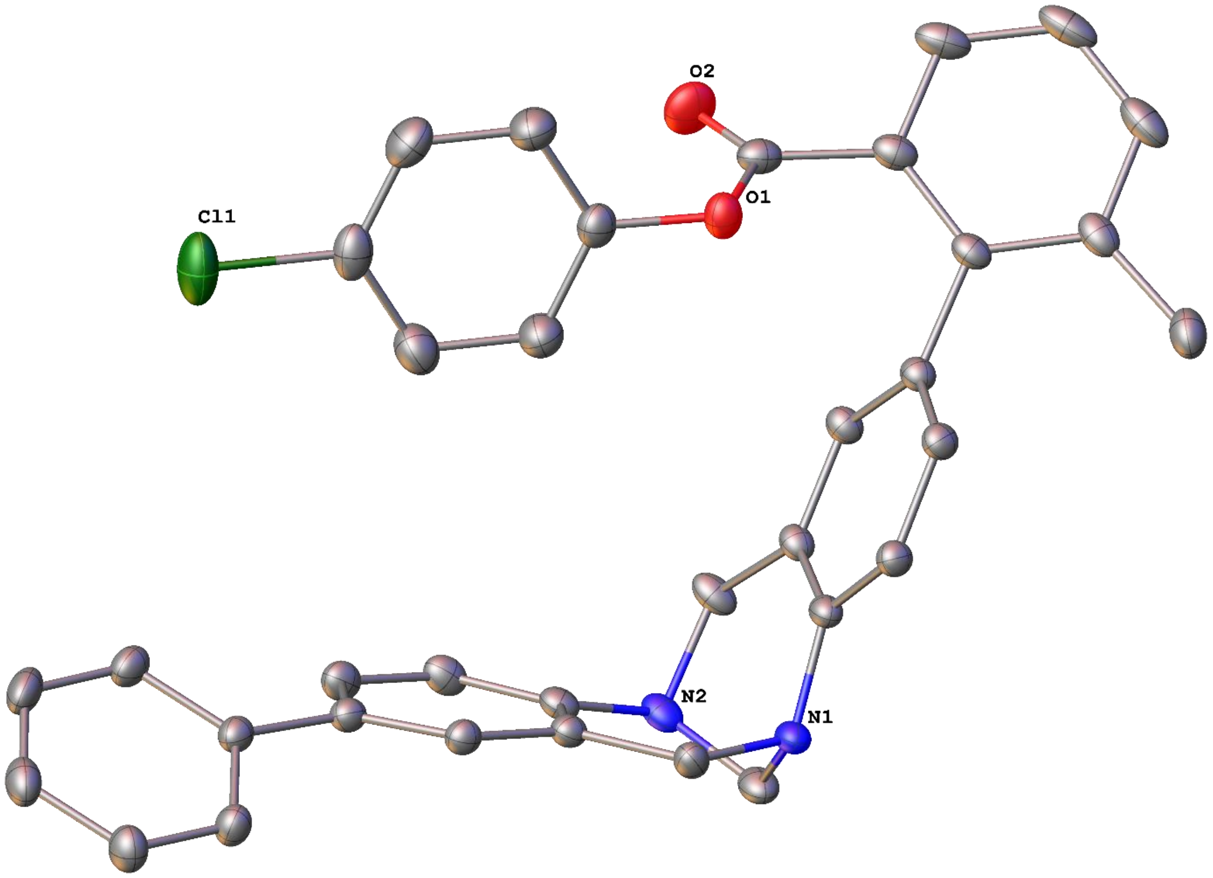

Figure S6: The asymmetric unit of SC17001. Displacement ellipsoids are at the 50% probability level and H atoms are not shown.

Data Plots: Diffraction Data

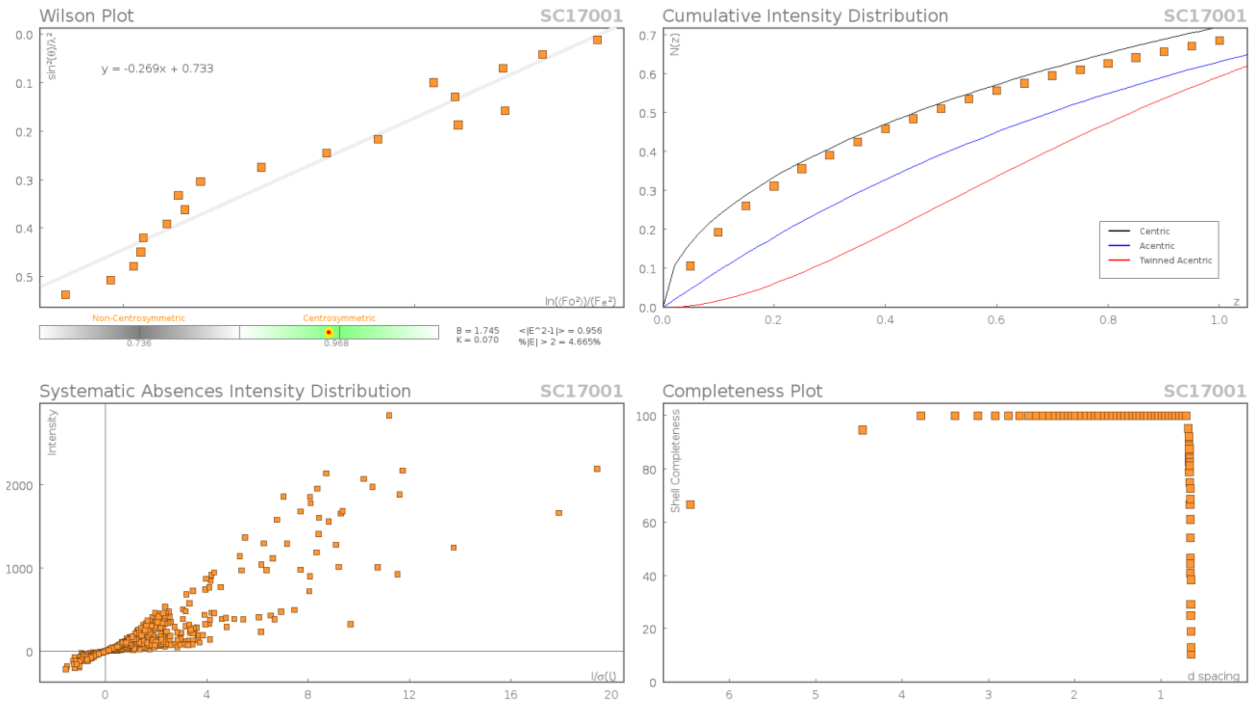

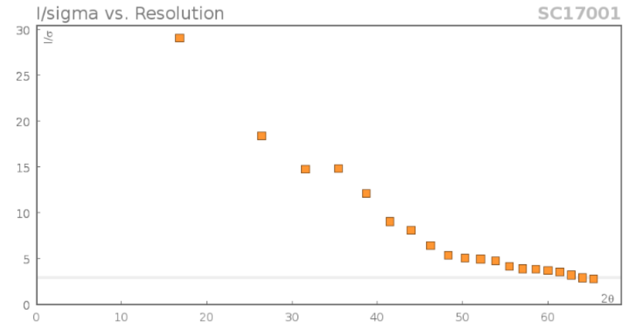

Data Plots: Refinement and Data

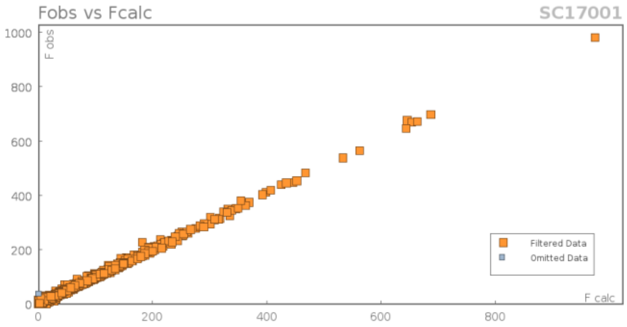

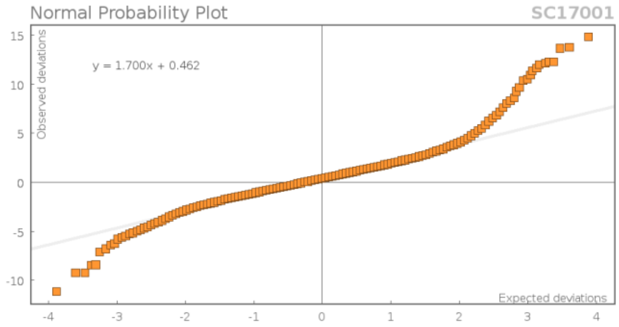

Reflection Statistics

Total reflections (after filtering)	104461	Unique reflections	9722
Completeness	0.967	Mean I/σ	29.88
hkl_{max} collected	(15, 32, 19)	hkl_{min} collected	(-14, -32, -18)
hkl_{max} used	(14, 32, 19)	hkl_{min} used	(-15, 0, 0)
Lim d_{max} collected	100.0	Lim d_{min} collected	0.36
d_{max} used	7.52	d_{min} used	0.65
Friedel pairs	17445	Friedel pairs merged	1
Inconsistent equivalents	1	R_{int}	0.0374
R_{sigma}	0.0206	Intensity transformed	0
Omitted reflections	0	Omitted by user (OMIT hkl)	13
Multiplicity	(8144, 9841, 7215, 5427, 3345, 1532, 576, 152, 58, 67, 46, 24, 9, 1)	Maximum multiplicity	35
Removed systematic absences	1475	Filtered off (Shel/OMIT)	0

Table S31: Fractional Atomic Coordinates (×10^4) and Equivalent Isotropic Displacement Parameters (Å^2×10^3) for **SC17001.** U_{eq} is defined as 1/3 of the trace of the orthogonalised U_{ij}.

Atom	x	y	z	U_{eq}
Cl1	1536.7(5)	5742.0(2)	8472.9(4)	42.87(12)
O1	3478.3(10)	3732.6(4)	11379.5(8)	23.79(19)
O2	5017.4(12)	4378.3(5)	12222.3(9)	34.5(2)
N1	5128.0(11)	2152.2(5)	8027.4(8)	18.86(19)
N2	6711.5(11)	3014.7(5)	7840.3(8)	20.8(2)
C1	4065.1(13)	2419.9(6)	7288.1(9)	19.2(2)
C2	4364.4(12)	3099.5(5)	7010.7(9)	17.0(2)
C3	3410.9(12)	3446.4(5)	6390.0(9)	17.5(2)
C4	3661.1(12)	4065.6(6)	6086.2(9)	17.9(2)
C5	4895.3(13)	4346.1(6)	6461.3(10)	21.0(2)
C6	5857.3(13)	4005.8(6)	7071.8(10)	21.4(2)
C7	5620.5(12)	3375.4(6)	7328.2(9)	18.0(2)
C8	2693.8(12)	4403.1(5)	5339.1(9)	18.6(2)

Atom	x	y	z	U_{eq}
C9	1934.8(13)	4065.7(6)	4559.8(10)	21.9(2)
C10	1057.9(14)	4369.8(7)	3823.0(11)	25.8(3)
C11	933.7(15)	5021.7(7)	3848.2(11)	27.5(3)
C12	1673.6(16)	5363.5(6)	4618.5(12)	28.6(3)
C13	2541.1(14)	5059.6(6)	5362.1(11)	23.9(2)
C14	6454.7(13)	2339.1(6)	7689.2(10)	23.0(2)
C15	6921.3(12)	3136.5(7)	8970.5(9)	21.8(2)
C16	5907.7(11)	2778.7(6)	9581.9(9)	16.8(2)
C17	5011.2(12)	2341.4(5)	9088.5(9)	16.6(2)
C18	4022.2(12)	2054.3(5)	9652.8(9)	18.0(2)
C19	3973.6(12)	2169.5(6)	10710.4(9)	18.1(2)
C20	4921.9(11)	2576.3(5)	11229.8(9)	16.6(2)
C21	5860.5(12)	2884.4(6)	10650.7(9)	17.7(2)
C22	4987.2(12)	2685.2(6)	12379.0(9)	17.8(2)
C23	5270.6(12)	2184.4(7)	13086.5(10)	21.9(2)
C24	5535.0(14)	2325.7(8)	14146.6(10)	28.2(3)
C25	5498.3(14)	2938.9(8)	14518.8(10)	30.2(3)
C26	5173.6(13)	3430.0(7)	13834.5(10)	26.0(3)
C27	4910.1(12)	3303.0(6)	12770.1(9)	20.1(2)
C28	5289.4(15)	1503.4(7)	12747.4(11)	28.0(3)
C29	4523.6(13)	3862.8(6)	12105.9(10)	21.6(2)
C30	3052.6(14)	4229.1(6)	10705.4(11)	23.2(2)
C31	3476.3(16)	4227.7(7)	9704.7(12)	29.4(3)
C32	3013.9(17)	4700.0(8)	9008.6(12)	32.4(3)
C33	2140.9(15)	5154.5(7)	9340.5(12)	29.4(3)
C34	1715.6(16)	5153.3(7)	10342.3(13)	32.6(3)
C35	2179.7(16)	4683.8(7)	11035.0(12)	30.2(3)

Table S32: Anisotropic Displacement Parameters (×10[4]) **SC17001.** The anisotropic displacement factor exponent takes the form: $-2\pi^2[h^2a^{*2} \times U_{11} + ... + 2hka^* \times b^* \times U_{12}]$

Atom	U_{11}	U_{22}	U_{33}	U_{23}	U_{13}	U_{12}
Cl1	40.9(2)	32.44(19)	52.4(3)	16.35(17)	-14.55(18)	-3.41(15)
O1	26.4(4)	19.5(4)	24.8(4)	2.4(3)	-2.4(3)	-3.0(3)
O2	38.9(6)	23.9(5)	39.9(6)	-5.6(4)	-2.4(5)	-8.7(4)
N1	22.7(5)	19.6(4)	14.8(4)	0.7(3)	4.9(3)	3.7(4)
N2	17.2(4)	29.5(5)	16.3(4)	3.2(4)	5.0(3)	1.0(4)
C1	24.5(5)	16.4(5)	16.5(5)	0.0(4)	0.8(4)	-1.2(4)
C2	19.3(5)	17.2(5)	15.0(5)	-0.5(4)	3.9(4)	-1.1(4)
C3	19.0(5)	15.9(5)	18.0(5)	-0.8(4)	2.8(4)	-1.7(4)
C4	20.5(5)	16.4(5)	17.2(5)	-0.9(4)	3.5(4)	-0.8(4)
C5	24.5(6)	17.7(5)	21.0(5)	-0.3(4)	3.1(4)	-5.3(4)
C6	20.9(5)	24.5(6)	18.9(5)	-0.1(4)	2.1(4)	-7.1(4)
C7	18.1(5)	22.7(5)	13.7(5)	0.1(4)	4.7(4)	-1.1(4)
C8	20.9(5)	15.5(5)	19.9(5)	0.7(4)	5.4(4)	-0.1(4)
C9	24.8(6)	17.1(5)	23.7(6)	0.9(4)	0.5(4)	1.2(4)
C10	27.1(6)	24.2(6)	25.7(6)	3.8(5)	-1.2(5)	1.6(5)
C11	28.7(6)	25.5(6)	28.8(6)	9.5(5)	6.2(5)	7.0(5)
C12	36.2(7)	16.2(5)	34.6(7)	5.1(5)	11.0(6)	5.4(5)
C13	29.4(6)	15.6(5)	27.6(6)	-0.2(4)	7.6(5)	-0.7(4)
C14	23.8(6)	27.0(6)	19.3(5)	2.3(4)	8.7(4)	6.6(5)
C15	16.1(5)	33.5(6)	16.1(5)	3.9(4)	2.9(4)	-3.8(4)
C16	14.7(4)	20.3(5)	15.8(5)	3.0(4)	3.8(4)	0.8(4)
C17	18.2(5)	17.5(5)	14.5(5)	1.3(4)	3.9(4)	3.0(4)
C18	19.6(5)	16.6(5)	18.2(5)	0.3(4)	3.5(4)	-1.3(4)
C19	18.7(5)	17.8(5)	18.2(5)	1.2(4)	4.5(4)	-1.6(4)
C20	16.1(5)	18.6(5)	15.4(5)	1.1(4)	3.3(4)	0.9(4)
C21	15.6(5)	21.1(5)	16.3(5)	1.5(4)	1.6(4)	-0.9(4)
C22	15.1(5)	24.0(5)	14.7(5)	1.1(4)	3.4(4)	-1.6(4)
C23	16.0(5)	30.9(6)	19.1(5)	6.3(4)	3.8(4)	-0.4(4)
C24	21.2(6)	45.9(8)	17.6(5)	9.4(5)	2.7(4)	-2.3(5)

Atom	U_{11}	U_{22}	U_{33}	U_{23}	U_{13}	U_{12}
C25	22.4(6)	54.3(9)	14.1(5)	-0.6(5)	3.1(4)	-5.8(6)
C26	21.8(6)	38.6(7)	18.0(5)	-7.1(5)	4.4(4)	-7.0(5)
C27	17.2(5)	26.7(6)	16.7(5)	-2.2(4)	3.3(4)	-3.8(4)
C28	28.9(6)	27.0(6)	28.6(6)	10.2(5)	6.0(5)	4.8(5)
C29	22.3(5)	23.0(6)	20.0(5)	-4.4(4)	5.4(4)	-2.9(4)
C30	25.1(6)	18.3(5)	26.0(6)	1.2(4)	1.5(5)	-2.0(4)
C31	32.8(7)	27.7(6)	28.5(7)	1.3(5)	6.6(5)	4.4(5)
C32	35.4(7)	34.0(7)	28.3(7)	6.7(6)	4.8(6)	-0.3(6)
C33	28.3(6)	22.5(6)	36.2(7)	6.1(5)	-5.3(5)	-4.2(5)
C34	31.9(7)	25.5(6)	40.4(8)	-1.2(6)	2.2(6)	6.5(5)
C35	33.5(7)	28.4(7)	29.4(7)	-1.2(5)	7.5(5)	4.3(5)

Table S33: Bond Lengths in Å for **SC17001**.

Atom	Atom	Length/Å
Cl1	C33	1.7437(15)
O1	C29	1.3628(16)
O1	C30	1.4051(16)
O2	C29	1.1999(16)
N1	C1	1.4714(16)
N1	C14	1.4645(17)
N1	C17	1.4345(15)
N2	C7	1.4357(16)
N2	C14	1.4630(18)
N2	C15	1.4735(16)
C1	C2	1.5169(16)
C2	C3	1.3932(16)
C2	C7	1.4003(16)
C3	C4	1.3955(16)
C4	C5	1.4045(17)
C4	C8	1.4823(17)
C5	C6	1.3845(18)
C6	C7	1.3990(17)
C8	C9	1.3981(17)
C8	C13	1.3988(17)
C9	C10	1.3889(18)
C10	C11	1.3865(19)
C11	C12	1.386(2)
C12	C13	1.389(2)
C15	C16	1.5208(16)
C16	C17	1.3970(17)
C16	C21	1.3960(16)
C17	C18	1.3988(16)
C18	C19	1.3853(16)
C19	C20	1.4010(16)
C20	C21	1.3941(16)
C20	C22	1.4909(16)
C22	C23	1.4103(17)
C22	C27	1.4057(18)
C23	C24	1.3989(19)
C23	C28	1.507(2)
C24	C25	1.385(2)
C25	C26	1.383(2)
C26	C27	1.3980(17)
C27	C29	1.4925(19)
C30	C31	1.383(2)
C30	C35	1.3798(19)
C31	C32	1.394(2)
C32	C33	1.381(2)
C33	C34	1.385(2)
C34	C35	1.388(2)

Table S34: Bond Angles in ° for **SC17001**.

Atom	Atom	Atom	Angle/°
C29	O1	C30	116.35(10)
C14	N1	C1	108.13(9)
C17	N1	C1	113.56(9)
C17	N1	C14	110.29(10)
C7	N2	C14	110.11(10)
C7	N2	C15	113.61(10)
C14	N2	C15	108.14(10)
N1	C1	C2	112.06(10)
C3	C2	C1	119.99(10)
C3	C2	C7	119.19(11)
C7	C2	C1	120.74(11)
C2	C3	C4	121.97(11)
C3	C4	C5	117.95(11)
C3	C4	C8	121.02(11)
C5	C4	C8	120.93(11)
C6	C5	C4	120.69(11)
C5	C6	C7	120.70(11)
C2	C7	N2	121.64(11)
C6	C7	N2	118.86(11)
C6	C7	C2	119.31(11)
C9	C8	C4	119.88(11)
C9	C8	C13	118.07(12)
C13	C8	C4	122.01(11)
C10	C9	C8	121.36(12)
C11	C10	C9	119.80(13)
C12	C11	C10	119.60(13)
C11	C12	C13	120.66(12)
C12	C13	C8	120.48(13)
N2	C14	N1	111.97(10)
N2	C15	C16	112.02(10)
C17	C16	C15	120.90(10)
C21	C16	C15	120.16(11)

Atom	Atom	Atom	Angle/˚
C21	C16	C17	118.95(10)
C16	C17	N1	121.42(10)
C16	C17	C18	119.63(10)
C18	C17	N1	118.85(11)
C19	C18	C17	120.75(11)
C18	C19	C20	120.15(10)
C19	C20	C22	122.83(10)
C21	C20	C19	118.64(10)
C21	C20	C22	118.52(10)
C20	C21	C16	121.66(11)
C23	C22	C20	120.87(11)
C27	C22	C20	119.92(11)
C27	C22	C23	118.86(11)
C22	C23	C28	122.64(12)
C24	C23	C22	118.64(13)
C24	C23	C28	118.73(12)
C25	C24	C23	121.92(13)
C26	C25	C24	119.76(12)
C25	C26	C27	119.55(13)
C22	C27	C29	123.59(11)
C26	C27	C22	121.19(12)
C26	C27	C29	115.21(12)
O1	C29	C27	112.10(10)
O2	C29	O1	123.14(13)
O2	C29	C27	124.65(13)
C31	C30	O1	118.31(12)
C35	C30	O1	119.74(12)
C35	C30	C31	121.87(13)
C30	C31	C32	119.19(14)
C33	C32	C31	118.82(14)
C32	C33	Cl1	119.31(12)
C32	C33	C34	121.83(13)
C34	C33	Cl1	118.85(12)
C33	C34	C35	119.27(14)
C30	C35	C34	119.02(14)

Table S35: Torsion Angles in ˚ for **SC17001**.

Atom	Atom	Atom	Atom	Angle/˚
Cl1	C33	C34	C35	179.33(12)
O1	C30	C31	C32	177.05(13)
O1	C30	C35	C34	-176.83(13)
N1	C1	C2	C3	-174.52(10)
N1	C1	C2	C7	8.68(15)
N1	C17	C18	C19	172.65(11)
N2	C15	C16	C17	6.40(16)
N2	C15	C16	C21	-173.53(11)
C1	N1	C14	N2	70.06(12)
C1	N1	C17	C16	-106.48(13)
C1	N1	C17	C18	77.00(13)
C1	C2	C3	C4	-177.81(11)
C1	C2	C7	N2	5.78(17)
C1	C2	C7	C6	-179.13(11)
C2	C3	C4	C5	-2.58(17)
C2	C3	C4	C8	173.70(11)
C3	C2	C7	N2	-171.04(10)
C3	C2	C7	C6	4.05(17)
C3	C4	C5	C6	3.07(18)
C3	C4	C8	C9	-31.62(17)
C3	C4	C8	C13	150.58(12)
C4	C5	C6	C7	-0.02(19)
C4	C8	C9	C10	-177.53(12)
C4	C8	C13	C12	176.86(12)
C5	C4	C8	C9	144.56(12)
C5	C4	C8	C13	-33.25(18)
C5	C6	C7	N2	171.63(11)
C5	C6	C7	C2	-3.59(18)
C7	N2	C14	N1	-54.73(13)
C7	N2	C15	C16	80.02(13)

Atom	Atom	Atom	Atom	Angle/°
C7	C2	C3	C4	-0.96(17)
C8	C4	C5	C6	-173.22(11)
C8	C9	C10	C11	0.6(2)
C9	C8	C13	C12	-0.98(19)
C9	C10	C11	C12	-0.9(2)
C10	C11	C12	C13	0.2(2)
C11	C12	C13	C8	0.7(2)
C13	C8	C9	C10	0.36(19)
C14	N1	C1	C2	-43.61(13)
C14	N1	C17	C16	15.06(15)
C14	N1	C17	C18	-161.45(11)
C14	N2	C7	C2	16.43(15)
C14	N2	C7	C6	-158.68(11)
C14	N2	C15	C16	-42.53(13)
C15	N2	C7	C2	-105.03(13)
C15	N2	C7	C6	79.86(13)
C15	N2	C14	N1	69.94(13)
C15	C16	C17	N1	8.29(17)
C15	C16	C17	C18	-175.22(11)
C15	C16	C21	C20	178.39(11)
C16	C17	C18	C19	-3.93(17)
C17	N1	C1	C2	79.13(12)
C17	N1	C14	N2	-54.66(13)
C17	C16	C21	C20	-1.54(17)
C17	C18	C19	C20	-0.12(18)
C18	C19	C20	C21	3.28(17)
C18	C19	C20	C22	-175.59(11)
C19	C20	C21	C16	-2.45(17)
C19	C20	C22	C23	61.65(16)
C19	C20	C22	C27	-125.16(13)
C20	C22	C23	C24	169.95(11)
C20	C22	C23	C28	-10.52(18)
C20	C22	C27	C26	-170.19(11)
C20	C22	C27	C29	11.04(17)
C21	C16	C17	N1	-171.78(10)
C21	C16	C17	C18	4.71(17)
C21	C20	C22	C23	-117.22(13)
C21	C20	C22	C27	55.97(15)
C22	C20	C21	C16	176.47(11)
C22	C23	C24	C25	1.34(19)
C22	C27	C29	O1	41.04(16)
C22	C27	C29	O2	-142.55(14)
C23	C22	C27	C26	3.14(18)
C23	C22	C27	C29	-175.63(11)
C23	C24	C25	C26	0.9(2)
C24	C25	C26	C27	-1.2(2)
C25	C26	C27	C22	-0.88(19)
C25	C26	C27	C29	177.99(12)
C26	C27	C29	O1	-137.79(11)

Atom	Atom	Atom	Atom	Angle/°
C26	C27	C29	O2	38.61(18)
C27	C22	C23	C24	-3.31(17)
C27	C22	C23	C28	176.22(12)
C28	C23	C24	C25	-178.20(12)
C29	O1	C30	C31	101.67(15)
C29	O1	C30	C35	-81.46(16)
C30	O1	C29	O2	4.38(19)
C30	O1	C29	C27	-179.15(10)
C30	C31	C32	C33	-0.2(2)
C31	C30	C35	C34	-0.1(2)
C31	C32	C33	Cl1	-179.16(12)
C31	C32	C33	C34	0.0(2)
C32	C33	C34	C35	0.1(2)
C33	C34	C35	C30	-0.1(2)
C35	C30	C31	C32	0.2(2)

Table S36: Hydrogen Fractional Atomic Coordinates (×10^4) and Equivalent Isotropic Displacement Parameters (Å^2×10^3) for **SC17001.** U_{eq} is defined as 1/3 of the trace of the orthogonalised U_{ij}.

Atom	x	y	z	U_{eq}
H1A	3178.75	2400.34	7599.66	23
H1B	3992.45	2162.22	6643.29	23
H3	2565.11	3255.74	6166.83	21
H5	5071.83	4773.86	6294.34	25
H6	6687.21	4202.44	7319.16	26
H9	2020.65	3619.23	4533.69	26
H10	544.79	4131.76	3303.38	31
H11	345.16	5232.91	3340.5	33
H12	1586.4	5810.05	4638.02	34
H13	3034.24	5299.33	5890.16	29
H14A	7178.5	2096.18	8089.15	28
H14B	6491.4	2234.2	6941.12	28
H15A	6828.67	3594.83	9099.47	26
H15B	7856.92	3009.53	9221.88	26
H18	3376.49	1777.25	9306.73	22
H19	3295.61	1972.19	11084.39	22
H21	6483.69	3173.7	10992.12	21
H24	5745.91	1991.34	14625.17	34
H25	5695.49	3021.86	15241.46	36
H26	5129.86	3850.84	14085.96	31
H28A	4357.34	1338.84	12679.24	42
H28B	5837.84	1255.53	13269.31	42
H28C	5685.4	1473.04	12073.08	42
H31	4075.15	3908.89	9494.15	35
H32	3294.47	4708.92	8318.17	39
H34	1112.73	5470.44	10552.99	39
H35	1900.59	4675.6	11725.87	36

```
#==============================================================================
# PLATON/CHECK-(160117) versus check.def version of 170116 for Entry: sc17001
# Data: SC17001.cif - Type: CIF                  Bond Precision   C-C = 0.0018 A
# Refl: SC17001.fcf - Type: LIST4                                  Temp = 120 K
# Audit:OLEX2 1.2-BETA (COMPILED 2017.01.04 SVN.R3372 FOR OLEXSYS, GUI SVN.R5292
# Refin:SHELXL (SHELDRICK, 2015)
# X-ray MoKa              R(int) = 0.037,  wR2/R(int) =  3.6,  Nref/Npar =  26.9
# Cell 9.85577(17)  21.1751(3)  12.8496(2)           90  94.5118(15)          90
# Wavelength 0.71073    Volume Reported    2673.36(8)   Calculated    2673.36(7)
# SpaceGroup from Symmetry P 21/n     Hall: -P 2yn                 monoclinic
#                 Reported P 1 21/n 1       -P 2yn                 monoclinic
# MoietyFormula C35 H27 Cl N2 O2
#      Reported C35 H27 Cl N2 O2
#    SumFormula C35 H27 Cl N2 O2
#      Reported C35 H27 Cl N2 O2
# Mr        =    543.04[Calc],    543.03[Rep]
# Dx,gcm-3  =     1.349[Calc],     1.349[Rep]
# Z         =         4[Calc],         4[Rep]
# Mu (mm-1) =     0.180[Calc],     0.180[Rep]
# F000      =    1136.0[Calc],    1136.0[Rep]  or F000' =   1137.03[Calc]
# Reported   T Limits: Tmin=0.520              Tmax=1.000  AbsCorr = GAUSSIAN
# Calculated T Limits: Tmin=0.922 Tmin'=0.922  Tmax=0.967
# Reported   Hmax= 15, Kmax= 32, Lmax= 19, Nref=   9722        , Th(max)= 32.992
# Obs in FCF Hmax= 15, Kmax= 32, Lmax= 19, Nref=   9722[  9722], Th(max)= 32.992
# Calculated Hmax= 15, Kmax= 32, Lmax= 19, Nref=  10064        , Ratio  =  0.966
# Reported   Rho(min) = -0.49, Rho(max) =  0.47 e/Ang**3 (From CIF)
# Calculated Rho(min) = -0.46, Rho(max) =  0.47 e/Ang**3 (From CIF+FCF data)
# w=1/[sigma**2(Fo**2)+(0.0505P)**2+  1.5788P], P=(Fo**2+2*Fc**2)/3
# R= 0.0564(  8876), wR2= 0.1360(  9722), S = 1.122      (From CIF+FCF data)
# R= 0.0564(  8876), wR2= 0.1360(  9722), S = 1.122      (From FCF data only)
# R= 0.0564(  8876), wR2= 0.1360(  9722), S = 1.122, Npar=  362
#==============================================================================
For Documentation: http://http://www.platonsoft.nl/CIF-VALIDATION.pdf
#==============================================================================

#==============================================================================
>>> The Following Improvement and Query ALERTS were generated - (Acta-Mode) <<<
#==============================================================================
Format: alert-number_ALERT_alert-type_alert-level text

906_ALERT_3_C Large K value in the Analysis of Variance ......      7.197 Check
910_ALERT_3_C Missing # of FCF Reflection(s) Below Theta(Min)           6 Note
911_ALERT_3_C Missing # FCF Refl Between THmin &amp; STh/L=  0.600          3 Report
#==============================================================================
912_ALERT_4_G Missing # of FCF Reflections Above STh/L=  0.600        333 Note
913_ALERT_3_G Missing # of Very Strong Reflections in FCF ....          1 Note
933_ALERT_2_G Number of OMIT Records in Embedded .res File ...          1 Note
978_ALERT_2_G Number C-C Bonds with Positive Residual Density.         24 Note
#==============================================================================

ALERT_Level and ALERT_Type Summary
==================================
3 ALERT_Level_C = Check. Ensure it is Not caused by an Omission or Oversight
4 ALERT_Level_G = General Info/Check that it is not Something Unexpected

2 ALERT_Type_2 Indicator that the Structure Model may be Wrong or Deficient.
4 ALERT_Type_3 Indicator that the Structure Quality may be Low.
1 ALERT_Type_4 Improvement, Methodology, Query or Suggestion.
#==============================================================================

0 Missing  Experimental  Info Issue(s) (Out of  59 Tests) -  100 % Satisfied
0 Experimental  Data  Related Issue(s) (Out of  28 Tests) -  100 % Satisfied
3 Structural  Model   Related Issue(s) (Out of 127 Tests) -   98 % Satisfied
4 Unresolved or to be Checked Issue(s) (Out of 254 Tests) -   98 % Satisfied

#==============================================================================
```

Compound (±)-**1Br** CCDC Deposition Number 2244211

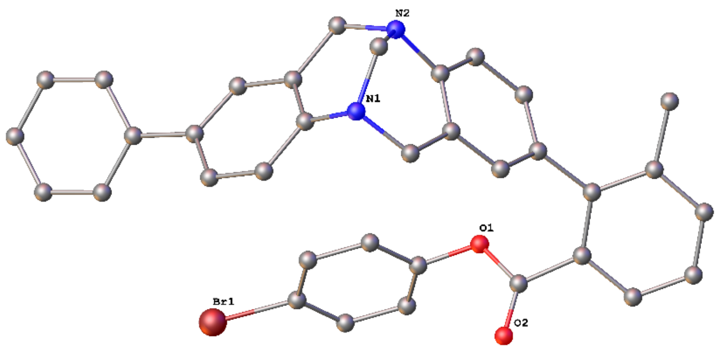

Experimental. Single clear colourless prism-shaped crystals of (**SC16010**) were recrystallised from a mixture of hexane and diethyl ether by slow evaporation. A suitable crystal (0.57×0.30×0.21) mm^3 was selected and mounted on a MITIGEN holder in Paratone oil on a Rigaku Oxford Diffraction SuperNova diffractometer. The crystal was kept at T = 120.0 K during data collection. Using **Olex2** (Dolomanov et al., 2009), the structure was solved with the **ShelXS** (Sheldrick, 2008) structure solution program, using the Direct Methods solution method. The model was refined with version 2014/7 of **ShelXL** (Sheldrick, 2015) using Least Squares minimisation.

Crystal Data. $C_{35}H_{27}BrN_2O_2$, M_r = 587.49, monoclinic, P2$_1$/n (No. 14), a = 9.9379(4) Å, b = 21.2168(8) Å, c = 12.8378(5) Å, β = 93.567(4)°, α = γ = 90°, V = 2701.60(19) Å^3, T = 120.0 K, Z = 4, Z' = 1, μ(MoK$_\alpha$) = 1.558, 68991 reflections measured, 9729 unique (R_{int} = 0.0751) which were used in all calculations. The final wR_2 was 0.1184 (all data) and R_1 was 0.0504 (I > 2(I)).

**Compound**	**SC16010**
Formula	$C_{35}H_{27}BrN_2O_2$
$D_{calc.}$/ g cm^{-3}	1.444
μ/mm^{-1}	1.558
Formula Weight	587.49
Colour	clear colourless
Shape	prism
Size/mm^3	0.57×0.30×0.21
T/K	120.0
Crystal System	monoclinic
Space Group	P2$_1$/n
a/Å	9.9379(4)
b/Å	21.2168(8)
c/Å	12.8378(5)
α/°	90
β/°	93.567(4)
γ/°	90
V/Å^3	2701.60(19)
Z	4
Z'	1
Wavelength/Å	0.71073
Radiation type	MoK$_\alpha$
Θ_{min}/°	3.167
Θ_{max}/°	33.010
Measured Refl.	68991
Independent Refl.	9729
Reflections Used	6901
R_{int}	0.0751
Parameters	469
Restraints	0
Largest Peak	0.555
Deepest Hole	-0.830
GooF	1.019
wR_2 (all data)	0.1184
wR_2	0.1049
R_1 (all data)	0.0840
R_1	0.0504

Structure Quality Indicators

**Reflections:**	d min (Mo) 0.65	I/σ 13.2	Rint 7.51%	complete at 2θ=61° 96%
**Refinement:**	Shift -0.002	Max Peak 0.6	Min Peak -0.8	GooF 1.019

A clear colourless prism-shaped crystal with dimensions 0.57×0.30×0.21 mm^3 was mounted on a MITIGEN holder in Paratone oil.X-ray diffraction data were collected using a Rigaku Oxford Diffraction SuperNova diffractometer equipped with a Oxford Cryosystems Cryostream 700+ low-temperature device, operating at T = 120.0 K.

Data were measured using ω scans scans of 1.0 ° per frame for 5.0 s using MoK_α radiation (micro-focus sealed X-ray tube, 50 kV, 0.8 mA). The total number of runs and images was based on the strategy calculation from the program CrysAlisPro (Agilent).The maximum resolution achieved was Θ = 33.010. °

Cell parameters were retrieved using the CrysAlisPro (Agilent) software and refined using CrysAlisPro (Agilent) on 12687 reflections, 18 % of the observed reflections. Data reduction was performed using the CrysAlisPro (Agilent) software which corrects for Lorentz polarisation. The final completeness is 99.80 out to 33.010 in Θ. The absorption coefficient μ of this material is 1.558 at this wavelength (λ = 0.71073) and the minimum and maximum transmissions are 0.458 and 1.000.

The structure was solved in the space group $P2_1/n$ (# 14) by Direct Methods using the **ShelXS** (Sheldrick, 2008) structure solution program and refined by Least Squares using version 2014/7 of **ShelXL** (Sheldrick, 2015). All non-hydrogen atoms were refined anisotropically. Hydrogen atom positions were calculated geometrically and refined using the riding model.

_*refine_special_details*: H atoms were located in a difference Fourier map and freely refined.

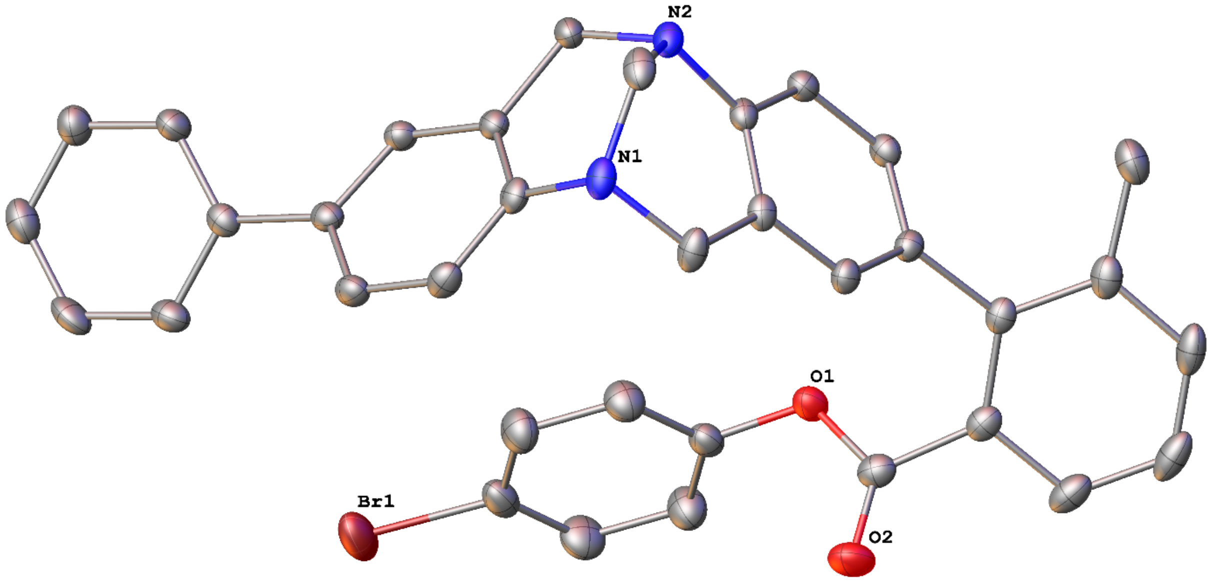

Figure S7: The molecular structure of SC16010. Displacement ellipsoids are at the 50% probability level and H atoms are not shown.

Data Plots: Diffraction Data

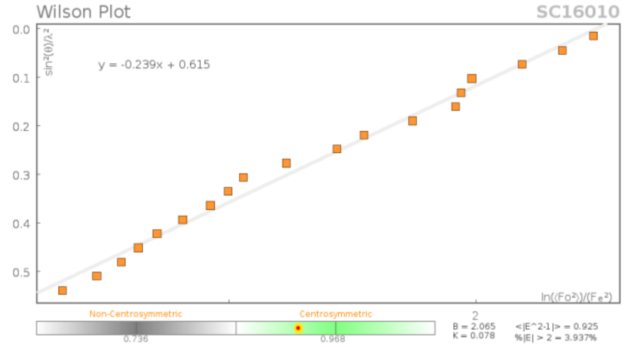

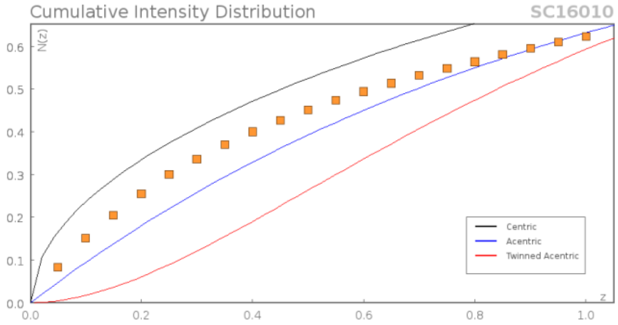

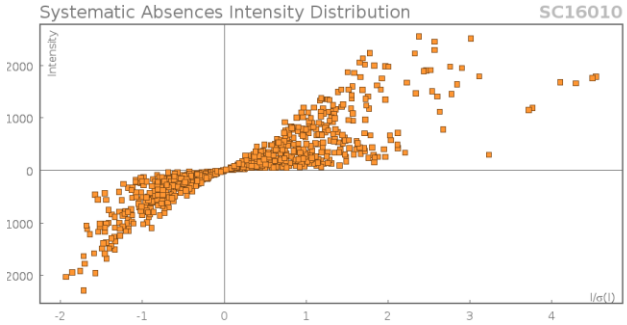

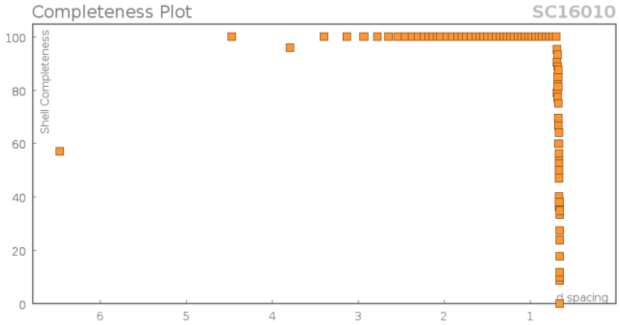

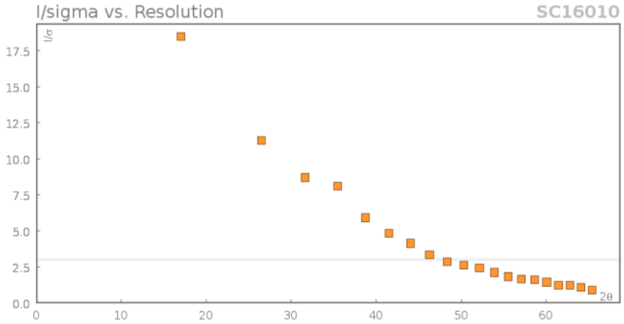

Data Plots: Refinement and Data

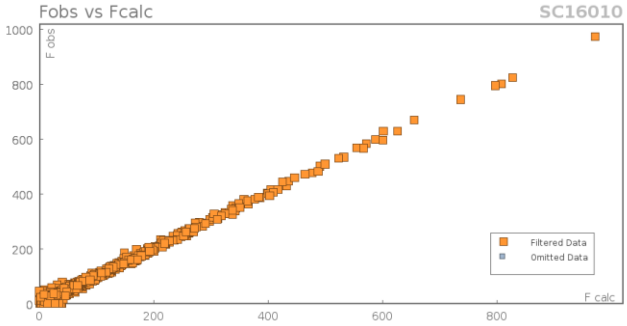

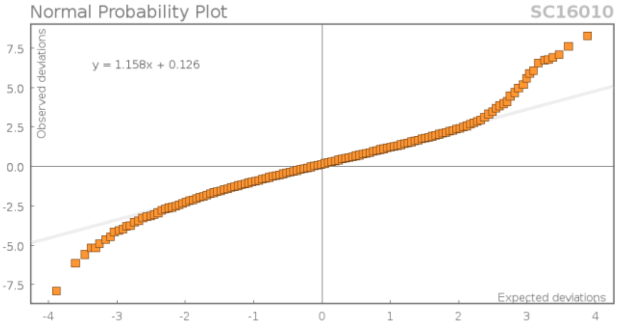

Reflection Statistics

Total reflections (after filtering)	70089	Unique reflections	9729
Completeness	0.956	Mean I/σ	13.2
hkl_{max} collected	(15, 32, 18)	hkl_{min} collected	(-14, -32, -19)
hkl_{max} used	(15, 32, 19)	hkl_{min} used	(-14, 0, 0)
Lim d_{max} collected	100.0	Lim d_{min} collected	0.36
d_{max} used	6.43	d_{min} used	0.65
Friedel pairs	16811	Friedel pairs merged	1

Inconsistent equivalents	0	R_{int}	0.0751
R_{sigma}	0.0535	Intensity transformed	0
Omitted reflections	0	Omitted by user (OMIT hkl)	0
Multiplicity	(13820, 13179, 5949, 1882, 539, 218, 53, 18, 2)	Maximum multiplicity	22
Removed systematic absences	1098	Filtered off (Shel/OMIT)	0

Table S37: Fractional Atomic Coordinates (×10[4]) and Equivalent Isotropic Displacement Parameters (Å[2]×10[3]) for **SC16010.** U_{eq} is defined as 1/3 of the trace of the orthogonalised U_{ij}.

Atom	x	y	z	U_{eq}
Br1	1489.6(2)	5729.9(2)	8416.9(2)	36.90(8)
O1	3582.8(14)	3703.1(6)	11404.2(10)	23.5(3)
O2	5051.8(17)	4352.7(7)	12274.4(13)	33.6(4)
N1	6759.2(16)	2986.1(8)	7827.7(12)	20.7(3)
N2	5170.0(16)	2129.1(7)	8028.8(11)	19.6(3)
C1	4122(2)	2406.4(9)	7307.0(14)	19.1(3)
C2	4429.5(18)	3085.4(8)	7034.6(13)	16.6(3)
C3	5681.3(18)	3354.9(9)	7339.2(13)	18.5(3)
C4	5921.9(19)	3984.1(9)	7092.0(14)	21.1(4)
C5	4974(2)	4329.8(9)	6498.7(14)	20.6(4)
C6	3745.5(19)	4055.8(9)	6132.3(13)	18.2(3)
C7	3487.6(18)	3437.5(9)	6431.8(13)	17.6(3)
C8	2786.4(19)	4397.0(9)	5399.4(14)	18.7(3)
C9	2035(2)	4063.8(9)	4622.7(15)	21.8(4)
C10	1166(2)	4371.8(10)	3902.8(16)	25.2(4)
C11	1040(2)	5022.8(10)	3938.9(16)	27.1(4)
C12	1779(2)	5357.7(10)	4703.8(17)	27.9(4)
C13	2637(2)	5051.8(9)	5433.9(16)	23.6(4)
C14	6489(2)	2313.4(10)	7672.6(15)	24.0(4)
C15	6980.8(19)	3097.5(11)	8961.1(14)	22.6(4)
C16	5967.1(17)	2746.4(9)	9579.6(13)	17.4(3)
C17	5061.2(18)	2317.1(8)	9094.8(13)	16.9(3)
C18	4071.1(18)	2038.1(9)	9667.8(14)	18.0(3)
C19	4028.8(18)	2151.0(9)	10728.6(13)	17.5(3)
C20	4982.5(17)	2549.4(8)	11240.2(13)	16.0(3)
C21	5919.7(18)	2851.0(9)	10651.9(13)	17.6(3)
C22	5050.6(17)	2657.8(9)	12393.1(13)	17.4(3)
C23	5335.1(19)	2153.3(10)	13090.8(14)	22.5(4)
C24	5607(2)	2288.4(12)	14154.6(15)	27.8(4)
C25	5573(2)	2900.0(12)	14529.4(15)	29.7(5)
C26	5251(2)	3392.2(11)	13859.6(15)	26.0(4)
C27	4978.4(18)	3272.8(9)	12791.7(14)	19.6(3)
C28	5338(2)	1475.7(10)	12746.1(18)	27.7(4)
C29	4589.8(19)	3834.8(9)	12143.0(14)	21.0(4)
C30	3152(2)	4197.5(9)	10734.4(15)	22.0(4)
C31	2258(2)	4640.7(11)	11068.9(17)	30.5(5)
C32	1769(2)	5102.8(11)	10374.3(18)	33.1(5)
C33	2198(2)	5109.9(10)	9370.4(16)	26.3(4)
C34	3098(2)	4670.2(11)	9034.6(17)	30.3(4)
C35	3584(2)	4205.5(11)	9732.1(17)	29.0(4)

Table S38: Anisotropic Displacement Parameters (×10[4]) **SC16010.** The anisotropic displacement factor exponent takes the form: $-2\pi^2[h^2a^{*2} \times U_{11}+ \ldots +2hka^* \times b^* \times U_{12}]$

Atom	U_{11}	U_{22}	U_{33}	U_{23}	U_{13}	U_{12}
Br1	34.80(13)	29.35(12)	45.05(14)	13.01(10)	-9.55(10)	-1.59(9)
O1	23.8(7)	19.7(7)	26.2(7)	2.2(5)	-3.3(5)	-3.2(5)
O2	37.7(9)	23.3(8)	38.9(9)	-4.6(6)	-3.9(7)	-7.5(6)
N1	17.4(7)	29.4(9)	15.8(7)	4.3(6)	4.5(5)	3.2(6)
N2	23.0(8)	20.8(8)	15.2(7)	0.8(6)	3.9(5)	5.1(6)
C1	25.5(9)	15.7(8)	16.0(8)	0.8(6)	0.8(7)	0.2(7)

Atom	U_{11}	U_{22}	U_{33}	U_{23}	U_{13}	U_{12}
C2	19.9(8)	16.3(8)	13.9(7)	-0.4(6)	2.6(6)	-0.2(6)
C3	19.1(8)	24.0(9)	12.6(7)	1.3(6)	3.4(6)	0.6(7)
C4	18.6(9)	26.8(10)	18.0(8)	0.1(7)	1.6(6)	-6.5(7)
C5	24.4(9)	17.7(9)	20.0(8)	0.1(7)	3.4(7)	-4.2(7)
C6	21.8(9)	16.4(8)	16.8(8)	-1.5(6)	3.6(6)	-0.4(7)
C7	17.9(8)	16.8(8)	18.2(8)	-1.2(6)	1.1(6)	-1.9(7)
C8	19.1(8)	17.4(8)	20.3(8)	1.6(6)	5.5(6)	0.2(7)
C9	25.0(9)	16.1(8)	24.1(9)	0.4(7)	-0.5(7)	1.3(7)
C10	24.7(10)	25.4(10)	25.4(9)	4.0(8)	-0.7(8)	0.3(8)
C11	27.3(10)	26.3(10)	28(1)	8.7(8)	4.8(8)	5.4(8)
C12	35.2(11)	14.7(9)	34.8(11)	3.8(8)	9.3(9)	5.6(8)
C13	28.8(10)	17.0(9)	25.6(9)	-1.4(7)	6.2(8)	-0.9(8)
C14	26.5(10)	27.7(10)	18.5(8)	3.0(7)	7.7(7)	9.3(8)
C15	16.0(8)	35.4(11)	16.6(8)	5.2(7)	2.2(6)	-2.1(8)
C16	14.5(8)	21.4(8)	16.6(8)	3.5(6)	3.3(6)	3.4(6)
C17	19.4(8)	16.6(8)	14.9(7)	1.6(6)	3.4(6)	4.0(6)
C18	18.9(8)	16.0(8)	19.0(8)	0.4(6)	1.2(6)	-0.1(7)
C19	17.9(8)	17.4(8)	17.6(8)	2.0(6)	4.9(6)	-0.7(7)
C20	15.9(8)	17.8(8)	14.5(7)	-0.1(6)	1.5(6)	2.0(6)
C21	15.4(8)	21.3(9)	16.1(7)	2.2(6)	0.7(6)	0.5(7)
C22	13.1(7)	23.6(9)	15.7(7)	1.7(6)	3.0(6)	-0.6(6)
C23	16.7(8)	31.4(10)	19.6(8)	6.1(7)	3.5(6)	-1.2(7)
C24	20.5(9)	44.7(13)	18.4(8)	11.4(8)	1.4(7)	-2.3(9)
C25	20.1(9)	54.2(14)	14.8(8)	-0.5(9)	1.2(7)	-7.5(9)
C26	21.2(9)	37.8(12)	19.2(9)	-7.0(8)	3.2(7)	-6.7(8)
C27	15.4(8)	25.9(9)	17.8(8)	-1.7(7)	3.2(6)	-2.9(7)
C28	28.1(11)	26.9(10)	28.6(10)	9.7(8)	4.6(8)	3.5(9)
C29	19.1(8)	23.6(9)	20.8(8)	-3.2(7)	5.3(6)	-2.4(7)
C30	23.0(9)	18.1(9)	24.7(9)	1.3(7)	0.0(7)	-2.2(7)
C31	35.9(12)	30.0(11)	26.5(10)	2.0(8)	8.4(8)	5.5(9)
C32	32.6(12)	29.3(11)	37.8(12)	-0.9(9)	4.7(9)	10.0(9)
C33	26.1(10)	20.0(9)	32.2(10)	5.1(8)	-3.6(8)	-3.1(8)
C34	34.1(11)	31.7(11)	25.9(10)	5.3(8)	7.5(8)	1.2(9)
C35	30.8(11)	27.1(11)	29.7(10)	0.1(8)	6.6(8)	5.2(9)

Table S39: Bond Lengths in Å for **SC16010**.

Atom	Atom	Length/Å
Br1	C33	1.902(2)
O1	C29	1.364(2)
O1	C30	1.406(2)
O2	C29	1.199(2)
N1	C3	1.439(2)
N1	C14	1.464(3)
N1	C15	1.477(2)
N2	C1	1.473(2)
N2	C14	1.468(3)
N2	C17	1.436(2)
C1	C2	1.518(3)
C2	C3	1.403(3)
C2	C7	1.394(2)
C3	C4	1.396(3)
C4	C5	1.385(3)
C5	C6	1.406(3)
C6	C7	1.395(3)
C6	C8	1.485(3)
C8	C9	1.400(3)
C8	C13	1.398(3)
C9	C10	1.388(3)
C10	C11	1.388(3)
C11	C12	1.385(3)

Atom	Atom	Length/Å
C12	C13	1.388(3)
C15	C16	1.517(3)
C16	C17	1.399(3)
C16	C21	1.398(2)
C17	C18	1.396(3)
C18	C19	1.386(2)
C19	C20	1.402(2)
C20	C21	1.391(2)
C20	C22	1.495(2)
C22	C23	1.413(3)
C22	C27	1.405(3)
C23	C24	1.405(3)
C23	C28	1.504(3)
C24	C25	1.385(3)
C25	C26	1.378(3)
C26	C27	1.404(3)
C27	C29	1.491(3)
C30	C31	1.380(3)
C30	C35	1.382(3)
C31	C32	1.392(3)
C32	C33	1.382(3)
C33	C34	1.380(3)
C34	C35	1.398(3)

Table S40: Bond Angles in ° for **SC16010**.

Atom	Atom	Atom	Angle/°
C29	O1	C30	117.05(15)
C3	N1	C14	110.23(15)
C3	N1	C15	113.81(15)
C14	N1	C15	107.75(15)
C14	N2	C1	107.92(14)
C17	N2	C1	113.35(14)
C17	N2	C14	110.15(15)
N2	C1	C2	112.31(15)
C3	C2	C1	120.69(16)
C7	C2	C1	119.92(16)
C7	C2	C3	119.31(16)
C2	C3	N1	121.55(17)
C4	C3	N1	119.06(16)
C4	C3	C2	119.20(17)
C5	C4	C3	120.74(17)
C4	C5	C6	120.79(17)
C5	C6	C8	121.32(17)
C7	C6	C5	117.85(17)
C7	C6	C8	120.74(16)
C2	C7	C6	121.90(17)
C9	C8	C6	119.87(16)
C13	C8	C6	121.97(17)
C13	C8	C9	118.12(18)
C10	C9	C8	121.24(18)
C11	C10	C9	120.0(2)
C12	C11	C10	119.26(19)
C11	C12	C13	121.00(19)
C12	C13	C8	120.35(19)
N1	C14	N2	112.16(15)
N1	C15	C16	111.96(16)
C17	C16	C15	121.12(15)
C21	C16	C15	120.11(16)
C21	C16	C17	118.77(16)
C16	C17	N2	121.35(16)
C18	C17	N2	118.79(16)
C18	C17	C16	119.77(16)
C19	C18	C17	120.65(17)
C18	C19	C20	120.23(16)
C19	C20	C22	122.95(16)
C21	C20	C19	118.61(15)
C21	C20	C22	118.43(15)
C20	C21	C16	121.75(17)
C23	C22	C20	120.40(17)
C27	C22	C20	120.21(16)
C27	C22	C23	119.03(16)
C22	C23	C28	122.78(17)
C24	C23	C22	118.72(19)
C24	C23	C28	118.50(18)
C25	C24	C23	121.42(19)
C26	C25	C24	120.20(18)
C25	C26	C27	119.7(2)
C22	C27	C29	123.81(16)
C26	C27	C22	120.81(18)
C26	C27	C29	115.37(18)
O1	C29	C27	112.20(16)
O2	C29	O1	122.92(18)
O2	C29	C27	124.80(18)
C31	C30	O1	119.70(18)
C31	C30	C35	121.64(19)
C35	C30	O1	118.57(18)
C30	C31	C32	119.1(2)
C33	C32	C31	119.3(2)
C32	C33	Br1	118.98(17)
C34	C33	Br1	119.19(16)
C34	C33	C32	121.8(2)
C33	C34	C35	118.8(2)
C30	C35	C34	119.4(2)

Table S41: Torsion Angles in ° for **SC16010**.

Atom	Atom	Atom	Atom	Angle/°
Br1	C33	C34	C35	-178.22(17)
O1	C30	C31	C32	-175.7(2)
O1	C30	C35	C34	175.94(19)
N1	C3	C4	C5	171.28(16)
N1	C15	C16	C17	7.0(3)
N1	C15	C16	C21	-172.64(16)
N2	C1	C2	C3	8.7(2)
N2	C1	C2	C7	-174.49(15)
N2	C17	C18	C19	172.56(16)
C1	N2	C14	N1	69.85(19)
C1	N2	C17	C16	-106.58(19)
C1	N2	C17	C18	76.8(2)
C1	C2	C3	N1	6.1(3)
C1	C2	C3	C4	-

Atom	Atom	Atom	Atom	Angle/°
				178.86(16)
C1	C2	C7	C6	-177.75(16)
C2	C3	C4	C5	-3.9(3)
C3	N1	C14	N2	-54.48(19)
C3	N1	C15	C16	79.4(2)
C3	C2	C7	C6	-0.9(3)
C3	C4	C5	C6	0.1(3)
C4	C5	C6	C7	3.3(3)
C4	C5	C6	C8	-173.12(17)
C5	C6	C7	C2	-2.9(3)
C5	C6	C8	C9	144.33(19)
C5	C6	C8	C13	-33.4(3)
C6	C8	C9	C10	-177.66(18)
C6	C8	C13	C12	176.83(18)
C7	C2	C3	N1	-170.78(16)
C7	C2	C3	C4	4.3(3)
C7	C6	C8	C9	-32.0(3)
C7	C6	C8	C13	150.26(19)
C8	C6	C7	C2	173.54(16)
C8	C9	C10	C11	0.6(3)
C9	C8	C13	C12	-1.0(3)
C9	C10	C11	C12	-0.5(3)
C10	C11	C12	C13	-0.3(3)
C11	C12	C13	C8	1.0(3)
C13	C8	C9	C10	0.2(3)
C14	N1	C3	C2	16.0(2)
C14	N1	C3	C4	-159.06(16)
C14	N1	C15	C16	-43.2(2)
C14	N2	C1	C2	-43.6(2)
C14	N2	C17	C16	14.4(2)
C14	N2	C17	C18	-162.19(16)
C15	N1	C3	C2	-105.2(2)
C15	N1	C3	C4	79.8(2)
C15	N1	C14	N2	70.26(19)
C15	C16	C17	N2	8.4(3)
C15	C16	C17	C18	-175.00(17)
C15	C16	C21	C20	178.41(17)
C16	C17	C18	C19	-4.1(3)
C17	N2	C1	C2	78.70(19)
C17	N2	C14	N1	-54.37(19)
C17	C16	C21	C20	-1.2(3)
C17	C18	C19	C20	0.1(3)
C18	C19	C20	C21	3.3(3)
C18	C19	C20	C22	-175.85(17)
C19	C20	C21	C16	-2.7(3)
C19	C20	C22	C23	62.7(2)
C19	C20	C22	C27	-124.26(19)
C20	C22	C23	C24	169.78(17)
C20	C22	C23	C28	-11.2(3)
C20	C22	C27	C26	-169.95(17)
C20	C22	C27	C29	11.4(3)
C21	C16	C17	N2	-

Atom	Atom	Atom	Atom	Angle/˚
				171.97(16)
C21	C16	C17	C18	4.6(3)
C21	C20	C22	C23	-116.4(2)
C21	C20	C22	C27	56.6(2)
C22	C20	C21	C16	176.44(16)
C22	C23	C24	C25	1.4(3)
C22	C27	C29	O1	38.5(2)
C22	C27	C29	O2	-144.7(2)
C23	C22	C27	C26	3.2(3)
C23	C22	C27	C29	-175.46(17)
C23	C24	C25	C26	0.8(3)
C24	C25	C26	C27	-1.0(3)
C25	C26	C27	C22	-1.0(3)
C25	C26	C27	C29	177.77(17)
C26	C27	C29	O1	-140.19(17)
C26	C27	C29	O2	36.5(3)
C27	C22	C23	C24	-3.4(3)
C27	C22	C23	C28	175.66(18)
C28	C23	C24	C25	-177.66(19)
C29	O1	C30	C31	-80.9(2)
C29	O1	C30	C35	102.6(2)
C30	O1	C29	O2	4.3(3)
C30	O1	C29	C27	-178.91(15)
C30	C31	C32	C33	-0.5(4)
C31	C30	C35	C34	-0.5(3)
C31	C32	C33	Br1	178.43(18)
C31	C32	C33	C34	0.1(4)
C32	C33	C34	C35	0.1(3)
C33	C34	C35	C30	0.1(3)
C35	C30	C31	C32	0.8(3)

Table S42: Hydrogen Fractional Atomic Coordinates (×10[4]) and Equivalent Isotropic Displacement Parameters (Å[2]×10[3]) for **SC16010.** U_{eq} is defined as 1/3 of the trace of the orthogonalised U_{ij}.

Atom	x	y	z	U_{eq}
H1A	3280(20)	2377(10)	7618(17)	20(5)
H1B	4060(20)	2164(10)	6646(17)	18(5)
H4	6780(20)	4155(10)	7287(17)	19(5)
H5	5210(20)	4750(11)	6275(17)	22(6)
H7	2630(20)	3239(11)	6222(17)	20(5)
H9	2130(20)	3628(12)	4583(17)	25(6)
H10	680(30)	4143(11)	3383(19)	28(6)
H11	430(20)	5231(12)	3427(19)	30(6)
H12	1670(30)	5790(13)	4730(20)	40(7)
H13	3090(30)	5264(12)	5979(19)	32(6)
H14A	7200(20)	2071(10)	8062(16)	14(5)
H14B	6460(20)	2210(11)	6948(19)	28(6)
H15A	6870(20)	3555(11)	9095(16)	15(5)
H15B	7910(20)	2957(11)	9196(17)	23(6)
H18	3440(20)	1769(10)	9342(16)	17(5)
H19	3370(20)	1951(11)	11085(17)	19(5)
H21	6550(20)	3153(10)	10978(16)	15(5)
H24	5860(20)	1955(12)	14611(18)	27(6)
H25	5770(20)	2997(12)	15230(20)	31(6)
H26	5190(20)	3809(11)	14094(17)	19(5)
H28A	5730(30)	1422(12)	12100(20)	37(7)
H28B	5850(30)	1229(14)	13220(20)	49(8)
H28C	4410(30)	1314(13)	12700(20)	46(8)
H31	1990(30)	4624(13)	11760(20)	41(7)
H32	1160(30)	5406(13)	10590(20)	41(7)
H35	4170(30)	3889(13)	9490(20)	44(8)
H34	3370(30)	4674(13)	8370(20)	40(7)

```
#==============================================================================
# PLATON/CHECK-(150616) versus check.def version of 160610 for Entry: sc16010
# Data: SC16010.cif - Type: CIF                  Bond Precision   C-C = 0.0028 A
# Refl: SC16010.fcf - Type: LIST4                                 Temp = 120 K
# X-ray MoKa              R(int) = 0.075,  wR2/R(int) =  1.6,  Nref/Npar =  20.7
# Cell   9.9379(4)  21.2168(8)  12.8378(5)          90    93.567(4)          90
# Wavelength 0.71073    Volume Reported   2701.60(19)   Calculated   2701.61(18)
# SpaceGroup from Symmetry P 21/n     Hall: -P 2yn                monoclinic
#                 Reported P 1 21/n 1       -P 2yn                monoclinic
# MoietyFormula C35 H27 Br N2 O2
#      Reported C35 H27 Br N2 O2
#    SumFormula C35 H27 Br N2 O2
#      Reported C35 H27 Br N2 O2
# Mr        =    587.49[Calc],    587.49[Rep]
# Dx,gcm-3  =     1.444[Calc],     1.444[Rep]
# Z         =         4[Calc],         4[Rep]
# Mu (mm-1) =     1.558[Calc],     1.558[Rep]
# F000      =    1208.0[Calc],    1208.0[Rep]  or F000' =   1207.29[Calc]
# Reported   T Limits: Tmin=0.458              Tmax=1.000  AbsCorr = GAUSSIAN
# Calculated T Limits: Tmin=0.580 Tmin'=0.406  Tmax=0.720
# Reported   Hmax= 15, Kmax= 32, Lmax= 19, Nref=   9729        , Th(max)= 33.010
# Obs in FCF Hmax= 15, Kmax= 32, Lmax= 19, Nref=   9729[  9729], Th(max)= 33.010
# Calculated Hmax= 15, Kmax= 32, Lmax= 19, Nref=  10184        , Ratio  =  0.955
# Reported   Rho(min) = -0.83, Rho(max) =  0.56 e/Ang**3 (From CIF)
# Calculated Rho(min) = -0.76, Rho(max) =  0.49 e/Ang**3 (From CIF+FCF data)
# w=1/[sigma**2(Fo**2)+(0.0448P)**2+  1.8661P], P=(Fo**2+2*Fc**2)/3
# R= 0.0504(  6901), wR2= 0.1184(  9729), S = 1.019      (From CIF+FCF data)
# R= 0.0504(  6901), wR2= 0.1184(  9729), S = 1.019      (From FCF data only)
# R= 0.0504(  6901), wR2= 0.1184(  9729), S = 1.019, Npar=  469
#==============================================================================
For Documentation: http://http://www.platonsoft.nl/CIF-VALIDATION.pdf
#==============================================================================

#==============================================================================
>>> The Following Improvement and Query ALERTS were generated - (Acta-Mode) <<<
#==============================================================================
Format: alert-number_ALERT_alert-type_alert-level text

906_ALERT_3_C Large K value in the Analysis of Variance ......      5.715 Check
910_ALERT_3_C Missing # of FCF Reflection(s) Below Theta(Min)           9 Note
#==============================================================================
164_ALERT_4_G Nr. of Refined C-H H-Atoms in Heavy-Atom Struct.         27 Note
912_ALERT_4_G Missing # of FCF Reflections Above STh/L=  0.600        446 Note
913_ALERT_3_G Missing # of Very Strong Reflections in FCF ....          1 Note
978_ALERT_2_G Number C-C Bonds with Positive Residual Density          19 Note
#==============================================================================

ALERT_Level and ALERT_Type Summary
==================================
2 ALERT_Level_C = Check. Ensure it is Not caused by an Omission or Oversight
4 ALERT_Level_G = General Info/Check that it is not Something Unexpected

1 ALERT_Type_2 Indicator that the Structure Model may be Wrong or Deficient.
3 ALERT_Type_3 Indicator that the Structure Quality may be Low.
2 ALERT_Type_4 Improvement, Methodology, Query or Suggestion.
#==============================================================================

0 Missing  Experimental  Info Issue(s) (Out of  62 Tests) -  100 % Satisfied
0 Experimental  Data  Related Issue(s) (Out of  28 Tests) -  100 % Satisfied
3 Structural  Model   Related Issue(s) (Out of 126 Tests) -   98 % Satisfied
3 Unresolved or to be Checked Issue(s) (Out of 247 Tests) -   99 % Satisfied

#==============================================================================
```

Compound (±)-1I CCDC Deposition Number 2244210

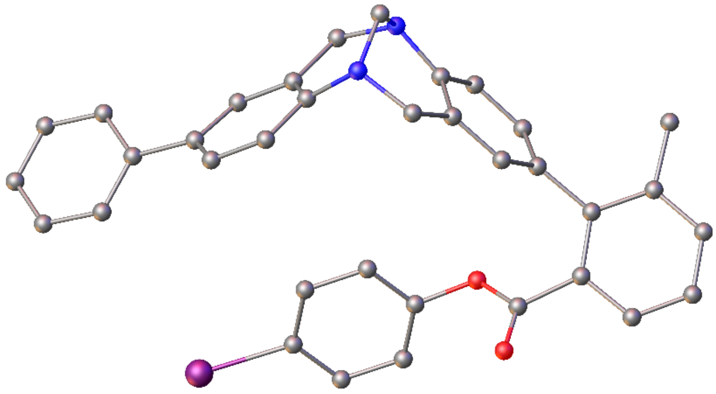

Experimental. Single colourless plate-shaped crystals of (**sc16005**) were recrystallised from DCM by slow evaporation. A suitable crystal (0.65×0.07×0.05) mm^3 was selected and mounted on a MITIGEN holder in Paratone oil on a Rigaku Oxford Diffraction XCalibur diffractometer. The crystal was kept at T = 170.0 K during data collection. Using **Olex2** (Dolomanov et al., 2009), the structure was solved with the **ShelXS** (Sheldrick, 2008) structure solution program, using the Direct Methods solution method. The model was refined with version 2014/7 of **ShelXL** (Sheldrick, 2015) using Least Squares minimisation.

Crystal Data. $C_{35}H_{27}IN_2O_2$, M_r = 634.48, monoclinic, P2$_1$/n (No. 14), a = 10.1218(4) Å, b = 21.3189(7) Å, c = 12.8204(5) Å, β = 92.701(4)°, α = γ = 90°, V = 2763.38(18) Å^3, T = 170.0 K, Z = 4, Z' = 1, μ(MoK$_\alpha$) = 1.195, 53054 reflections measured, 5055 unique (R_{int} = 0.0570) which were used in all calculations. The final wR_2 was 0.1344 (all data) and R_1 was 0.0801 (I > 2(I)).

**Compound**	**sc16005**
Formula	$C_{35}H_{27}IN_2O_2$
$D_{calc.}$/ g cm^{-3}	1.525
μ/mm^{-1}	1.195
Formula Weight	634.48
Colour	colourless
Shape	plate
Size/mm^3	0.65×0.07×0.05
T/K	170.0
Crystal System	monoclinic
Space Group	P2$_1$/n
a/Å	10.1218(4)
b/Å	21.3189(7)
c/Å	12.8204(5)
α/°	90
β/°	92.701(4)
γ/°	90
V/Å^3	2763.38(18)
Z	4
Z'	1
Wavelength/Å	0.71073
Radiation type	MoK$_\alpha$
Θ_{min}/°	3.153
Θ_{max}/°	25.350
Measured Refl.	53054
Independent Refl.	5055
Reflections Used	4731
R_{int}	0.0570
Parameters	362
Restraints	0
Largest Peak	1.138
Deepest Hole	-0.840
GooF	1.361
wR_2 (all data)	0.1344
wR_2	0.1312
R_1 (all data)	0.0884
R_1	0.0801

Structure Quality Indicators

**Reflections:**	d min (Mo) 0.83	I/σ 19.8	Rint 5.70%	complete 100%
**Refinement:**	Shift 0.000	Max Peak 1.1	Min Peak -0.8	GooF 1.361

A colourless plate-shaped crystal with dimensions 0.65×0.07×0.05 mm^3 was mounted on a MITIGEN holder in Paratone oil.X-ray diffraction data were collected using a Rigaku Oxford Diffraction XCalibur diffractometer equipped with a Oxford Cryosystems Desktop Cooler low-temperature device, operating at T = 170.0 K.

Data were measured using ω scans scans of 0.5 ° per frame for 30.0 s using MoK_α radiation (fine-focus sealed X-ray tube, 50 kV, 0.8 mA). The total number of runs and images was based on the strategy calculation from the program CrysAlisPro (Agilent).The maximum resolution achieved was Θ = 25.350. °

Cell parameters were retrieved using the CrysAlisPro (Agilent) software and refined using CrysAlisPro (Agilent) on 11641 reflections, 22 % of the observed reflections. Data reduction was performed using the CrysAlisPro (Agilent) software which corrects for Lorentz polarisation. The final completeness is 99.80 out to 25.350 in Θ. The absorption coefficient μ of this material is 1.195 at this wavelength (λ = 0.71073) and the minimum and maximum transmissions are 0.9221 and 1.0000.

The structure was solved in the space group P2$_1$/n (# 14) by Direct Methods using the **ShelXS** (Sheldrick, 2008) structure solution program and refined by Least Squares using version 2014/7 of **ShelXL** (Sheldrick, 2015). All non-hydrogen atoms were refined anisotropically. Hydrogen atom positions were calculated geometrically and refined using the riding model.

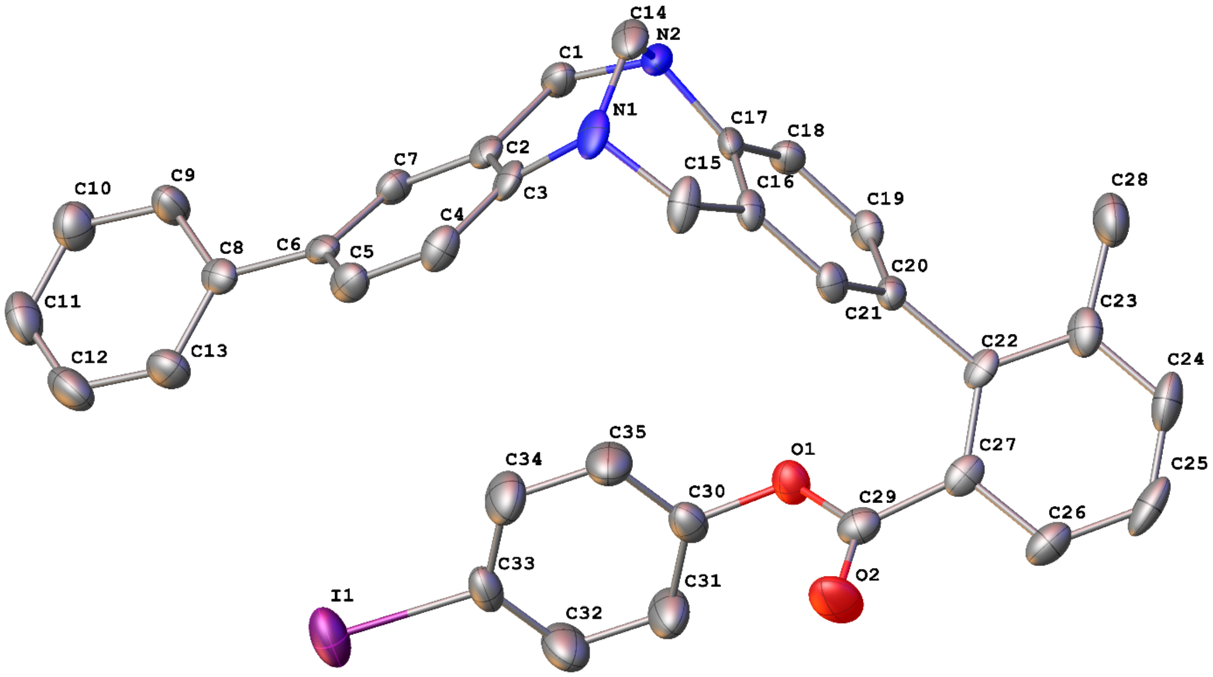

Figure S8: The asymmetric unit of SC16005. Displacement ellipsoids are at the 50% probability level and H atoms are omitted.

Data Plots: Diffraction Data

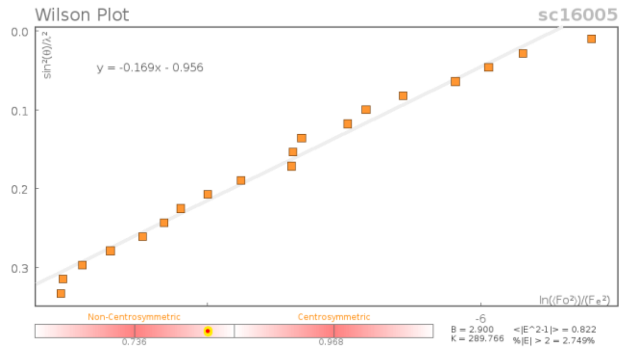

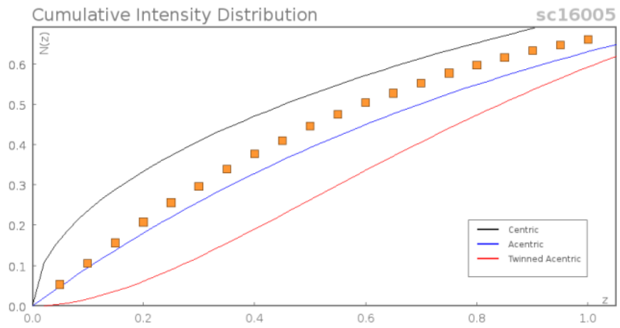

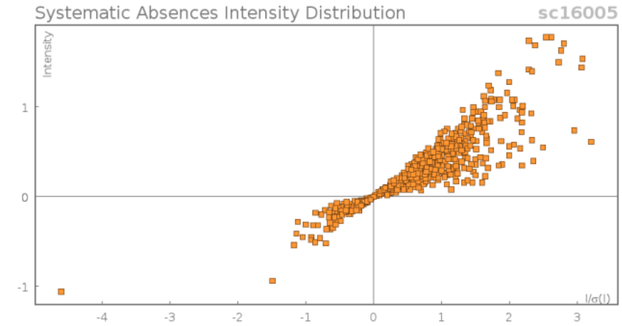

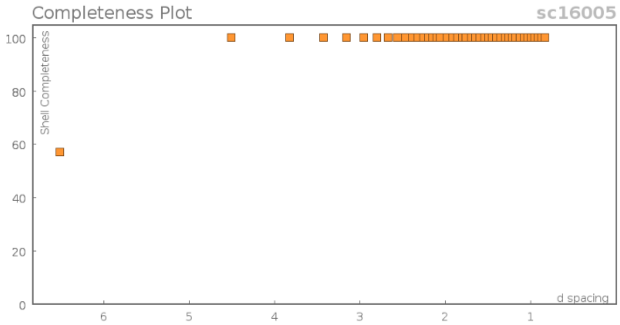

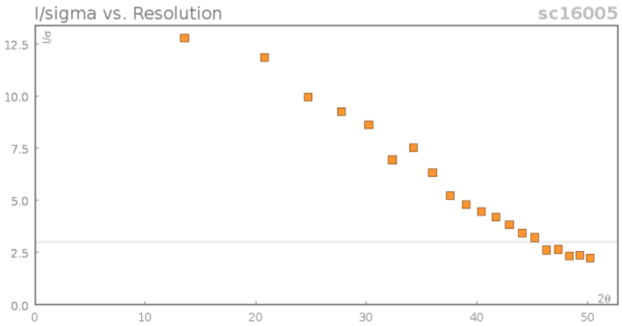

Data Plots: Refinement and Data

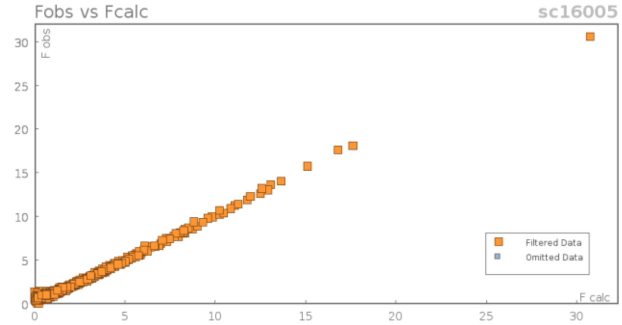

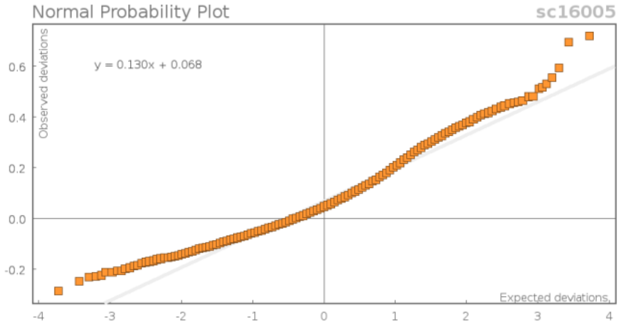

Reflection Statistics

Total reflections (after filtering)	54010	Unique reflections	5055
Completeness	1.0	Mean I/σ	19.84
hkl_{max} collected	(12, 25, 15)	hkl_{min} collected	(-12, -25, -15)
hkl_{max} used	(12, 25, 15)	hkl_{min} used	(-12, 0, 0)
Lim d_{max} collected	100.0	Lim d_{min} collected	0.36
d_{max} used	7.11	d_{min} used	0.83
Friedel pairs	10073	Friedel pairs merged	1

Inconsistent equivalents	0	R_{int}	0.057
R_{sigma}	0.0358	Intensity transformed	0
Omitted reflections	0	Omitted by user (OMIT hkl)	0
Multiplicity	(3478, 7359, 5101, 2245, 1055, 570, 233, 102, 31, 11)	Maximum multiplicity	29
Removed systematic absences	956	Filtered off (Shel/OMIT)	0

Table S43: Fractional Atomic Coordinates (×10[4]) and Equivalent Isotropic Displacement Parameters (Å[2]×10[3]) for **sc16005.** U_{eq} is defined as 1/3 of the trace of the orthogonalised U_{ij}.

Atom	x	y	z	U_{eq}
I1	3545.3(5)	4295.9(2)	6648.4(4)	47.59(17)
O1	1281(4)	6347.1(17)	3590(3)	30.5(9)
O2	-47(5)	5697(2)	2648(4)	49.2(12)
N1	-1780(4)	7077(2)	7195(3)	30.2(11)
N2	-173(5)	7909(2)	6980(3)	26.3(10)
C1	840(6)	7615(2)	7676(4)	26.0(12)
C2	495(5)	6942(2)	7937(4)	21.8(11)
C3	-738(5)	6694(3)	7647(4)	24.5(12)
C4	-999(5)	6070(3)	7884(4)	29.1(13)
C5	-88(5)	5715(3)	8448(4)	28.9(12)
C6	1118(5)	5966(2)	8808(4)	21.2(11)
C7	1396(5)	6574(2)	8511(4)	23.0(11)
C8	2059(5)	5610(2)	9516(4)	24.0(11)
C9	2832(6)	5928(3)	10271(4)	32.0(13)
C10	3685(6)	5611(3)	10956(5)	38.4(15)
C11	3782(6)	4972(3)	10905(5)	40.3(15)
C12	3024(7)	4653(3)	10168(5)	43.4(16)
C13	2175(6)	4967(3)	9469(5)	34.2(14)
C14	-1477(6)	7741(3)	7361(4)	33.1(14)
C15	-2022(5)	6980(3)	6064(4)	32.3(14)
C16	-1016(5)	7316(2)	5431(4)	21.8(11)
C17	-96(5)	7728(2)	5908(4)	21.5(11)
C18	889(5)	7986(2)	5318(4)	23.6(11)
C19	917(5)	7875(2)	4260(4)	22.9(11)
C20	-40(5)	7493(2)	3761(4)	19.7(11)
C21	-976(5)	7218(2)	4358(4)	22.7(11)
C22	-112(5)	7390(2)	2603(4)	20.9(11)
C23	-398(5)	7892(3)	1917(4)	30.6(13)
C24	-669(6)	7764(3)	862(4)	37.7(15)
C25	-636(6)	7161(3)	476(4)	39.5(16)
C26	-314(6)	6670(3)	1130(4)	33.0(14)
C27	-39(5)	6782(3)	2193(4)	24.4(12)
C28	-413(7)	8567(3)	2276(5)	42.2(16)
C29	351(5)	6216(3)	2818(4)	27.8(12)
C30	1715(6)	5852(2)	4247(4)	29.2(13)
C31	2609(7)	5434(3)	3910(5)	45.7(17)
C32	3120(7)	4980(3)	4598(5)	50.8(18)
C33	2709(6)	4957(3)	5594(5)	31.2(13)
C34	1788(7)	5372(3)	5931(5)	45.0(17)
C35	1291(7)	5832(3)	5243(5)	42.9(16)

Table S44: Anisotropic Displacement Parameters (×10[4]) **sc16005.** The anisotropic displacement factor exponent takes the form: $-2\pi^2[h^2a^{*2} \times U_{11} + ... + 2hka^* \times b^* \times U_{12}]$

Atom	U_{11}	U_{22}	U_{33}	U_{23}	U_{13}	U_{12}
I1	47.8(3)	39.4(2)	54.3(3)	18.2(2)	-10.7(2)	-0.3(2)
O1	32(2)	26(2)	32(2)	4.3(16)	-4.6(18)	-4.7(17)
O2	59(3)	28(2)	59(3)	-7(2)	-16(2)	-8(2)
N1	24(2)	50(3)	18(2)	10(2)	10.1(19)	7(2)
N2	38(3)	25(2)	17(2)	0.1(18)	3.6(19)	10(2)
C1	36(3)	22(3)	21(3)	-1(2)	1(2)	-4(2)

Atom	U_{11}	U_{22}	U_{33}	U_{23}	U_{13}	U_{12}
C2	25(3)	27(3)	14(2)	-1(2)	7(2)	2(2)
C3	26(3)	37(3)	11(2)	2(2)	6(2)	1(2)
C4	24(3)	42(3)	22(3)	1(2)	1(2)	-7(3)
C5	36(3)	25(3)	26(3)	1(2)	5(2)	-8(3)
C6	27(3)	20(3)	17(3)	-2(2)	8(2)	1(2)
C7	22(3)	27(3)	19(3)	-3(2)	-2(2)	0(2)
C8	27(3)	24(3)	22(3)	2(2)	10(2)	-2(2)
C9	41(3)	21(3)	34(3)	2(2)	-7(3)	0(2)
C10	37(3)	40(4)	37(3)	4(3)	-5(3)	1(3)
C11	36(3)	43(4)	41(4)	16(3)	2(3)	13(3)
C12	56(4)	26(3)	50(4)	8(3)	13(3)	10(3)
C13	43(4)	23(3)	37(3)	0(2)	3(3)	-2(3)
C14	37(3)	42(3)	21(3)	3(2)	12(3)	17(3)
C15	18(3)	55(4)	24(3)	13(3)	-1(2)	-5(3)
C16	15(3)	29(3)	22(3)	8(2)	0(2)	5(2)
C17	28(3)	20(3)	17(3)	5(2)	2(2)	9(2)
C18	28(3)	19(3)	24(3)	-1(2)	-1(2)	-3(2)
C19	20(3)	25(3)	24(3)	7(2)	6(2)	-1(2)
C20	19(3)	22(3)	18(3)	3(2)	1(2)	4(2)
C21	17(3)	26(3)	24(3)	5(2)	-5(2)	1(2)
C22	14(2)	34(3)	15(3)	0(2)	6(2)	-5(2)
C23	25(3)	41(3)	26(3)	11(3)	7(2)	-1(3)
C24	28(3)	62(4)	23(3)	16(3)	-3(2)	-2(3)
C25	27(3)	78(5)	13(3)	1(3)	3(2)	-10(3)
C26	29(3)	46(4)	24(3)	-8(3)	3(2)	-5(3)
C27	14(3)	38(3)	22(3)	-3(2)	2(2)	-4(2)
C28	48(4)	46(4)	33(3)	16(3)	5(3)	7(3)
C29	26(3)	32(3)	26(3)	-5(2)	7(2)	-2(2)
C30	31(3)	23(3)	34(3)	1(2)	-5(3)	-2(2)
C31	53(4)	53(4)	32(3)	10(3)	15(3)	17(3)
C32	58(5)	46(4)	50(4)	7(3)	13(4)	24(4)
C33	34(3)	25(3)	34(3)	8(2)	-8(3)	-1(3)
C34	50(4)	55(4)	31(3)	11(3)	12(3)	8(3)
C35	47(4)	46(4)	36(4)	0(3)	10(3)	18(3)

Table S45: Bond Lengths in Å for **sc16005**.

Atom	Atom	Length/Å
I1	C33	2.104(5)
O1	C29	1.363(6)
O1	C30	1.408(6)
O2	C29	1.193(7)
N1	C3	1.435(7)
N1	C14	1.461(8)
N1	C15	1.473(7)
N2	C1	1.467(7)
N2	C14	1.473(7)
N2	C17	1.433(6)
C1	C2	1.519(7)
C2	C3	1.389(7)
C2	C7	1.387(7)
C3	C4	1.392(8)
C4	C5	1.373(8)
C5	C6	1.392(7)
C6	C7	1.385(7)
C6	C8	1.492(7)
C8	C9	1.392(8)
C8	C13	1.376(8)
C9	C10	1.378(8)
C10	C11	1.369(9)
C11	C12	1.369(9)

Atom	Atom	Length/Å
C12	C13	1.385(9)
C15	C16	1.513(7)
C16	C17	1.401(7)
C16	C21	1.394(7)
C17	C18	1.392(7)
C18	C19	1.378(7)
C19	C20	1.398(7)
C20	C21	1.377(7)
C20	C22	1.499(7)
C22	C23	1.406(7)
C22	C27	1.402(7)
C23	C24	1.393(8)
C23	C28	1.512(9)
C24	C25	1.379(9)
C25	C26	1.371(9)
C26	C27	1.399(7)
C27	C29	1.491(8)
C30	C31	1.355(8)
C30	C35	1.367(8)
C31	C32	1.392(9)
C32	C33	1.363(9)
C33	C34	1.368(9)
C34	C35	1.398(9)

Table S46: Bond Angles in ° for **sc16005**.

Atom	Atom	Atom	Angle/°
C29	O1	C30	117.8(4)
C3	N1	C14	110.3(4)
C3	N1	C15	113.7(4)
C14	N1	C15	107.7(4)
C1	N2	C14	107.8(4)
C17	N2	C1	113.6(4)
C17	N2	C14	110.0(4)
N2	C1	C2	112.1(4)
C3	C2	C1	120.9(5)
C7	C2	C1	119.9(5)
C7	C2	C3	119.2(5)
C2	C3	N1	121.7(5)
C2	C3	C4	118.8(5)
C4	C3	N1	119.2(5)
C5	C4	C3	120.8(5)
C4	C5	C6	121.2(5)
C5	C6	C8	122.0(5)
C7	C6	C5	117.1(5)
C7	C6	C8	120.8(5)
C6	C7	C2	122.5(5)
C9	C8	C6	119.8(5)
C13	C8	C6	122.2(5)
C13	C8	C9	117.9(5)
C10	C9	C8	121.3(5)
C11	C10	C9	120.1(6)
C10	C11	C12	119.2(6)
C11	C12	C13	121.2(6)
C8	C13	C12	120.3(6)
N1	C14	N2	111.9(4)
N1	C15	C16	112.1(5)
C17	C16	C15	120.9(5)
C21	C16	C15	120.7(5)
C21	C16	C17	118.4(5)
C16	C17	N2	121.6(5)
C18	C17	N2	119.2(5)
C18	C17	C16	119.2(5)
C19	C18	C17	121.1(5)
C18	C19	C20	120.2(5)
C19	C20	C22	122.6(4)
C21	C20	C19	118.4(5)
C21	C20	C22	119.0(4)
C20	C21	C16	122.5(5)
C23	C22	C20	120.5(5)
C27	C22	C20	120.4(5)
C27	C22	C23	118.8(5)
C22	C23	C28	122.6(5)
C24	C23	C22	118.9(6)
C24	C23	C28	118.5(5)
C25	C24	C23	121.6(6)
C26	C25	C24	120.1(5)
C25	C26	C27	119.7(6)
C22	C27	C29	124.4(5)
C26	C27	C22	120.8(5)
C26	C27	C29	114.8(5)
O1	C29	C27	112.3(5)
O2	C29	O1	122.6(5)
O2	C29	C27	125.0(5)
C31	C30	O1	119.8(5)
C31	C30	C35	121.4(5)
C35	C30	O1	118.6(5)
C30	C31	C32	119.3(6)
C33	C32	C31	119.8(6)
C32	C33	I1	119.6(5)
C32	C33	C34	121.1(5)
C34	C33	I1	119.3(4)
C33	C34	C35	118.9(6)
C30	C35	C34	119.5(6)

Table S47: Torsion Angles in ° for **sc16005**.

Atom	Atom	Atom	Atom	Angle/°
I1	C33	C34	C35	176.5(5)
O1	C30	C31	C32	174.3(6)
O1	C30	C35	C34	-175.2(6)
N1	C3	C4	C5	-170.7(5)
N1	C15	C16	C17	-7.5(7)
N1	C15	C16	C21	171.9(5)
N2	C1	C2	C3	-8.5(7)
N2	C1	C2	C7	174.3(4)
N2	C17	C18	C19	-173.3(5)
C1	N2	C14	N1	-69.9(5)
C1	N2	C17	C16	106.3(5)
C1	N2	C17	C18	-76.0(6)
C1	C2	C3	N1	-6.9(7)
C1	C2	C3	C4	178.7(5)
C1	C2	C7	C6	177.3(5)
C2	C3	C4	C5	3.9(8)
C3	N1	C14	N2	54.1(5)
C3	N1	C15	C16	-78.7(6)

Atom	Atom	Atom	Atom	Angle/°
C3	C2	C7	C6	0.1(8)
C3	C4	C5	C6	0.5(8)
C4	C5	C6	C7	-4.4(8)
C4	C5	C6	C8	173.4(5)
C5	C6	C7	C2	4.1(8)
C5	C6	C8	C9	-146.0(5)
C5	C6	C8	C13	32.5(8)
C6	C8	C9	C10	178.2(5)
C6	C8	C13	C12	-177.5(5)
C7	C2	C3	N1	170.3(5)
C7	C2	C3	C4	-4.1(7)
C7	C6	C8	C9	31.7(7)
C7	C6	C8	C13	-149.8(5)
C8	C6	C7	C2	-173.7(5)
C8	C9	C10	C11	-0.2(10)
C9	C8	C13	C12	1.0(9)
C9	C10	C11	C12	0.0(10)
C10	C11	C12	C13	0.7(10)
C11	C12	C13	C8	-1.2(10)
C13	C8	C9	C10	-0.3(9)
C14	N1	C3	C2	-15.4(6)
C14	N1	C3	C4	159.1(5)
C14	N1	C15	C16	43.8(6)
C14	N2	C1	C2	43.7(6)
C14	N2	C17	C16	-14.6(6)
C14	N2	C17	C18	163.1(5)
C15	N1	C3	C2	105.7(6)
C15	N1	C3	C4	-79.9(6)
C15	N1	C14	N2	-70.5(5)
C15	C16	C17	N2	-8.0(7)
C15	C16	C17	C18	174.3(5)
C15	C16	C21	C20	-177.1(5)
C16	C17	C18	C19	4.5(7)
C17	N2	C1	C2	-78.4(6)
C17	N2	C14	N1	54.4(5)
C17	C16	C21	C20	2.3(8)
C17	C18	C19	C20	-0.9(8)
C18	C19	C20	C21	-2.0(7)
C18	C19	C20	C22	175.9(5)
C19	C20	C21	C16	1.3(8)
C19	C20	C22	C23	-64.5(7)
C19	C20	C22	C27	122.4(6)
C20	C22	C23	C24	-169.7(5)
C20	C22	C23	C28	10.8(8)
C20	C22	C27	C26	169.7(5)
C20	C22	C27	C29	-11.5(8)
C21	C16	C17	N2	172.6(4)
C21	C16	C17	C18	-5.1(7)
C21	C20	C22	C23	113.4(6)
C21	C20	C22	C27	-59.6(7)
C22	C20	C21	C16	-176.8(5)
C22	C23	C24	C25	-1.4(9)
C22	C27	C29	O1	-34.5(7)
C22	C27	C29	O2	148.7(6)
C23	C22	C27	C26	-3.4(8)
C23	C22	C27	C29	175.3(5)
C23	C24	C25	C26	-0.8(9)
C24	C25	C26	C27	1.0(9)
C25	C26	C27	C22	1.2(8)
C25	C26	C27	C29	-177.7(5)
C26	C27	C29	O1	144.4(5)
C26	C27	C29	O2	-32.5(8)

Atom	Atom	Atom	Atom	Angle/°
C27	C22	C23	C24	3.5(8)
C27	C22	C23	C28	-176.1(5)
C28	C23	C24	C25	178.1(6)
C29	O1	C30	C31	78.9(7)
C29	O1	C30	C35	-105.9(6)
C30	O1	C29	O2	-4.1(8)
C30	O1	C29	C27	179.0(4)
C30	C31	C32	C33	0.6(11)
C31	C30	C35	C34	-0.1(10)
C31	C32	C33	I1	-177.3(6)
C31	C32	C33	C34	0.6(11)
C32	C33	C34	C35	-1.5(10)
C33	C34	C35	C30	1.2(10)
C35	C30	C31	C32	-0.8(10)

Table S48: Hydrogen Fractional Atomic Coordinates (×10⁴) and Equivalent Isotropic Displacement Parameters (Å²×10³) for **sc16005.** U_{eq} is defined as 1/3 of the trace of the orthogonalised U_{ij}.

Atom	x	y	z	U_{eq}
H1A	939	7861	8330	31
H1B	1699	7623	7337	31
H4	-1818	5888	7652	35
H5	-286	5289	8596	35
H7	2237	6747	8707	28
H9	2771	6371	10315	38
H10	4206	5837	11464	46
H11	4368	4752	11376	48
H12	3082	4209	10135	52
H13	1671	4738	8955	41
H14A	-1492	7837	8116	40
H14B	-2166	7999	6993	40
H15A	-2918	7133	5854	39
H15B	-1991	6525	5911	39
H18	1552	8241	5650	28
H19	1590	8060	3869	27
H21	-1619	6951	4025	27
H24	-883	8100	399	45
H25	-836	7086	-245	47
H26	-277	6255	862	40
H28A	494	8729	2327	63
H28B	-800	8591	2961	63
H28C	-943	8818	1771	63
H31	2885	5451	3213	55
H32	3754	4687	4374	61
H34	1491	5347	6622	54
H35	664	6129	5465	52

```
#===============================================================================
# PLATON/CHECK-(150616) versus check.def version of 160610 for Entry: sc16005
# Data: sc16005.cif - Type: CIF                  Bond Precision   C-C = 0.0078 A
# Refl: sc16005.fcf - Type: LIST4                                    Temp = 170 K
# X-ray MoKa              R(int) = 0.057,  wR2/R(int) =  2.4,  Nref/Npar =  14.0
# Cell  10.1218(4)  21.3189(7)  12.8204(5)          90    92.701(4)          90
# Wavelength 0.71073    Volume Reported   2763.38(18)   Calculated   2763.39(18)
# SpaceGroup from Symmetry P 21/n     Hall: -P 2yn               monoclinic
#                 Reported P 1 21/n 1       -P 2yn               monoclinic
# MoietyFormula C35 H27 I N2 O2
#      Reported C35 H27 I N2 O2
#    SumFormula C35 H27 I N2 O2
#      Reported C35 H27 I N2 O2
# Mr        =    634.49[Calc],    634.48[Rep]
# Dx,gcm-3  =     1.525[Calc],     1.525[Rep]
# Z         =         4[Calc],         4[Rep]
# Mu (mm-1) =     1.195[Calc],     1.195[Rep]
# F000      =    1280.0[Calc],    1280.0[Rep]  or F000' =   1278.56[Calc]
# Reported   T Limits: Tmin=0.922              Tmax=1.000  AbsCorr = MULTI-SCAN
# Calculated T Limits: Tmin=0.899 Tmin'=0.458  Tmax=0.947
# Reported   Hmax= 12, Kmax= 25, Lmax= 15, Nref=   5055        , Th(max)= 25.350
# Obs in FCF Hmax= 12, Kmax= 25, Lmax= 15, Nref=   5055[  5055], Th(max)= 25.350
# Calculated Hmax= 12, Kmax= 25, Lmax= 15, Nref=   5064        , Ratio  =  0.998
# Reported   Rho(min) = -0.84, Rho(max) =  1.14 e/Ang**3 (From CIF)
# Calculated Rho(min) = -0.92, Rho(max) =  1.21 e/Ang**3 (From CIF+FCF data)
# w=1/[sigma**2(Fo**2)+(0.0260P)**2+ 10.0838P], P=(Fo**2+2*Fc**2)/3
# R= 0.0801(  4731), wR2= 0.1345(  5055), S = 1.362      (From CIF+FCF data)
# R= 0.0801(  4731), wR2= 0.1344(  5055), S = 1.361      (From FCF data only)
# R= 0.0801(  4731), wR2= 0.1344(  5055), S = 1.361, Npar=  362
#===============================================================================
For Documentation: http://http://www.platonsoft.nl/CIF-VALIDATION.pdf
#===============================================================================

#===============================================================================
>>> The Following Improvement and Query ALERTS were generated - (Acta-Mode) <<<
#===============================================================================
Format: alert-number_ALERT_alert-type_alert-level text

242_ALERT_2_C Low    'MainMol' Ueq as Compared to Neighbors of        C33 Check
334_ALERT_2_C Small Average Benzene  C-C Dist. C30    -C35           1.37 Ang.
906_ALERT_3_C Large K value in the Analysis of Variance ......     16.466 Check
906_ALERT_3_C Large K value in the Analysis of Variance ......      3.298 Check
910_ALERT_3_C Missing # of FCF Reflection(s) Below Theta(Min)           9 Note
#===============================================================================
063_ALERT_4_G Crystal Size Likely too Large for Beam Size ....       0.65 mm
083_ALERT_2_G SHELXL Second Parameter in WGHT  Unusually Large      10.08 Why ?
978_ALERT_2_G Number C-C Bonds with Positive Residual Density           4 Note
#===============================================================================

ALERT_Level and ALERT_Type Summary
==================================
5 ALERT_Level_C = Check. Ensure it is Not caused by an Omission or Oversight
3 ALERT_Level_G = General Info/Check that it is not Something Unexpected

4 ALERT_Type_2 Indicator that the Structure Model may be Wrong or Deficient.
3 ALERT_Type_3 Indicator that the Structure Quality may be Low.
1 ALERT_Type_4 Improvement, Methodology, Query or Suggestion.
#===============================================================================

0 Missing  Experimental  Info Issue(s) (Out of  62 Tests) -  100 % Satisfied
0 Experimental  Data  Related Issue(s) (Out of  28 Tests) -  100 % Satisfied
5 Structural  Model   Related Issue(s) (Out of 126 Tests) -   96 % Satisfied
3 Unresolved or to be Checked Issue(s) (Out of 247 Tests) -   99 % Satisfied

#===============================================================================
```

Compound (±)-**1Me** CCDC Deposition Number 2244208

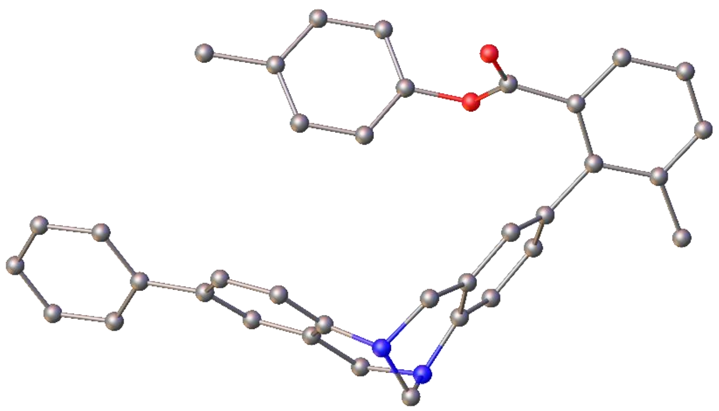

Experimental. Single colourless block-shaped crystals of (**SC16013**) were recrystallised from a mixture of diethyl ether and hexane by slow evaporation. A suitable crystal (0.44×0.34×0.10) mm^3 was selected and mounted on a MITIGEN holder in Paratone oil on a Rigaku Oxford Diffraction SuperNova diffractometer. The crystal was kept at T = 120.0 K during data collection. Using **Olex2** (Dolomanov et al., 2009), the structure was solved with the **ShelXS** (Sheldrick, 2008) structure solution program, using the Direct Methods solution method. The model was refined with version 2016/6 of **ShelXL** (Sheldrick, 2015) using Least Squares minimisation.

Crystal Data. $C_{36}H_{30}N_2O_2$, M_r = 522.62, monoclinic, P2$_1$/c (No. 14), a = 9.9025(10) Å, b = 21.2942(10) Å, c = 16.7874(17) Å, β = 130.354(16)°, α = γ = 90°, V = 2697.6(6) Å^3, T = 120.0 K, Z = 4, Z' = 1, μ(MoK$_\alpha$) = 0.080, 24533 reflections measured, 6733 unique (R_{int} = 0.0455) which were used in all calculations. The final wR_2 was 0.1202 (all data) and R_1 was 0.0581 (I > 2(I)).

**Compound**	**SC16013**
Formula	$C_{36}H_{30}N_2O_2$
$D_{calc.}$/ g cm^{-3}	1.287
μ/mm^{-1}	0.080
Formula Weight	522.62
Colour	colourless
Shape	block
Size/mm^3	0.44×0.34×0.10
T/K	120.0
Crystal System	monoclinic
Space Group	P2$_1$/c
a/Å	9.9025(10)
b/Å	21.2942(10)
c/Å	16.7874(17)
α/°	90
β/°	130.354(16)
γ/°	90
V/Å^3	2697.6(6)
Z	4
Z'	1
Wavelength/Å	0.71073
Radiation type	MoK$_\alpha$
Θ_{min}/°	3.155
Θ_{max}/°	29.687
Measured Refl.	24533
Independent Refl.	6733
Reflections Used	5215
R_{int}	0.0455
Parameters	364
Restraints	0
Largest Peak	0.309
Deepest Hole	-0.268
GooF	1.050
wR_2 (all data)	0.1202
wR_2	0.1102
R_1 (all data)	0.0795
R_1	0.0581

Structure Quality Indicators

**Reflections:**	d min (Mo) 0.72	I/σ 12.9	Rint 4.55%	complete at 2θ=53° 88%
**Refinement:**	Shift -0.001	Max Peak 0.8	Min Peak -0.4	GooF 1.050

A colourless block-shaped crystal with dimensions 0.44×0.34×0.10 mm^3 was mounted on a MITIGEN holder in Paratone oil.X-ray diffraction data were collected using a Rigaku Oxford Diffraction SuperNova diffractometer equipped with a Oxford Cryosystems Cryostream 700+ low-temperature device, operating at T = 120.0 K.

Data were measured using ω scans scans of 1.0 ° per frame for 10.0 s using MoK_{α} radiation (micro-focus sealed X-ray tube, 50 kV, 0.8 mA). The total number of runs and images was based on the strategy calculation from the program CrysAlisPro (Agilent).The maximum resolution achieved was Θ = 29.687. °

Cell parameters were retrieved using the CrysAlisPro (Agilent) software and refined using CrysAlisPro (Agilent) on 6693 reflections, 27 % of the observed reflections. Data reduction was performed using the CrysAlisPro (Agilent) software which corrects for Lorentz polarisation. The final completeness is 99.70 out to 29.687 in Θ. The absorption coefficient μ of this material is 0.080 at this wavelength (λ = 0.71073) and the minimum and maximum transmissions are 0.99566 and 0.99566.

The structure was solved in the space group $P2_1/c$ (# 14) by Direct Methods using the **ShelXS** (Sheldrick, 2008) structure solution program and refined by Least Squares using version 2016/6 of **ShelXL** (Sheldrick, 2015). All non-hydrogen atoms were refined anisotropically. Hydrogen atom positions were calculated geometrically and refined using the riding model.

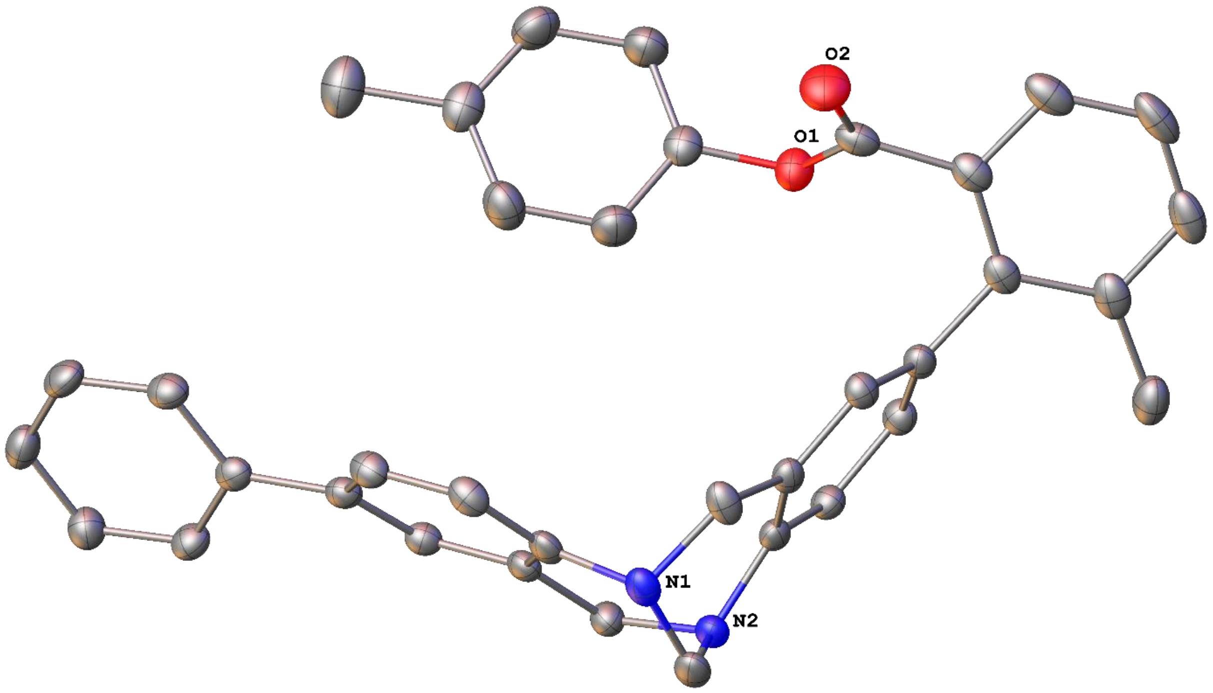

Figure S9: The molecular structure of SC16013. Displacement ellipsoids are at the 50% probability level and H atoms are not shown.

Data Plots: Diffraction Data

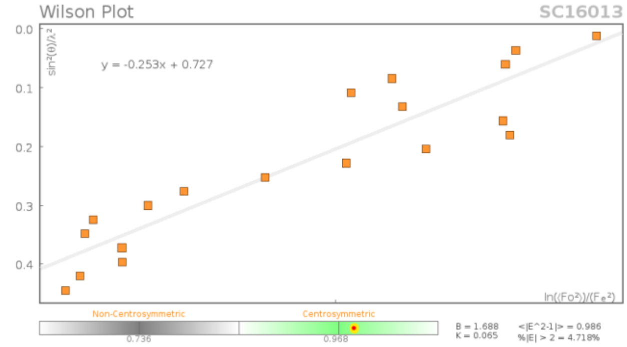

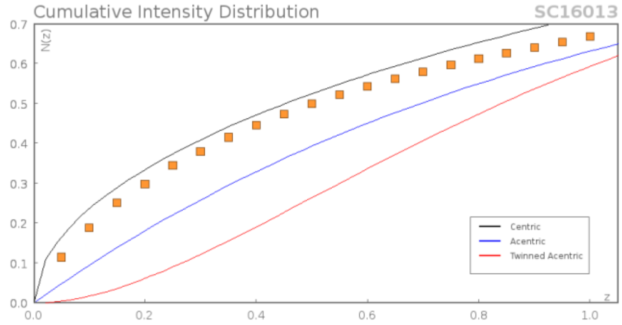

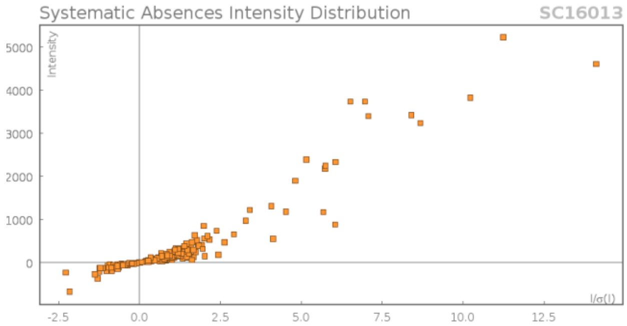

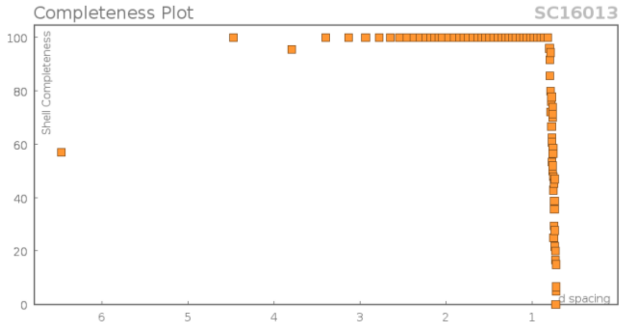

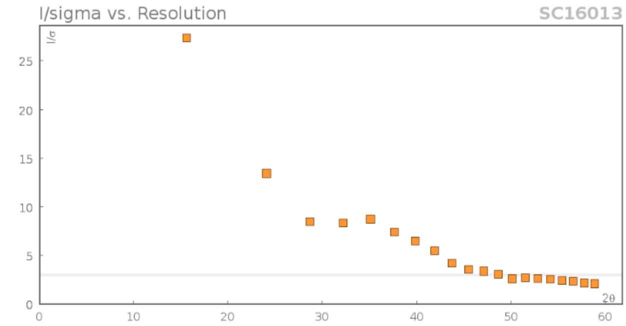

Data Plots: Refinement and Data

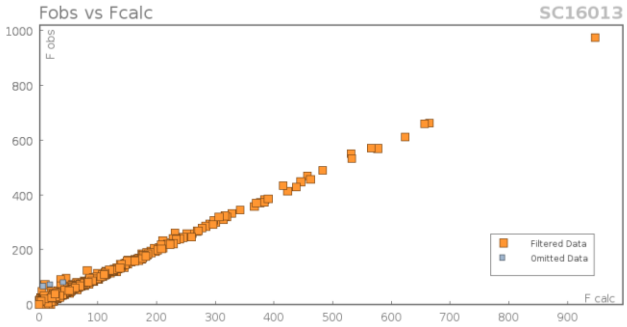

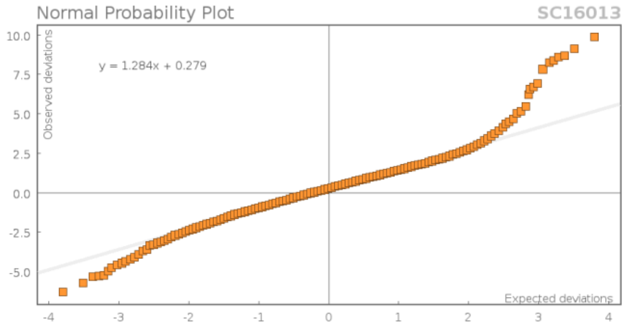

Reflection Statistics

Total reflections (after filtering)	24939	Unique reflections	6733
Completeness	0.882	Mean I/σ	12.93
hkl_{max} collected	(13, 26, 20)	hkl_{min} collected	(-8, -29, -22)
hkl_{max} used	(10, 29, 22)	hkl_{min} used	(-13, 0, 0)
Lim d_{max} collected	100.0	Lim d_{min} collected	0.36
d_{max} used	6.46	d_{min} used	0.72
Friedel pairs	3981	Friedel pairs merged	1

Inconsistent equivalents	6	R_{int}	0.0455
R_{sigma}	0.0493	Intensity transformed	0
Omitted reflections	0	Omitted by user (OMIT hkl)	12
Multiplicity	(9412, 5695, 1231, 102, 6, 1)	Maximum multiplicity	11
Removed systematic absences	394	Filtered off (Shel/OMIT)	0

Table S49: Fractional Atomic Coordinates (×10^4) and Equivalent Isotropic Displacement Parameters (Å^2×10^3) for **SC16013.** U_{eq} is defined as 1/3 of the trace of the orthogonalised U_{ij}.

Atom	x	y	z	U_{eq}
O1	-88.2(15)	6308.0(5)	-1506.7(9)	26.8(3)
O2	-2483.7(17)	5683.9(6)	-2303.9(10)	37.2(3)
N1	283.2(18)	6999.9(7)	2092(1)	23.7(3)
N2	1683.0(18)	7857.7(6)	1915.1(10)	22.1(3)
C1	3477(2)	7589.4(8)	2652.9(12)	22.6(3)
C2	3452(2)	6913.6(7)	2928.1(12)	20.1(3)
C3	1884(2)	6637.9(8)	2606.0(12)	21.4(3)
C4	1899(2)	6012.1(8)	2854.5(12)	24.5(4)
C5	3456(2)	5672.7(8)	3458.6(12)	24.1(4)
C6	5059(2)	5948.4(7)	3836.8(12)	21.1(3)
C7	5011(2)	6564.6(8)	3542.1(12)	20.5(3)
C8	6764(2)	5606.3(8)	4573.8(12)	21.5(3)
C9	8322(2)	5931.4(8)	5343.2(13)	24.6(4)
C10	9918(2)	5621.7(8)	6063.6(13)	28.1(4)
C11	9982(2)	4974.9(8)	6033.7(14)	29.3(4)
C12	8453(2)	4643.9(8)	5277.1(14)	29.0(4)
C13	6864(2)	4952.7(8)	4549.7(13)	26.1(4)
C14	696(2)	7669.7(8)	2249.2(13)	26.2(4)
C15	-1054(2)	6885.2(9)	960.4(12)	24.3(4)
C16	-653(2)	7244.6(7)	353.5(12)	20.0(3)
C17	741(2)	7674.4(7)	852.1(12)	19.8(3)
C18	1170(2)	7959.4(7)	293.1(12)	20.9(3)
C19	157(2)	7853.6(8)	-767.2(12)	21.4(3)
C20	-1322(2)	7457.2(8)	-1296.2(12)	20.1(3)
C21	-1679(2)	7148.6(8)	-719.5(12)	20.4(3)
C22	-2551(2)	7360.3(8)	-2452.7(12)	21.9(3)
C23	-3529(2)	7867.0(9)	-3142.6(13)	26.6(4)
C24	-4863(2)	7736.5(10)	-4206.7(13)	32.7(4)
C25	-5215(2)	7131(1)	-4591.8(13)	35.0(5)
C26	-4228(2)	6635.6(10)	-3929.6(13)	30.5(4)
C27	-2888(2)	6750.7(8)	-2859.5(12)	24.1(4)
C28	-3174(3)	8539.6(9)	-2783.6(15)	33.9(4)
C29	-1851(2)	6189.8(8)	-2204.3(13)	24.9(4)
C30	1026(2)	5816.4(8)	-814.0(13)	25.1(4)
C31	1619(3)	5374.3(9)	-1118.8(15)	34.5(4)
C32	2811(3)	4922.7(9)	-402.2(16)	37.3(5)
C33	3416(2)	4913.9(9)	608.2(15)	31.8(4)
C34	2773(3)	5365.3(10)	885.8(15)	36.7(5)
C35	1591(3)	5821.1(9)	182.5(15)	34.2(4)
C36	4772(3)	4434.5(10)	1398.5(17)	44.3(5)

Table S50: Anisotropic Displacement Parameters (×10^4) **SC16013.** The anisotropic displacement factor exponent takes the form: $-2\pi^2[h^2a^{*2} \times U_{11}+ ... +2hka^* \times b^* \times U_{12}]$

Atom	U_{11}	U_{22}	U_{33}	U_{23}	U_{13}	U_{12}
O1	24.4(6)	24.8(6)	28.8(6)	1.8(5)	16.2(5)	-1.6(5)
O2	35.8(7)	29.5(7)	40.7(8)	-6.3(6)	22.2(6)	-10.6(6)
N1	23.2(7)	32.5(8)	18.8(6)	3.2(6)	15.2(6)	4.3(6)
N2	26.1(7)	23.4(7)	18.0(6)	0.9(5)	14.7(6)	4.4(6)
C1	23.9(8)	20.4(8)	19.3(8)	-0.2(6)	12.1(7)	0.3(7)
C2	24.1(8)	21.0(8)	15.3(7)	-0.8(6)	12.8(7)	-0.7(6)

Atom	U_{11}	U_{22}	U_{33}	U_{23}	U_{13}	U_{12}
C3	23.2(8)	28.7(9)	14.4(7)	0.4(6)	13.1(7)	2.1(7)
C4	23.8(8)	30.4(9)	21.1(8)	-1.2(7)	15.5(7)	-6.0(7)
C5	28.8(9)	21.2(8)	23.1(8)	-1.0(7)	17.2(7)	-4.4(7)
C6	25.4(8)	20.7(8)	19.8(7)	-2.1(6)	15.8(7)	-1.2(7)
C7	21.2(8)	22.0(8)	20.1(7)	-2.4(6)	14.2(7)	-2.7(6)
C8	27.9(8)	20.5(8)	22.2(8)	1.4(6)	19.0(7)	0.2(7)
C9	28.8(9)	18.5(8)	28.4(8)	0.5(7)	19.4(8)	0.1(7)
C10	26.3(9)	28.6(10)	25.9(8)	2.7(7)	15.4(8)	1.4(7)
C11	31.1(9)	29(1)	30.0(9)	8.8(8)	20.8(8)	10.4(8)
C12	40.5(10)	18.2(9)	34.2(9)	3.3(7)	26.9(9)	5.4(8)
C13	32.4(9)	20.8(9)	27.5(9)	-2.1(7)	20.6(8)	-3.0(7)
C14	31.3(9)	32.5(10)	20.0(8)	2.9(7)	19.0(7)	8.5(8)
C15	19.3(8)	36.6(10)	19.9(8)	4.6(7)	14.0(7)	1.3(7)
C16	20.6(8)	22.6(8)	20.2(7)	3.6(6)	14.7(7)	5.2(6)
C17	21.3(8)	20.2(8)	19.1(7)	1.8(6)	13.6(7)	5.7(6)
C18	23.0(8)	17.1(8)	22.7(8)	0.4(6)	14.8(7)	0.3(6)
C19	24.7(8)	20.5(8)	22.4(8)	3.0(6)	16.7(7)	1.7(7)
C20	21.6(8)	22.6(8)	18.9(7)	2.4(6)	14.3(7)	3.8(6)
C21	17.5(8)	23.5(8)	20.0(7)	0.7(6)	12.1(7)	0.3(6)
C22	18.3(8)	32.0(9)	19.8(7)	1.8(7)	14.3(7)	-0.4(7)
C23	23.6(8)	38.5(10)	24.4(8)	7.4(7)	18.6(7)	3.2(7)
C24	24.5(9)	53.7(13)	22.9(8)	12.3(8)	16.7(8)	5.4(9)
C25	22.6(9)	63.8(14)	16.5(8)	1.3(9)	11.7(7)	-4.6(9)
C26	25.8(9)	46.3(12)	22.9(8)	-6.9(8)	17.4(8)	-10.3(8)
C27	22.2(8)	32.2(9)	21.2(8)	-1.8(7)	15.5(7)	-4.8(7)
C28	36.8(10)	35.8(11)	33.4(10)	12.6(8)	24.7(9)	11.1(8)
C29	27.2(9)	30(1)	22.5(8)	-6.7(7)	18.3(7)	-6.9(7)
C30	24.2(8)	22.6(9)	28.0(9)	0.4(7)	16.6(7)	-2.7(7)
C31	40.5(11)	37.3(11)	30.1(9)	-0.1(8)	24.8(9)	4.1(9)
C32	40.9(11)	34.4(11)	43.4(11)	-0.3(9)	30.4(10)	6.8(9)
C33	26.2(9)	29.4(10)	39.7(10)	4.9(8)	21.3(8)	-0.7(8)
C34	36.6(11)	45.7(12)	28.6(9)	6.1(9)	21.5(9)	5.0(9)
C35	38.2(10)	35.7(11)	34.1(10)	-0.2(8)	26.0(9)	4.9(9)
C36	36.0(11)	44.1(13)	51.2(13)	14.7(10)	27.6(10)	6.6(9)

Table S51: Bond Lengths in Å for **SC16013**.

Atom	Atom	Length/Å
O1	C29	1.354(2)
O1	C30	1.416(2)
O2	C29	1.202(2)
N1	C3	1.442(2)
N1	C14	1.460(2)
N1	C15	1.471(2)
N2	C1	1.472(2)
N2	C14	1.463(2)
N2	C17	1.432(2)
C1	C2	1.516(2)
C2	C3	1.400(2)
C2	C7	1.392(2)
C3	C4	1.394(2)
C4	C5	1.380(2)
C5	C6	1.401(2)
C6	C7	1.392(2)
C6	C8	1.486(2)
C8	C9	1.395(2)
C8	C13	1.398(2)
C9	C10	1.386(2)
C10	C11	1.381(3)
C11	C12	1.383(3)
C12	C13	1.382(2)

Atom	Atom	Length/Å
C15	C16	1.519(2)
C16	C17	1.395(2)
C16	C21	1.396(2)
C17	C18	1.395(2)
C18	C19	1.383(2)
C19	C20	1.400(2)
C20	C21	1.392(2)
C20	C22	1.494(2)
C22	C23	1.411(2)
C22	C27	1.403(2)
C23	C24	1.399(2)
C23	C28	1.505(3)
C24	C25	1.382(3)
C25	C26	1.377(3)
C26	C27	1.400(2)
C27	C29	1.493(2)
C30	C31	1.372(3)
C30	C35	1.378(3)
C31	C32	1.388(3)
C32	C33	1.386(3)
C33	C34	1.390(3)
C33	C36	1.516(3)
C34	C35	1.384(3)

Table S52: Bond Angles in ° for **SC16013**.

**Atom**	**Atom**	**Atom**	**Angle/°**
C29	O1	C30	117.08(13)
C3	N1	C14	110.09(13)
C3	N1	C15	113.93(13)
C14	N1	C15	107.94(13)
C14	N2	C1	108.25(13)
C17	N2	C1	113.59(13)
C17	N2	C14	110.20(13)
N2	C1	C2	112.07(13)
C3	C2	C1	120.87(14)
C7	C2	C1	120.22(14)
C7	C2	C3	118.86(15)
C2	C3	N1	121.42(15)
C4	C3	N1	118.96(15)
C4	C3	C2	119.45(15)
C5	C4	C3	120.71(15)
C4	C5	C6	120.85(16)
C5	C6	C8	121.03(15)
C7	C6	C5	117.77(15)
C7	C6	C8	121.12(14)
C2	C7	C6	122.21(15)
C9	C8	C6	120.45(15)
C9	C8	C13	117.67(15)
C13	C8	C6	121.84(15)
C10	C9	C8	121.62(16)
C11	C10	C9	119.73(17)
C10	C11	C12	119.55(16)
C13	C12	C11	120.79(16)
C12	C13	C8	120.62(16)
N1	C14	N2	112.10(13)
N1	C15	C16	112.02(14)
C17	C16	C15	121.00(14)
C17	C16	C21	118.76(15)
C21	C16	C15	120.24(15)
C16	C17	N2	121.30(14)
C18	C17	N2	118.90(14)
C18	C17	C16	119.71(14)
C19	C18	C17	120.77(15)
C18	C19	C20	120.24(15)
C19	C20	C22	123.01(14)
C21	C20	C19	118.41(14)
C21	C20	C22	118.57(14)
C20	C21	C16	121.84(15)
C23	C22	C20	120.63(15)
C27	C22	C20	119.81(15)
C27	C22	C23	119.18(15)
C22	C23	C28	122.75(15)
C24	C23	C22	118.35(17)
C24	C23	C28	118.89(16)
C25	C24	C23	121.75(17)
C26	C25	C24	120.32(16)
C25	C26	C27	119.28(18)
C22	C27	C29	123.26(14)
C26	C27	C22	121.05(16)
C26	C27	C29	115.69(16)
O1	C29	C27	112.04(14)
O2	C29	O1	123.42(16)
O2	C29	C27	124.48(16)
C31	C30	O1	119.68(16)
C31	C30	C35	121.47(17)
C35	C30	O1	118.68(16)
C30	C31	C32	118.79(17)
C33	C32	C31	121.40(18)
C32	C33	C34	118.20(17)
C32	C33	C36	121.06(18)
C34	C33	C36	120.73(18)
C35	C34	C33	121.11(18)
C30	C35	C34	119.02(18)

Table S53: Torsion Angles in ° for **SC16013**.

**Atom**	**Atom**	**Atom**	**Atom**	**Angle/°**
O1	C30	C31	C32	175.39(16)
O1	C30	C35	C34	-175.81(16)
N1	C3	C4	C5	-171.92(14)
N1	C15	C16	C17	-6.1(2)
N1	C15	C16	C21	173.86(14)
N2	C1	C2	C3	-8.2(2)
N2	C1	C2	C7	174.25(14)
N2	C17	C18	C19	-172.11(14)
C1	N2	C14	N1	-70.00(16)
C1	N2	C17	C16	106.95(17)
C1	N2	C17	C18	-76.60(18)
C1	C2	C3	N1	-6.1(2)
C1	C2	C3	C4	178.77(14)
C1	C2	C7	C6	178.29(14)
C2	C3	C4	C5	3.4(2)

Atom	Atom	Atom	Atom	Angle/˚
C3	N1	C14	N2	54.76(17)
C3	N1	C15	C16	-80.12(18)
C3	C2	C7	C6	0.7(2)
C3	C4	C5	C6	0.0(2)
C4	C5	C6	C7	-2.9(2)
C4	C5	C6	C8	173.80(15)
C5	C6	C7	C2	2.6(2)
C5	C6	C8	C9	-146.41(16)
C5	C6	C8	C13	31.3(2)
C6	C8	C9	C10	177.67(16)
C6	C8	C13	C12	-176.83(16)
C7	C2	C3	N1	171.46(14)
C7	C2	C3	C4	-3.7(2)
C7	C6	C8	C9	30.2(2)
C7	C6	C8	C13	-152.04(16)
C8	C6	C7	C2	-174.17(14)
C8	C9	C10	C11	-0.7(3)
C9	C8	C13	C12	1.0(2)
C9	C10	C11	C12	0.7(3)
C10	C11	C12	C13	0.1(3)
C11	C12	C13	C8	-1.0(3)
C13	C8	C9	C10	-0.2(3)
C14	N1	C3	C2	-16.33(19)
C14	N1	C3	C4	158.84(14)
C14	N1	C15	C16	42.47(18)
C14	N2	C1	C2	43.27(17)
C14	N2	C17	C16	-14.7(2)
C14	N2	C17	C18	161.75(14)
C15	N1	C3	C2	105.07(17)
C15	N1	C3	C4	-79.75(18)
C15	N1	C14	N2	-70.16(16)
C15	C16	C17	N2	-8.8(2)
C15	C16	C17	C18	174.78(15)
C15	C16	C21	C20	-178.31(15)
C16	C17	C18	C19	4.4(2)
C17	N2	C1	C2	-79.46(17)
C17	N2	C14	N1	54.76(17)
C17	C16	C21	C20	1.6(2)
C17	C18	C19	C20	0.0(2)
C18	C19	C20	C21	-3.5(2)
C18	C19	C20	C22	175.27(15)
C19	C20	C21	C16	2.7(2)
C19	C20	C22	C23	-62.1(2)
C19	C20	C22	C27	124.99(18)
C20	C22	C23	C24	-169.97(15)
C20	C22	C23	C28	11.2(2)
C20	C22	C27	C26	170.23(15)
C20	C22	C27	C29	-10.9(2)
C21	C16	C17	N2	171.26(14)
C21	C16	C17	C18	-5.2(2)
C21	C20	C22	C23	116.72(18)
C21	C20	C22	C27	-56.2(2)
C22	C20	C21	C16	-176.16(15)
C22	C23	C24	C25	-1.2(3)
C22	C27	C29	O1	-43.1(2)

Atom	Atom	Atom	Atom	Angle/°
C22	C27	C29	O2	139.58(18)
C23	C22	C27	C26	-2.8(2)
C23	C22	C27	C29	176.05(15)
C23	C24	C25	C26	-1.0(3)
C24	C25	C26	C27	1.2(3)
C25	C26	C27	C22	0.7(3)
C25	C26	C27	C29	-178.27(16)
C26	C27	C29	O1	135.82(15)
C26	C27	C29	O2	-41.5(2)
C27	C22	C23	C24	3.0(2)
C27	C22	C23	C28	-175.87(16)
C28	C23	C24	C25	177.75(17)
C29	O1	C30	C31	86.1(2)
C29	O1	C30	C35	-98.53(19)
C30	O1	C29	O2	-5.0(2)
C30	O1	C29	C27	177.66(14)
C30	C31	C32	C33	-0.5(3)
C31	C30	C35	C34	-0.6(3)
C31	C32	C33	C34	1.1(3)
C31	C32	C33	C36	-177.63(19)
C32	C33	C34	C35	-1.5(3)
C33	C34	C35	C30	1.2(3)
C35	C30	C31	C32	0.2(3)
C36	C33	C34	C35	177.25(18)

Table S54: Hydrogen Fractional Atomic Coordinates (×10[4]) and Equivalent Isotropic Displacement Parameters (Å^2×10^3) for **SC16013**. U_{eq} is defined as 1/3 of the trace of the orthogonalised U_{ij}.

Atom	x	y	z	U_{eq}
H1A	4195.8	7844.64	3299.73	27
H1B	4046.22	7608.88	2339.62	27
H4	824.4	5817.17	2605.28	29
H5	3441.04	5246.5	3620.12	29
H7	6077.34	6753.14	3767.77	25
H9	8287.89	6376.06	5373.98	29
H10	10964.63	5853.3	6575.89	34
H11	11068.41	4758.92	6529.64	35
H12	8494.9	4198.92	5256.86	35
H13	5830.16	4718.45	4028.49	31
H14A	1403.44	7771.48	2999.01	31
H14B	-421.53	7912.47	1850	31
H15A	-2233.52	7012.48	711.7	29
H15B	-1097.78	6430.29	824.01	29
H18	2170.43	8229.24	644.85	25
H19	464.76	8050.63	-1138.07	26
H21	-2648.68	6864.64	-1065.91	24
H24	-5545.41	8072.98	-4676.99	39
H25	-6141.41	7056.13	-5316.85	42
H26	-4453.43	6220.25	-4195.83	37
H28A	-2184.02	8700.71	-2723.23	51
H28B	-2872.43	8563.76	-2101.39	51
H28C	-4234.24	8792.49	-3292.23	51
H31	1220.39	5377.17	-1807.73	41
H32	3221.41	4613.04	-608.7	45
H34	3149.97	5360.96	1569.82	44
H35	1176.32	6132.51	383.8	41
H36A	5933.35	4636.55	1891.06	66
H36B	4841.37	4093.5	1034.29	66
H36C	4416.74	4263.47	1780.87	66

```
#==============================================================================
# PLATON/CHECK-(150616) versus check.def version of 160610 for Entry: sc16013
# Data: SC16013.cif - Type: CIF                  Bond Precision    C-C = 0.0027 A
# Refl: SC16013.fcf - Type: LIST4                                   Temp = 120 K
# X-ray MoKa              R(int) = 0.045,  wR2/R(int) =  2.6,  Nref/Npar =  18.5
# Cell  9.9025(10) 21.2942(10) 16.7874(17)           90  130.354(16)          90
# Wavelength 0.71073    Volume Reported     2697.6(6)   Calculated     2697.6(8)
# SpaceGroup from Symmetry P 21/c     Hall: -P 2ybc              monoclinic
#                 Reported P 1 21/c 1       -P 2ybc              monoclinic
# MoietyFormula C36 H30 N2 O2
#      Reported C36 H30 N2 O2
#    SumFormula C36 H30 N2 O2
#      Reported C36 H30 N2 O2
# Mr        =    522.62[Calc],    522.62[Rep]
# Dx,gcm-3  =     1.287[Calc],     1.287[Rep]
# Z         =         4[Calc],         4[Rep]
# Mu (mm-1) =     0.080[Calc],     0.080[Rep]
# F000      =    1104.0[Calc],    1104.0[Rep]  or F000' =   1104.44[Calc]
# Reported   T Limits: Tmin=0.996             Tmax=0.996  AbsCorr = MULTI-SCAN
# Calculated T Limits: Tmin=0.968 Tmin'=0.966  Tmax=0.992  Exti    =     0.00170
# Reported   Hmax= 13, Kmax= 29, Lmax= 22, Nref=   6733        , Th(max)= 29.687
# Obs in FCF Hmax= 13, Kmax= 29, Lmax= 22, Nref=   6733[  6733], Th(max)= 29.687
# Calculated Hmax= 13, Kmax= 29, Lmax= 23, Nref=   7640        , Ratio  =  0.881
# Reported   Rho(min) = -0.27, Rho(max) =  0.31 e/Ang**3 (From CIF)
# Calculated Rho(min) = -0.28, Rho(max) =  0.30 e/Ang**3 (From CIF+FCF data)
# w=1/[sigma**2(Fo**2)+(0.0320P)**2+  1.5813P], P=(Fo**2+2*Fc**2)/3
# R= 0.0580(  5213), wR2= 0.1202(  6733), S = 1.050      (From CIF+FCF data)
# R= 0.0580(  5213), wR2= 0.1202(  6733), S = 1.050      (From FCF data only)
# R= 0.0581(  5215), wR2= 0.1202(  6733), S = 1.050, Npar=  364
#==============================================================================
For Documentation: http://http://www.platonsoft.nl/CIF-VALIDATION.pdf
#==============================================================================

#==============================================================================
>>> The Following Improvement and Query ALERTS were generated - (Acta-Mode) <<<
#==============================================================================
Format: alert-number_ALERT_alert-type_alert-level text

906_ALERT_3_C Large K value in the Analysis of Variance ......      8.548 Check
906_ALERT_3_C Large K value in the Analysis of Variance ......      2.021 Check
910_ALERT_3_C Missing # of FCF Reflection(s) Below Theta(Min)           9 Note
911_ALERT_3_C Missing # FCF Refl Between THmin & STh/L=  0.600          4 Report
#==============================================================================
128_ALERT_4_G Alternate Setting for Input Space Group  P21/c        P21/n Note
152_ALERT_1_G The Supplied and Calc. Volume s.u. Differ by ...          2 Units
912_ALERT_4_G Missing # of FCF Reflections Above STh/L=  0.600        880 Note
913_ALERT_3_G Missing # of Very Strong Reflections in FCF ....          1 Note
978_ALERT_2_G Number C-C Bonds with Positive Residual Density          14 Note
#==============================================================================

ALERT_Level and ALERT_Type Summary
==================================
4 ALERT_Level_C = Check. Ensure it is Not caused by an Omission or Oversight
5 ALERT_Level_G = General Info/Check that it is not Something Unexpected

1 ALERT_Type_1 CIF Construction/Syntax Error, Inconsistent or Missing Data.
1 ALERT_Type_2 Indicator that the Structure Model may be Wrong or Deficient.
5 ALERT_Type_3 Indicator that the Structure Quality may be Low.
2 ALERT_Type_4 Improvement, Methodology, Query or Suggestion.
#==============================================================================

0 Missing  Experimental  Info Issue(s) (Out of  62 Tests) -  100 % Satisfied
0 Experimental  Data  Related Issue(s) (Out of  28 Tests) -  100 % Satisfied
3 Structural  Model   Related Issue(s) (Out of 126 Tests) -   98 % Satisfied
5 Unresolved or to be Checked Issue(s) (Out of 247 Tests) -   98 % Satisfied
```

#==

S4. Supporting References

1. B. W. Gung, B. U. Emenike, M. Lewis, K. Kirschbaum. Quantification of CH···π Interactions: Implications on How Substituent Effects Influence Aromatic Interactions. *Chem. Eur. J.,* **16**, 12357–12362 (2010).

2. M. Bauer, A. Bertario, G. Boccardi, X. Fontaine, R. Rao, D. Verrier. Reproducibility of ^{1}H-NMR integrals: a collaborative study. *J. Pharma. Biomed. Anal.* **17**, 419–425 (1998).

3. *Gaussian '09*. M. J. Frisch, G. W. Trucks, H. B. Schlegel, G. E. Scuseria, M. A. Robb, J. R. Cheeseman, G. Scalmani, V. Barone, G. A. Petersson, H. Nakatsuji, X. Li, M. Caricato, A. Marenich, J. Bloino, B. G. Janesko, R. Gomperts, B. Mennucci, H. P. Hratchian, J. V. Ortiz, A. F. Izmaylov, J. L. Sonnenberg, D. Williams-Young, F. Ding, F. Lipparini, F. Egidi, J. Goings, B. Peng, A. Petrone, T. Henderson, D. Ranasinghe, V. G. Zakrzewski, J. Gao, N. Rega, G. Zheng, W. Liang, M. Hada, M. Ehara, K. Toyota, R. Fukuda, J. Hasegawa, M. Ishida, T. Nakajima, Y. Honda, O. Kitao, H. Nakai, T. Vreven, K. Throssell, J. A. Montgomery, Jr., J. E. Peralta, F. Ogliaro, M. Bearpark, J. J. Heyd, E. Brothers, K. N. Kudin, V. N. Staroverov, T. Keith, R. Kobayashi, J. Normand, K. Raghavachari, A. Rendell, J. C. Burant, S. S. Iyengar, J. Tomasi, M. Cossi, J. M. Millam, M. Klene, C. Adamo, R. Cammi, J. W. Ochterski, R. L. Martin, K. Morokuma, O. Farkas, J. B. Foresman, and D. J. Fox, Gaussian, Inc., Wallingford CT (2016).

4. S. Spicher, S. Grimme. Single-Point Hessian Calculations for Improved Vibrational Frequencies and Rigid-Rotor-Harmonic-Oscillator Thermodynamics. *J. Chem. Theory Comput.*, **17** (3), 1701–1714 (2021).

5. Psi4: An open-source *ab initio* electronic structure program, J. M. Turney, A. C. Simmonett, R. M. Parrish, E. G. Hohenstein, F. Evangelista, J. T. Fermann, B. J. Mintz, L. A. Burns, J. J. Wilke, M. L. Abrams, N. J. Russ, M. L. Leininger, C. L. Janssen, E. T. Seidl, W. D. Allen, H. F. Schaefer, R. A. King, E. F. Valeev, C. D. Sherrill, T. D. Crawford. *WIREs Comput. Mol. Sci.,* **2**, 556 (2012).

6. T. M. Parker, L. A. Burns, R. M. Parrish, A. G. Ryno, C. D. Sherrill. Levels of Symmetry Adapted Perturbation Theory (SAPT). I. Efficiency and Performance for Interaction Energies. *J. Chem. Phys.*, **140** (9), 094106 (2014).

7. C. Bannwarth, E. Caldeweyher, S. Ehlert, A. Hansen, P. Pracht, J. Seibert, S. Spicher, S. Grimme. Extended tight-binding quantum chemistry methods. *WIREs Comput. Mol. Sci.*, **11**, e01493 (2021).

8. O. V. Dolomanov, L. J. Bourhis, R. J. Gildea, J. A. K. Howard, H. Puschmann, Olex2: A complete structure solution, refinement and analysis program, *J. Appl. Cryst.*, **42**, 339-341 (2009).

9. G. M. Sheldrick, A short history of ShelX, *Acta Cryst.*, **A64**, 339-341 (2008).

10. G. M. Sheldrick, Crystal structure refinement with ShelXL, *Acta Cryst.*, **C27**, 3-8 (2015).
